# Reversibility and reactivity in an acid catalyzed cyclocondensation to give furanochromanes – a reaction at the ‘oxonium-Prins’ *vs.* ‘*ortho*-quinone methide cycloaddition’ mechanistic nexus†
†Electronic supplementary information (ESI) available: C. D.-T. Nielsen, W. J. Mooij, D. Sale, H. S. Rzepa, J. Burés and A. C. Spivey, FAIR data archives, Imperial College Research Computing Services data repository, 2018, DOI: 10.14469/hpc/3943 and sub-collections therein. See DOI: 10.1039/c8sc04302g


**DOI:** 10.1039/c8sc04302g

**Published:** 2018-10-19

**Authors:** Christian D.-T. Nielsen, Wouter J. Mooij, David Sale, Henry S. Rzepa, Jordi Burés, Alan C. Spivey

**Affiliations:** a Department of Chemistry , Imperial College London , Exhibition Road , London , SW7 2AZ , UK . Email: a.c.spivey@imperial.ac.uk; b Process Studies Group , Syngenta , Jealott's Hill , Bracknell , Berkshire RG42 6EY , UK; c School of Chemistry , University of Manchester , Oxford Road , Manchester , M13 9PL , UK

## Abstract

Herein we report a combined experimental and computational investigation of the acid catalyzed cyclocondensation reaction between styrenyl homoallylic alcohols and salicylaldehyde to form furanochromanes. We disclose a previously unreported isomerisation of the ‘unnatural’ *trans*-fused products to the diastereomeric ‘natural’ *cis*-fused congeners. Notwithstanding the appeal of assuming this corresponds to *endo* to *exo* isomerisation of Diels–Alder (D–A) adducts *via* concerted retro-cycloaddition/cycloaddition reactions of an *in situ* generated *ortho*-quinone methide with the styrenyl alkene, our combined Hammett/DFT study reveals a stepwise Prins-like process *via* discrete benzylic carbocation intermediates for all but the most electron deficient styrenes. As these reactions fortuitously lie at the intersection of these two mechanistic manifolds, it allows us to propose an experimentally determined indicative *ρ*^+^ value of *ca.* –3 as marking this nexus between a stepwise Prins-type pathway and a concerted cycloaddition reaction. This value should prove useful for categorising other reactions formally involving ‘*ortho*-quinomethides’, without the need for the extensive computation performed here. Logical optimisation of the reaction based upon the mechanistic insight led to the use of HFIP as an additive which enables exclusive formation of ‘natural’ *cis*-fused products with a ∼100-fold reaction rate increase and improved scope.

## Abstract 2

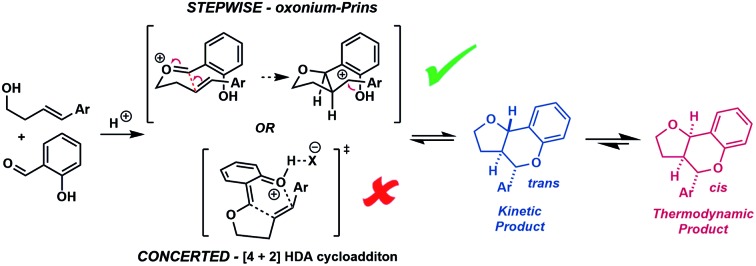
Herein we report a combined experimental and computational investigation of the acid catalyzed cyclocondensation reaction between styrenyl homoallylic alcohols and salicylaldehyde to form furanochromanes.

## Introduction

**Scheme 1:**
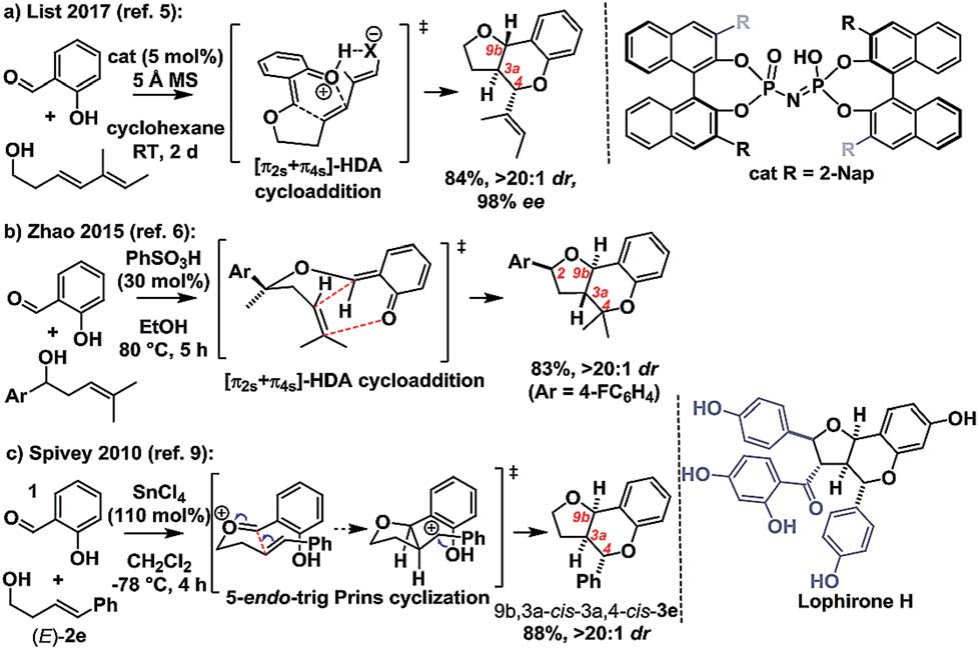
Furanochromane forming reactions.

*Ortho*-quinone methides (*o*QMs) are useful intermediates in synthesis.^1^ Answering the call of Pettus in his 2002 review of their synthetic utility, these “underdeveloped and underutilized”^2^ intermediates have enjoyed a significant revival in the literature since then. In particular, legions of new acid catalyzed cyclocondensations have been reported as being *o*QM cycloaddition processes.^3^,^4^ A case in point is the reaction between salicylaldehyde and homoallylic alcohols, featuring C–C and C–O bond formation, to furnish furanochromanes (Scheme 1).

**Scheme 2:**
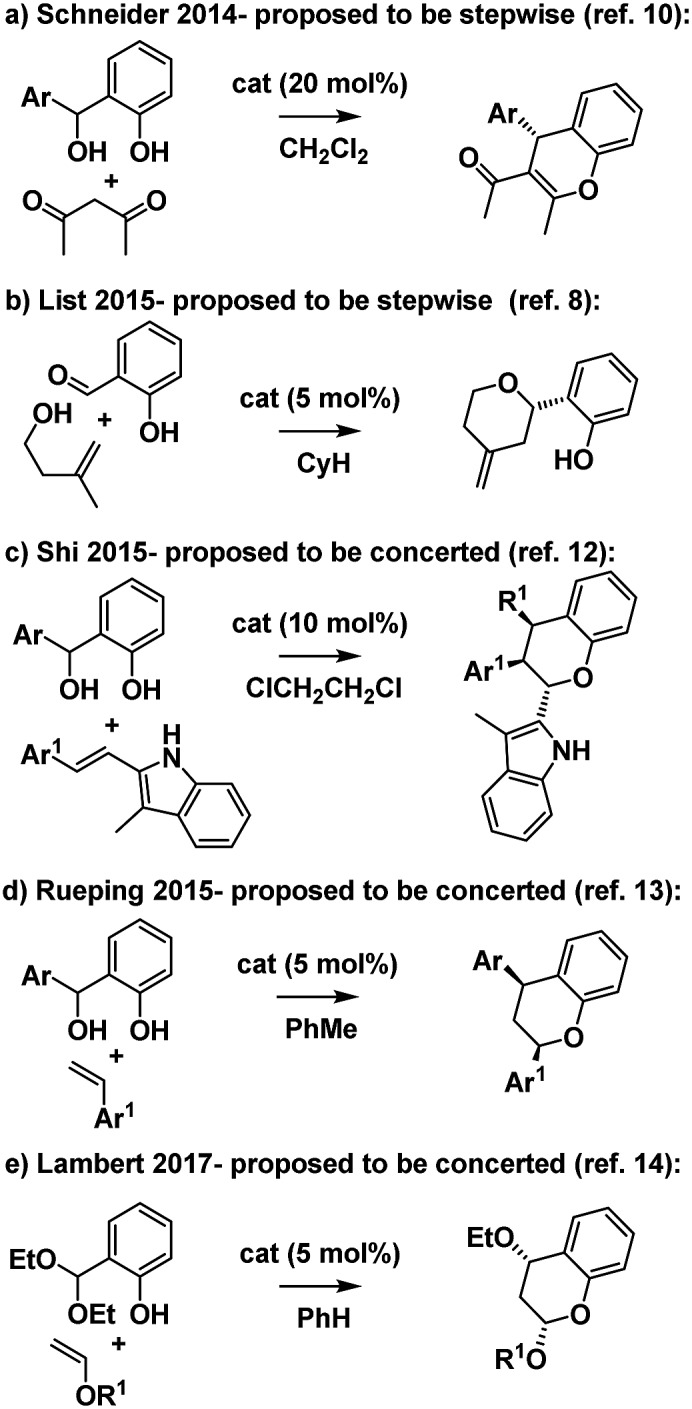
Related Brønsted acid catalyzed hydropyran forming reactions.

Specifically, Brønsted acids have been shown to give *trans*-fused furanochromanes, putatively *via* either a protonated or neutral *o*QM cycloaddition process (Scheme 1a and b).^5^,^6^ Although List *et al.* had proposed a Prins-type mechanism for related reactions with other aldehydes (*cf.*Scheme 2b),^7^,^8^ in this case they cited the stereochemical fidelity by which (*E*)- and (*Z*)-4-phenylbut-3-en-1-ol (**2e**) converted to only 3a,4-*trans* or 3a,4-*cis* products **3e**, respectively, as experimental support for a concerted mechanism. However, faithful translation of double bond stereochemistry could be an artefact arising from the rate of carbocation trapping exceeding the rate of C_3a_–C_4_ bond rotation rather than implicating a concerted *o*QM cycloaddition pathway. In our Lewis acid promoted reactions (Scheme 1c), we proposed a stepwise oxonium-Prins pathway in which a benzylic carbocation is trapped by the phenolic hydroxyl group, as supported experimentally by competitive trapping of the intermediate cation by bromine when using SnBr_4_ in the absence of the intramolecular nucleophile.^9^

Intrigued by these mechanistic discrepancies and cognizant of both the increasing number of related Brønsted acid catalyzed reactions and the ambiguity that remains between a concerted *o*QM cycloaddition pathway and a stepwise carbocation-mediated alternative for a variety of reactions (*e.g.*Scheme 2),^2^,^10^–^14^ we decided to investigate further.

## Results and discussion

Initially, we planned to distinguish between the stepwise and concerted pathways by carrying out a Singleton ^13^C KIE at natural abundance experiment, recovering (*E*)-4-(4-tolyl)-but-3-en-1-ol [(*E*)-**2c**] from a reaction driven close to completion.^15^,^16^ However, DFT calculations revealed that the expected ratios for both pathways would be indistinguishable due to the highly asynchronous nature of the computed *o*QM cycloaddition transition state (TS). An analogous situation was reported by List when studying the chiral Brønsted acid catalyzed formation of dihydropyran from benzaldehydes and dienes in which the isotopic distribution in the product was inconclusive by virtue of being consistent with both stepwise carbocation-mediated and highly asynchronous cycloaddition pathways.^17^

**Scheme 3:**
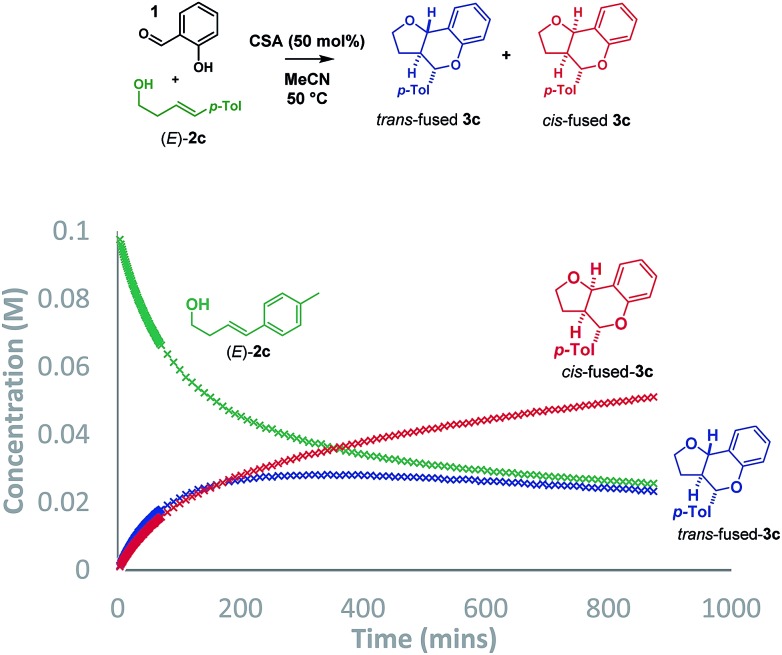
Plot of reaction progress.

As such, we sought an alternative method to probe the mechanism and opted to collect reaction progress data by ^1^H NMR for the Brønsted acid promoted reaction of salicylaldehyde (**1**) with (*E*)-**2c**. The conditions described by List using *p*-TSA (10 mol%) in cyclohexane afforded a ∼2 : 1 mixture of *trans* : *cis*-fused isomers at the ring-junction as reported,^5^ but the conditions were inhomogeneous. Alternatively, a reaction mixture having (±)-camphorsulfonic acid (CSA, 50 mol%) in acetonitrile at 50 °C was homogeneous and allowed us to monitor the reaction progress by ^1^H NMR to 74% conversion in 14.5 h (Scheme 3).

Intriguingly, although the reaction was initially moderately *trans*-ring junction selective (*cf.* initial rates in the 0–50 min region), it was apparent that a thermodynamically driven *trans* to *cis* isomerization was taking place leading to progressive enrichment in the latter as the reaction progressed. DFT analysis (B3LYP+GD3BJ/Def2-TZVPP) suggested that *cis*-fused **3c** is 3.9 kcal mol^–1^ lower in free energy than *trans*-fused **3c**. This thermodynamic difference likely has a component attributable to a destabilizing 4 electron n_O_ ↔ σ_C_9b_–H_ interaction in the *trans*-fused structure. Indeed, the prevalence of 5,6-*cis*-fused systems in bioactive natural products (*e.g.* flavonoids: lophirone H^18^ and cordigol;^19^ and pterocarpan isoflavonoids: medicarpin^20^ and phaseolin^21^) suggests their relative thermodynamic stability. Irrespective of the thermodynamic basis of the phenomenon, a reversible isomerization process renders Singleton analysis unsuitable for interrogation of the mechanism of this reaction.

**Scheme 4:**
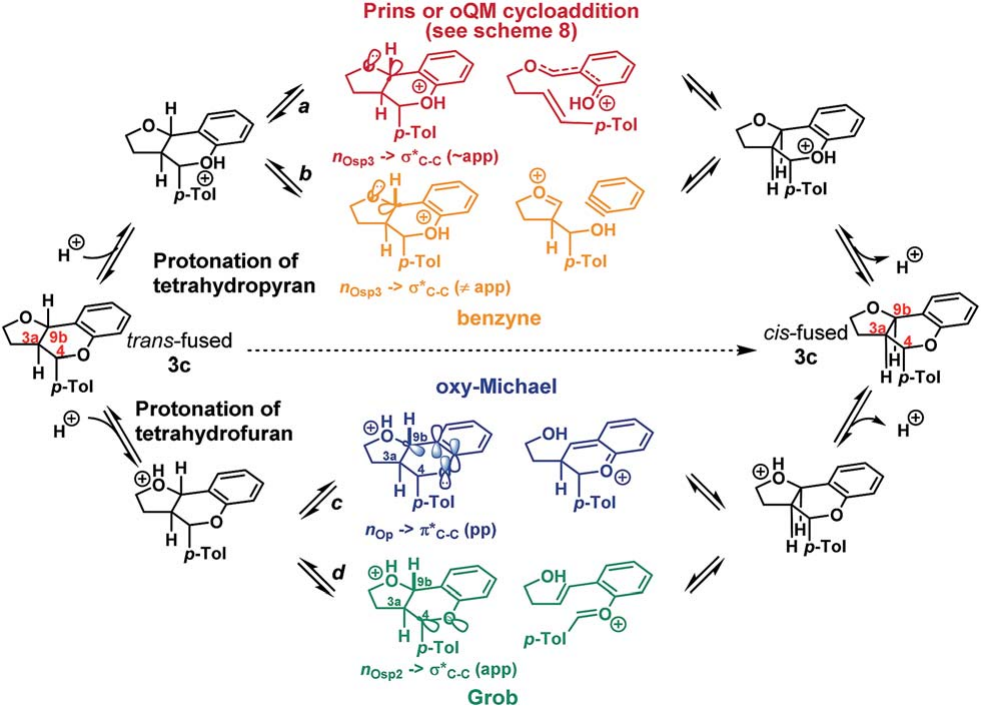
Potential pathways for isomerization.

We envisioned that isomerization was most likely occurring by reversion of the forwards reaction: *i.e.* either *via* a retro-Prins/Prins pathway or a retro-cycloaddition/cycloaddition pathway (path a, Scheme 4), following protonation of the tetrahydropyranyl oxygen. Fragmentation-recombination *via* a benzyne (path b, Scheme 4) from this same protonated species appeared unlikely given the poor n_O_ → σ* orbital alignment for cleavage of the C_9b_–C_Ar_ bond, but could not be entirely discounted. Alternatively, protonation of the furan could facilitate formation of the *cis*-fused product *via* a retro-oxy-Michael/oxy-Michael pathway (path c, Scheme 4) or a Grob fragmentation-bicyclisation pathway (path d, Scheme 4).^22^

**Scheme 5:**
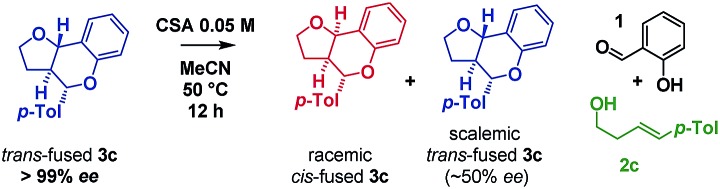
Isomerization study on enantiopure furanochromane monitored by CSP-HPLC.

Inspired by the work of Rychnovsky,^23^ we anticipated that racemization would be a key indicator of mechanism as pathways a and d proceed *via* achiral intermediates, whereas pathways b and c proceed *via* intermediates which retain two stereocentres from the substrate. To test this, we isolated the enantiomerically pure *trans*-fused **3c** and subjected it to the reaction conditions (Scheme 5).

After 12 h, analysis revealed the formation of racemic *cis*-fused **3c** as well as partially racemized recovered *trans*-fused **3c** (∼50% ee). This indicated that the isomerization occurs *via* an achiral intermediate which can convert to the thermodynamically preferred *cis*-fused isomer but also revert to the *trans*-fused isomer and implicates the Prins/*o*QM cycloaddition and/or Grob mechanisms as operating (paths a and d).

**Scheme 6:**
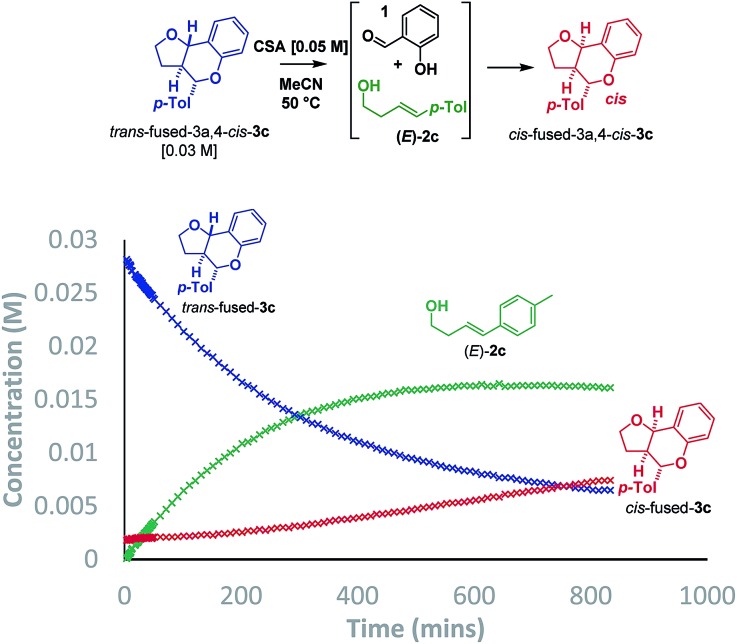
Isomerization study by ^1^H NMR.

Additionally, two new peaks were observed by CSP-HPLC; these were identified as the salicylaldehyde (**1**) and homoallylic alcohol (*E*)-**2c**. The formation of these compounds rules out the Grob fragmentation pathway (path d), for which adventitious hydrolysis of the intermediate oxonium ion would lead to *p*-tolualdehyde and 2-[(1*E*)-4-hydroxy-1-buten-1-yl]phenol (which were not observed). All evidence therefore pointed towards a mechanism of isomerization *via* either a retro-Prins/Prins pathway or a retro-cycloaddition/cycloaddition pathway (path a) as originally anticipated. To corroborate this, ^1^H NMR reaction progress analysis monitoring the isomerization of isolated *trans*-fused **3c**^22^ was performed (Scheme 6).

In agreement with the racemization study, a build-up of homoallylic alcohol (*E*)-**2c** was observed over a period of 15 h as the concentration of the *trans*-fused isomer reduced and that of the *cis*-fused isomer increased. It was also possible to isolate (*E*)-**2c** from a reaction mixture wherein the *trans*-fused diastereomer was partially isomerized.

**Scheme 7:**
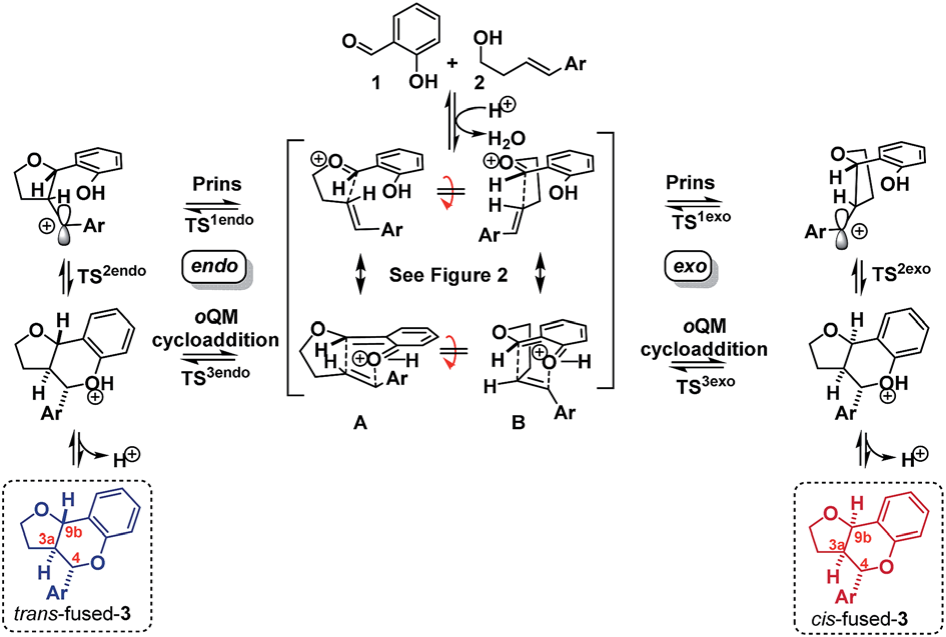
‘*Endo*’ *vs.* ‘*exo*’ transition states.

Having established that the isomerization, like the furanochromane formation itself, takes place *via* either a Prins or *o*QM cycloaddition pathway, we set out to distinguish between these possibilities. Inspection of the oxonium ion/*ortho*-quinone methide conformations required to access the isomeric products *via* either mechanistic manifold reveals that the *trans*-fused products are formed from *endo*-like conformation **A**, whereas the *cis* fused products are formed from *exo*-like conformation **B** (Scheme 7).

The *endo* conformer **A** appears poised for π–π stacking interactions whereas the *exo* conformer **B** is not, suggesting that ‘secondary orbital overlap’, as widely invoked for D–A reactions, could take place in the *endo* TSs, accounting for the kinetic preference.

**Scheme 8:**
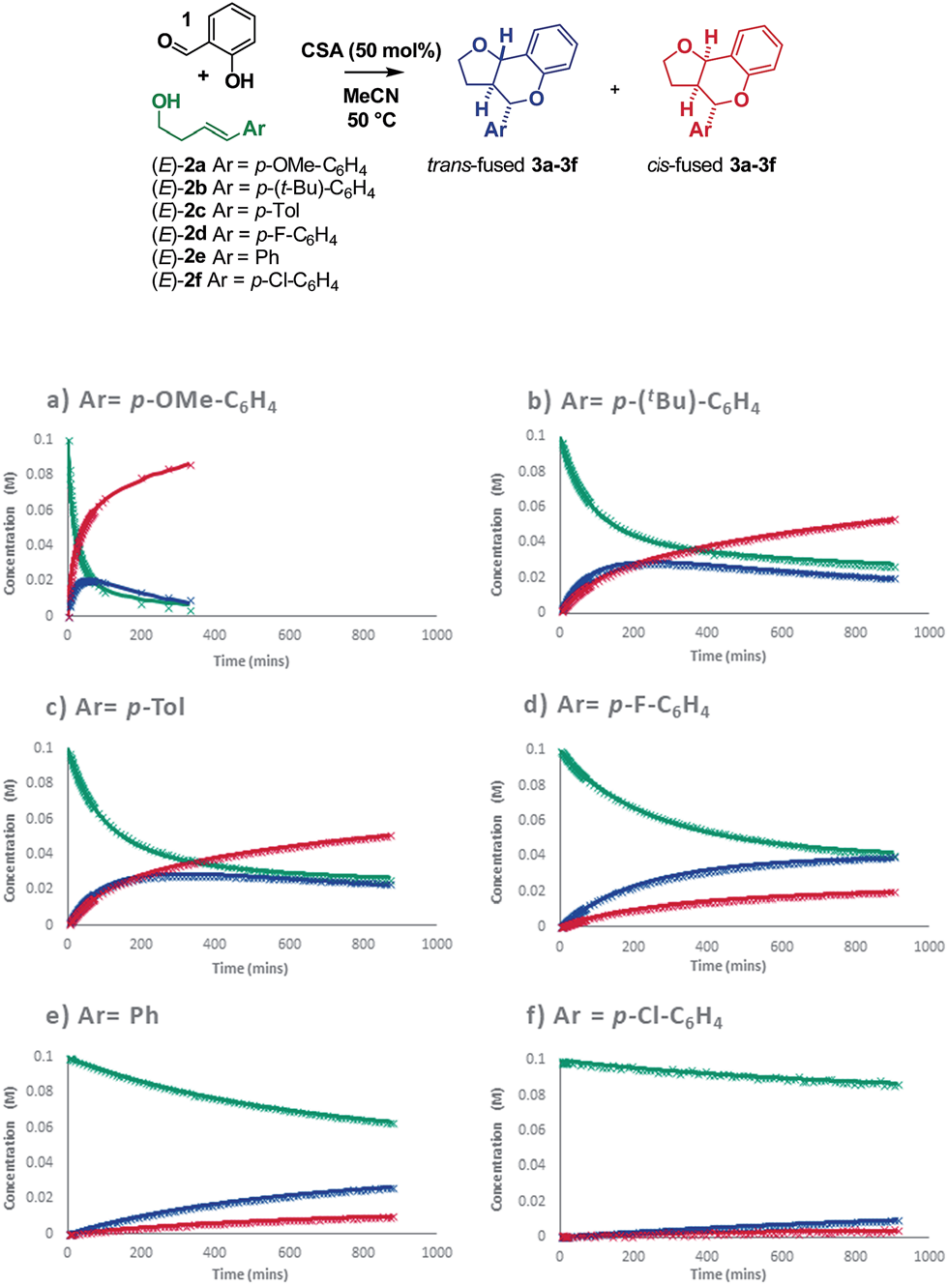
Reaction progress with varying homoallylic alcohols.

Intrigued as to how this would manifest itself in experimental data, we set out to determine a Hammett correlation for the reaction.^24^–^27^ To this end, six homoallylic alcohols with *para*-substituents of varying electronic demand from *p*-OMe to *p*-Cl (**2a–2f**) were each subjected to the reaction conditions and the reactions monitored by ^1^H NMR. While traditional Hammett analysis utilizes initial rates,^24^,^27^ we tracked the reactions for an extended period of time to allow COPASI^28^ fitting of kinetic parameters. Use of these kinetic parameters would allow us to deconvolute the complexity arising from *in situ* isomerization in our subsequent Hammett analysis and obtain separate values for each isomer (Scheme 8).

**Fig. 1:**
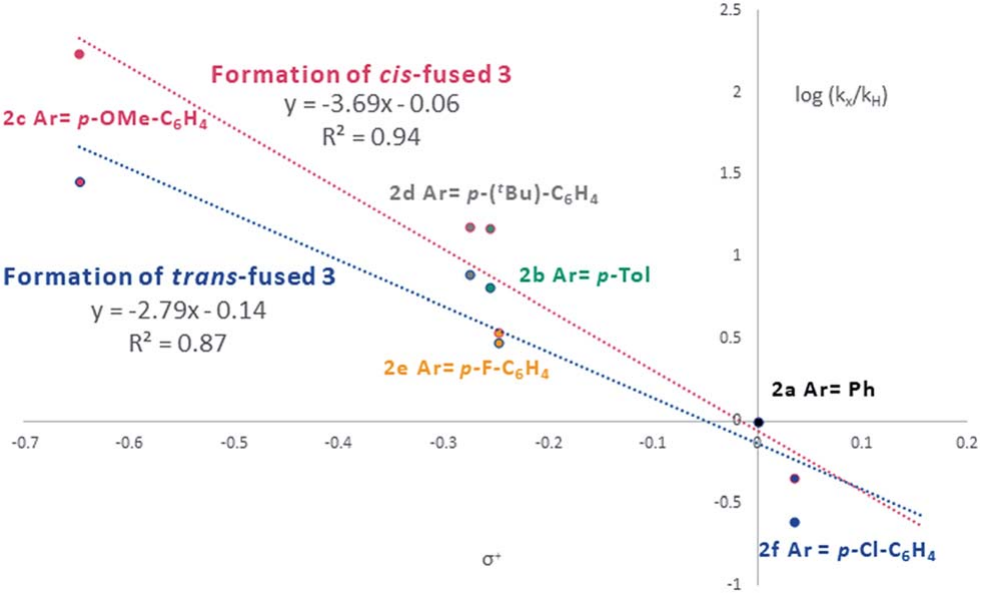
Hammett plot of COPASI fitted rate constants for formation of *cis*-fused (red) and *trans*-fused (blue) products *vs.* σ^+^.

Visual inspection of the graphs shows that the substituent has a profound effect on the rate of reaction. This was corroborated by the Hammett plots obtained from COPASI modelling of the data. The *ρ*^+^ value for formation of the *trans*-fused products was found to be less negative (*ρ*^+^ = –2.79, *R*^2^ = 0.87) than that for the formation of the *cis*-fused products (*ρ*^+^ = –3.69, *R*^2^ = 0.94), but the rate-determining TSs in both cases clearly have substantial carbocation character (Fig. 1).

To better understand the origin of this disparity and to help interpret the observed data in terms of mechanism, we turned to DFT analysis at the B3LYP+GD3BJ/Def2-TZVPP level, using HCl as the acid and a continuum solvation model. The B3LYP functional with inclusion of GD3BJ dispersion terms has recently been shown to give suitably reliable activation energies.^29^ The TS activation free energies associated with the *o*QM cycloaddition pathway were significantly lowered (∼9 kcal mol^–1^) following protonation leading us to discount the neutral *o*QM mediated pathway (*cf.*Scheme 1b). Intrinsic reaction coordinate (IRC) analysis revealed that for all six substrates, an intermediate carbocation is formed en route to the kinetic *trans*-fused products. Moreover, the Hammett plot for this HCl catalyzed Prins pathway, constructed using computed activation free energies for the stepwise transition states TS1^*endo*^ and TS2^*endo*^, correlates well with that from the CSA catalyzed experiments (*ρ*_calc_^+^ = –3.73, *R*^2^ = 0.96). IRC analysis suggests that for the *cis*-fused pathway, the substrates having electron releasing substituents (negative σ^+^ values) should proceed *via* a carbocation intermediate but that the substrates having electron withdrawing substituents (positive σ^+^ values) should follow a concerted asynchronous, acid catalyzed HDA pathway (*i.e.* one having no intermediate by IRC and hence no stationary point corresponding to TS2). As the switch of mechanism to a concerted process only applies to the *p*-Cl substituent in our experimental series (because more electron deficient substrates fail to react) the overall sensitivity to electronics as represented by the experimental *ρ*^+^, predominantly reflects that of the stepwise mechanism. Notwithstanding this, the computed Hammett *ρ*^+^ value for the *cis*-fused pathway (*ρ*_calc_^+^ = –4.34, *R*^2^ = 0.98), like the experimental one, is more negative than that for the *trans*-fused pathway.

**Fig. 2:**
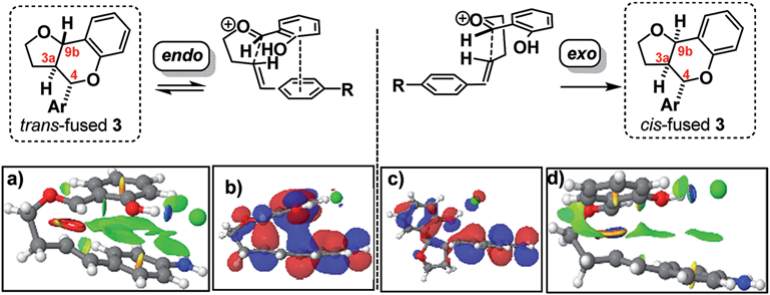
Analysis of *endo* and *exo* TS structures showing NCI surfaces (a and d)/and molecular orbitals (b and c). See FAIR data sub-collection ; https://doi.org/10.14469/hpc/4175 for 3D rotatable structures and relative transition state and product free energies.

DFT computation also supports the idea that intramolecular π–π stacking lowers the energies of the *endo*- relative to *exo*-TSs by a combination of dispersion and electrostatic attractive forces [*i.e.* noncovalent-interactions (NCIs)] attractive forces which are more significant in the former than the latter (*cf.*Fig. 2a *vs.*2d).^30^

Quantitatively, the free energies of the *endo* TSs are computed to be lower by 6–8 kcal mol^–1^*cf.* the *exo* isomers. The difference between *endo* and *exo* TS energies more normally does not exceed 4 kcal mol^–1^,^40^ currently attributed to the accumulation of attractive dispersive and electrostatic (NCI) effects. The larger stabilization of the *endo* TS in our system may also be due to contributions from genuine orbital interactions^38^ (Fig. 2b *vs.*2c) reminiscent of those found in the π-complex-like TS involved in the benzidine rearrangement.^31^,^32^

Since these interactions are strongest for electron rich substrates, they increasingly compete for the electron density released by the *para*-substituent as σ^+^ becomes more negative. This dilutes the ability of the *para* substituent to stabilize the benzylic cation, explaining the aforementioned disparity in *ρ*_calc_^+^ values for formation of *trans*- *vs. cis*-fused products.

We conclude that under Brønsted acid conditions these furanochromane forming reactions proceed to the kinetic *trans*-fused products *via* an oxonium-Prins pathway, despite commonly being classed as *o*QM cycloadditions in the literature. However, as revealed by DFT IRC analyses, there is a continuum between these mechanistic extremes with the acid catalyzed cycloaddition pathway becoming competitive for the associated thermodynamically driven isomerization to give *cis*-fused products as the homoallylic alcohol-derived alkenes become more electron deficient. So, as suggested by our initial Singleton analysis, this reaction lies at the intersection of these two mechanistic manifolds. This situation provides an opportunity to use our findings to propose a qualitative benchmark for correlation of experimentally determined *ρ*^+^ values with likely mechanism for related acid catalyzed cyclocondensation reactions. Those systems with relatively low sensitivity to the electronics of the *para* substituents are likely concerted *o*QM cycloadditions, whereas those with relatively high sensitivity are likely stepwise Prins-type reactions with the *ρ*^+^ value corresponding to this nexus being *ca.* –3. We hope that this value will prove useful for indicative mechanistic categorisation of related reactions formally involving *o*QMs (*cf.*Scheme 2), particularly where Singleton analysis is inappropriate/inconclusive and extensive computation along the lines performed here has yet to be undertaken.

**Scheme 9:**
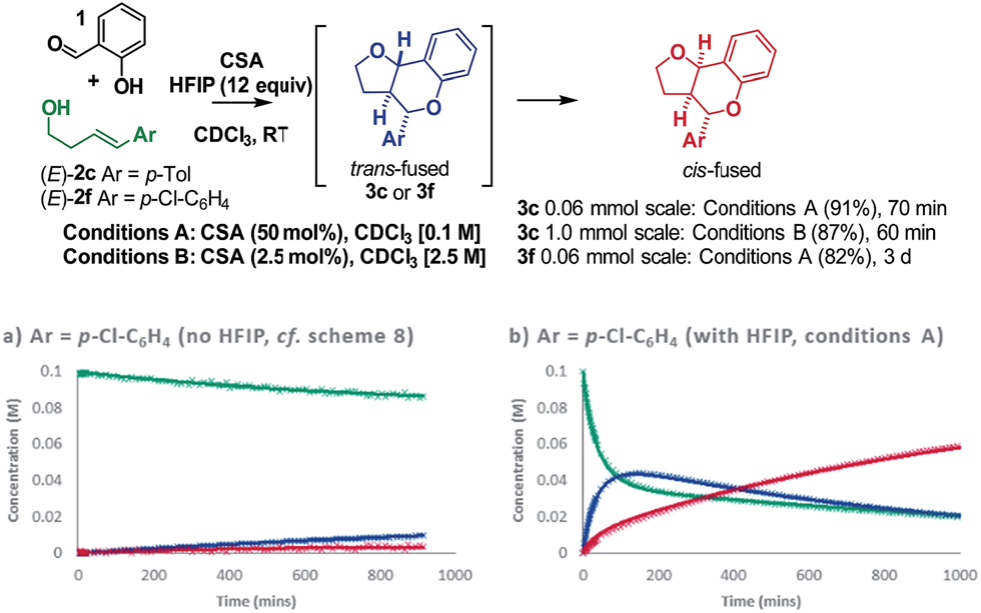
Use of HFIP as an additive.

Having resolved these mechanistic questions, we sought to utilize this insight to optimize the conditions used for the practical synthesis of furanochromanes. Carbocations are known to be stabilized by hexafluoroisopropanol (HFIP)^33^–^35^ and recent calculations by Houk^36^ have illuminated the role of H-bonding by HFIP in accelerating the rate of certain inverse-electron demand D–A reactions by favoring a zwitterionic pathway over a concerted alternative. In light of this, we investigated the effect of HFIP in these reactions. HFIP alone did not furnish product but as an additive resulted in a substantial increase in reactivity upon addition of as little as 8 equivalents.^22^ Taking advantage of the fact that HFIP also improved the solubility of the CSA, we opted to use 12 equivalents of HFIP in chloroform at RT. Using these conditions (conditions **A**), formation of *cis*-fused **3c** (Ar = *p*-Tol) from homoallylic alcohol (*E*)-**2c** was complete in just over 1 h giving an isolated yield of 91% (Scheme 9).

This result compares with a 70% conversion to a ∼2 : 1 *cis* : *trans* fused mixture of **3c** after 15 h under the previous conditions at 50 °C. Similarly, the electron poor homoallylic alcohol (*E*)-**2f**, which progressed with <20% conversion to a ∼1 : 2. *cis* : *trans*-fused mixture of **3f** after 15 h under the previous conditions, proceeded smoothly under condition **A** to furnish exclusively the *cis*-fused **3f** product in an isolated yield of 82%. Comparison of reaction profiles illustrates this HFIP induced rate increase (Scheme 9a *vs.*9b). Moreover, the COPASI fitted rate constants indicate a ∼100-fold rate enhancement. Increasing the concentration of the reaction and dropping the amount of CSA to 2.5 mol% allowed a practical preparative synthesis of **3c** in just 1 h (conditions **B**, 87%, Scheme 9).

**Scheme 10:**
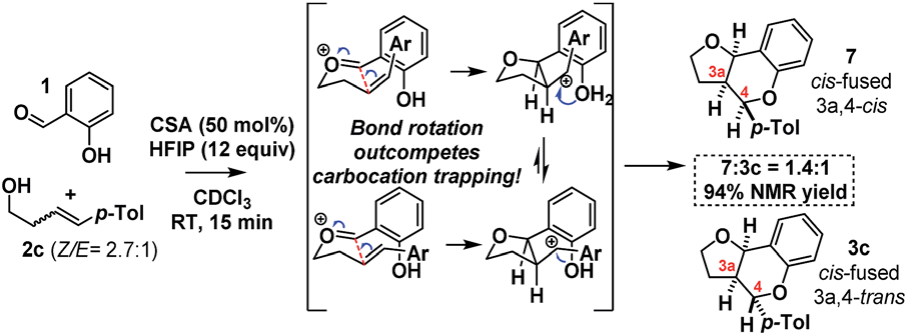
Stereochemistry scrambling with HFIP.

As HFIP is itself a Brønsted acid, we sought to confirm that the observed rate acceleration was due to its ability to stabilize the intermediate benzylic carbocation. To this end, we investigated the stereochemical fidelity with which the *Z*/*E* stereochemistry of homoallylic alcohol **2c** was translated into *cis*/*trans*-3a,4-relative stereochemistry in the product **3c**/**7** (Scheme 10).

It was found that in contrast to the complete fidelity displayed by this reaction when conducted in the absence of HFIP, a 2.7 : 1 mixture of (*Z*/*E*)-**2c** led to partially scrambled C_3a_–C_4_-stereochemistry in the product. This suggests that the acceleration is due to the stabilization of the intermediate benzylic carbocation. Recent calculations suggest carbocation lifetimes must exceed 1000 fs for loss of stereochemical information.^37^ In a control reaction, under the same conditions but in the absence of the salicylaldehyde (**1**), **2c** was found to cyclize to cleanly furnish 2-tolyl-tetrahydrofuran. This reaction presumably occurs *via* protonation of the alkene, carbocation formation and then intramolecular cyclization.^38^

**Scheme 11:**
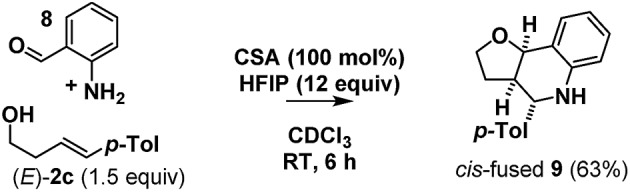
Tetrahydroquinoline formation with HFIP.

Looking to test the limits of the new protocol, we looked to deploy 2-aminobenzaldehyde (**8**) in place of salicylaldehyde (**1**) as a substrate. While this aniline has previously only been employed in Friedländer quinoline synthesis,^39^,^40^ and despite concerns regarding its polymerization and its basicity relative to phenol, we were delighted to find that the desired furanotetrahydroquinoline **9** could be isolated using the chloroform/HFIP conditions albeit promoted by a stoichiometric amount of CSA (Scheme 11).

**Scheme 12:**
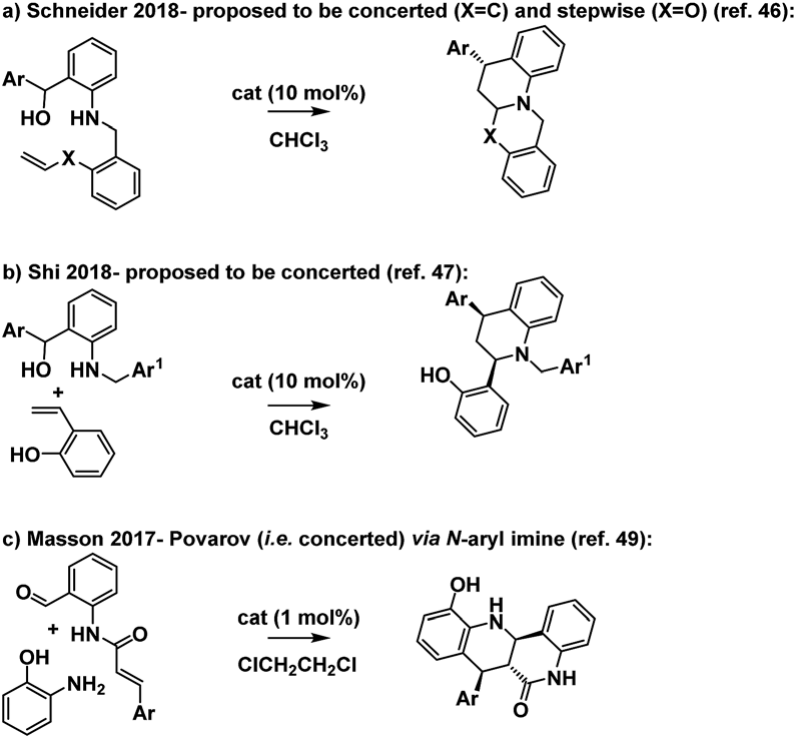
Tetrahydroquinoline forming reactions.

Based on the foregoing studies we expect that this reaction proceeds *via* a stepwise Prins pathway involving the relatively rarely encountered trapping of a benzylic carbocation by an aniline nitrogen.^41^,^42^ Related reactions have however recently been suggested to proceed *via o*QM imine formation then cycloaddition (Scheme 12a and b).^42^–^48^

The reaction is notable in providing access to a substituted tetrahydroquinoline *via* a disconnection which is complimentary to the more established hetero-D–A reaction of an *N*-aryl imine with an electron rich alkene (the Povarov reaction, *e.g.*Scheme 12c).^49^–^51^ The Povarov reaction has also been proposed to proceed *via* both stepwise and concerted pathways.^52^

## Conclusion

In summary, we have conducted an experimental and computational investigation into the mechanism of formation of furanochromanes under Brønsted acid catalysis. Kinetic *trans*-fused products are all shown to be formed *via* a stepwise oxonium-Prins reaction. A new isomerization to furnish the thermodynamic and biologically active *cis*-fused congeners also follows this pathway for all but the most electron deficient substrates, which react slowly *via* an asynchronous acid catalyzed *o*QM cycloaddition pathway.^25^ Rational modification of the conditions by addition of HFIP achieved a *ca.* 100-fold rate enhancement to give *cis*-fused configured furanochromane products exclusively and allowed access to a furanotetrahydroquinoline by using 2-aminobenzaldehyde in place of salicylaldehyde.

Beyond the mechanistic insight and practical scope extension the work has provided for this furanochromane-forming reaction, the study has illuminated a correlation between the Hammett *ρ*^+^ value and mechanism for this class of acid catalyzed cyclocondensation. High sensitivity to electronic factors (*i.e. ρ*^+^ value more negative than *ca.* –3) militates in favour of a concerted *o*QM cycloaddition pathway, particularly for electron withdrawing substituents, whereas low sensitivity (*i.e. ρ*^+^ value less negative than *ca.* –3) is indicative of a stepwise Prins-type pathway. Moreover, this sensitivity and hence mechanism of formation can differ between stereoisomeric products because the electronic communication between the Hammett *para*-substituent and the benzylic position is ‘intercepted’ by intramolecular π–π interactions in *endo*- but not *exo*-TSs – a phenomenon referred to as ‘secondary orbital overlap’ in D–A parlance.

## Conflicts of interest

There are no conflicts to declare.

## Supplementary Material

Supplementary information
