# Supplementary material for: Reversibility and reactivity in an acid catalyzed cyclocondensation to give furanochromanes – a reaction at the ‘oxonium-Prins’ vs. ‘ortho-quinone methide cycloaddition’ mechanistic nexus
Source: Chem Sci. 2018 Oct 19;10(2):406–12. doi: 10.1039/c8sc04302g (PMC6334628; doi:10.1039/c8sc04302g)
Supplement: Supplementary file 1 [file SC-010-C8SC04302G-s001.pdf]

Electronic Supplementary Material (ESI) for Chemical Science.
This journal is © The Royal Society of Chemistry 2018

Reversibility and reactivity in an acid catalyzed cyclocondensation to give furanochromanes – A reaction at the 'oxonium-Prins' *vs.* '*ortho*-quinone methide cycloaddition' mechanistic nexus

Christian D.-T. Nielsen, Wouter J. Mooij, David Sale, Henry S. Rzepa, Jordi Burés and Alan C. Spivey*

Supporting Information

Contents

1. General directions 2
2. Procedures for the synthesis of homoallylic alcohols 2
2.1 General Procedure 2
3. Isomerisation study 5
3.1 Orbital analysis for isomerisation 5
3.2 Racemisation Study 5
3.3 NMR tracking of isomerisation 8
4. Representative processing of raw spectral data 9
5. Processing data using COPASI 11
6. Procedure for NMR tracking of reactions 12
7. Initial rate Hammett plot 13
8. COPASI modelling for Hammett plot 17
9. DFT modelling 20
10. Determination of the optimal quantity of HFIP 25
11. NMR tracking of reactions using HFIP as an additive 26
12. Homoallylic alcohol alkene scrambling experiments 28
13. Oxonium-Prins products 32
14. References 34
15. NMR spectra for new compounds 35
16. Appendix 40

1. General directions

All reactions were performed under nitrogen using oven-dried glassware unless stated otherwise. Yields refer to chromatographically and spectroscopically (1H-NMR) homogenous materials, unless otherwise indicated. MeCN, CH_2Cl_2, THF, Et_2O, DMF, and toluene were dried and deoxygenated with a Grubbs PureSolv 400 solvent purification system. The moisture content of the solvents was monitored by Karl Fischer coulometric titration (Mettler-Toledo DL39). **Reagents**: used as purchased from commercial sources, unless otherwise stated, and used according to COSHH regulations. **Chromatography**: Flash chromatography (FC) was performed on silica gel (Merck Kieselgel 60 F254 230-400 mesh) unless otherwise stated. **Melting Points**: determined on a Stanford Research System OptiMelt. Thin Layer Chromatography (TLC): performed on Merck aluminium-backed plates pre-coated with silica (0.2 mm, 60 F254) which were visualized either by quenching of ultraviolet fluorescence (λmax = 254 and 366 nm) or staining with; potassium permanganate/Δ, bromocresol green/Δ or phosphomolybdic acid/Δ TLC dips prepared according to general procedures. 1H NMR spectra: recorded on a 400 or 500 MHz Bruker AMX-400/500 instrument. Chemical shifts (δH) are quoted in parts per million (ppm), referenced to the appropriate residual solvent peak. 13C NMR spectra: recorded at 101 MHz or 125 MHz on a Bruker AMX-400/500 instrument Chemical shifts (δC) are quoted in parts per million (ppm), referenced to the appropriate residual solvent peak. High Resolution Mass Spectra: recorded on either a VG platform II or VG AutoSpec spectrometer, with only molecular ions ([MH]+, [MNa]+, [MNH4]+, [MH2O]+, [MH]-) and major peaks being reported. **Analytical HPLC analyses:** carried out on an Agilent 1260 Infinity Series system, employing Daicel Chiracel columns. **Preparative HPLC**: carried out on an Agilent 1200 series, employing Daicel Chiralpak columns.

2. Procedures for the synthesis of homoallylic alcohols[1]

2.1 General Procedure

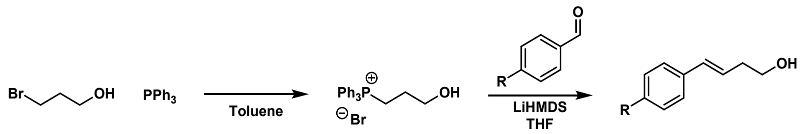

Under Ar, triphenylphosphine (2.62 g, 10 mmols, 1 equiv) was dissolved in dry toluene (9 mL). To this was added 3-bromopropan-1-ol (0.9 mL, 10 mmol, 1 equiv) and refluxed for 4 h. After this time, the reaction was allowed to cool, solids filtered, washed with cold diethyl ether and dried under vacuum to afford 3-(triphenylphsophonium)propan-1-ol-bromide as a white powder (4.0 g, 80%) which was used without purification in the next step.

Lithium bis(trimethylsilyl)amide (1 M in THF, 2.8 mL, 3.5 equiv) was added dropwise to 3-(triphenylphsophonium)propan-1-ol-bromide (400 mg, 1 mmol, 1.25 equiv) in THF (5 mL) under N_2 at −20 °C. This was allowed to stir for 30 min before addition of the desired aldehyde (0.8 mmol, 1 equiv). This solution was then stirred overnight and allowed to warm to room temperature. The reaction was quenched by addition of aqueous NH_4Cl. 1 M HCl was added to take the solution to pH 1, extracted with CH_2Cl_2, dried over Na_2SO_4 and concentrated under reduced pressure. The resultant oil was purified by flash column chromatography (10% EtOAc/CH_2Cl_2) to yield product.

(*E*)-4-(4-Methoxyphenyl)but-3-en-1-ol[2] (**2a**)

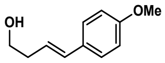

Following the general procedure, isolated as an off white solid (**2a**, 108 mg, 76%)

^{1}H NMR (400 MHz, Chloroform-*d*) δ 7.30 (d, *J* = 8.7 Hz, 2H), 6.88 – 6.81 (m, 2H), 6.45 (d, *J* = 15.8 Hz, 1H), 6.06 (dt, *J* = 15.9, 7.2 Hz, 1H), 3.80 (d, *J* = 1.1 Hz, 3H), 3.74 (q, *J* = 6.1 Hz, 2H), 2.47 (td, *J* = 7.6, 7.0, 5.6 Hz, 2H), 1.42 (t, *J* = 5.8 Hz, 1H). In agreement with literature.[2]

(*E*)-4-(4-(*tert*-Butyl)phenyl)but-3-en-1-ol (**2b**)

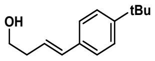

Following the general procedure, isolated as a clear oil (**2b**, 90 mg, 55%).

^{1}H NMR (400 MHz, Chloroform-*d*) δ 6.49 (dt, *J* = 15.9, 1.4 Hz, 1H), 6.16 (dt, *J* = 15.9, 7.2 Hz, 1H), 3.75 (t, *J* = 6.3 Hz, 2H), 2.48 (dtd, *J* = 7.6, 6.3, 1.4 Hz, 2H), 1.47 (s, 1H), 1.32 (s, 9H). ^{13}C NMR (101 MHz, Chloroform-*d*) δ 150.5, 134.6, 132.8, 125.9, 125.6, 62.2, 36.6, 34.7, 31.4. HRMS (EI) $C_{12}H_{20}O$ requires 204.1514, found: 204.1511 (**Δ**= -1.55 ppm).

(*E*)-4-(*p*-Tolyl)but-3-en-1-ol[2] [(*E*)-**2c**]

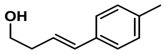

Following the general procedure, isolated as an off white solid [(*E*)-**2c**, 98 mg, 76%).

^{1}H NMR (400 MHz, Chloroform-*d*) δ 7.26 (d, *J* = 8.1 Hz, 2H), 7.11 (d, *J* = 7.8 Hz, 2H), 6.47 (dt, *J* = 15.8, 1.4 Hz, 1H), 6.15 (dt, *J* = 15.8, 7.2 Hz, 1H), 3.75 (q, *J* = 6.1 Hz, 2H), 2.48 (dtd, *J* = 7.6, 6.3, 1.4 Hz, 2H), 1.48 – 1.40 (m, 1H). Data in agreement with literature.[2]

(*Z*)-4-(*p*-Tolyl)but-3-en-1-ol[3] [(*Z*)-**2c**]

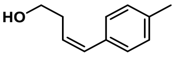

Commentary on synthesis: To synthesize the *Z* homoallylic we opted to conduct an unselective Wittig reaction. Surprisingly, no reaction was observed when employing the unprotected phosphonium bromide under typical unselective Wittig conditions (NaH, KO^{t}Bu, NaHMDS). As such we hypothesised that the prior Wittig was in part successful due to the lithium coordination with the free alcohol. We therefore protected the alcohol which allowed us to access a mixture of *Z/E* alkenes. While the protection was facile, formation of the phosphonium was significantly slower (24 h, reflux, approx. 70% conversion by ^{31}P NMR). Further to this, the phosphonium was now a dense oil, unsuitable for purification by filtration. Instead, the toluene was decanted off before addition of THF and addition of NaHMDS in THF (1 M). This reaction was quenched by addition of NH_4Cl (sat. soln.) and the organic extracted with diethyl ether. The organic phase was dried over $MgSO_4$ and concentrated. This was then subjected to TBAF in THF (1M) which in turn was concentrated and purified over silica gel eluting 10% EtOAc/$CH_2Cl_2$to yield a 2.7:1 mixture of *Z* and *E* isomers:

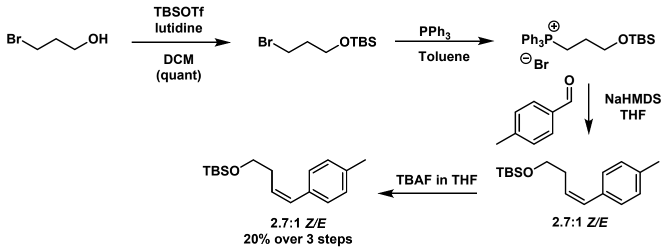

3-Bromopropan-1-ol (0.2 mL, 2.1 mmol, 1 equiv) was allowed to stir in dry CH_2Cl_2 (5 ml) under N_2. To this was added lutidine (0.78 ml, 6.3 mmols, 3 equiv) and TBSOTf (1 mL, 4.2 mmols, 2 equiv) at 0 °C. This was allowed to warm to room temperature and continued to be stirred for 12 h. After this time, the reaction was quenched by addition of NH_4Cl (sat soln). The organic layer was extracted with ethyl acetate and concentrated before being purified over silica gel eluting 1% diethyl ether/hexane to yield 3-(*tert*-Butyldimethylsilyloxy)propyl bromide[4] as a clear oil (535 mg, quant). ^{1}H NMR (400 MHz, Chloroform-*d*) δ 3.73 (t, *J* = 5.7 Hz, 2H), 3.52 (t, *J* = 6.4 Hz, 2H), 2.03 (tt, *J* = 6.4, 5.7 Hz, 2H), 0.90 (s, 9H), 0.07 (s, 6H). In agreement with literature.[4]

Then, under Ar, triphenylphosphine (524 mg, 2 mmol, 2 equiv) was dissolved in dry toluene (1.8 mL). To this was added butyldimethylsilyloxy)propyl bromide (504 mg, 2 mmol, 2 equiv) and refluxed for 24 h. At this point the mixture was cooled and phases allowed to separate. The top layer of toluene was decanted off before addition of THF (2mL). The vessel was cooled to – 20 °C and NaHMDS (1M in THF, 3 mL, 3 equiv) was added dropwise. This was allowed to stir for 20 minutes before addition of *p*-methyl benzaldehyde (0.12 mL, 1 mmol, 1 equiv). This was allowed to warm to room temperature and stirred overnight. At this point the reaction was quenched by addition of NH_4Cl (sat soln). The organic layer was extracted with diethyl ether and dried over $MgSO_4$. This yielded 100 mg of crude protected homoallylic alcohol which was carried directly into TBAF deprotection.

Protected homoallylic alcohol (80 mg, 0.28 mmol, 1 equiv) was stirred in TBAF (1 M in THF, 1.5 ml, 5 equiv) at rt, under N_2 for 20 min. The reaction was simply concentrated under a stream of N_2 and purified over silica gel 10% EtOAc/$CH_2Cl_2$to yield a mixture of 2.7:1 *Z*/*E* homoallylic alcohols **2c** (32 mg, 20% over 3 steps).

E isomer, data as above.

Z isomer: ^{1}H NMR (400 MHz, Chloroform-*d*) δ 7.21 (d, *J* = 8.2 Hz, 2H), 7.14 (d, *J* = 8.0 Hz, 2H), 6.56 (d, *J* = 11.6, 1.9 Hz, 1H), 5.64 (dt, *J* = 11.6, 7.3 Hz, 1H), 3.75 (t, *J* = 6.4 Hz, 2H), 2.62 (dtd, *J* = 7.3, 6.4, 1.9 Hz, 2H), 2.35 (s, 3H), 1.47 (s, 1H). In agreement with literature.[3]

(*E*)-4-(4-Fluorophenyl)but-3-en-1-ol[2] (**2d**)

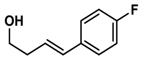

Following the general procedure, isolated as a colourless oil (**2d**, 45 mg, 27%).

^{1}H NMR (400 MHz, Chloroform-*d*) δ 7.42 – 7.28 (m, 2H), 7.08 – 6.92 (m, 2H), 6.46 (dt, *J* = 15.9, 1.5 Hz, 1H), 6.12 (dt, *J* = 15.8, 7.1 Hz, 1H), 3.76 (q, *J* = 6.1 Hz, 2H), 2.56 – 2.41 (m, 2H), 1.43 (t, *J* = 5.7 Hz, 1H). In agreement with literature.[2]

(*E*)-4-Phenylbut-3-en-1-ol[2] (**2e**)

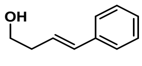

Following the general procedure, isolated as a clear oil (**2e**, 75 mg, 63%).

^{1}H NMR (400 MHz, Chloroform-*d*) δ 7.42 – 7.34 (m, 2H), 7.34 – 7.28 (m, 2H), 7.25 – 7.20 (m, 1H), 6.51 (dt, *J* = 16.0, 1.5 Hz, 1H), 6.21 (dt, *J* = 15.9, 7.1 Hz, 1H), 3.76 (t, *J* = 6.3 Hz, 2H), 2.49 (dtd, *J* = 7.7, 6.4, 1.4 Hz, 2H), 1.69 (s, 1H). Data in agreement with literature.[2]

(*E*)-4-(4-Chlorophenyl)but-3-en-1-ol (2f)

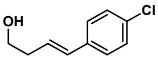

Following the general procedure, isolated as a colourless oil (**2f**, 109 mg, 75%).

^{1}H NMR (400 MHz, Chloroform-*d*) δ 7.30 – 7.24 (m, 4H), 6.45 (dt, *J* = 15.9, 1.4 Hz, 1H), 6.19 (dt, *J* = 15.9, 7.1 Hz, 1H), 3.76 (t, *J* = 6.3 Hz, 2H), 2.48 (dtd, *J* = 7.7, 6.4, 1.5 Hz, 2H), 1.56 (s, 1H). In agreement with literature.[2]

3. Isomerisation study

3.1 Orbital analysis for isomerisation

Four possible pathways were envisioned for isomerisation from the kinetic *trans*-fused products to the thermodynamic *cis*-fused ones: two oxygens can be protonated and there are two 'directions' in which the electrons can flow, either around the *sp³* C-C framework or into the aryl ring. The orbital overlap for the initial interaction of the appropriate oxygen lone pair with the appropriate C-C anti-bonding orbital is good for the *retro* Prins/HDA, oxy Michael and Grob pathways but not for the benzyne pathway:

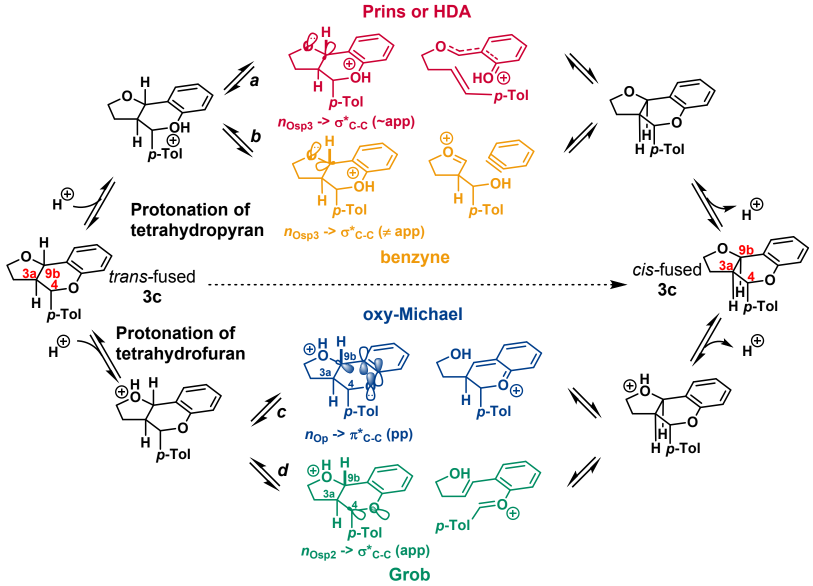

Consequently, we hypothesized that the *retro* Prins/HDA, oxy Michael and Grob pathways were more likely to be operative.

3.2 Racemisation Study

Analysis for this experiment was carried out by analytical chiral stationary phase HPLC using a Diacel IF column (10 mm x 20 mmL) eluting 5% IPA/Hexane at 1 mL/min and visualised at 210 nm.

Both enantiomers of *cis*-fused-**3c** and of *trans*-fused-**3c** were partially resolved under these conditions.

A sample of *cis*-fused-**3c** (*dr* ~1:3) was obtained from the following reaction:

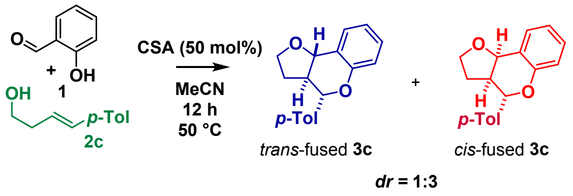

HPLC chromatogram of (±)-*cis*-fused-**3c** (*dr* ~1:3) (Figure S1)

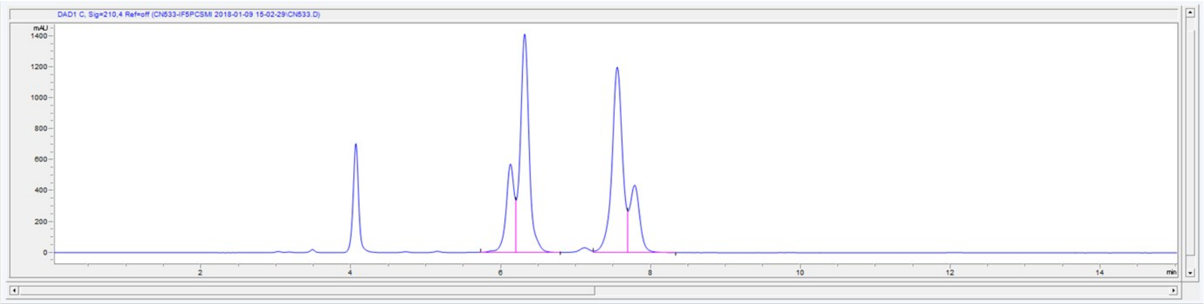

File Information

LC-File	CN533.D
File Path	D:\HPLC\DATA\CN533-IF5PCSMI 2018-01-09 15-02-29
Date	09-Jan-18, 15:26:09
Sample	cn533
Sample Info	
Barcode	
Operator	SYSTEM
Method	IF_5%_30.M
Reference	
Analysis Time	29.993 min
Sampling Rate	0.0067 min (0.402 sec), 4500 datapoints

#	Time	Area	Height	Width	Area%	Symmetry
1	6.128	4429.1	572	0.1123	13.979	1.231
2	6.318	11543.2	1410.9	0.1234	36.433	0.911
3	7.553	11728.6	1197.1	0.1463	37.018	1.021
4	7.784	3982.7	435.9	0.1366	12.570	0.825

A sample of *trans*-fused-**3c** (*dr* ~5:1) was obtained from the following reaction (*t* = 5 h):

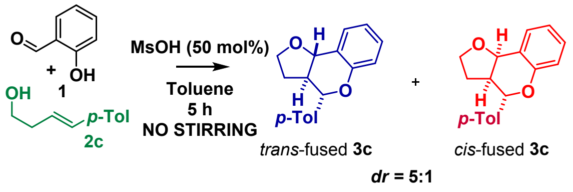

The diastereomeric purity was then increased (to dr >20:1) by preparative TLC (20% diethyl ether in petroleum ether).

HPLC chromatogram of (±)-*trans*-fused-**3c** (*dr* >20:1) (Figure S2)

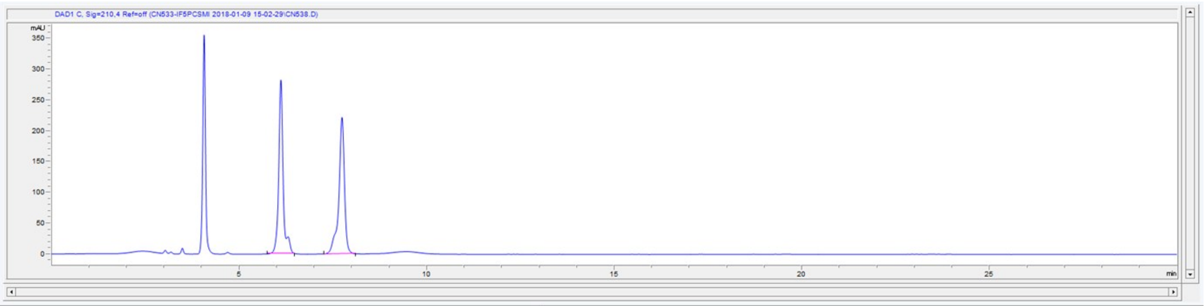

File Information

LC-File	CN538.D
File Path	D:\HPLC\DATA\CN533-IF5PCSMI 2018-01-09 15-02-29
Date	09-Jan-18, 15:57:38
Sample	cn538
Sample Info	
Barcode	
Operator	SYSTEM
Method	IF_5%_30.M
Reference	
Analysis Time	29.993 min
Sampling Rate	0.0067 min (0.402 sec), 4500 datapoints

#	Time	Area	Height	Width	Area%	Symmetry
1	6.115	2345.3	282.4	0.1384	50.098	0.914
2	7.744	2336.2	221.7	0.1756	49.902	1.286

Preparative HPLC was then used to separate the *trans*-fused-**3c** sample into its enantiomers using a Diacel Chiralpak IF column (20 mm diameter x 250 mm), eluting with 5% IPA/Hexane at 18 mL/min with 2.3 mg injected. The slower eluting enantiomer was collected:

HPLC chromatogram of single enantiomer of *trans*-fused-**3c** product (Figure S3)

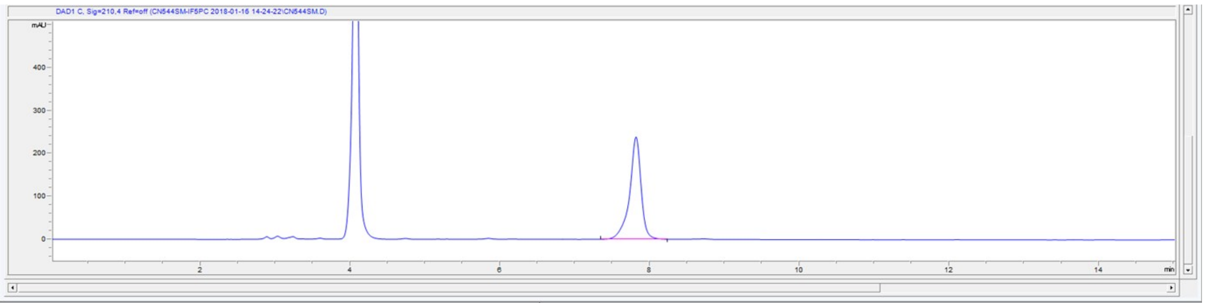

File Information

LC-File	CN544SM.D
File Path	D:\HPLC\DATA\CN544SM-IF5PC 2018-01-16 14-24-22
Date	16-Jan-18, 14:49:22
Sample	cn544SM
Sample Info	
Barcode	
Operator	SYSTEM
Method	IF_5%_20.M
Reference	
Analysis Time	19.993 min
Sampling Rate	0.0067 min (0.402 sec), 3000 datapoints

#	Time	Area	Height	Width	Area%	Symmetry
1	7.817	2542.5	239.1	0.156	100.000	1.258

This single *trans*-fused-**3c** enantiomer was subjected to the reaction conditions for the cyclocondensation reacton: CSA (1.15 mg, 0.005 mmol) in MeCN (0.1 mL) at 50 °C for 12 h. After this time, the solution was eluted through a silica plug and flushed with ethyl acetate to remove CSA before HPLC analysis.

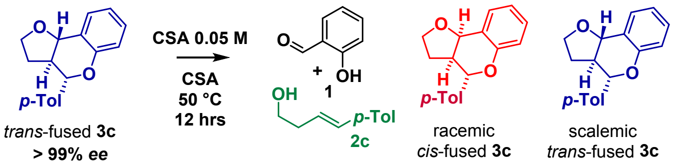

HPLC chromatogram of reaction mixture after 12 h (Figure S4)

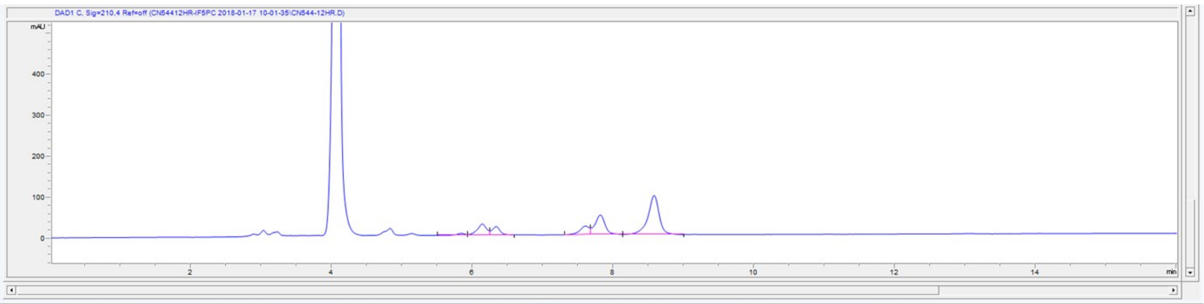

File Information

LC-File	CN544-12HR.D
File Path	D:\HPLC\DATA\CN54412HR-IF5PC 2018-01-17 10-01-35
Date	17-Jan-18, 10:25:23
Sample	cn544-12hr
Sample Info	
Barcode	
Operator	SYSTEM
Method	IF_5%_20.M
Reference	
Analysis Time	20 min
Sampling Rate	0.0067 min (0.402 sec), 3001 datapoints

#	Time	Area	Height	Width	Area%	Symmetry
1	5.85	45.1	4.8	0.1359	2.025	1.72
2	6.147	257.9	27.4	0.1337	11.578	1.236
3	6.342	164	20.8	0.118	7.362	0.919
4	7.611	209.1	21.3	0.1425	9.388	1.503
5	7.823	483.7	48.3	0.1489	21.718	1.014
6	8.587	1067.5	95.6	0.1643	47.929	1.236

To confirm the identity of the two new peaks at 5.85 min and 8.59 min, first homoallylic alcohol **2c** was added to the vial.

HPLC chromatogram of reaction after 12 h spiked with starting homoallylic alcohol **2c** (Figure S5)

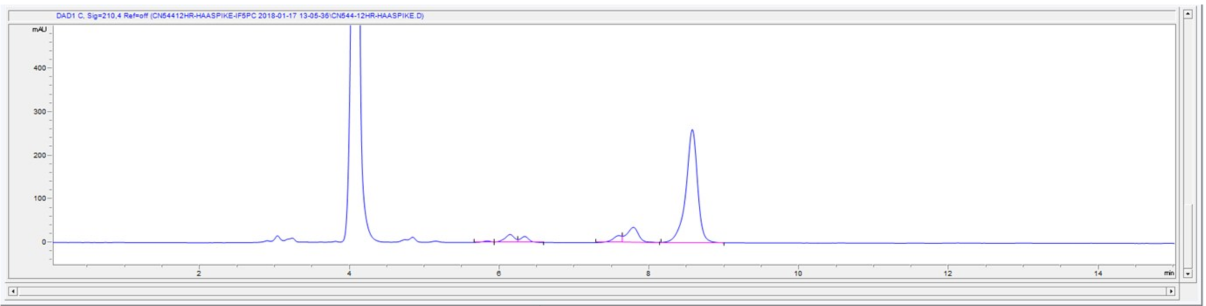

File Information

LC-File	CN544-12HR-HAASPIKE.D
File Path	D:\HPLC\DATA\CN54412HR-HAASPIKE-IF5PC 2018-01-17 13-05-36
Date	17-Jan-18, 13:29:22
Sample	cn544-12hr-HAAspike
Sample Info	
Barcode	
Operator	SYSTEM
Method	IF_5%_20.M
Reference	
Analysis Time	20 min
Sampling Rate	0.0067 min (0.402 sec), 3001 datapoints

#	Time	Area	Height	Width	Area%	Symmetry
1	5.84	26.9	3.3	0.1201	0.733	1.171
2	6.144	172.4	18.5	0.1347	4.705	1.238
3	6.339	110	14	0.1176	3.000	0.927
4	7.595	140.8	16.2	0.1277	3.840	2.1
5	7.79	386.4	35.2	0.162	10.542	1.116
6	8.578	2828.6	259.9	0.161	77.179	1.213

The peak at 8.59 min increased indicating its identity as homoallylic alcohol **2c**.

To confirm the identity of the salicylaldehyde (**1**), an authentic sample of this was added to the vial.

HPLC of reaction after 12 h spiked with homoallylic alcohol **2c** and salicylaldehyde (**1**) (Figure S6)

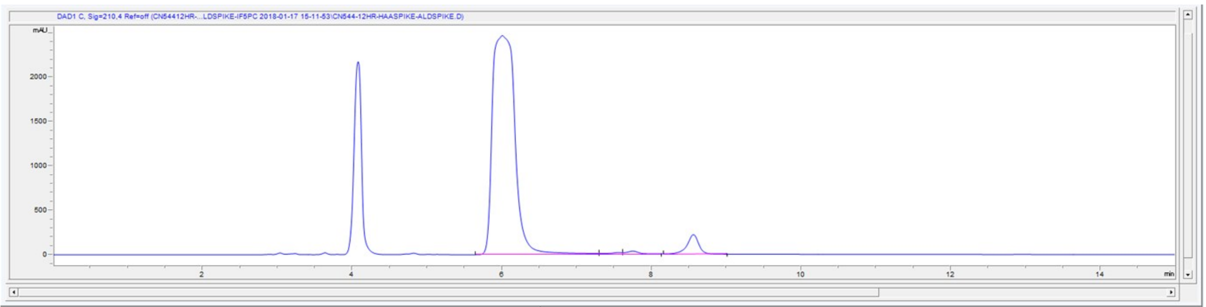

File Information

LC-File	CN544-12HR-HAASPIKE-ALDSPIKE.D
File Path	D:\HPLC\DATA\CN54412HR-HAASPIKE-ALDSPIKE-IF5PC 2018-01-1
Date	17-Jan-18, 15:35:39
Sample	cn544-12hr-HAAspike-Aldspike
Sample Info	
Barcode	
Operator	SYSTEM
Method	IF_5%_20.M
Reference	
Analysis Time	20 min
Sampling Rate	0.0067 min (0.402 sec), 3001 datapoints

#	Time	Area	Height	Width	Area%	Symmetry
1	6.008	51477.7	2464.8	0.2557	94.097	0.75
2	7.557	272.9	20.5	0.1834	0.499	3.19
3	7.752	489.2	36.8	0.1892	0.894	0.745
4	8.561	2467	221.5	0.1639	4.509	1.225

The peak at 5.85 min increased (to saturation) indicating its identity as salicylaldehyde (**1**).

3.3 NMR tracking of isomerisation

The isomerization of *trans*-fused **3c to** *cis*-fused **3c** was monitored by ^{1}H NMR.

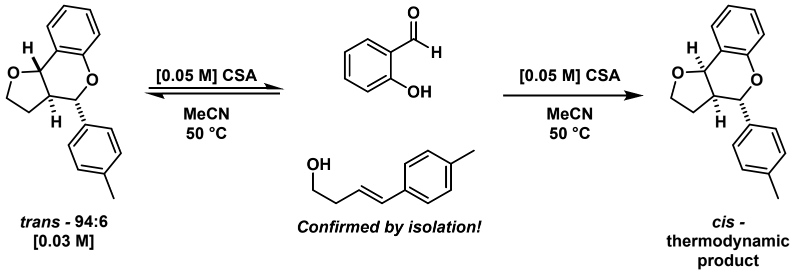

Thus, *trans*-fused-**3c** (*dr* 94:6, 3.3 mg, 0.01 mmol) was weighed into a vial. To this was added CSA (4.6 mg, 0.02 mmol) in d3-MeCN (0.4 mL) which had been pre heated to 50 °C. This combined mixture was transferred to an NMR tube and placed in a pre-heated NMR spectrometer at 50 °C.

From this, integration of spectra could reveal how distribution of species changed with respect to time. The reaction was observed to be clean with retention of mass balance and so allowed plotting of concentration as a function of time using the initial concentration (Scheme 5, main manuscript)

4. Representative processing of raw spectral data

NMR data was processed using MestReNova. Spectra were automatically phase corrected and baselined (either Whittaker smoother or Ablative). Spectra were stacked and superimposed with characteristic peaks integrated. Raw integration values were then processed in Excel. All integrals were summed together, and a concentration of species was obtained. Below is processing procedure for the isomerisation spectra.

Superimposed ^{1}H NMR spectra for isomerisation (Figure S7)

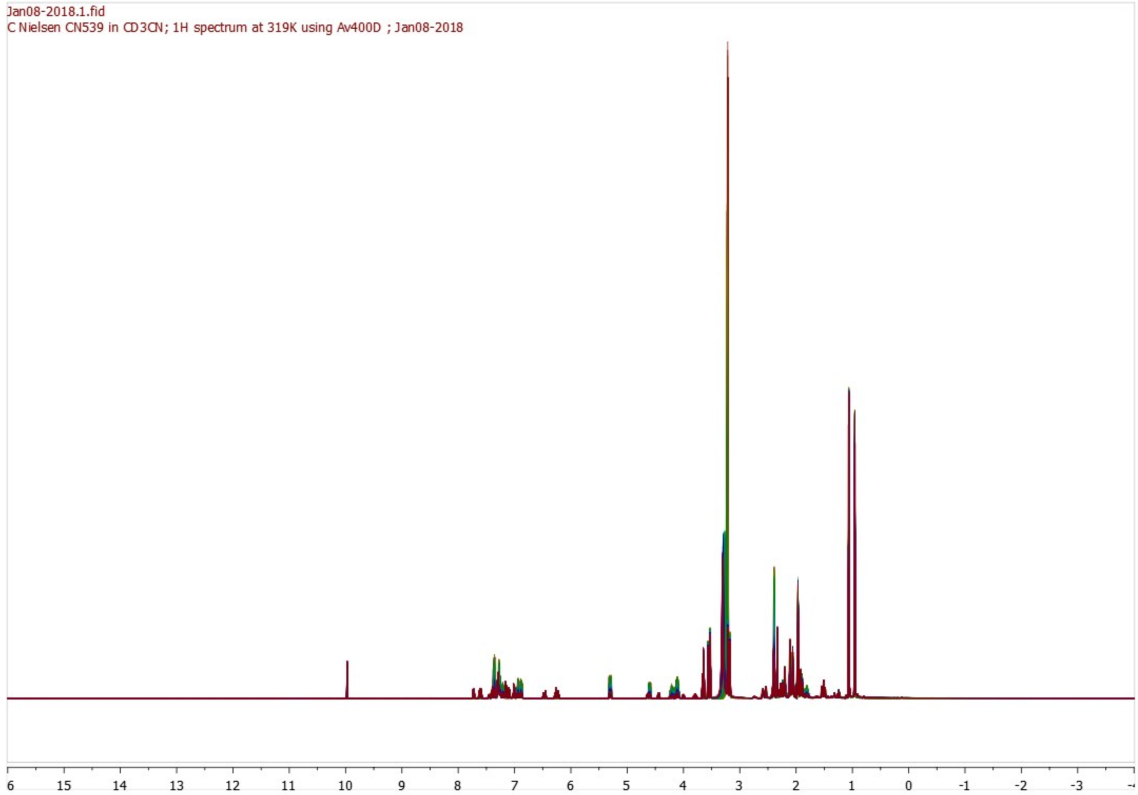

Characteristic ^{1}H NMR peaks of compounds **2c** and **3c** (Figure S8)

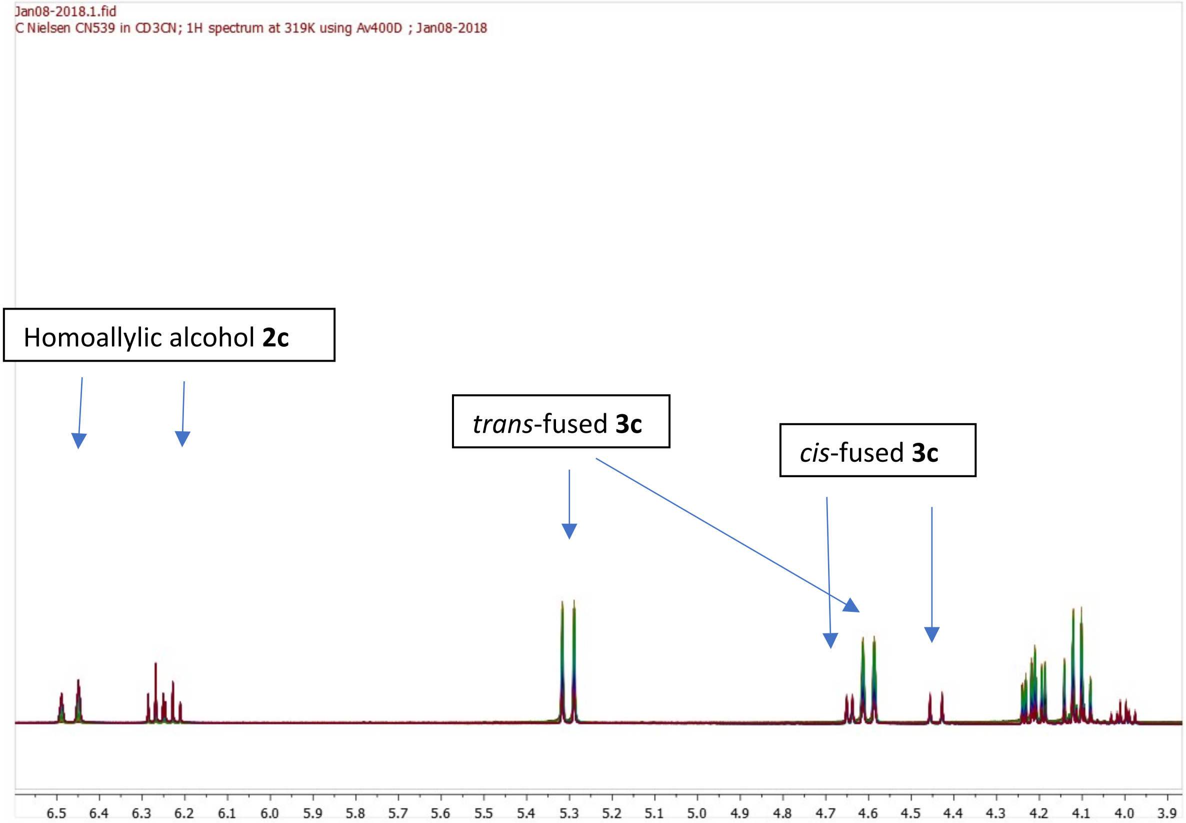

The *cis*-fused and *trans*-fused diastereoisomers were assigned by reference to the isolated single diastereomers of **3c** (chemical shift and coupling constant) and by Mass spectrometry.

For tabulated data see **Appendix 1.**

Stacked ^{1}H NMR spectra for isomerization of *trans*-fused-**3c** → *cis*-fused-**3c** (Figure S9).

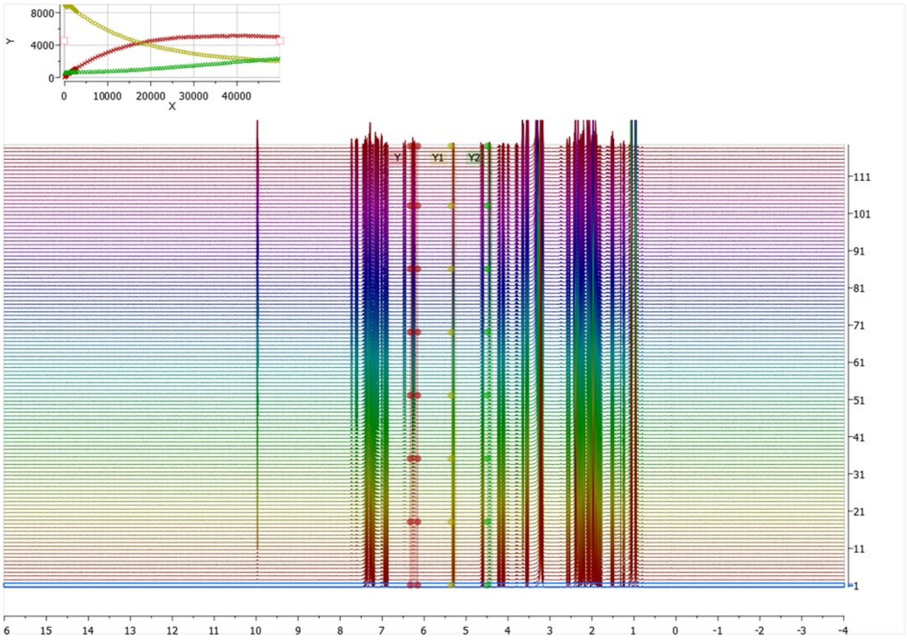

Graph of isomerization of *trans*-fused-**3c** ⟶ *cis*-fused-**3c** (Figure S10)

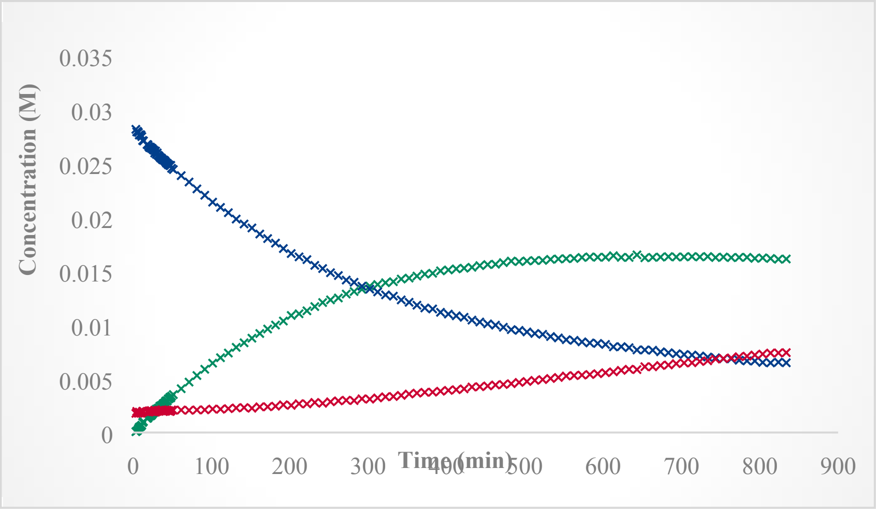

5. Processing data using COPASI

Using COPASI, the experimental data collected for the full reaction courses were taken and modelled. Inputting the reaction progress from ^{1}H NMR and using the scatter search method with 200 iterations led to curves with good levels of fitting. The lower bound for all reactions was set to zero with an upper bound of infinity. Alternative methods were trialled sequentially to ensure correct best fit identification. As a representation, the isomerization data for the transformation of *trans*-fused-**3c** ⟶ *cis*-fused-**3c** is shown below.

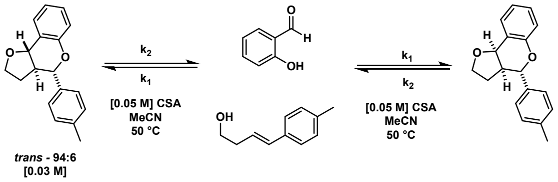

The process was modelled as being reversible in both the formation of the *trans*-fused- and *cis*-fused diastereomers. The fitted parameters showed good agreement with the experimental data.

For tabulated data see **Appendix 2.**

Table of COPASI estimated kinetic constants for the isomerisation of *trans*-fused **3c** ⟶ *cis*-fused **3c** (Table S1)

***trans*-fused 3c**	
k_1	0.0410025
k_2	0.00265045
***cis*-fused 3c**	
k_1	0.0384099
k_2	6.806939283e-16

Graph of experimental data overlayed with COPASI fitted parameters for the isomerisation of *trans*-fused **3c** ⟶ *cis*-fused **3c** (Figure S11)

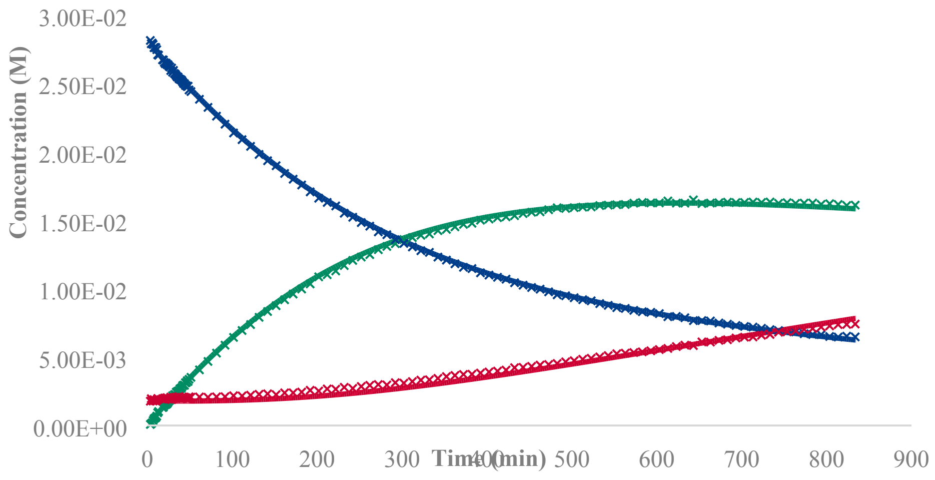

6. Procedure for NMR tracking of reactions

In a vial, a stock solution of CSA (9.2 mg, 0.04 mmol) was made in MeCN-d_3 (0.8 mL). This was placed in a preheated oil bath at 50 °C for 10 min. To this was added salicylaldehyde (**1**, 9.4 microliters, 0.09 mmol). From this stock solution 0.4 mL was taken out and transferred into an NMR tube containing homoallylic alcohol **2** (0.04 mmol). This was then immediately transferred into an NMR spectrometer which had been preheated to 50 °C. The first spectrum was recorded after 3.5 min from addition of starting materials into NMR tube (time required to load sample, lock, shim etc). Spectra were then taken continuously (1 every minute followed by one every 10 min for 12 h).

The reactions were observed to be clean with retention of mass balance. This allowed the concentration to be plotted as a function of time using the initial concentration. This data was then processed as outlined in section 4. For tabulated data see **Appendix 3.**

Graphs of experimental data from ^{1}H NMR with fitted COPASI parameters from data obtained as in section 5 overlayed (Figure S12)

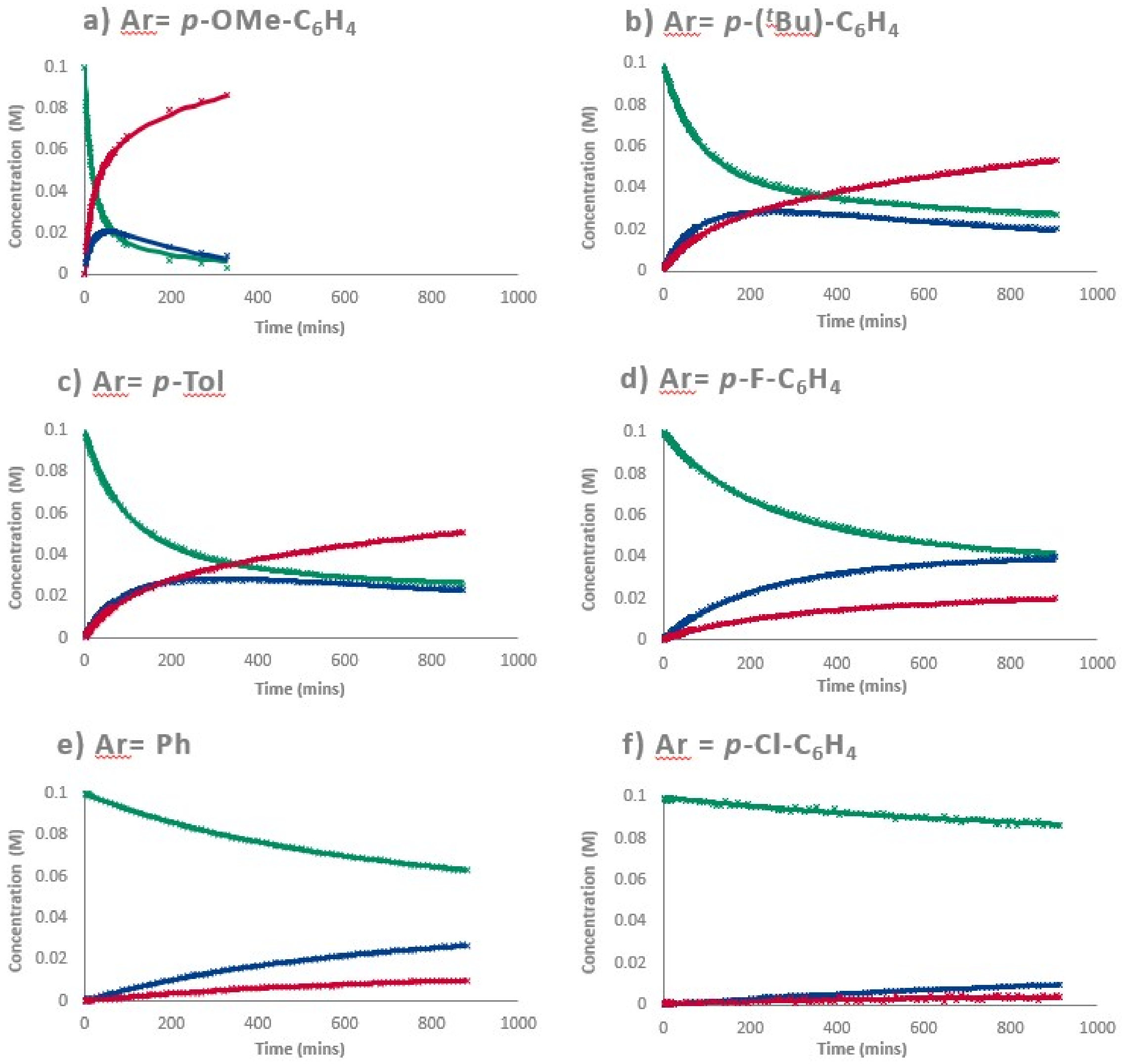

7. Initial rate Hammett plot

For the Hammett plot, conversion was calculated as the consumption of the starting homoallylic alcohol **2** and taken to 20% for the initial rate (or for the 13 h period in the case of **2f** to reach 14% conv.). At low conversions the isomerisation was assumed to be negligible. A line of best fit was taken for these points and displayed in fig with R^2 shown indicating good linearity in all reactions. These initial rates were then divided by k_H, the logarithm taken and plotted against known sigma plus values[5] to yield the Hammett plot as shown in Figure S14 (below).

Table showing conversion of **2** → **3** from ^{1}H NMR integrations (Table S2)

**OMe**		**tBu**		**Me**		**F**		**H**		**Cl**	
**Time (min)**	**Conv (%)**	**Time (min)**	**Conv (%)**	**Time (min)**	**Conv (%)**	**Time (min)**	**Conv (%)**	**Time (min)**	**Conv (%)**	**Time (min)**	**Conv (%)**
3.50	16.73	3.50	3.35	3.50	2.43	3.50	0.81	3.50	0.22	3.50	0.38
4.58	20.87	4.57	4.12	5.27	3.86	4.58	1.20	4.58	0.36	4.58	1.08
		5.65	4.99	6.35	4.58	5.65	1.33	5.67	0.43	5.67	1.74
		6.73	5.86	7.43	5.37	6.73	1.62	6.75	0.53	6.73	1.06

		7.82	6.68	8.50	6.06	7.82	2.07	7.83	0.58	7.82	1.66
		8.90	7.39	9.58	6.80	8.90	2.46	8.90	0.65	8.90	1.08
		9.97	8.18	10.67	7.57	9.98	2.69	9.98	0.78	9.98	0.78
		11.05	8.93	11.75	8.32	11.05	2.98	11.07	0.93	11.07	0.39
		12.13	9.67	12.83	9.01	12.13	3.27	18.48	1.54	12.13	0.59
		13.22	10.35	13.90	9.70	13.22	3.53	28.52	2.36	13.22	1.03
		14.62	11.33	14.98	10.33	14.30	3.82	38.55	3.20	14.30	1.43
		15.70	11.99	16.07	10.89	15.38	4.10	48.58	3.87	15.38	0.58
		16.78	12.64	17.15	11.61	16.45	4.46	58.60	4.74	16.47	0.83
		17.85	13.32	18.23	12.20	17.53	4.66	68.63	5.58	17.53	0.57
		18.93	13.95	19.30	12.89	18.62	5.03	78.67	6.12	18.62	0.82
		20.02	14.58	20.38	13.36	19.70	5.12	88.70	6.82	21.88	0.47
		21.10	15.18	21.47	13.99	20.77	5.52	98.73	7.64	31.92	1.35
		22.18	15.82	22.55	14.58	21.85	5.67	108.77	8.27	41.93	1.10
		23.25	16.33	23.63	15.28	22.93	6.03	118.80	8.97	51.97	2.24
		24.33	16.94	24.70	15.79	24.02	6.40	128.82	9.65	62.00	1.86
		25.42	17.55	25.78	16.26	25.10	6.70	138.85	10.26	72.03	1.67
		26.50	18.11	26.87	16.93	26.17	6.91	148.88	10.83	82.07	3.04
		27.58	18.69	27.95	17.49	27.25	7.27	158.92	11.49	92.10	2.14
		28.65	19.27	29.03	17.88	28.33	7.60	168.95	12.10	102.13	3.04
		29.73	19.69	30.10	18.38	29.42	7.93	178.98	12.67	112.15	3.49
		30.82	20.32	31.18	18.83	30.50	8.11	189.02	13.30	122.18	3.79
				32.27	19.51	31.57	8.44	199.05	13.78	132.22	4.05
				33.35	20.11	32.65	8.63	209.08	14.37	142.25	2.53
						33.73	9.00	219.10	14.95	152.28	4.13
						34.82	9.30	229.13	15.41	162.32	4.20
						35.90	9.39	239.17	16.01	172.35	3.06
						36.98	9.56	249.20	16.56	182.38	3.83
						38.05	9.79	259.23	17.07	192.42	3.30
						39.13	10.10	269.27	17.29	202.43	4.65
						40.22	10.40	279.30	17.96	212.47	4.96
						41.30	10.59	289.33	18.48	222.50	4.96
						42.38	10.84	299.35	18.92	232.53	5.13
						43.45	11.12	309.38	19.59	242.57	4.89
						44.53	11.32	319.42	19.68	252.60	5.61
						45.62	11.48	329.45	20.38	262.63	6.46
						46.70	11.75			272.67	6.10
						47.77	11.99			282.68	5.97
						48.85	12.00			292.72	6.96
						49.93	12.38			302.75	4.73
						51.02	12.63			312.78	6.41
						52.10	12.78			322.82	7.16
						53.17	13.16			332.85	5.67
						54.25	13.27			342.88	6.30

						55.33	13.57			352.92	5.39
						56.42	13.59			362.93	6.68
						57.50	13.98			372.97	7.49
						58.57	14.06			383.00	6.73
						59.65	14.35			393.03	5.86
						60.73	14.51			403.07	8.51
						61.82	14.73			413.10	8.21
						62.90	14.86			423.13	7.11
						63.97	15.08			433.17	8.31
						65.05	15.17			443.18	7.54
						66.13	15.55			453.22	7.45
						67.22	15.79			463.25	8.71
						68.70	15.97			473.28	8.78
						78.40	17.65			483.32	8.46
						88.10	19.29			493.35	8.00
						97.78	20.71			503.38	7.82
										513.40	9.11
										523.43	8.80
										533.47	10.23
										543.50	9.32
										553.53	9.51
										563.57	9.60
										573.60	9.60
										583.63	9.00
										593.65	9.76
										603.68	9.35
										613.72	11.22
										623.75	10.21
										633.78	10.29
										643.82	9.21
										653.85	11.61
										663.88	11.64
										673.90	10.03
										683.93	11.97
										693.97	11.43
										704.00	10.98
										714.03	10.77
										724.07	10.56
										734.10	10.38
										744.13	11.65
										754.15	11.68
										764.18	12.72
										774.22	12.69
										784.25	11.88

										794.28	13.17
										804.32	11.88
										814.35	13.18
										824.38	12.46
										834.40	11.77
										844.43	12.39
										854.47	12.74
										864.50	11.35
										874.53	14.08
										884.57	12.74
										894.60	13.11
										904.63	14.22
										914.67	13.37

Graph of initial rates for conversion of **2** → **3** from ^{1}H NMR integrations (Figure S13)

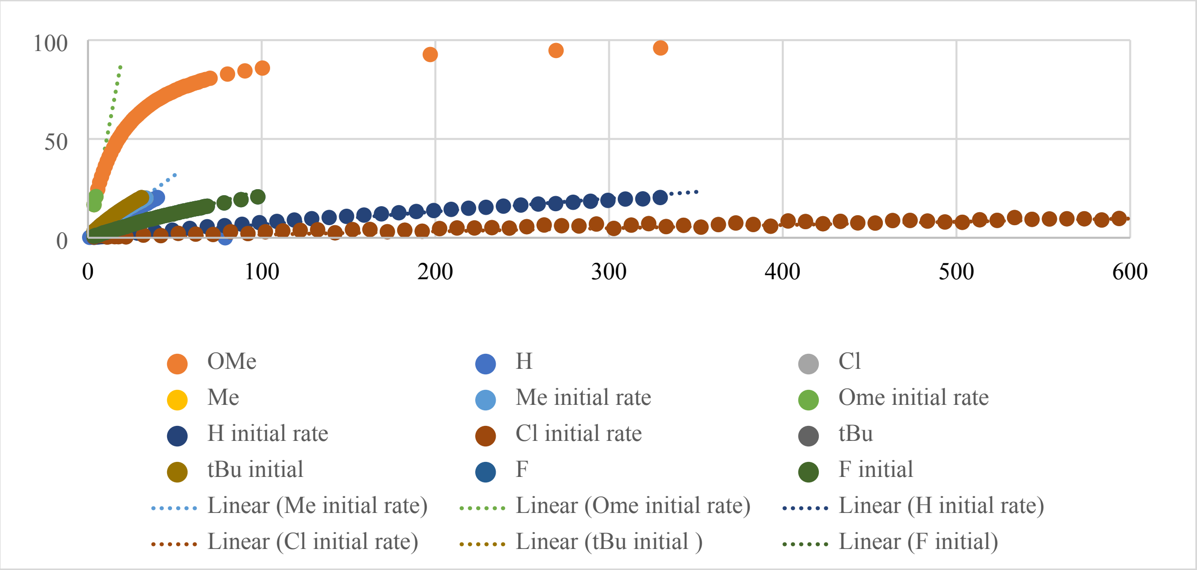

Table of initial rates taken from Figure S13 (Table S3)

**Substrate**	**kobs**	**kH**	**kobs/kH**	**Sigma plus value**	**log (kobs/kH)**
OMe **(3a)**	4.6365	0.0664	69.82681	-0.648	1.844022
tBu **(3b)**	0.707	0.0664	10.64759	-0.275	1.027251
Me **(3c)**	0.6356	0.0664	9.572289	-0.256	0.981016
F **(3d)**	0.2411	0.0664	3.631024	-0.247	0.560029
H **(3e)**	0.0664	0.0664	1	0	0
Cl **(3f)**	0.0162	0.0664	0.243976	0.035	-0.61265

Hammett plot obtained using experimental initial rate data from Table S3 (Figure S14)

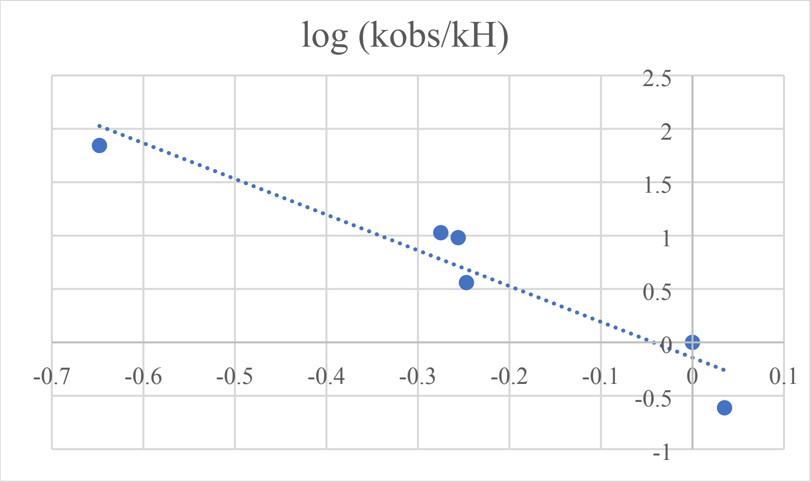

8. COPASI modelling for Hammett plot

Using COPASI, the experimental full reaction course data collected for the six reactions were taken and modelled. Inputting the reaction progress from ^{1}H NMR and using the scatter search method with 200 iterations led to curves with good levels of fitting. The lower bound for all reactions was set to zero with an upper bound of infinity. Alternative methods were trialled sequentially to ensure correct best fit identification. The start point was not randomised to ensure sequential optimisation. Optimisation was repeated until the best value was found and global minimum was confirmed by altering parameters and observing alternative local minimum. Initially, both the formation of *cis*-fused- and *trans*-fused diastereomers of **3** were modelled as reversible reactions and this resulted in the following results. The readout was overlayed with the experimental data to verify the accuracy of the fitting.

Table of COPASI fitted constants – modelled assuming all reactions are reversible (Table S4)

	**OMe**	**tBu**	**Me**	**F**	**H**	**Cl**
***trans*-fused**						
k1	0.1495390000	0.0401996000	0.033506	0.0153274000	0.0051266100	0.0012
k2	0.0073581800	0.0034085500	0.002402	0.0000950276	0.0005311841	0.000446275
kx/KH	29.16917807	7.841361055	6.535722	2.989772969	1	0.234072808
log	1.464924192	0.894391451	0.815294	0.475638211	0	-0.630649034
***cis*-fused**						
k1	0.3055330000	0.0269871000	0.026569	0.0062269500	0.0017813100	0.000801261
k2	0.0000000000	0.0002704680	0.000263	0.0005132280	0.0003378700	0.00187609
kx/KH	171.5215207	15.15014231	14.91543	3.495713829	1	0.449815585
log	2.234318618	1.180416712	1.173636	0.543535873	0	-0.346965501
**Both**						
Total sum	0.4550720000	0.0671867000	0.060075	0.0215543500	0.0069079200	0.00
total Kx/kH	65.8768486	9.726039097	8.696554	3.120237351	1	0.29
log total kx/KH	1.818732815	0.987936011	0.939347	0.494187631	0	-0.538043567

Hammett plot for the formation of *trans*-fused-**3** – modelled assuming all reactions are reversible (Figure S15)

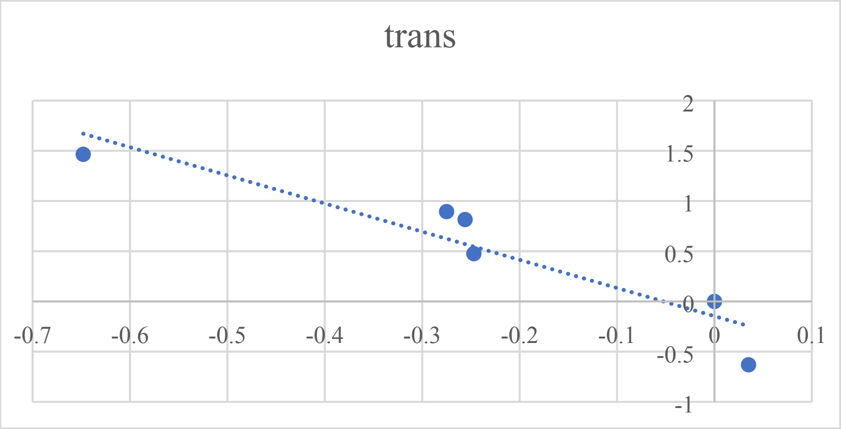

Hammett plot for the formation of *cis*-fused-**3** – modelled assuming all reactions are reversible (Figure S16)

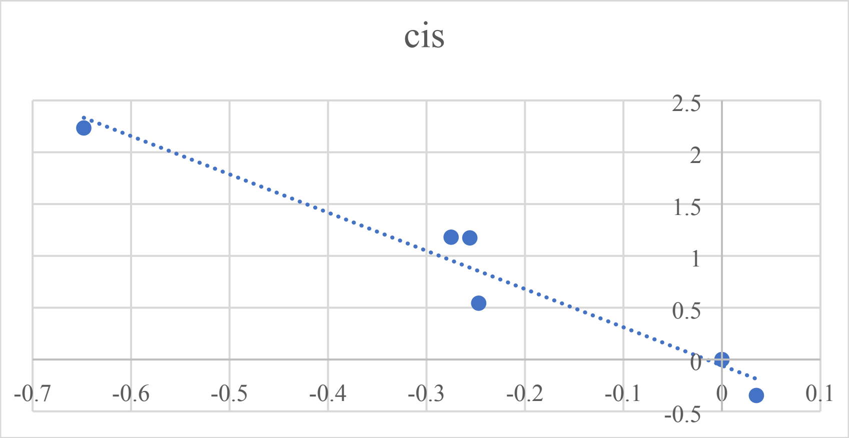

Hammett plot for the formation of *cis*-fused- and *trans*-fused-**3** – modelled assuming all reactions are reversible (Figure S17)

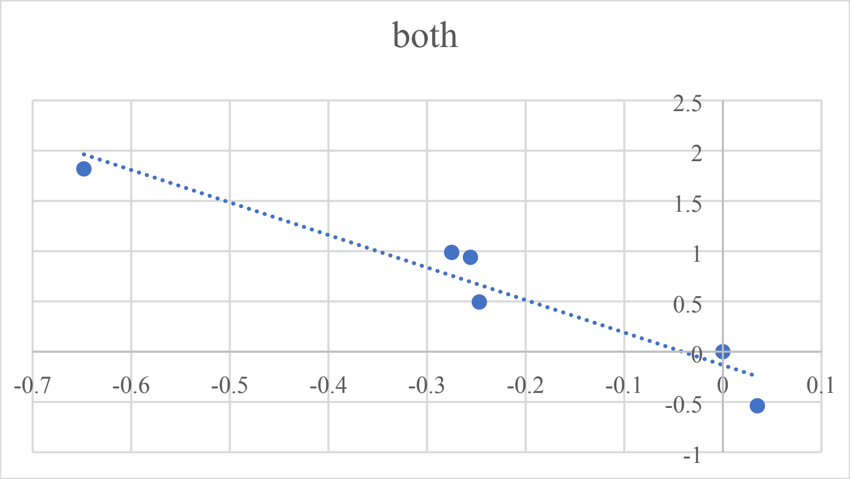

Modelling was also carried out assuming that the formation of the *trans*-fused diastereomer **3** was reversible but the formation of *cis*-fused diastereoisomer **3** was *irreversible*. This was done to mirror the experimental observation that reversion of *cis*-fused products **3** back to starting materials never

occurred (fitted graphs with experimental NMR data not shown). This alternative method of modelling yielded comparable results to that which assumed all reactions are reversible but gave a marginally poorer R^2 fit. Both modelling methods indicated a notable difference in ρ^+ between the formation of the *cis*-fused- and *trans*-fused products **3**. Only the all-reversible derived data is reported in the main manuscript as irreversibility is usually reserved for reactions leading to gas formation.

Table of COPASI fitted constants – modelled assuming reversible formation of *trans*-fused **3** and irreversible formation of *cis*-fused **3** (Table S4)

***trans*-fused**	**OMe**	**tBu**	**Me**	**F**	**H**	**Cl**
k1	0.1496320000	0.0391542000	0.034133	0.0157484000	0.0052193200	0.00139988
k2	0.0073580200	0.0032770500	0.00246199	0.0010538600	0.0000591610	0.000822776
kx/KH	28.66886874	7.501781841	6.539741	3.017327928	1	0.268211185
log	1.457410556	0.87516443	0.815561	0.479622513	0	-0.571523116
***cis*-fused**						
k1	0.3056720000	0.0243226000	0.0240244	0.0051320600	0.0015818700	0.000449815
kx/KH	193.2345894	15.37585263	15.18734	2.301337104	1	0.081008476
log	2.286084869	1.186839208	1.181482	0.36198024	0	-1.091469538
**Both**						
k1	0.455304	0.0634768	0.0581574	0.02088046	0.00680119	0.001849695
Kx/KH	66.94475526	9.333190221	8.551062388	3.070118612	1	0.271966376
Log(Kx/KH)	1.825716558	0.970030117	0.932020075	0.487155154	0	-0.565484785

Hammett plot for the formation of *trans*-fused-**3** – modelled assuming reversible formation of *trans*-fused **3** and irreversible formation of *cis*-fused **3** (Figure S18)

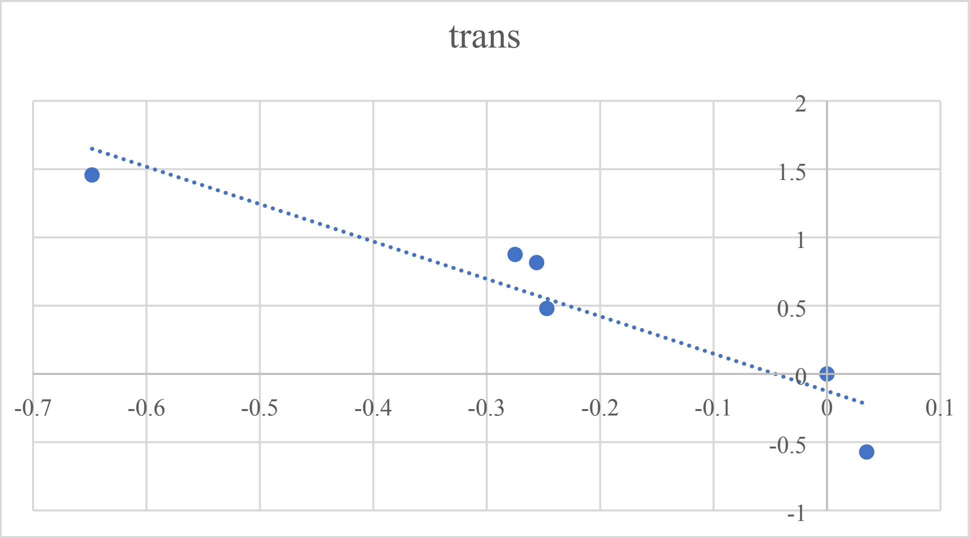

Hammett plot for the formation of *cis*-fused-**3** – modelled assuming reversible formation of *trans*-fused **3** and irreversible formation of *cis*-fused **3** (Figure S19)

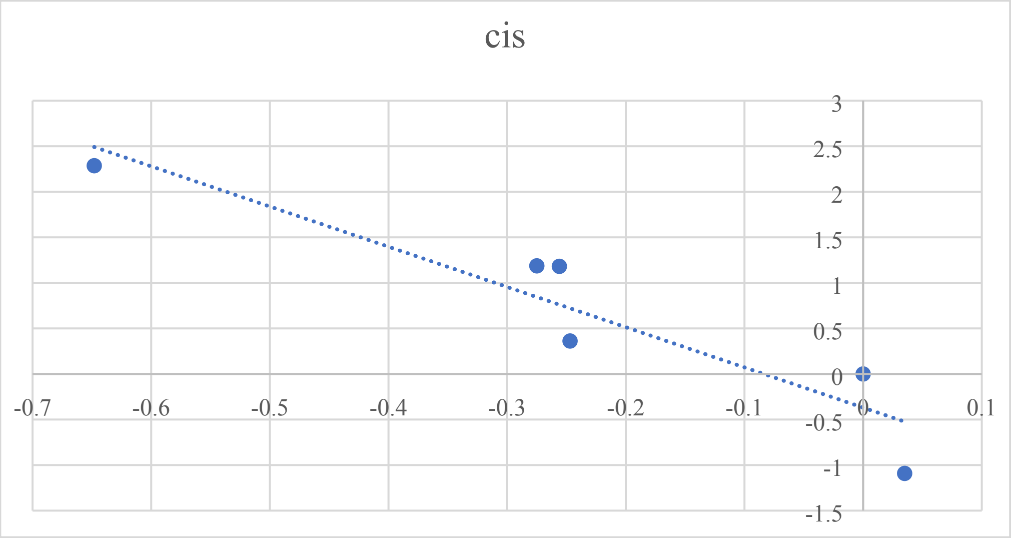

Hammett plot for the formation of *cis*-fused- and *trans*-fused-**3** – modelled assuming reversible formation of *trans*-fused **3** and irreversible formation of *cis*-fused **3** (Figure S20)

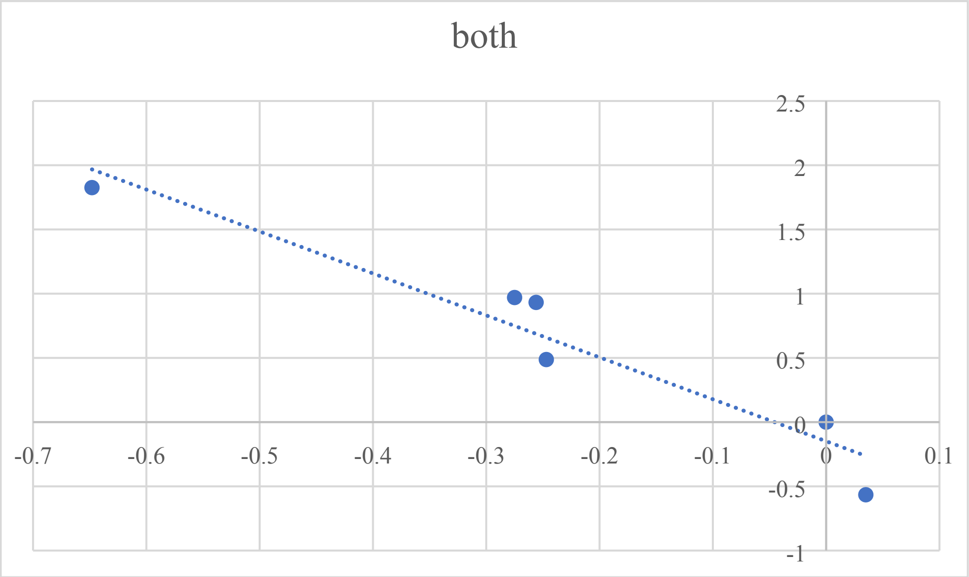

9. DFT modelling

All DFT results including computed structures *etc*. can be found in the FAIR Data Archive (DOI: 10.14469/hpc/3943) and sub-collections therein.

As discussed in the main manuscript, inspection of the HOMO in the *endo* TSs suggested π-π stacking being significant in the more electron rich TSs and less so in the more electron deficient TSs. This is also evident from inspection of their NCI surfaces (Figures S21-22).

Computed NCI of the *endo* TS[1] (p-NH_2 NCI) showing a significant attractive interaction surface (Figure S21)

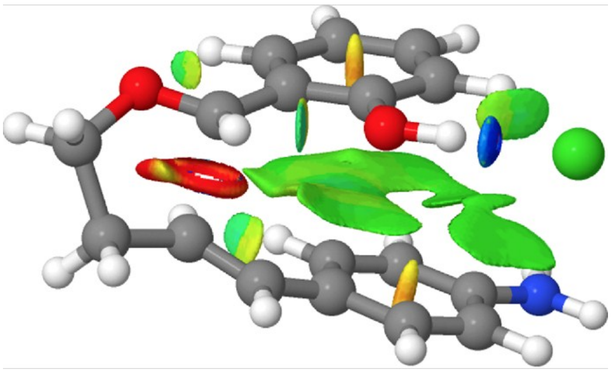

Computed NCI of the *exo* TS[1] (p-NH_2 NCI) showing a mimimal interaction surface (Figure S22)

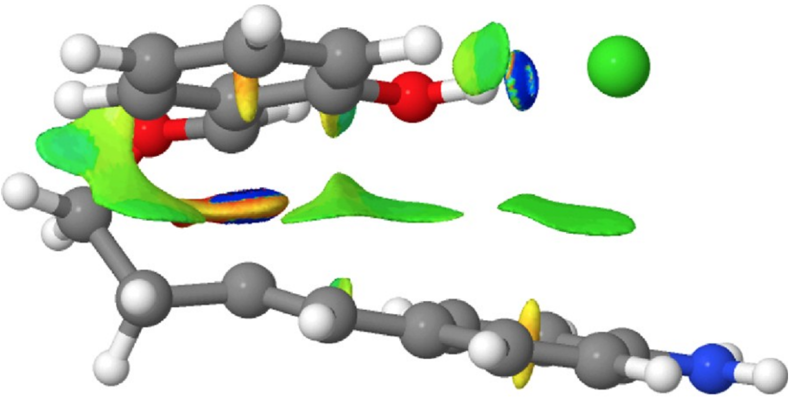

To corroborate our experimental Hammett analysis, we sought a comparison with a computed value. Key numbers have been extracted from Gaussian files and are reported here. For Gaussian files, the FAIR Data Archive, the relevant ending of DOIs are listed in the table. To extend the range of Hammett analysis we computed energies for reactions using homoallylic alcohols **2** with a wider range of substituents than experimentally, namely *p*-NH2, *p*-OMe (**2a**), *p*-H (**2e**), *p*-Cl (**2f**), *p*-CN and *p*-NO_2.

Table showing the key DOIs and associated energies for the computed reaction **2** → **3** pathways using HCl catalysis (Table S5)

***p*-NH_2**				
doi ending	SM	doi ending	TS1, *endo*	dG (kcal/mol)
3966	-1324.25	3944	-1324.25	4.0266675
			TS2, *endo*	
		3978	-1324.24	6.361595
			TS1, *exo*	
		4080	-1324.24	12.2142875
			TS2, *exo*	
		4122	-1324.2	6.0667
			Trans-fused product	
		4088	-1324.25	-0.194525
			Cis-fused product	
		4082	-1324.26	-5.0432175

***p*-OMe**				
doi ending	SM	doi ending	TS1, *endo*	dG (kcal/mol)
3969	-1383.43	3962	-1383.42	8.4505425
			TS2, *endo*	
		3981	-1383.42	8.480035
			TS1, *exo*	
		4097	-1383.4	16.499485
			TS2, *exo*	
		4117	-1383.41	9.588827
			Trans-fused **3a**	
		4089	-1383.43	-0.67017
			Cis-fused **3a**	
		4083	-1383.44	-4.8725375
***p*-Me**				
doi ending	SM	doi ending	TS1, *endo*	dG (kcal/mol)
4153	-1308.19	4152	-1308.18	9.846103
			TS2, *endo*	
		4163	-1308.177	9.99231
			TS1, *exo*	
		4147	-1308.17	18.43846
			TS2, *exo*	
		4173	-1308.17	11.63573
			Trans-fused **3c**	
		4138	-1308.16	-1.31838
			Cis-fused **3c**	
		4136	-1308.20	-5.24653
***p*-H**				
doi ending	SM	doi ending	TS1, *endo*	dG (kcal/mol)
3965	-1268.88	3950	-1268.86	13.005565
			TS2, *endo*	
		3974	-1268.86	11.50584
			TS1, *exo*	
		4074	-1268.85	20.03482
			TS2, *exo*	
		4075	*Trans*-fused **3e**	
			-1268.88	-0.192015
			Cis-fused **3e**	
		4079	-1268.89	-4.74892
				-796225.4417
***p*-Cl**				
doi ending	SM	doi ending	TS1, *endo*	dG (kcal/mol)

3967	-1728.52	3968	-1728.5	12.2808025
			TS2, *endo*	
		3977	-1728.5	11.8390425
			TS1, *exo*	
		4087	-1728.49	20.723815
			TS2, *exo*	
			Trans-fused **3f**	
		4090	-1728.52	-0.1123225
			Cis-fused **3f**	
		4084	-1728.53	-4.469055
***p*-CN**				
doi ending	SM	doi ending	TS1, *endo*	dG (kcal/mol)
3964	-1361.17	3945	-1361.14	17.618945
			TS2, *endo*	
		3993	-1361.14	18.0211725
			TS1, *exo*	
		4081	-1361.13	23.4226925
			TS2, *exo*	
			Trans-fused product	
		4094	-1361.17	0.171935
			Cis-fused product	
		4085	-1361.18	-3.9350525
***p*-NO2**				
doi ending	SM	doi ending	TS1, *endo*	dG (kcal/mol)
3971	-1473.48	3972	-1473.45	18.2420525
			TS2, *endo*	
		3992	-1473.45	19.8534725
			TS1, *exo*	
		4096	-1473.44	25.2625225
			TS2, *exo*	
			Trans-fused product	
		4095	-1473.48	0.952545
			Cis-fused product	
		4086	-1473.49	-3.2473125

Inspection of the Gibbs free energy of the *trans*-fused *p*-CN and *p*-NO_2 products suggested that these reactions would be endergonic and therefore unfeasible (as shown in table S5). As such they were not included in our Hammett analysis. Values for σ^+ were taken from ref. 5.

Table of computed Hammett parameters for the reaction **2** → **3**, utilizing values shown in Table S5 (HCl catalyzed) as well as for the uncatalyzed process (Table S6).

***trans*-fused**	**dG (kcal mol^{-1})**	**J mol^{-1}**	**lnk**	**k**	**kx/k$_H$**	**σ$^+$**	**log kx/K$_H$**
p-NH2	6.36	26610.24	-9.904546861	4.99471E-05	31450.47	-1.11	4.497627
p-OMe	8.48	35480.32	-13.20606248	1.83942E-06	1158.236	-0.648	3.063797
p-Me	10.04	42007.36	-15.63547964	1.62031E-07	102.0269	-0.256	2.008715
p-Cl	12.28	51379.52	-19.1238735	4.95002E-09	3.116912	0.035	0.493724
p-H	13.01	54433.84	-20.26071614	1.58812E-09	1	0	0
***cis*-fused**							
p-NH2	12.21	51086.64	-19.01486119	5.52015E-09	194670.1	-1.11	5.289299
p-OMe	16.5	69036	-25.69575836	6.92586E-12	246.0767	-0.648	2.39107
p-Me	18.48	77320.32	-28.77924937	3.17198E-13	11.26105	-0.256	1.051579
p-Cl	20.06	83931.04	-31.2398129	2.70846E-14	0.95511	0.035	-0.01995
p-H	20.03482	83825.69	-31.20059961	2.81677E-14	1	0	0
p-CN	23.42	97989.28	-36.47240369	1.44623E-16	0.005113	0.674	-2.29134
p-NO_2	25.26	105687.8	-39.33787008	8.23721E-18	0.000291	0.74	-3.53568
**Uncatalyzed**							
p-NH2	15.01	62801.84	-23.37535352	7.05039E-11	15850.38	-1.11	4.20004
p-OMe	18.3	76567.2	-28.498932	4.19828E-13	94.38391	-0.648	1.974898
p-Me	20.47	85646.48	-31.87831356	1.43029E-14	3.21552	-0.256	0.507251
p-Cl	18.51	77445.84	-28.82596893	3.0272E-13	68.05619	0.035	1.832868
p-H	21.22	88784.48	-33.04630258	4.44809E-15	1	0	0
p-CN	21.3	89119.2	-33.17088807	3.92705E-15	0.882863	0.674	-0.05411
p-NO_2	21.34	89286.56	-33.23318082	3.68989E-15	0.829545	0.74	-0.08116

Computed Hammett plot for *trans*-fused product **3** formation using values as shown in Table S5 (Figure S23)

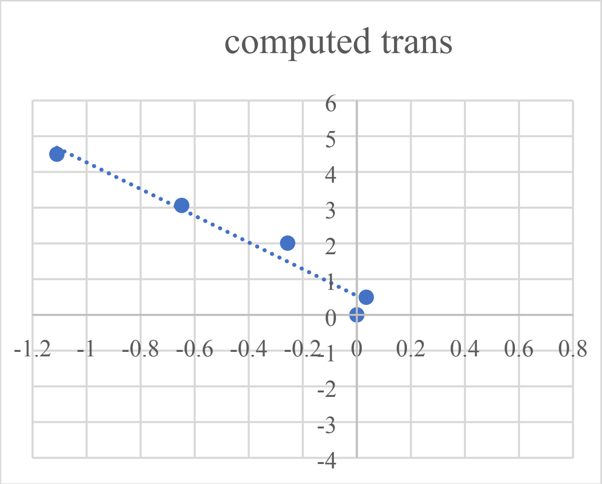

Computed Hammett plot for *cis*-fused product **3** formation using values as shown in Table S5 (Figure S24)

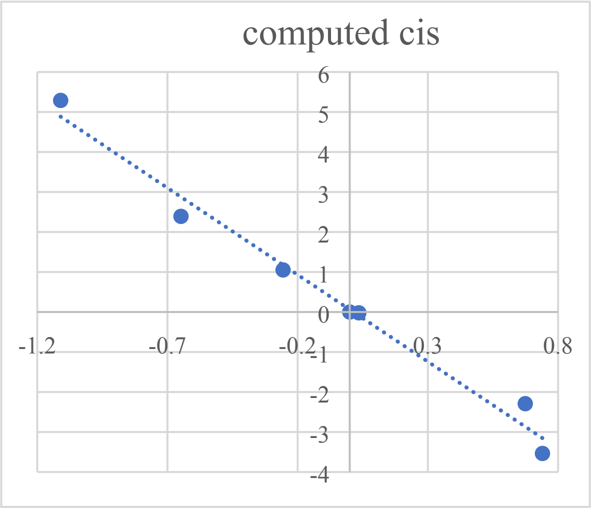

Computed Hammett plot for *trans*-fused product **3** formation using values as shown in Table S5 (Figure S25)

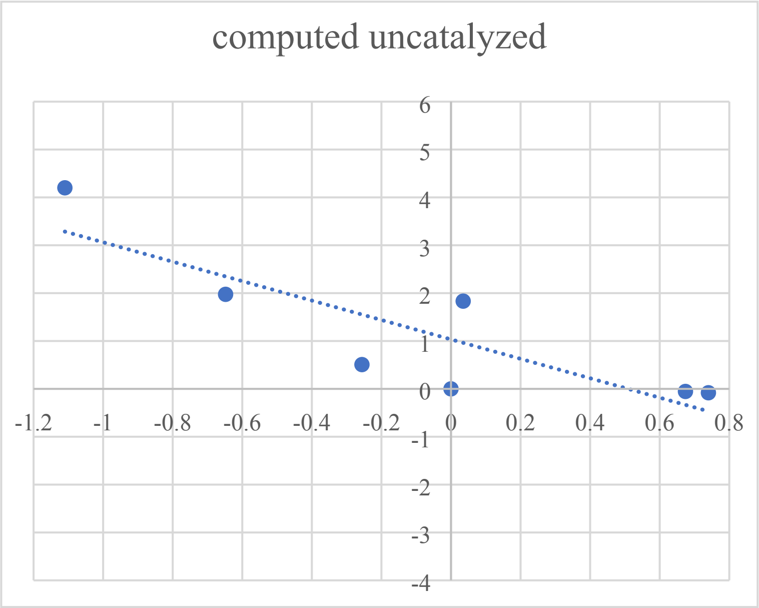

10. Determination of the optimal quantity of HFIP

The reaction to form products **3c** was used to evaluate the effect of HFIP as an additive in $CDCl_3$ as solvent for these reactions:

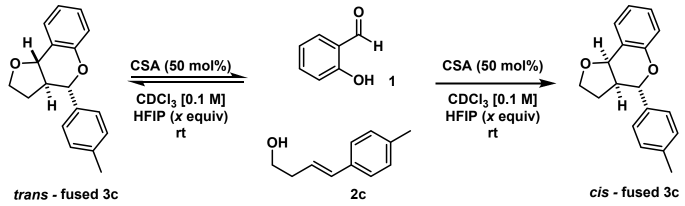

To an NMR tube was added homoallylic alcohol **2c** (6.5 mg, 0.04 mmol, 1 equiv). CSA (4.6 mg, 0.02 mmol, 0.5 equiv) was solubilised in $CDCl_3$ and HFIP of varying amounts to make up a total volume of

0.4 mL. To this solution salicylaldehyde (**1**, 4.7 µL, 0.044 mmol, 1.1 equiv) was added and transferred to the NMR tube. A ^{1}H NMR was taken after 10 min and analysed to identify optimal conditions. 12 equivalents of HFIP was found to be optimal.

Bar chart to show effect of number of equiv. of HFIP on the conversion of **2c** ⟶ **3c** after 10 min (Figure S26)

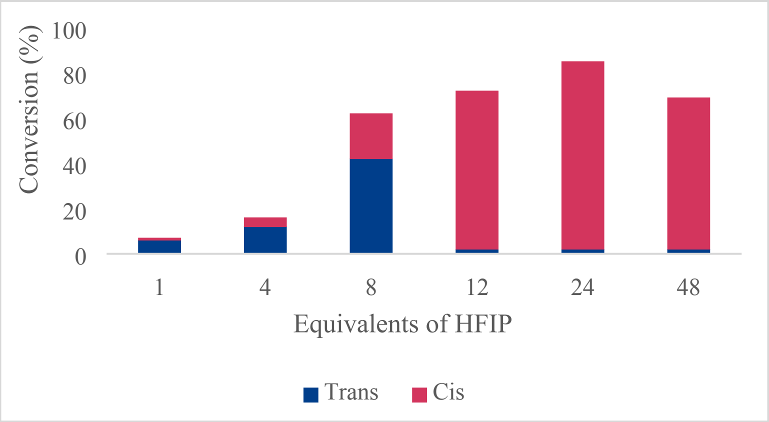

11. NMR tracking of reactions using HFIP as an additive

In a vial, a stock solution of CSA (13.8 mg, 0.06 mmol) was made in chloroform-*d* (1.05 mL) and HFIP (0.15 mL). To this was added salicylaldehyde (13.3 µL, 0.13 mmol). From this stock solution 0.6 mL was taken out and transferred into and NMR tube containing homoallylic alcohol (0.06 mmol). This was then immediately transferred into an NMR spectrometer. The first spectrum was recorded after 3.5 min from addition of starting materials into NMR tube (time required to load sample, lock, shim etc). The reaction was monitored by NMR and quenched by addition of $NaHCO_3$ (sat. soln.) after the stated time. The organic layer was extracted with CH_2Cl_2, dried over anhydrous Na_2SO_4 and concentrated under a stream of N_2. The product was isolated by flash column chromatography 25% diethyl ether/petroleum ether.

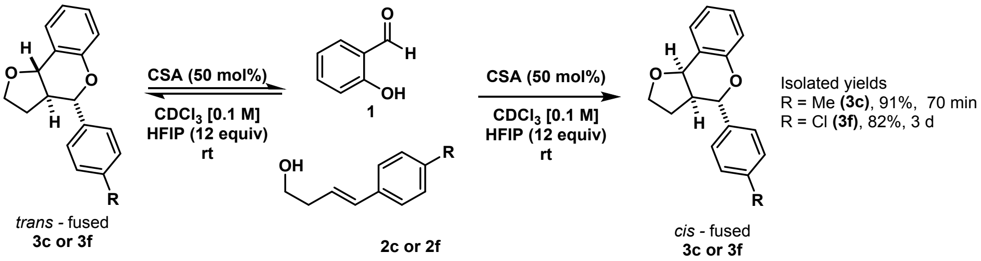

COPASI was used to model these reactions, again assuming all steps are reversible.

Graph of reaction progress monitored by ^{1}H NMR for the reaction **2c** → **3c** (*p*-Me) using HFIP (12 equiv.) as an additive (Figure S28). For tabulated data see **Appendix 4.**

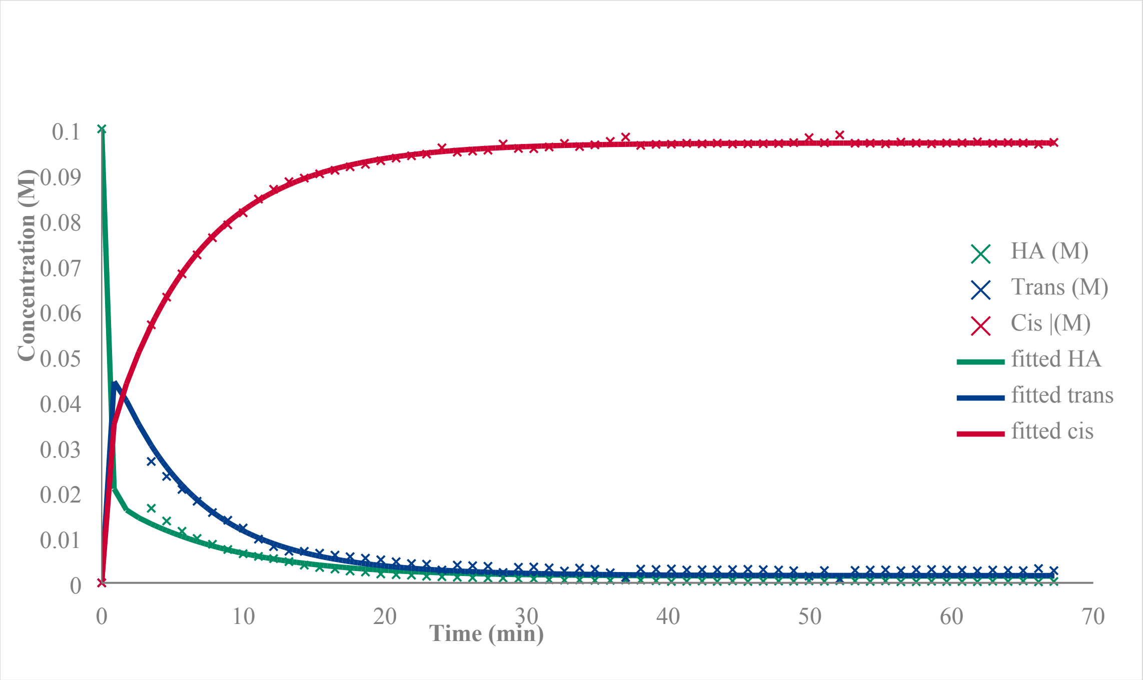

Graph of reaction progress monitored by ^{1}H NMR for the reaction **2f** → **3f** (*p*-Cl) using HFIP (12 equiv.) as an additive (Figure S29). For tabulated data see **Appendix 5**.

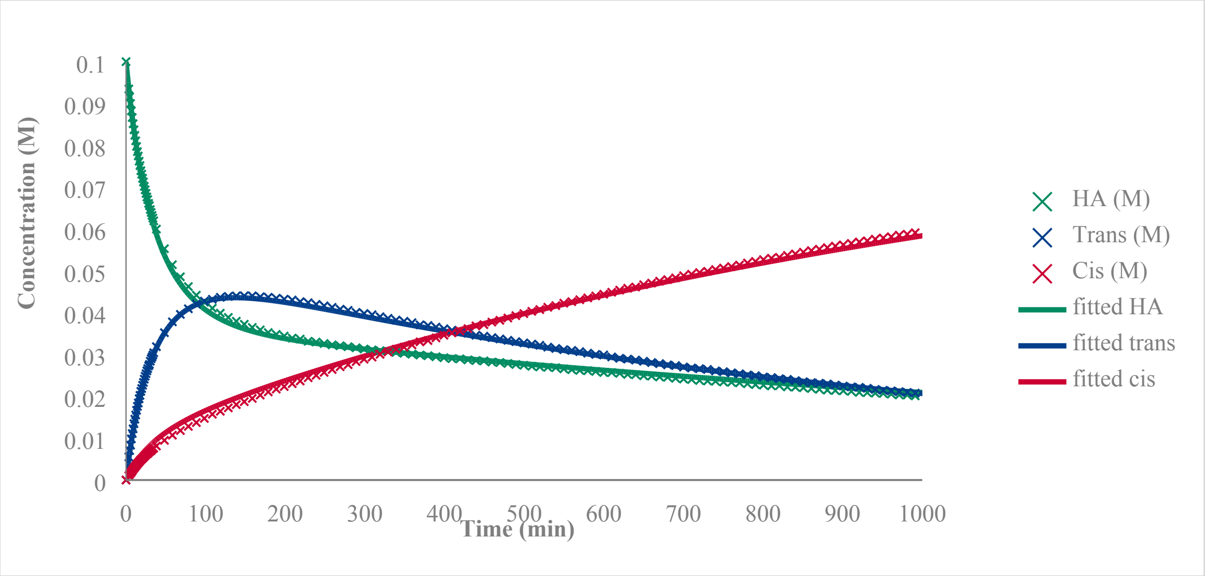

Table of COPASI fitted parameters comparing MeCN as solvent *vs*. $CDCl_3$ with HFIP (12 equiv.) as additive (Table S7)

		**Me (MeCN)**	**Cl (MeCN)**	**Me (HFIP)**	**Cl (HFIP)**
	trans-fused				
Forward	k1	0.0335066	0.00124431	27.0841	0.1303
Back	k2	0.00240198	0.000446367	0.5435	0.0063

	cis-fused				
Forward	k1	0.0265691	0.000801215	15.3902	0.0347
Back	k2	0.000262939	0.00187587	0.0051	0.0000

Comparison of the kinetic constants shows that the HFIP additive yields a ~100-fold increase in the rate (*i.e*. k1).

12. Homoallylic alcohol alkene scrambling experiments

Two experiments were performed. In the first, homoallylic alcohol **2c** was treated with CSA in $CDCl_3$ with HFIP (12 equiv.) in the presence of salicylaldehyde (**1**) to see if isomerization occurred during furanochromane formation. In the second, salicylaldehyde (**1**) was not added to see if isomerization occurred just upon subjecting the homoallylic alcohol to the CSA in $CDCl_3$ with HFIP (12 equiv.).

Reaction in presence of salicylaldehyde (**1**):

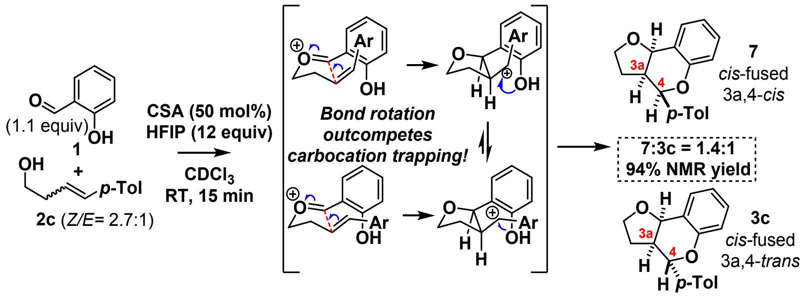

(*E/Z*)-4-(*p*-Tolyl)but-3-en-1-ol (*Z/E*= 2.7/1) **2c** (6.5 mg, 0.04 mmol, 1 equiv) was weighed into an NMR tube. In a separate vial, CSA (4.6 mg, 0.02 mmol, 0.5 equiv) was dissolved in 0.05 mL HFIP and 0.35 mL $CDCl_3$. To this vial, was added salicylaldehyde 1 (4.4 µL, 0.04 mmol, 1.1 equiv). This was then transferred to an NMR tube and subjected to ^{1}H NMR analysis. Within 15 min, inspection of the ^{1}H NMR revealed complete consumption of the alkene in the presence of salicylaldehyde. This reaction was then quenched by addition of the $NaHCO_3$ (sat. soln.), extracted with $CH_2Cl_2$and analysed by ^{1}H NMR to show a 1.4:1 mixture of *cis*-fused-3a,4-*cis*-**7** and *cis*-fused-3a,4-*trans*-**3c**.

(3a*SR*,4*RS*,9b*RS*)-4-(*p*-Tolyl)-2,3,3a,9b-tetrahydro-4H-furo[3,2-c]chromene (7)

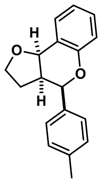

^{1}H NMR (400 MHz, Chloroform-*d*) δ_H (8 Aromatic protons overlayed with *cis*-fused-**3c** and unreacted excess salicylaldehyde). 5.34 (d, *J* = 8.2 Hz, 1H), 5.26 (d, *J* = 2.3 Hz, 1H), 3.80 – 3.74 (m, 2H), 2.93 (dtd, *J* = 10.6, 8.4, 2.3 Hz, 1H), 2.16 – 2.01 (m, 1H overlayed with with *cis*-fused-**3c**), 1.76 – 1.64 (m, 1H-overlayed with *cis*-fused-**3c**). ^{13}C NMR (101 MHz, Chloroform-*d*) δ_C (12 Aromatic carbons) overlayed with *cis*-fused-**3c** and unreacted excess salicylaldehyde 77.0, 74.8, 67.0, 44.6, 21.2, 24.2 (aromatic carbons

^{1}H NMR of crude reaction mixture showing complete consumption of homoallylic alcohol **2c** and formation of furanochromanes **7** and **3c** with ratio ~1.4:1 (Figure S30).

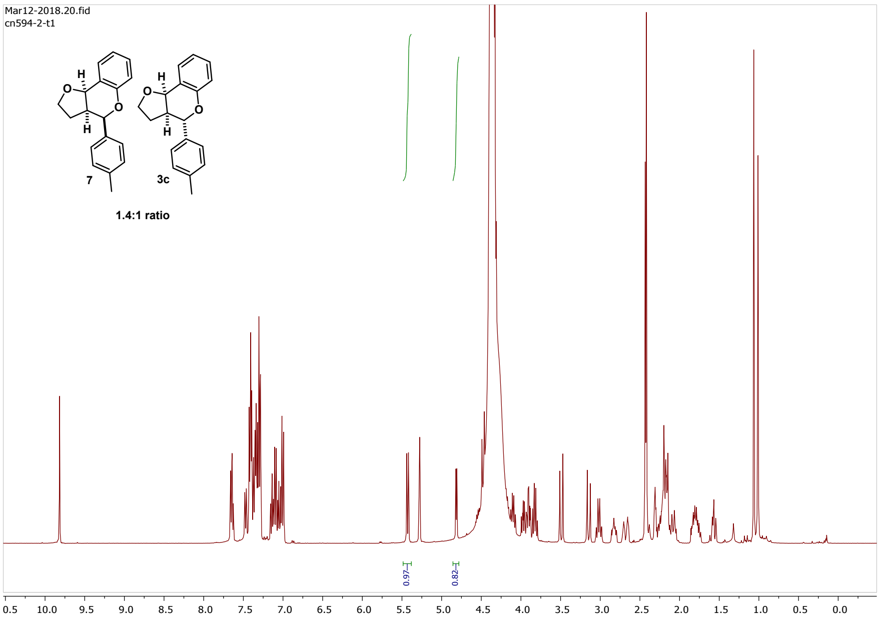

^{1}H NMR of reaction mixture after workup showing furanochromanes **7** and **3c** with ratio ~1.4:1 (Figure S31)

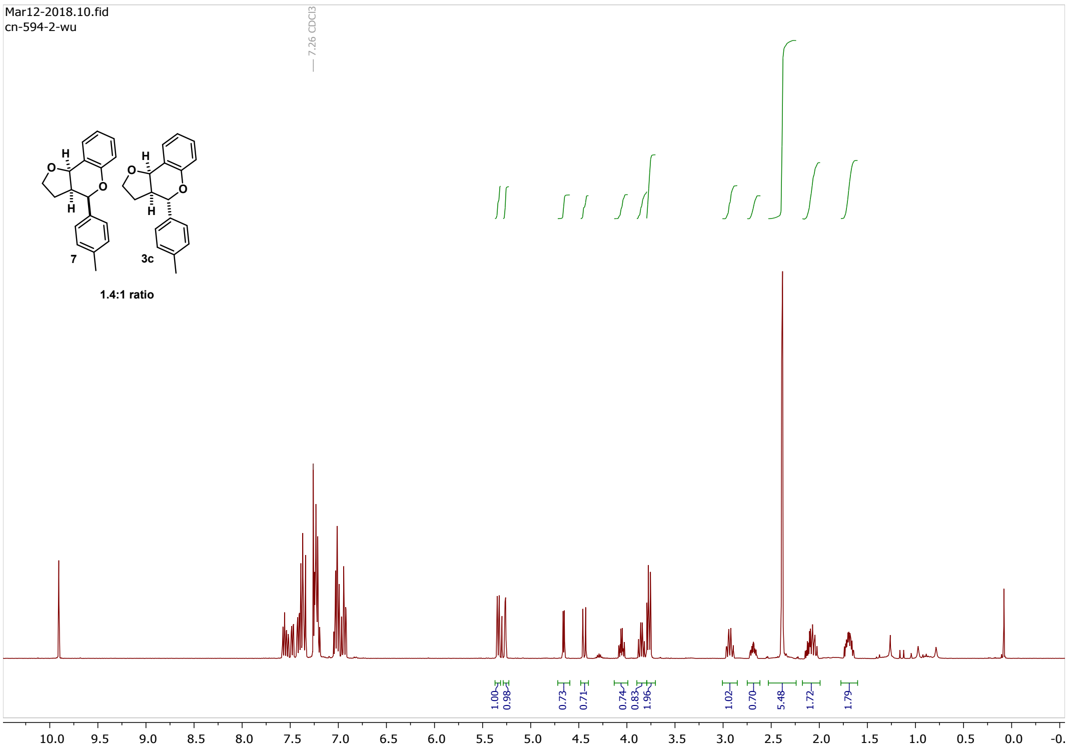

DEPT 135 ^{13}C NMR of reaction mixture after workup showing furanochromanes **7** and **3c** with ratio ~1.4:1 (Figure S32)

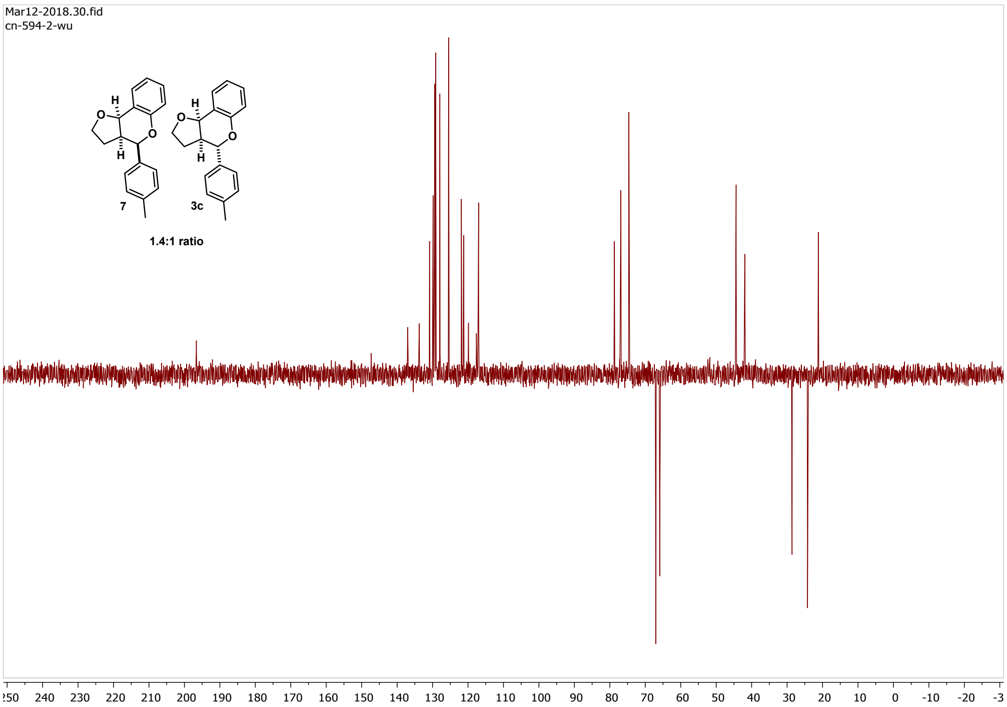

HSQC NMR of reaction mixture after workup showing furanochromanes **7** and **3c** with ratio ~1.4:1 (Figure S33)

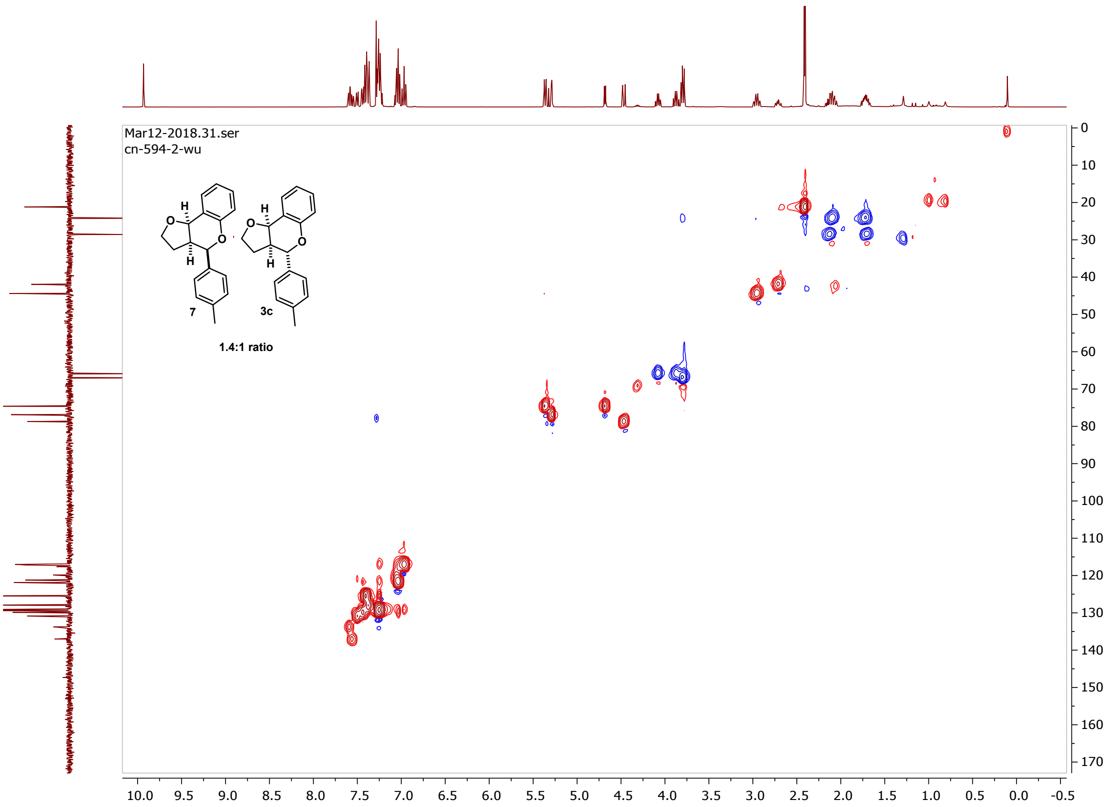

COSY NMR of reaction mixture after workup showing furanochromanes **7** and **3c** with ratio ~1.4:1 (Figure S34)

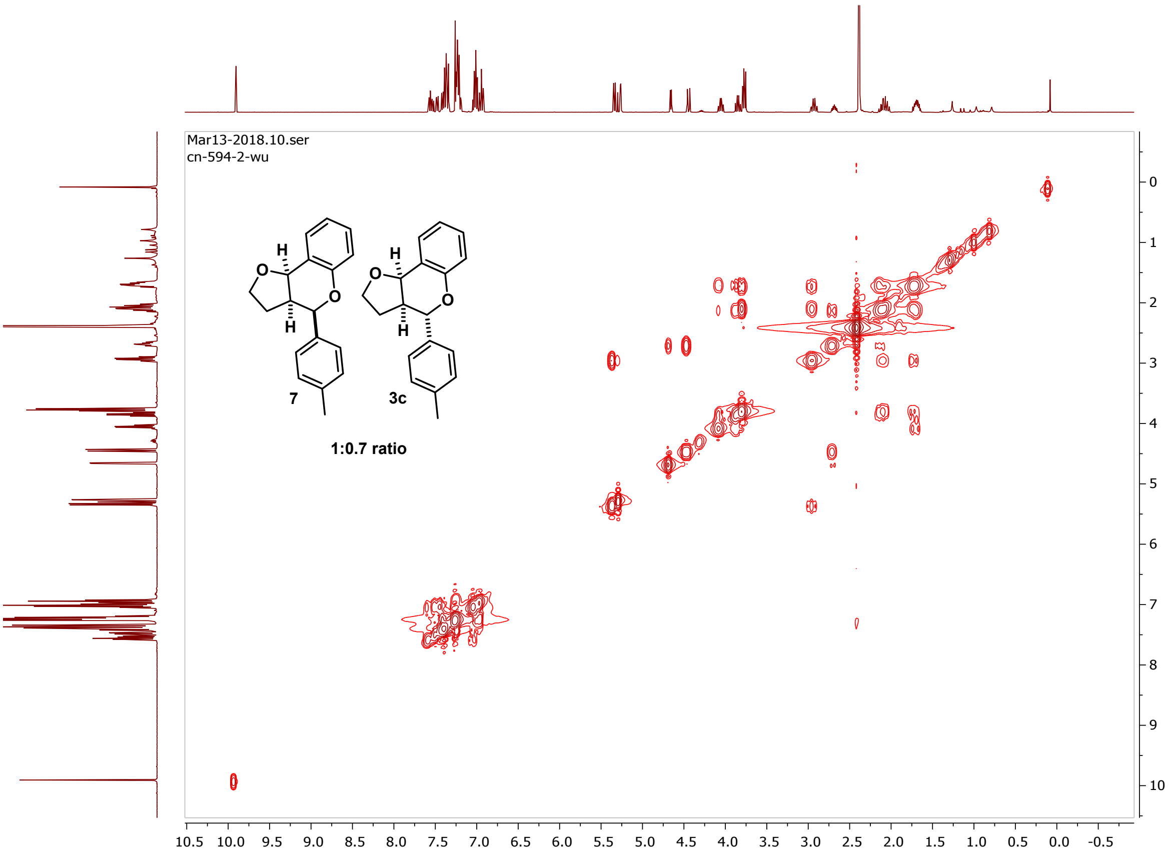

Reaction in absence of salicylaldehyde (**1**):

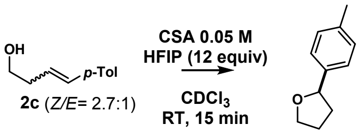

As for the above reaction but using 0.02 mmol of homoallylic alcohol and 0.02 mmol of CSA dissolved in 0.05 mL HFIP and 0.35 mL $CDCl_3$. 2-Tolyl-tetrahydrofuran was cleanly formed (70% conversion after 5 h) with the ratio of unreacted *E/Z* isomers of the starting homoallylic alcohol **2c** remaining unchanged.

^{1}H NMR in the absence of salicylaldehyde (**1**) showing initial formation of the 2-tolyltetrahydrofuran cyclisation product and unreacted homoallylic alcohol **2c** with Z/E ratio ~2.7:1 (Fig S35)

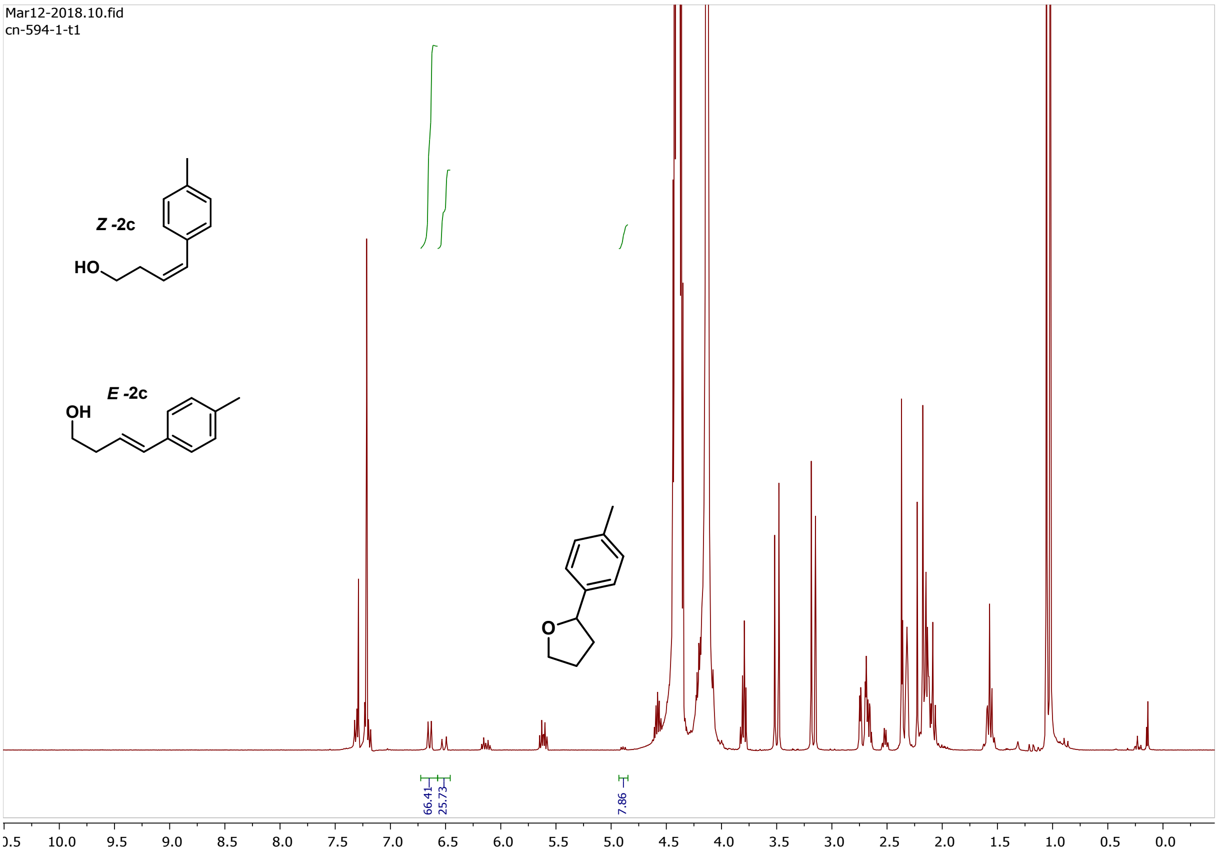

13. Oxonium-Prins products

(3a*RS*,4*RS*,9b*RS*)-4-(*p*-Tolyl)-2,3,3a,9b-tetrahydro-4H-furo[3,2-*c*]chromene (*trans*-fused 3c)

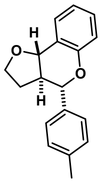

Following the procedure as detailed in section 3.2. Mp: 102-103 °C. ^{1}H NMR (400 MHz, Chloroform-*d*) δ 7.35 – 7.29 (m, 3H), 7.24 – 7.15 (m, 3H), 6.98 – 6.89 (m, 2H), 5.22 (d, *J* = 11.0 Hz, 1H), 4.61 (dt, *J* = 10.5, 1.0 Hz, 1H), 4.32 – 4.22 (m, 1H), 4.15 (td, *J* = 8.5, 7.6 Hz, 1H), 2.37 (s, 3H), 2.28 (qd, *J* = 10.9, 7.4 Hz, 1H), 1.93 – 1.78 (m, 2H). ^{13}C NMR (126 MHz, Chloroform-*d*) δ 153.8, 138.5, 137.2, 129.5, 128.7, 126.4, 125.4, 124.0, 120.4, 116.1, 82.6, 78.2, 69.3, 48.6, 27.9, 21.4. HRMS: (EI) found: 266.1294 ([M+] $C_{18}H_{18}O_2$ requires 266.1307, **Δ**= - 4.9 ppm).

(3a*RS*,4*RS*,9b*SR*)-4-(*p*-Tolyl)-2,3,3a,9b-tetrahydro-4*H*-furo[3,2-*c*]chromene (*cis*-fused 3c)

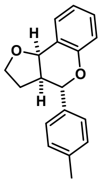

Following the general HFIP procedure as outlined in section 11.

Isolated as a white solid. Mp: 122-124 °C. ^{1}H NMR (400 MHz, Chloroform-*d*) δ 7.40 (dd, *J* = 7.6, 1.7 Hz, 1H), 7.30 – 7.25 (m, 2H), 7.20 – 7.12 (m, 3H), 6.98 – 6.85 (m, 2H), 4.57 (d, *J* = 5.1 Hz, 1H), 4.36 (d, *J* = 11.4 Hz, 1H), 3.97 (td, *J* = 8.5, 5.5 Hz, 1H), 3.77 (td, *J* = 8.8, 6.7 Hz, 1H), 2.60 (dddd, *J* = 11.2, 8.0, 5.2, 2.4 Hz, 1H), 2.31 (s, 3H), 2.07 – 1.96 (m, 1H), 1.60 (dddd, *J* = 13.2, 8.7, 6.6, 2.4 Hz, 1H). ^{13}C NMR (101 MHz, Chloroform-*d*) δ 155.4, 138.6, 136.1, 131.0, 130.0, 129.5, 128.1, 121.4 (2C), 117.3, 78.9, 74.7, 66.0, 42.1, 28.7, 21.4. HRMS: (EI) found: 266.1294 ([M+] $C_{18}H_{18}O_2$ requires 266.1307, **Δ**= - 4.9 ppm).

For the scaled up procedure: (*E*)-4-(p-tolyl)but-3-en-1-ol (162 mg, 1 mmol, 1 equiv) was weighed into an NMR tube. In a separate vial, CSA (5.8 mg, 0.025 mmol, 0.025 equiv) was weighed and dissolved in HFIP (1.3 mL, 12 mmol, 12 equiv) and 0.4 mL $CDCl_3$. To this was added salicylaldehyde (117 µL, 1.1 mmol, 1.1 equiv) and then transferred to the NMR tube. After 1 hour, ^{1}H NMR analysis suggested the reaction was done and so the reaction was quenched by addition of sodium bicarbonate and extracted with CH_2Cl_2. The organic layer was concentrated and purified over silica gel with 20% diethyl ether/hexane (232 mg, 87%). The spectra were identical to that of the small scale.

(3a*RS*,4*RS*,9b*SR*)-4-(4-Chlorophenyl)-2,3,3a,9b-tetrahydro-4*H*-furo[3,2-*c*]chromene (*cis*-fused 3f)

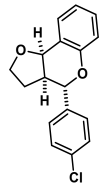

Following the general HFIP procedure as outlined in section 11. Reaction quenched after 96 h. Isolated as a white solid. Mp: 126-129 °C. ^{1}H NMR (400 MHz, Chloroform-*d*) δ 7.40 (dd, *J* = 7.6, 1.7 Hz, 1H), 7.33 (app. s, 4H), 7.21 – 7.15 (m, 1H), 6.95 (td, *J* = 7.5, 1.2 Hz, 1H), 6.87 (dd, *J* = 8.2, 1.2 Hz, 1H), 4.57 (d, *J* = 5.2 Hz, 1H), 4.37 (d, *J* = 11.3 Hz, 1H), 3.98 (td, *J* = 8.6, 5.4 Hz, 1H), 3.77 (td, *J* = 8.9, 6.7 Hz, 1H), 2.55 (dddd, *J* = 11.1, 8.0, 5.2, 2.5 Hz, 1H), 2.10 – 1.94 (m, 1H), 1.57 (dddd, *J* = 13.3, 8.4, 6.7, 2.5 Hz, 1H). ^{13}C NMR (101 MHz, Chloroform-*d*) δ 155.1, 137.7, 134.6, 131.0, 129.7, 129.5, 129.0, 121.6, 121.2, 117.2, 78.3, 74.6, 66.0, 42.3, 28.6. HRMS (EI) found 286.0769 ([M+] $C_{17}H_{15}O_2Cl$ requires 286.0761, **Δ**= 2.8 ppm).

(3a*RS*,4*SR*,9b*RS*)-4-(*p*-Tolyl)-2,3,3a,4,5,9b-hexahydrofuro[3,2-*c*]quinoline (9)

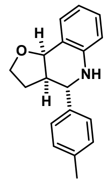

To an NMR tube was added *ortho*-aminobenzaldehye (7.7 mg, 0.06 mmol, 1 equiv) and homoallylic alcohol (14.6 mg, 0.09 mmol, 1.5 equiv). In a separate vial was weighed CSA (13.8 mg, 0.06 mmol, 1 equiv) which was dissolved in HFIP and CDCl3. This was then transferred to the NMR tube to yield a red solution. The reaction was monitored by NMR analysis and quenched after six h by addition of sat. $NaHCO_3$. The organic layer was extracted with CH_2Cl_2, dried over anhydrous Na_2SO4 and concentrated under a stream of N_2. The product was isolated by flash column chromatography 25% diethyl ether/petroleum ether (10 mg, 63% yield) as a white solid. Mp: 107-110 °C. ^{1}H NMR (400 MHz, Chloroform-*d*) δ 7.40 (dd, *J* = 7.7, 1.5 Hz, 1H), 7.35 – 7.30 (m, 2H), 7.20 (d, *J* = 7.8 Hz, 2H), 7.12 (ddd, *J* = 8.7, 7.4, 1.6 Hz, 1H), 6.79 (td, *J* = 7.5, 1.2 Hz, 1H), 6.62 (dd, *J* = 8.0, 1.1 Hz, 1H), 4.60 (d, *J* = 5.0 Hz, 1H), 4.11 (d, *J* = 3.2 Hz, 1H), 4.03 (td, *J* = 8.4, 6.1 Hz, 1H), 3.83 (ddd, *J* = 9.3, 8.5, 6.0 Hz, 1H), 3.77 (d, *J* = 11.1 Hz, 1H), 2.49 – 2.41 (m, 1H), 2.38 (s, 3H), 2.09 – 1.97 (m, 1H), 1.77 – 1.65 (m, 1H). ^{13}C NMR (101 MHz, Chloroform-*d*) δ 145.6, 138.8, 138.0, 131.4, 129.5, 129.1, 128.3, 120.2, 118.5, 114.8, 76.4, 65.4, 57.6, 43.5, 29.0, 21.3. HRMS: (ES+) found: 266.1550 ([M+H] $C_{18}H_{20}NO$ requires 266.1545, **Δ**= 1.9 ppm).

14. References

[1] A. Banerjee, H. Yamamoto, *Org. Lett.* **2017**, *19*, 4363–4366.

[2] X. Zeng, C. Miao, S. Wang, C. Xia, W. Sun, *Chem. Commun.* **2013**, *49*, 2418.

[3] Y. Wang, M. Jiang, J. T. Liu, *Adv. Synth. Catal.* **2016**, *358*, 1322–1327.

[4] J. Ciesielski, D. P. Canterbury, A. J. Frontier, *Org. Lett.* **2009**, *11*, 4374–4377.

[5] F. A. Carroll, *Perspectives on Structure and Mechanism in Organic Chemistry*, **1998**.

15. NMR spectra for new compounds

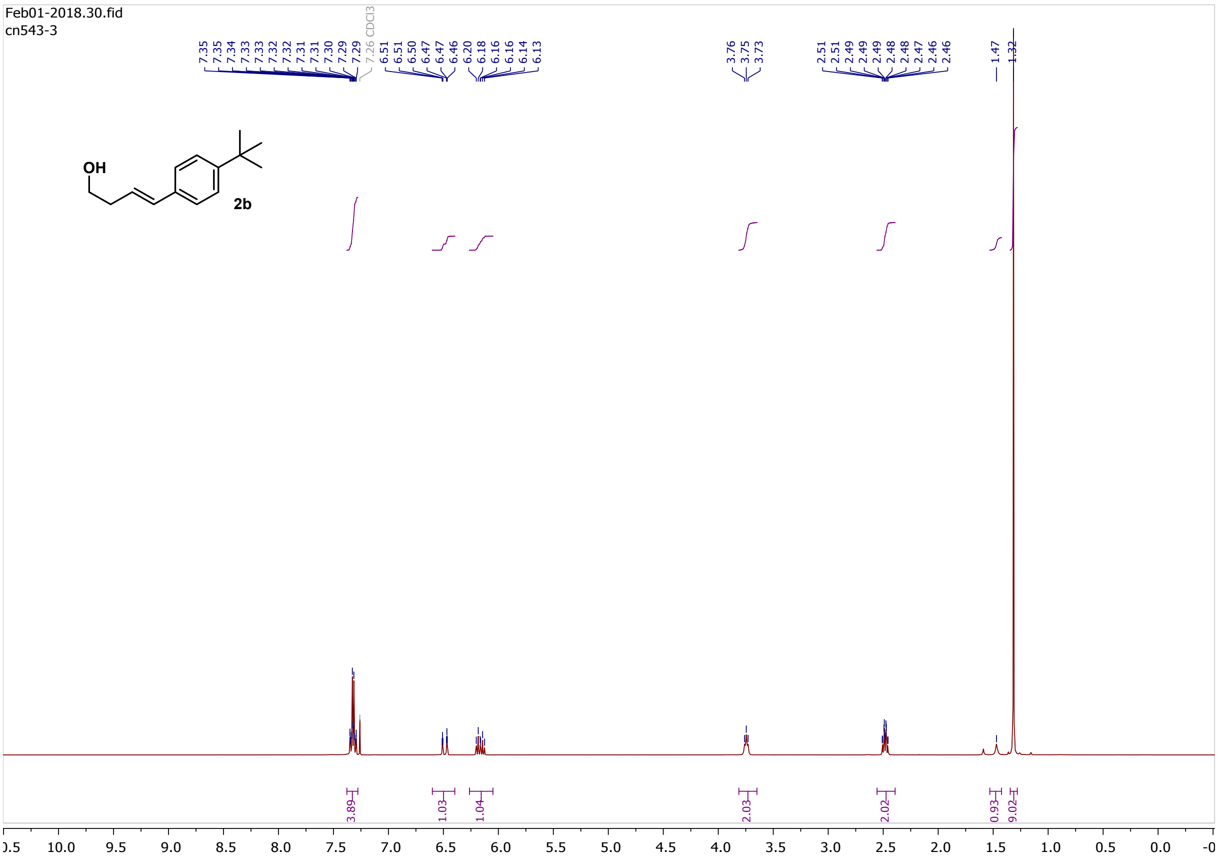

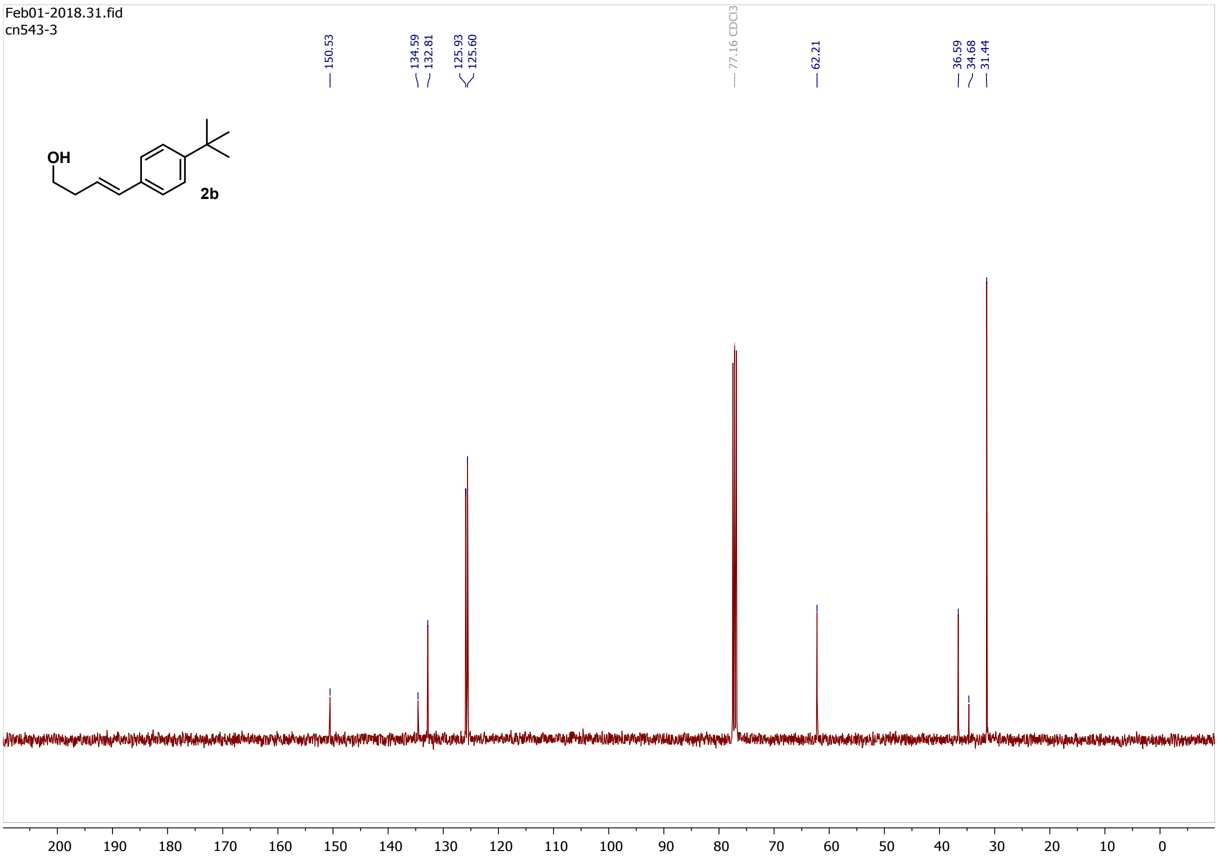

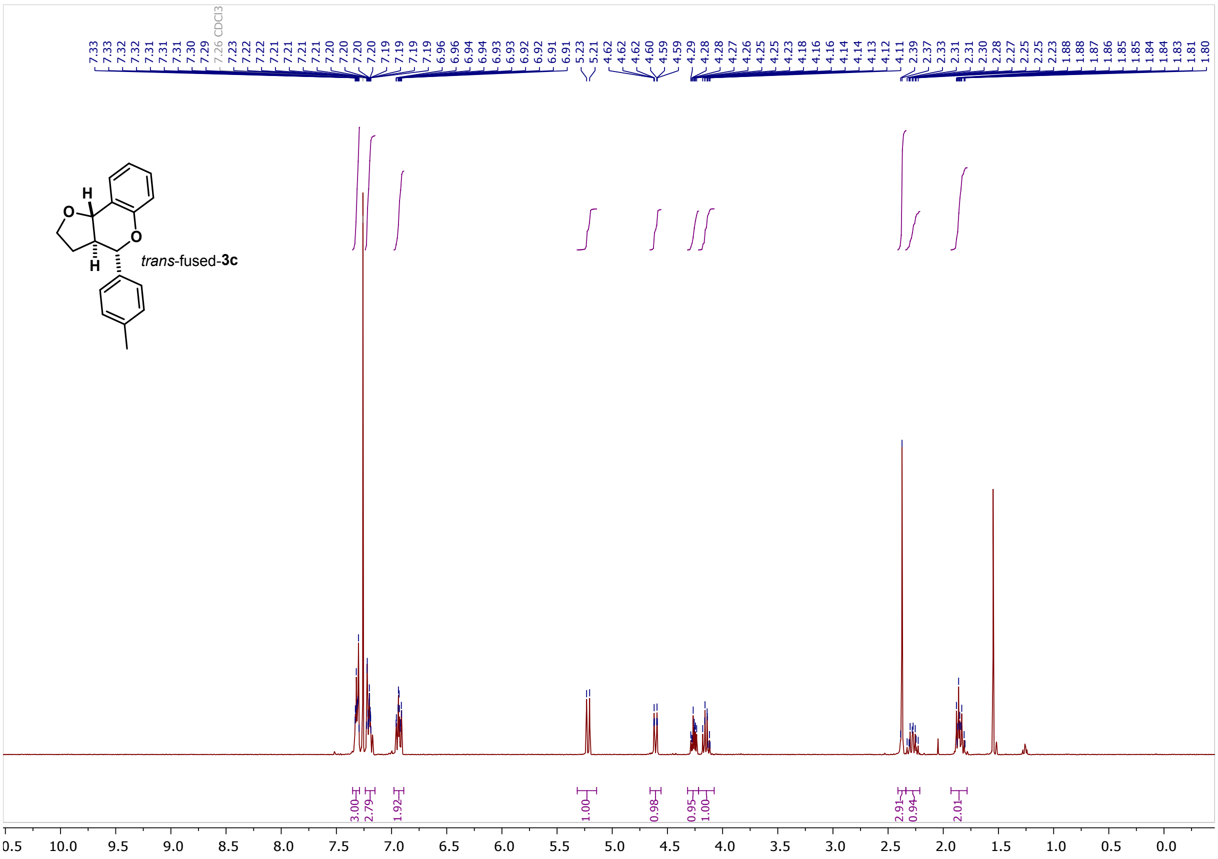
7.33
7.33
7.32
7.32
7.31
7.31
7.31
7.30
7.29
7.26 CDCl3
7.23
7.22
7.22
7.21
7.21
7.21
7.21
7.20
7.20
7.20
7.19
7.19
7.19
7.19
6.96
6.96
6.94
6.94
6.93
6.93
6.92
6.92
6.91
6.91
5.23
5.21
4.62
4.62
4.60
4.59
4.59
4.29
4.28
4.28
4.27
4.26
4.25
4.25
4.23
4.18
4.16
4.14
4.14
4.13
4.12
4.11
2.39
2.37
2.33
2.31
2.31
2.30
2.28
2.27
2.25
2.25
2.23
1.88
1.88
1.87
1.86
1.85
1.85
1.84
1.84
1.83
1.81
1.80
trans-fused-3c
3.00
2.79
1.92
1.00
0.98
0.95
1.00
2.91
0.94
2.01
0.5
10.0
9.5
9.0
8.5
8.0
7.5
7.0
6.5
6.0
5.5
5.0
4.5
4.0
3.5
3.0
2.5
2.0
1.5
1.0
0.5
0.0

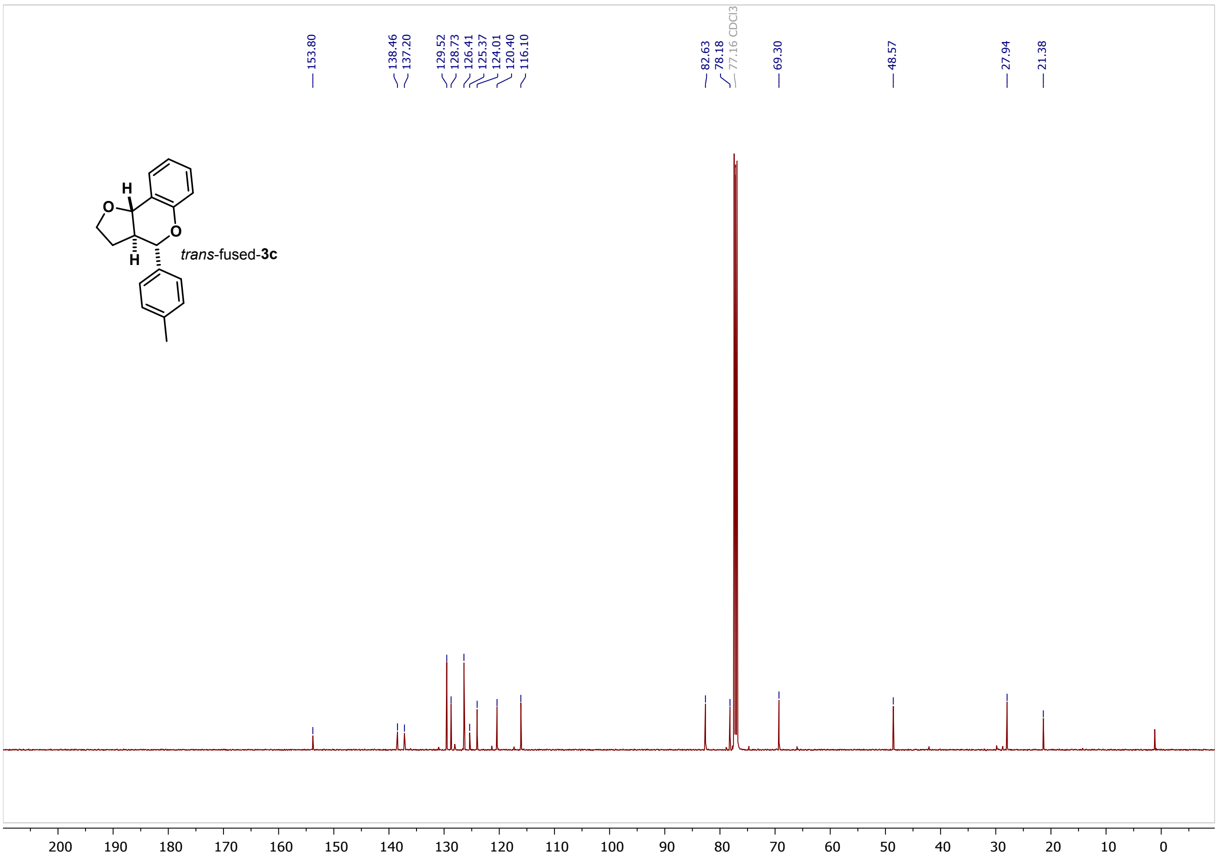
153.80
138.46
137.20
129.52
128.73
126.41
125.37
124.01
120.40
116.10
82.63
78.18
77.16 CDCl3
69.30
48.57
27.94
21.38
trans-fused-3c
200
190
180
170
160
150
140
130
120
110
100
90
80
70
60
50
40
30
20
10
0

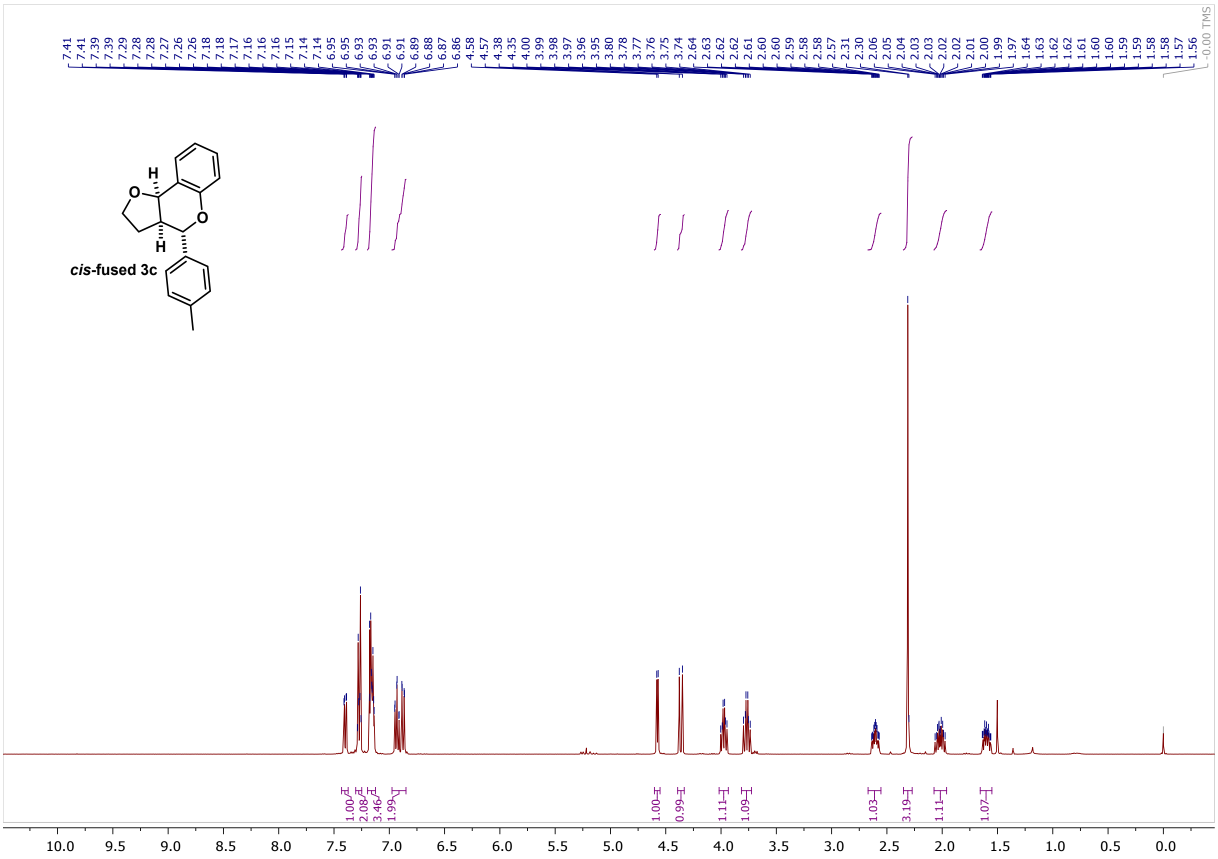

cis-fused 3c
7.41 7.41 7.39 7.29 7.28 7.27 7.26 7.26 7.18 7.17 7.16 7.16 7.15 7.14 6.95 6.95 6.93 6.93 6.91 6.91 6.89 6.88 6.87 6.86 4.58 4.57 4.38 4.35 4.00 3.99 3.98 3.97 3.96 3.95 3.80 3.78 3.77 3.76 3.75 3.74 2.64 2.63 2.62 2.62 2.61 2.60 2.60 2.59 2.58 2.58 2.57 2.31 2.30 2.06 2.05 2.04 2.03 2.03 2.02 2.02 2.01 2.00 1.99 1.97 1.64 1.63 1.62 1.61 1.60 1.60 1.59 1.59 1.58 1.58 1.57 1.56
0.00 TMS
1.00 2.08 3.46 1.99 1.00 0.99 1.11 1.09 1.03 3.19 1.11 1.07
10.0 9.5 9.0 8.5 8.0 7.5 7.0 6.5 6.0 5.5 5.0 4.5 4.0 3.5 3.0 2.5 2.0 1.5 1.0 0.5 0.0

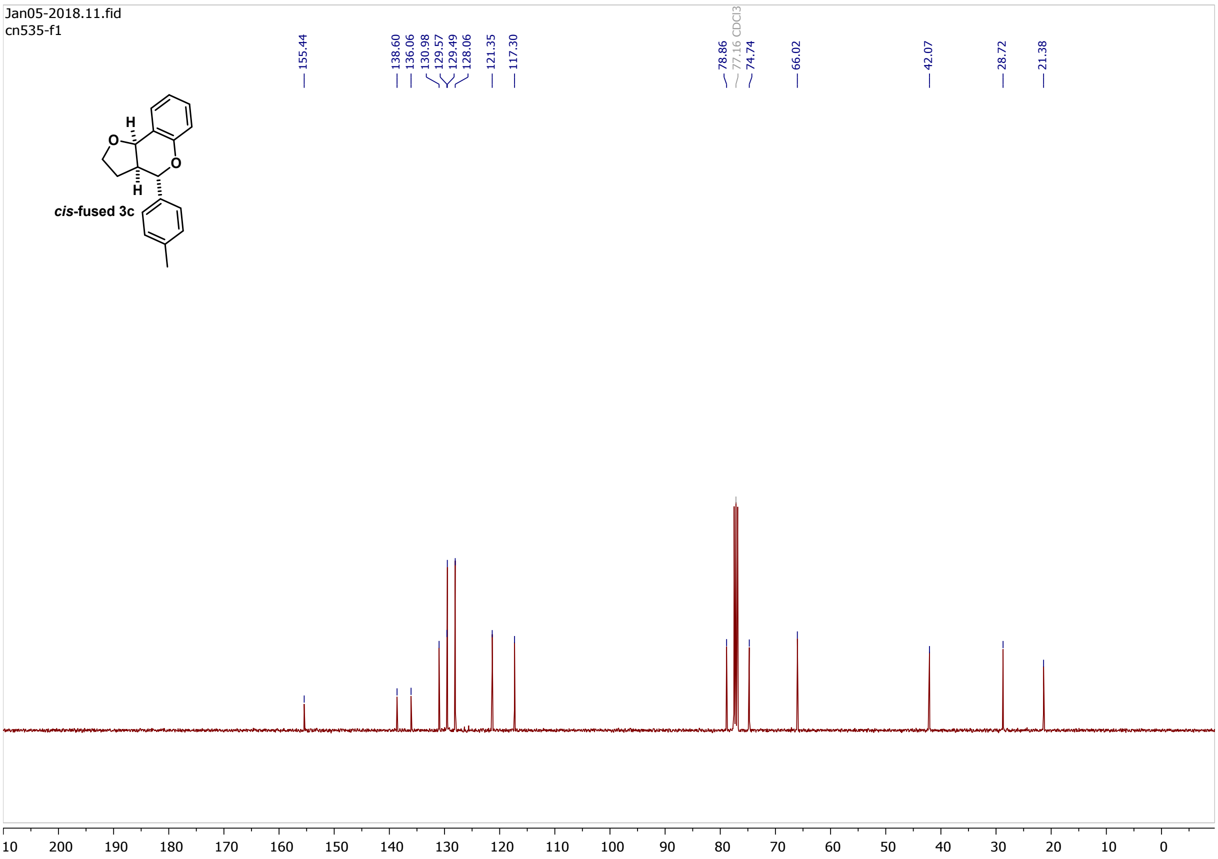

Jan05-2018.11.fid
cn535-f1
cis-fused 3c
155.44 138.60 136.06 130.98 129.57 129.49 128.06 121.35 117.30 78.86 77.16 CDCl3 74.74 66.02 42.07 28.72 21.38
210 200 190 180 170 160 150 140 130 120 110 100 90 80 70 60 50 40 30 20 10 0

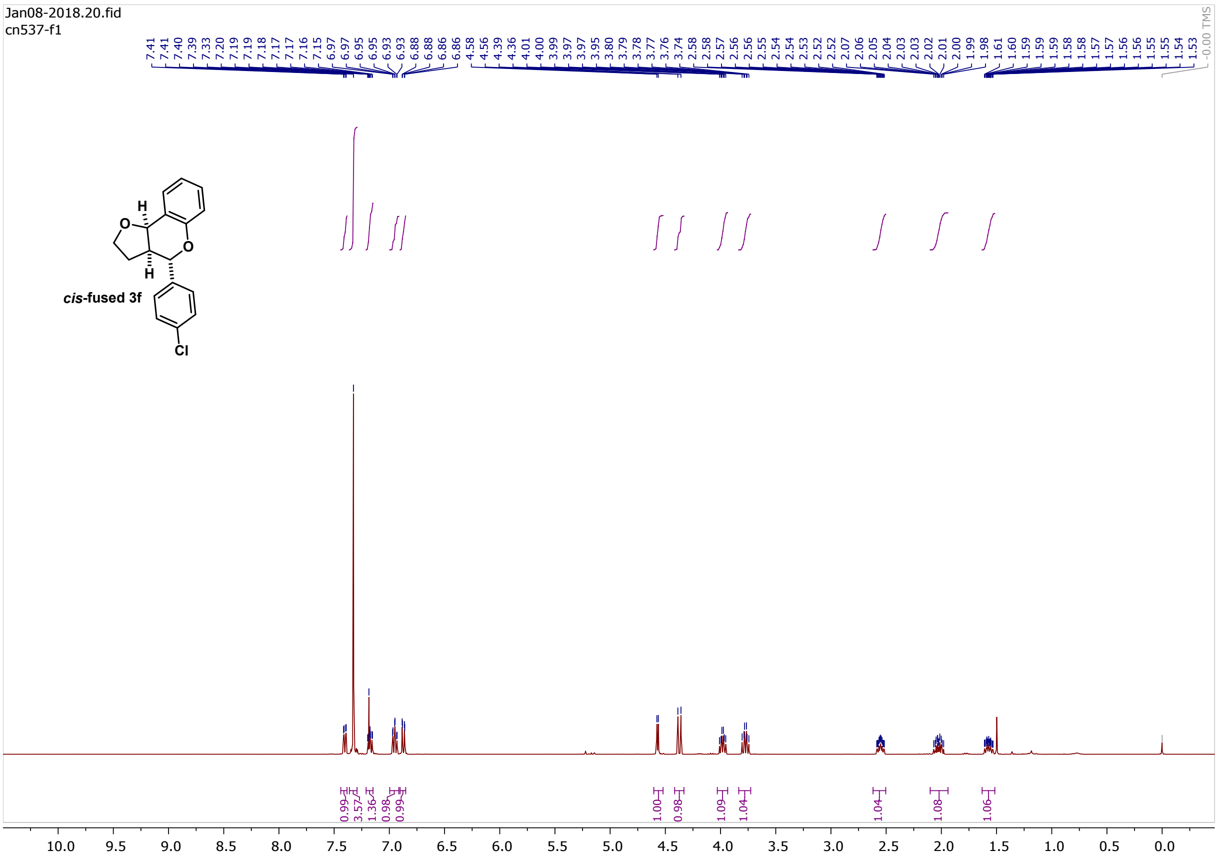
Jan08-2018.20.fid
cn537-f1
7.41 7.41 7.40 7.39 7.33 7.20 7.19 7.19 7.18 7.17 7.17 7.16 7.15 6.97 6.97 6.95 6.95 6.93 6.93 6.88 6.88 6.86 6.86 4.58 4.56 4.39 4.36 4.01 4.00 3.99 3.97 3.97 3.95 3.80 3.79 3.78 3.77 3.76 3.74 2.58 2.58 2.57 2.56 2.56 2.55 2.54 2.54 2.53 2.52 2.52 2.07 2.06 2.05 2.04 2.03 2.03 2.02 2.01 2.00 1.99 1.98 1.61 1.60 1.59 1.59 1.59 1.58 1.58 1.57 1.57 1.56 1.56 1.55 1.55 1.54 1.53
0.00 TMS
cis-fused 3f
0.99 3.57 1.36 0.98 0.99 1.00 0.98 1.09 1.04 1.04 1.08 1.06
10.0 9.5 9.0 8.5 8.0 7.5 7.0 6.5 6.0 5.5 5.0 4.5 4.0 3.5 3.0 2.5 2.0 1.5 1.0 0.5 0.0

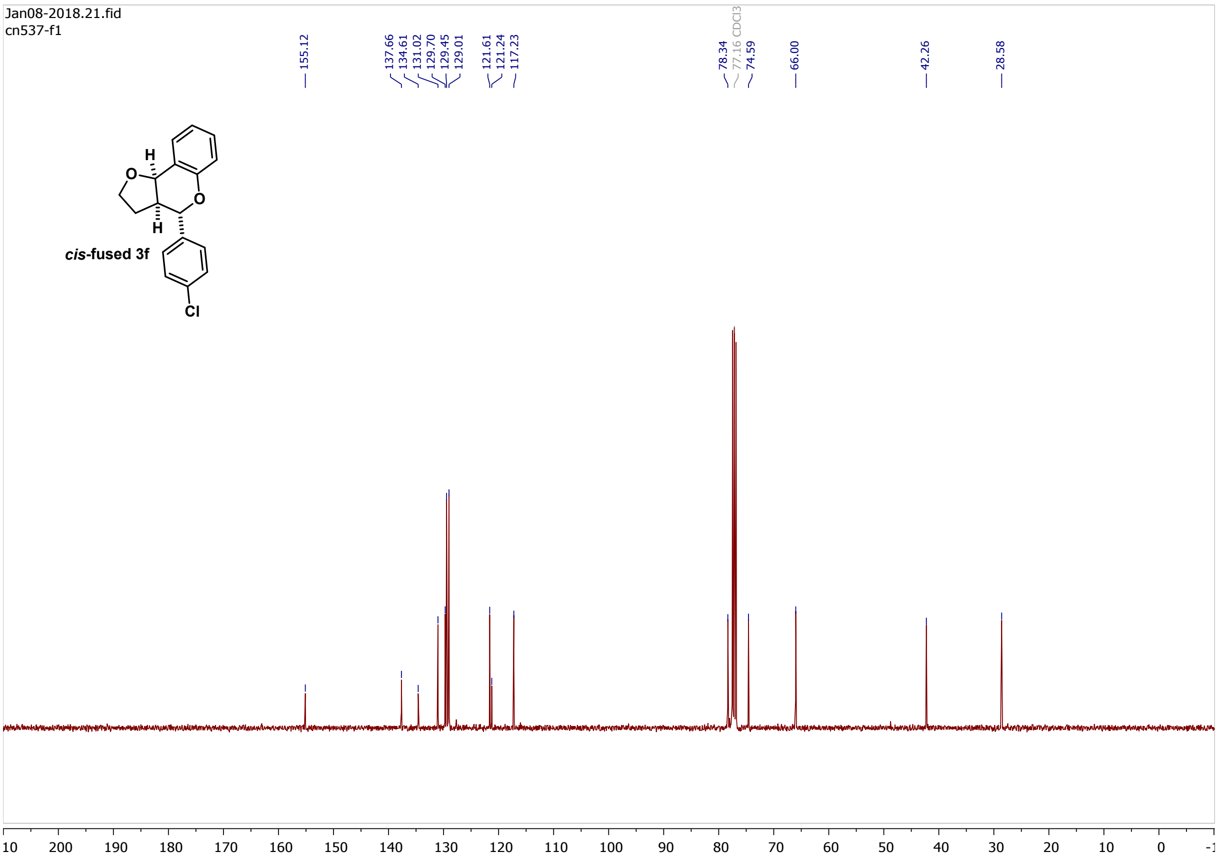
Jan08-2018.21.fid
cn537-f1
155.12
137.66 134.61 131.02 129.70 129.45 129.01
121.61 121.24 117.23
78.34 77.16 CDCl3 74.59
66.00
42.26
28.58
cis-fused 3f
210 200 190 180 170 160 150 140 130 120 110 100 90 80 70 60 50 40 30 20 10 0 -10

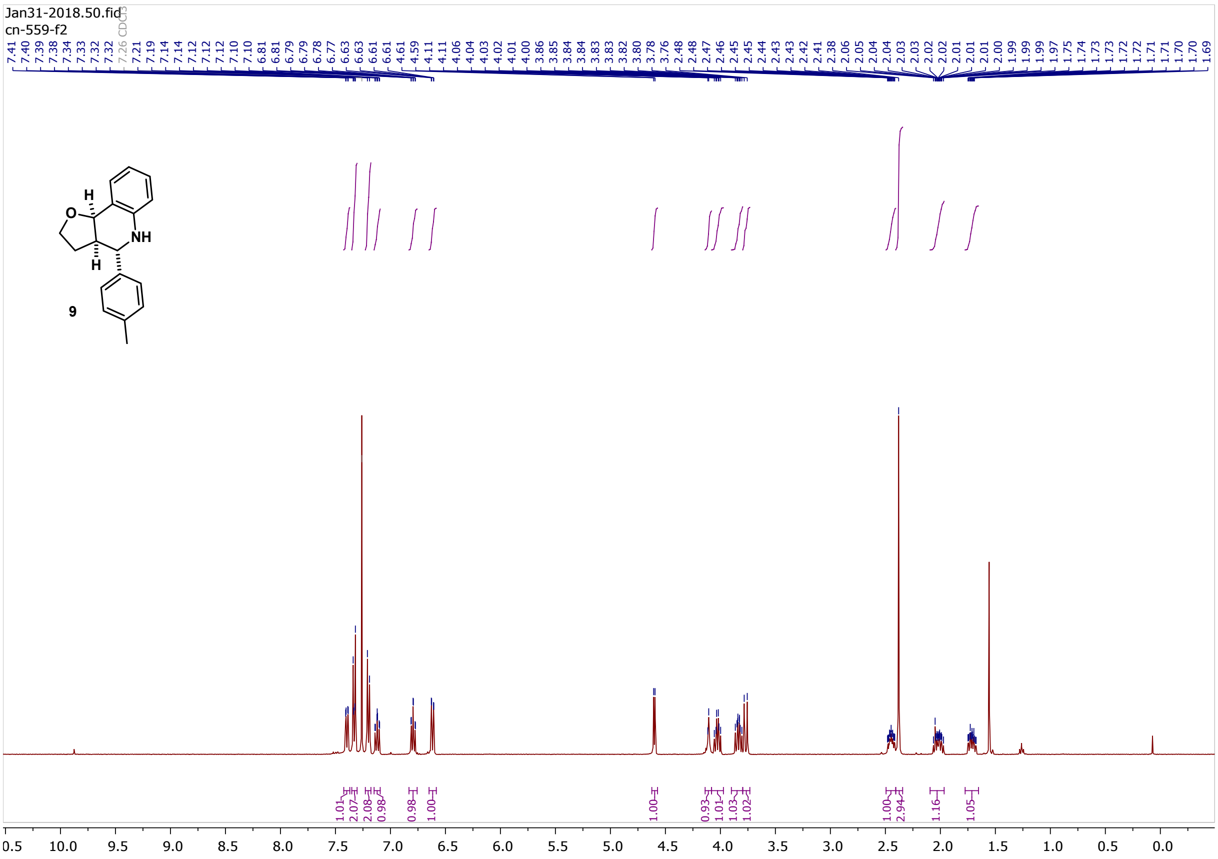
Jan31-2018.50.fid
cn-559-f2
7.41 7.40 7.39 7.38 7.34 7.33 7.32 7.32 7.26 CDCl3 7.21 7.19 7.14 7.14 7.12 7.12 7.12 7.10 6.81 6.81 6.79 6.79 6.78 6.77 6.63 6.63 6.61 6.61 4.61 4.59 4.11 4.11 4.06 4.04 4.03 4.02 4.01 4.00 3.86 3.85 3.84 3.84 3.83 3.83 3.82 3.80 3.78 3.76 2.48 2.48 2.47 2.46 2.45 2.45 2.44 2.43 2.43 2.42 2.41 2.38 2.06 2.05 2.04 2.04 2.03 2.03 2.02 2.02 2.01 2.01 2.00 1.99 1.99 1.97 1.75 1.74 1.73 1.73 1.72 1.72 1.71 1.71 1.70 1.69
H
O
H
NH
9
1.01 2.07 2.08 0.98 0.98 1.00 1.00 0.93 1.01 1.03 1.02 1.00 2.94 1.16 1.05
0.5 10.0 9.5 9.0 8.5 8.0 7.5 7.0 6.5 6.0 5.5 5.0 4.5 4.0 3.5 3.0 2.5 2.0 1.5 1.0 0.5 0.0

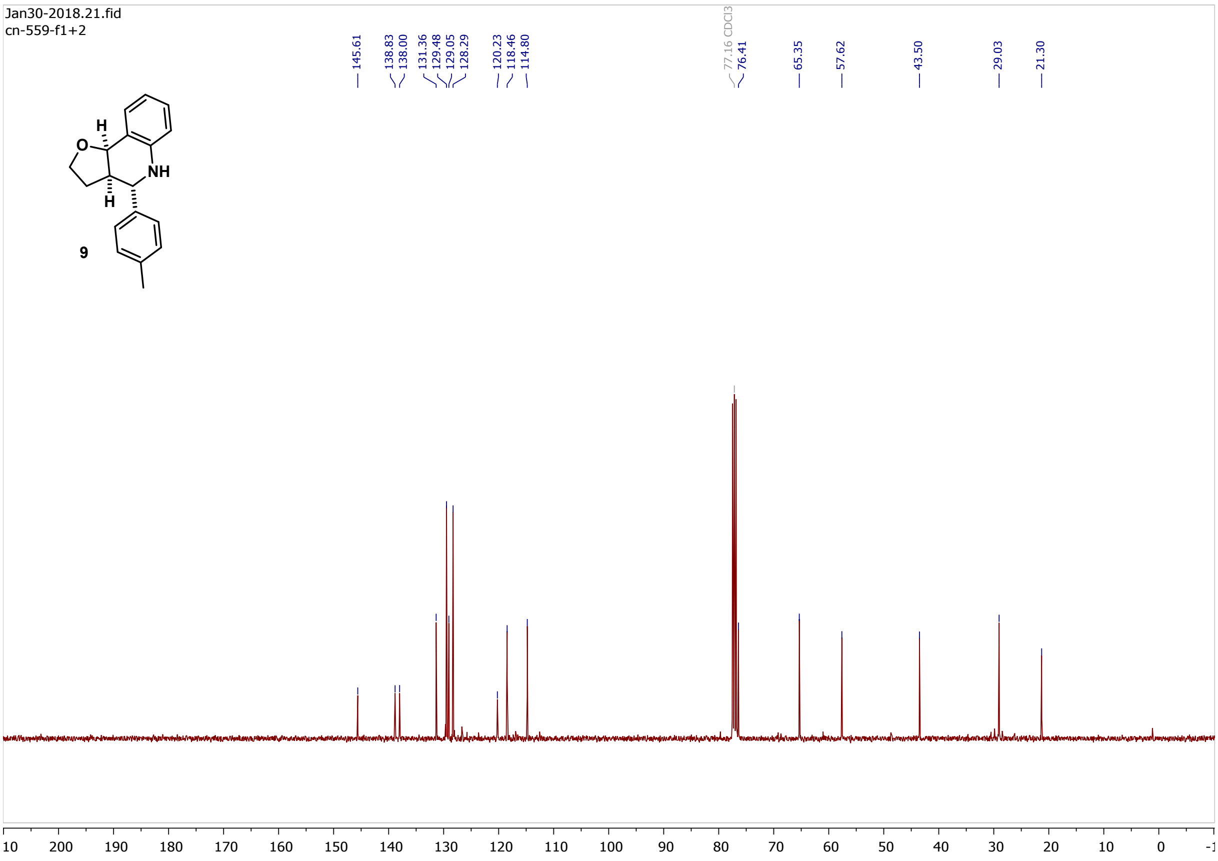
Jan30-2018.21.fid
cn-559-f1+2
145.61 138.83 138.00 131.36 129.48 129.05 128.29 120.23 118.46 114.80 77.16 CDCl3 76.41 65.35 57.62 43.50 29.03 21.30
H
O
H
NH
9
10 200 190 180 170 160 150 140 130 120 110 100 90 80 70 60 50 40 30 20 10 0 -1

16. Appendix

Appendix 1: Table of ^{1}H NMR integration data for isomerisation of *trans*-fused **3c** → *cis*-fused **3c** in MeCN

Integral (6.328275, 6.153810)	Integral (5.360598, 5.238878)	Integral (4.490298, 4.380750)	Sum	Time (min)	Homoallylic alcohol (M)	Trans (M)	Cis (M)
24.8555	8993.98	568.143	9586.979	3.5	7.77789E-05	0.028144	0.001778
44.4888	8996.52	631.52	9672.529	4.583333	0.000137985	0.027903	0.001959
63.2396	8823.72	581.785	9468.745	5.666667	0.000200363	0.027956	0.001843
116.472	8983.91	577.093	9677.475	6.75	0.000361061	0.02785	0.001789
157.7	8838.31	605.254	9601.264	7.816667	0.000492748	0.027616	0.001891
174.971	8929.12	588.826	9692.917	8.9	0.000541543	0.027636	0.001822
187.072	8931.47	579.685	9698.227	9.983333	0.000578679	0.027628	0.001793
218.363	8763.28	588.391	9570.034	11.06667	0.000684521	0.027471	0.001844
300.612	8777.59	638.332	9716.534	12.15	0.000928146	0.027101	0.001971
325.444	8628.86	610.37	9564.674	13.21667	0.001020769	0.027065	0.001914
440.677	8962.92	655.698	10059.3	17.81667	0.001314238	0.02673	0.001955
449.471	8981.82	655.861	10087.15	18.88333	0.001336763	0.026713	0.001951
492.566	8903.36	667.84	10063.77	19.96667	0.001468335	0.026541	0.001991
519.403	8886.65	675.313	10081.37	21.05	0.001545633	0.026445	0.00201
516.688	8881.82	660.213	10058.72	22.13333	0.001541015	0.02649	0.001969
534.352	8879.95	685.519	10099.82	23.2	0.001587212	0.026377	0.002036
563.61	8849.4	652.592	10065.6	24.28333	0.00167981	0.026375	0.001945
604.299	8822	651.108	10077.41	25.36667	0.001798972	0.026263	0.001938
621.839	8820.41	653.223	10095.47	26.45	0.001847875	0.026211	0.001941
668.752	8772.37	700.93	10142.05	27.53333	0.001978156	0.025949	0.002073
664.753	8742.82	632.393	10039.97	28.6	0.00198632	0.026124	0.00189
726.159	8625.08	668.167	10019.41	29.68333	0.002174258	0.025825	0.002001
701.987	8756.73	662.792	10121.51	30.76667	0.002080679	0.025955	0.001965
753.669	8600.92	673.546	10028.14	31.85	0.002254664	0.02573	0.002015
760.015	8650.04	696.926	10106.98	32.93333	0.002255911	0.025675	0.002069
821.271	8638.72	673.065	10133.06	34	0.002431461	0.025576	0.001993
824.561	8495.13	682.531	10002.22	35.08333	0.002473133	0.02548	0.002047
850.755	8624.37	682.505	10157.63	36.16667	0.002512658	0.025472	0.002016
857.587	8542.01	711.591	10111.19	37.25	0.00254447	0.025344	0.002111
894.791	8451.94	672.419	10019.15	38.33333	0.002679242	0.025307	0.002013
908.844	8492	676.875	10077.72	39.4	0.002705505	0.02528	0.002015
927.942	8428.93	667.36	10024.23	40.48333	0.002777097	0.025226	0.001997
974.2	8400.44	660.619	10035.26	41.56667	0.002912331	0.025113	0.001975
985.423	8358.4	686.236	10030.06	42.65	0.002947409	0.025	0.002053
981.153	8336.59	691.694	10009.44	43.71667	0.002940684	0.024986	0.002073
1038.61	8262.45	670.585	9971.645	44.8	0.00312469	0.024858	0.002017
1036.96	8222.61	668.957	9928.527	45.88333	0.003133274	0.024845	0.002021
1092.14	8260.76	650.574	10003.47	46.96667	0.003275282	0.024774	0.001951
1074.3	8277.42	661.744	10013.46	48.05	0.003218567	0.024799	0.001983

1120.46	8135.99	695.395	9951.845	49.11667	0.003377645	0.024526	0.002096
1169.09	8101.87	686.399	9957.359	51.33333	0.003522289	0.02441	0.002068
1340.75	7859.46	688.439	9888.649	61.36667	0.004067542	0.023844	0.002089
1531.02	7602.13	675.354	9808.504	71.4	0.004682732	0.023252	0.002066
1746.93	7465.6	692.356	9904.886	81.43333	0.005291116	0.022612	0.002097
1934.48	7257.07	693.392	9884.942	91.46667	0.00587099	0.022025	0.002104
2112.13	7013.09	714.48	9839.7	101.5	0.006439617	0.021382	0.002178
2261.8	6804.5	703.762	9770.062	111.5333	0.006945094	0.020894	0.002161
2419.08	6690.08	726.868	9836.028	121.5667	0.007378222	0.020405	0.002217
2577.59	6458.01	743.466	9779.066	131.5833	0.007907473	0.019812	0.002281
2709.85	6296.09	753.652	9759.592	141.6167	0.008329805	0.019354	0.002317
2832.17	6141.31	729.422	9702.902	151.65	0.008756669	0.018988	0.002255
2979.89	5973.5	774.161	9727.551	161.6833	0.009190052	0.018422	0.002388
3100.09	5809.33	771.185	9680.605	171.7167	0.009607116	0.018003	0.00239
3228.54	5689.72	790.931	9709.191	181.75	0.009975723	0.01758	0.002444
3329.65	5494.13	828.394	9652.174	191.7833	0.010348912	0.017076	0.002575
3487.57	5332.11	809.121	9628.801	201.8167	0.010866057	0.016613	0.002521
3552.28	5254.76	861.807	9668.847	211.8333	0.011021831	0.016304	0.002674
3617.49	5138.62	849.694	9605.804	221.8667	0.011297826	0.016048	0.002654
3732.54	4953.28	891.988	9577.808	231.9	0.011691214	0.015515	0.002794
3838.25	4841.58	863.191	9543.021	241.9333	0.012066148	0.01522	0.002714
3922.35	4725.21	909.119	9556.679	251.9667	0.012312907	0.014833	0.002854
4000.7	4666.88	941.513	9609.093	262	0.012490357	0.01457	0.002939
4125.8	4543.05	961.864	9630.714	272.0333	0.012852007	0.014152	0.002996
4159.38	4432.1	942.288	9533.768	282.0667	0.013088361	0.013947	0.002965
4199.81	4292.7	989.636	9482.146	292.0833	0.01328753	0.013581	0.003131
4295.53	4206.21	978.662	9480.402	302.1167	0.013592873	0.01331	0.003097
4329.28	4113.05	1012	9454.33	312.15	0.013737452	0.013051	0.003211
4420.97	4062.41	1054.8	9538.18	322.1833	0.013905074	0.012777	0.003318
4517.11	4082.5	1078.56	9678.17	332.2167	0.014001955	0.012655	0.003343
4542.83	3925.5	1099.32	9567.65	342.25	0.014244344	0.012309	0.003447
4576.51	3859.73	1141.66	9577.9	352.2833	0.014334593	0.012089	0.003576
4611.52	3753.21	1161.72	9526.45	362.3167	0.014522262	0.011819	0.003658
4687.11	3686.51	1190.33	9563.95	372.35	0.014702429	0.011564	0.003734
4690.63	3639.16	1189	9518.79	382.3667	0.014783276	0.011469	0.003747
4745.77	3528.98	1212.36	9487.11	392.4	0.015007004	0.011159	0.003834
4771.63	3476.26	1237.54	9485.43	402.4333	0.015091451	0.010995	0.003914
4830.46	3444.77	1264.68	9539.91	412.4667	0.015190269	0.010833	0.003977
4849.05	3399.61	1287.88	9536.54	422.5	0.015254117	0.010694	0.004051
4846.91	3297.69	1335.69	9480.29	432.5333	0.015337854	0.010435	0.004227
4938.64	3269.52	1355.42	9563.58	442.5667	0.015492023	0.010256	0.004252
4939.6	3196.01	1370.13	9505.74	452.6	0.015589318	0.010087	0.004324
4954.07	3160.32	1400.84	9515.23	462.6167	0.015619391	0.009964	0.004417
4956.02	3069.85	1401.05	9426.92	472.65	0.015771917	0.009769	0.004459

5018.83	3012.25	1429.59	9460.67	482.6833	0.015914824	0.009552	0.004533
4977.35	2974.33	1459.48	9411.16	492.7167	0.015866323	0.009481	0.004652
4975.23	2917.52	1479.99	9372.74	502.75	0.015924575	0.009338	0.004737
5038.1	2902.16	1528.34	9468.6	512.7833	0.01596255	0.009195	0.004842
4998.25	2851.02	1536.77	9386.04	522.8167	0.015975587	0.009113	0.004912
5039.08	2793.08	1572.12	9404.28	532.85	0.016074851	0.00891	0.005015
5040.72	2747.73	1599.55	9388	542.8667	0.016107968	0.008781	0.005111
5058.15	2700.55	1644.53	9403.23	552.9	0.016137487	0.008616	0.005247
5049.9	2674.19	1652.5	9376.59	562.9333	0.01615694	0.008556	0.005287
5073.42	2627.52	1664.54	9365.48	572.9667	0.016251447	0.008417	0.005332
5117.1	2610.46	1690.05	9417.61	583	0.016300633	0.008316	0.005384
5125.93	2606.05	1720.32	9452.3	593.0333	0.016268834	0.008271	0.00546
5073.66	2549.74	1731.21	9354.61	603.0667	0.016271101	0.008177	0.005552
5136.03	2488.67	1763.32	9388.02	613.1	0.016412502	0.007953	0.005635
5081.73	2492.21	1803.89	9377.83	623.1167	0.016256629	0.007973	0.005771
5060.95	2452.17	1828.53	9341.65	633.15	0.016252857	0.007875	0.005872
5150.19	2394.41	1824.83	9369.43	643.1833	0.016490405	0.007667	0.005843
5188.72	2452.89	1954.4	9596.01	653.2167	0.016221492	0.007668	0.00611
5139.5	2414.98	1927.79	9482.27	663.25	0.016260347	0.007641	0.006099
5124.45	2358.39	1959.05	9441.89	673.2833	0.016282069	0.007493	0.006225
5140.42	2332.5	1973.96	9446.88	683.3167	0.016324183	0.007407	0.006269
5158.69	2314.59	2024.87	9498.15	693.35	0.016293773	0.007311	0.006396
5182.5	2301.53	2052.85	9536.88	703.3833	0.016302501	0.00724	0.006458
5129.08	2259.61	2033	9421.69	713.4	0.01633172	0.007195	0.006473
5105.98	2225.1	2065.85	9396.93	723.4333	0.016301005	0.007104	0.006595
5139.95	2204.31	2094.5	9438.76	733.4667	0.016336733	0.007006	0.006657
5086.78	2172.95	2135.46	9395.19	743.5	0.016242716	0.006938	0.006819
5129.27	2155.75	2161.34	9446.36	753.5333	0.016289671	0.006846	0.006864
5065.61	2134.04	2160.81	9360.46	763.5667	0.016235132	0.00684	0.006925
5089.53	2099.68	2202.99	9392.2	773.6	0.01625667	0.006707	0.007037
5051.7	2065.04	2201.37	9318.11	783.6333	0.016264135	0.006648	0.007087
5055.99	2076.68	2251.33	9384	793.65	0.016163651	0.006639	0.007197
5043.4	2023.66	2265.04	9332.1	803.6833	0.016213071	0.006505	0.007281
5024.93	2010.96	2294.26	9330.15	813.7167	0.016157071	0.006466	0.007377
5041.58	2046.82	2316.01	9404.41	823.75	0.016082604	0.006529	0.007388
5020.83	2018.24	2311.73	9350.8	833.7833	0.016108237	0.006475	0.007417

Appendix 2: Table of COPASI fitted data for isomerization of *trans*-fused **3c** → *cis*-fused **3c** in MeCN

Time	Conc HA	Conc trans	Conc cis	Time	Fitted conc HA	Fitted conc trans	Fitted conc cis
3.5	7.78E-05	0.028144	0.001778	3.5	0.00026	0.027884	0.001778
4.58333	0.000138	0.027903	0.001959	3.77083	0.00028	0.027864	0.001778
5.66667	0.0002	0.027956	0.001843	4.04167	0.0003	0.027844	0.001778
6.75	0.000361	0.02785	0.001789	4.3125	0.00032	0.027824	0.001778
7.81667	0.000493	0.027616	0.001891	4.58333	0.00034	0.027804	0.001778
8.9	0.000542	0.027636	0.001822	4.85417	0.00036	0.027784	0.001778
9.98333	0.000579	0.027628	0.001793	5.125	0.00038	0.027764	0.001778
11.0667	0.000685	0.027471	0.001844	5.39583	0.0004	0.027744	0.001778
12.15	0.000928	0.027101	0.001971	5.66667	0.00042	0.027725	0.001778
13.2167	0.001021	0.027065	0.001914	5.9375	0.000439	0.027705	0.001778
17.8167	0.001314	0.02673	0.001956	6.20833	0.000459	0.027685	0.001778
18.8833	0.001337	0.026713	0.001951	6.47917	0.000479	0.027665	0.001778
19.9667	0.001468	0.026541	0.001991	6.75	0.000499	0.027645	0.001778
21.05	0.001546	0.026445	0.00201	7.01667	0.000519	0.027626	0.001778
22.1333	0.001541	0.02649	0.001969	7.28333	0.000538	0.027606	0.001778
23.2	0.001587	0.026377	0.002036	7.55	0.000558	0.027586	0.001778
24.2833	0.00168	0.026375	0.001945	7.81667	0.000577	0.027567	0.001778
25.3667	0.001799	0.026263	0.001938	8.0875	0.000597	0.027547	0.001778
26.45	0.001848	0.026211	0.001941	8.35833	0.000617	0.027527	0.001778
27.5333	0.001978	0.025949	0.002073	8.62917	0.000636	0.027508	0.001778
28.6	0.001986	0.026124	0.00189	8.9	0.000656	0.027488	0.001778
29.6833	0.002174	0.025825	0.002001	9.17083	0.000676	0.027468	0.001778
30.7667	0.002081	0.025955	0.001965	9.44167	0.000695	0.027449	0.001778
31.85	0.002255	0.02573	0.002015	9.7125	0.000715	0.027429	0.001778
32.9333	0.002256	0.025675	0.002069	9.98333	0.000735	0.027409	0.001778
34	0.002431	0.025576	0.001993	10.2542	0.000754	0.02739	0.001778
35.0833	0.002473	0.02548	0.002047	10.525	0.000774	0.02737	0.001778
36.1667	0.002513	0.025472	0.002016	10.7958	0.000794	0.02735	0.001778
37.25	0.002544	0.025344	0.002111	11.0667	0.000813	0.027331	0.001778
38.3333	0.002679	0.025307	0.002013	11.3375	0.000833	0.027311	0.001778
39.4	0.002706	0.02528	0.002015	11.6083	0.000853	0.027291	0.001778
40.4833	0.002777	0.025226	0.001997	11.8792	0.000872	0.027272	0.001778
41.5667	0.002912	0.025113	0.001975	12.15	0.000892	0.027252	0.001778
42.65	0.002947	0.025	0.002053	12.4167	0.000911	0.027233	0.001778
43.7167	0.002941	0.024986	0.002073	12.6833	0.00093	0.027214	0.001778
44.8	0.003125	0.024858	0.002017	12.95	0.000949	0.027195	0.001778
45.8833	0.003133	0.024845	0.002021	13.2167	0.000968	0.027175	0.001778
46.9667	0.003275	0.024774	0.001951	14.3667	0.001051	0.027093	0.001778
48.05	0.003219	0.024799	0.001983	15.5167	0.001133	0.02701	0.001778
49.1167	0.003378	0.024526	0.002096	16.6667	0.001216	0.026928	0.001778
51.3333	0.003522	0.02441	0.002068	17.8167	0.001297	0.026846	0.001778

61.3667	0.004068	0.023844	0.002089	18.0833	0.001316	0.026827	0.001778
71.4	0.004683	0.023252	0.002066	18.35	0.001335	0.026808	0.001778
81.4333	0.005291	0.022612	0.002097	18.6167	0.001354	0.02679	0.001778
91.4667	0.005871	0.022025	0.002104	18.8833	0.001373	0.026771	0.001778
101.5	0.00644	0.021382	0.002178	19.1542	0.001392	0.026751	0.001778
111.533	0.006945	0.020894	0.002161	19.425	0.001411	0.026732	0.001779
121.567	0.007378	0.020405	0.002217	19.6958	0.00143	0.026713	0.001779
131.583	0.007907	0.019812	0.002281	19.9667	0.00145	0.026694	0.001779
141.617	0.00833	0.019354	0.002317	20.2375	0.001469	0.026675	0.001779
151.65	0.008757	0.018988	0.002255	20.5083	0.001488	0.026656	0.001779
161.683	0.00919	0.018422	0.002388	20.7792	0.001507	0.026637	0.001779
171.717	0.009607	0.018003	0.00239	21.05	0.001526	0.026618	0.001779
181.75	0.009976	0.01758	0.002444	21.3208	0.001545	0.026598	0.001779
191.783	0.010349	0.017076	0.002575	21.5917	0.001564	0.026579	0.001779
201.817	0.010866	0.016613	0.002521	21.8625	0.001583	0.02656	0.001779
211.833	0.011022	0.016304	0.002674	22.1333	0.001602	0.026541	0.001779
221.867	0.011298	0.016049	0.002654	22.4	0.001621	0.026523	0.001779
231.9	0.011691	0.015515	0.002794	22.6667	0.001639	0.026504	0.001779
241.933	0.012066	0.01522	0.002714	22.9333	0.001658	0.026485	0.001779
251.967	0.012313	0.014833	0.002854	23.2	0.001677	0.026466	0.001779
262	0.01249	0.01457	0.002939	23.4708	0.001696	0.026448	0.001779
272.033	0.012852	0.014152	0.002996	23.7417	0.001715	0.026429	0.001779
282.067	0.013088	0.013947	0.002965	24.0125	0.001733	0.02641	0.001779
292.083	0.013288	0.013581	0.003131	24.2833	0.001752	0.026391	0.001779
302.117	0.013593	0.01331	0.003097	24.5542	0.001771	0.026372	0.001779
312.15	0.013738	0.013051	0.003211	24.825	0.00179	0.026353	0.001779
322.183	0.013905	0.012777	0.003318	25.0958	0.001809	0.026334	0.001779
332.217	0.014002	0.012655	0.003343	25.3667	0.001828	0.026315	0.001779
342.25	0.014244	0.012309	0.003447	25.6375	0.001847	0.026296	0.001779
352.283	0.014335	0.01209	0.003576	25.9083	0.001865	0.026278	0.001779
362.317	0.014522	0.011819	0.003658	26.1792	0.001884	0.026259	0.001779
372.35	0.014702	0.011564	0.003734	26.45	0.001903	0.02624	0.001779
382.367	0.014783	0.011469	0.003747	26.7208	0.001922	0.026221	0.001779
392.4	0.015007	0.011159	0.003834	26.9917	0.00194	0.026202	0.001779
402.433	0.015092	0.010995	0.003914	27.2625	0.001959	0.026184	0.001779
412.467	0.01519	0.010833	0.003977	27.5333	0.001978	0.026165	0.001779
422.5	0.015254	0.010695	0.004051	27.8	0.001996	0.026146	0.001779
432.533	0.015338	0.010435	0.004227	28.0667	0.002015	0.026128	0.001779
442.567	0.015492	0.010256	0.004252	28.3333	0.002033	0.02611	0.00178
452.6	0.015589	0.010087	0.004324	28.6	0.002051	0.026091	0.00178
462.617	0.015619	0.009964	0.004417	28.8708	0.00207	0.026072	0.00178
472.65	0.015772	0.009769	0.004459	29.1417	0.002089	0.026054	0.00178
482.683	0.015915	0.009552	0.004533	29.4125	0.002107	0.026035	0.00178
492.717	0.015866	0.009481	0.004652	29.6833	0.002126	0.026017	0.00178

502.75	0.015925	0.009338	0.004737	29.9542	0.002144	0.025998	0.00178
512.783	0.015963	0.009195	0.004842	30.225	0.002163	0.025979	0.00178
522.817	0.015976	0.009113	0.004912	30.4958	0.002181	0.025961	0.00178
532.85	0.016075	0.00891	0.005015	30.7667	0.0022	0.025942	0.00178
542.867	0.016108	0.008781	0.005111	31.0375	0.002218	0.025924	0.00178
552.9	0.016138	0.008616	0.005247	31.3083	0.002237	0.025905	0.00178
562.933	0.016157	0.008556	0.005287	31.5792	0.002255	0.025887	0.00178
572.967	0.016251	0.008417	0.005332	31.85	0.002274	0.025868	0.00178
583	0.016301	0.008316	0.005384	32.1208	0.002292	0.025849	0.00178
593.033	0.016269	0.008271	0.00546	32.3917	0.002311	0.025831	0.00178
603.067	0.016271	0.008177	0.005552	32.6625	0.002329	0.025813	0.00178
613.1	0.016413	0.007953	0.005635	32.9333	0.002348	0.025794	0.00178
623.117	0.016257	0.007973	0.005771	33.2	0.002366	0.025776	0.00178
633.15	0.016253	0.007875	0.005872	33.4667	0.002384	0.025758	0.00178
643.183	0.01649	0.007667	0.005843	33.7333	0.002402	0.02574	0.001781
653.217	0.016222	0.007668	0.00611	34	0.00242	0.025721	0.001781
663.25	0.01626	0.007641	0.006099	34.2708	0.002438	0.025703	0.001781
673.283	0.016282	0.007493	0.006225	34.5417	0.002457	0.025685	0.001781
683.317	0.016324	0.007407	0.006269	34.8125	0.002475	0.025666	0.001781
693.35	0.016294	0.007311	0.006396	35.0833	0.002493	0.025648	0.001781
703.383	0.016303	0.00724	0.006458	35.3542	0.002511	0.02563	0.001781
713.4	0.016332	0.007195	0.006473	35.625	0.00253	0.025611	0.001781
723.433	0.016301	0.007104	0.006595	35.8958	0.002548	0.025593	0.001781
733.467	0.016337	0.007006	0.006657	36.1667	0.002566	0.025575	0.001781
743.5	0.016243	0.006939	0.006819	36.4375	0.002584	0.025556	0.001781
753.533	0.01629	0.006846	0.006864	36.7083	0.002603	0.025538	0.001781
763.567	0.016235	0.00684	0.006925	36.9792	0.002621	0.02552	0.001781
773.6	0.016257	0.006707	0.007037	37.25	0.002639	0.025502	0.001781
783.633	0.016264	0.006648	0.007087	37.5208	0.002657	0.025484	0.001781
793.65	0.016164	0.006639	0.007197	37.7917	0.002675	0.025465	0.001782
803.683	0.016213	0.006505	0.007281	38.0625	0.002693	0.025447	0.001782
813.717	0.016157	0.006466	0.007377	38.3333	0.002711	0.025429	0.001782
823.75	0.016083	0.006529	0.007388	38.6	0.002729	0.025411	0.001782
833.783	0.016108	0.006475	0.007417	38.8667	0.002747	0.025393	0.001782
				39.1333	0.002765	0.025375	0.001782
				39.4	0.002783	0.025357	0.001782
				39.6708	0.002801	0.025339	0.001782
				39.9417	0.002819	0.025321	0.001782
				40.2125	0.002837	0.025303	0.001782
				40.4833	0.002855	0.025285	0.001782
				40.7542	0.002873	0.025267	0.001782
				41.025	0.002891	0.025249	0.001783
				41.2958	0.002908	0.025231	0.001783
				41.5667	0.002926	0.025213	0.001783

				41.8375	0.002944	0.025195	0.001783
				42.1083	0.002962	0.025177	0.001783
				42.3792	0.00298	0.025159	0.001783
				42.65	0.002998	0.025141	0.001783
				42.9167	0.003015	0.025123	0.001783
				43.1833	0.003033	0.025106	0.001783
				43.45	0.003051	0.025088	0.001783
				43.7167	0.003068	0.025071	0.001783
				43.9875	0.003086	0.025053	0.001784
				44.2583	0.003104	0.025035	0.001784
				44.5292	0.003121	0.025017	0.001784
				44.8	0.003139	0.024999	0.001784
				45.0708	0.003157	0.024981	0.001784
				45.3417	0.003175	0.024963	0.001784
				45.6125	0.003192	0.024946	0.001784
				45.8833	0.00321	0.024928	0.001784
				46.1542	0.003228	0.02491	0.001784
				46.425	0.003245	0.024892	0.001784
				46.6958	0.003263	0.024875	0.001785
				46.9667	0.003281	0.024857	0.001785
				47.2375	0.003298	0.024839	0.001785
				47.5083	0.003316	0.024821	0.001785
				47.7792	0.003333	0.024804	0.001785
				48.05	0.003351	0.024786	0.001785
				48.3167	0.003368	0.024769	0.001785
				48.5833	0.003385	0.024751	0.001785
				48.85	0.003403	0.024734	0.001786
				49.1167	0.00342	0.024717	0.001786
				49.6708	0.003456	0.024681	0.001786
				50.225	0.003491	0.024645	0.001786
				50.7792	0.003527	0.024609	0.001786
				51.3333	0.003563	0.024573	0.001787
				53.8417	0.003723	0.024411	0.001788
				56.35	0.003882	0.024251	0.001789
				58.8583	0.004039	0.024092	0.001791
				61.3667	0.004195	0.023934	0.001792
				63.875	0.00435	0.023777	0.001794
				66.3833	0.004504	0.023622	0.001796
				68.8917	0.004656	0.023468	0.001798
				71.4	0.004807	0.023314	0.0018
				73.9083	0.004957	0.023162	0.001803
				76.4167	0.005106	0.023011	0.001805
				78.925	0.005253	0.022862	0.001808
				81.4333	0.005399	0.022713	0.00181

				83.9417	0.005543	0.022566	0.001813
				86.45	0.005686	0.022419	0.001816
				88.9583	0.005828	0.022274	0.001819
				91.4667	0.005969	0.02213	0.001823
				93.975	0.006108	0.021987	0.001826
				96.4833	0.006247	0.021846	0.00183
				98.9917	0.006383	0.021705	0.001834
				101.5	0.006519	0.021565	0.001838
				104.008	0.006653	0.021427	0.001842
				106.517	0.006786	0.021289	0.001846
				109.025	0.006918	0.021153	0.001851
				111.533	0.007048	0.021018	0.001856
				114.042	0.007178	0.020884	0.001861
				116.55	0.007306	0.020751	0.001866
				119.058	0.007432	0.020619	0.001871
				121.567	0.007558	0.020488	0.001876
				124.071	0.007682	0.020359	0.001882
				126.575	0.007804	0.02023	0.001888
				129.079	0.007926	0.020103	0.001894
				131.583	0.008046	0.019976	0.0019
				134.092	0.008166	0.01985	0.001906
				136.6	0.008284	0.019726	0.001912
				139.108	0.008401	0.019602	0.001919
				141.617	0.008516	0.01948	0.001926
				144.125	0.008631	0.019358	0.001933
				146.633	0.008744	0.019238	0.00194
				149.142	0.008856	0.019118	0.001948
				151.65	0.008967	0.019	0.001956
				154.158	0.009077	0.018882	0.001963
				156.667	0.009185	0.018765	0.001971
				159.175	0.009293	0.01865	0.00198
				161.683	0.009399	0.018535	0.001988
				164.192	0.009504	0.018422	0.001997
				166.7	0.009608	0.018309	0.002005
				169.208	0.00971	0.018197	0.002014
				171.717	0.009812	0.018086	0.002024
				174.225	0.009913	0.017976	0.002033
				176.733	0.010012	0.017868	0.002043
				179.242	0.01011	0.01776	0.002052
				181.75	0.010207	0.017652	0.002062
				184.258	0.010303	0.017546	0.002072
				186.767	0.010398	0.017441	0.002083
				189.275	0.010492	0.017337	0.002093
				191.783	0.010585	0.017233	0.002104

				194.292	0.010677	0.01713	0.002115
				196.8	0.010767	0.017029	0.002126
				199.308	0.010857	0.016928	0.002137
				201.817	0.010946	0.016828	0.002149
				204.321	0.011033	0.016729	0.00216
				206.825	0.011119	0.016631	0.002172
				209.329	0.011205	0.016534	0.002184
				211.833	0.011289	0.016437	0.002196
				214.342	0.011372	0.016341	0.002209
				216.85	0.011455	0.016246	0.002221
				219.358	0.011536	0.016152	0.002234
				221.867	0.011616	0.016059	0.002247
				224.375	0.011696	0.015967	0.00226
				226.883	0.011774	0.015875	0.002273
				229.392	0.011852	0.015784	0.002287
				231.9	0.011928	0.015694	0.0023
				234.408	0.012004	0.015605	0.002314
				236.917	0.012078	0.015516	0.002328
				239.425	0.012152	0.015428	0.002342
				241.933	0.012224	0.015341	0.002356
				244.442	0.012296	0.015255	0.002371
				246.95	0.012367	0.01517	0.002386
				249.458	0.012437	0.015085	0.0024
				251.967	0.012506	0.015001	0.002415
				254.475	0.012574	0.014918	0.00243
				256.983	0.012641	0.014835	0.002446
				259.492	0.012708	0.014753	0.002461
				262	0.012773	0.014672	0.002477
				264.508	0.012838	0.014592	0.002493
				267.017	0.012902	0.014512	0.002509
				269.525	0.012964	0.014433	0.002525
				272.033	0.013027	0.014355	0.002541
				274.542	0.013088	0.014277	0.002557
				277.05	0.013148	0.0142	0.002574
				279.558	0.013208	0.014124	0.002591
				282.067	0.013266	0.014048	0.002608
				284.571	0.013324	0.013973	0.002625
				287.075	0.013381	0.013899	0.002642
				289.579	0.013438	0.013825	0.002659
				292.083	0.013493	0.013753	0.002677
				294.592	0.013548	0.01368	0.002694
				297.1	0.013602	0.013608	0.002712
				299.608	0.013655	0.013537	0.00273
				302.117	0.013707	0.013467	0.002748

				304.625	0.013759	0.013397	0.002766
				307.133	0.01381	0.013328	0.002784
				309.642	0.01386	0.013259	0.002803
				312.15	0.01391	0.013191	0.002821
				314.658	0.013959	0.013123	0.00284
				317.167	0.014007	0.013056	0.002859
				319.675	0.014054	0.01299	0.002878
				322.183	0.014101	0.012924	0.002897
				324.692	0.014147	0.012859	0.002916
				327.2	0.014192	0.012794	0.002935
				329.708	0.014237	0.01273	0.002955
				332.217	0.014281	0.012667	0.002975
				334.725	0.014324	0.012604	0.002994
				337.233	0.014366	0.012542	0.003014
				339.742	0.014408	0.01248	0.003034
				342.25	0.01445	0.012418	0.003054
				344.758	0.01449	0.012357	0.003074
				347.267	0.01453	0.012297	0.003095
				349.775	0.01457	0.012237	0.003115
				352.283	0.014609	0.012178	0.003135
				354.792	0.014647	0.012119	0.003156
				357.3	0.014684	0.012061	0.003177
				359.808	0.014721	0.012003	0.003198
				362.317	0.014757	0.011946	0.003219
				364.825	0.014793	0.011889	0.00324
				367.333	0.014828	0.011833	0.003261
				369.842	0.014863	0.011777	0.003282
				372.35	0.014897	0.011722	0.003303
				374.854	0.01493	0.011667	0.003325
				377.358	0.014963	0.011613	0.003346
				379.863	0.014995	0.011559	0.003368
				382.367	0.015027	0.011505	0.003389
				384.875	0.015058	0.011452	0.003411
				387.383	0.015089	0.0114	0.003433
				389.892	0.015119	0.011348	0.003455
				392.4	0.015149	0.011296	0.003477
				394.908	0.015178	0.011245	0.003499
				397.417	0.015207	0.011194	0.003522
				399.925	0.015235	0.011143	0.003544
				402.433	0.015262	0.011093	0.003566
				404.942	0.01529	0.011044	0.003589
				407.45	0.015316	0.010995	0.003611
				409.958	0.015342	0.010946	0.003634
				412.467	0.015368	0.010898	0.003657

				414.975	0.015393	0.01085	0.003679
				417.483	0.015418	0.010802	0.003702
				419.992	0.015442	0.010755	0.003725
				422.5	0.015466	0.010708	0.003748
				425.008	0.015489	0.010662	0.003771
				427.517	0.015512	0.010616	0.003794
				430.025	0.015534	0.01057	0.003818
				432.533	0.015556	0.010525	0.003841
				435.042	0.015578	0.01048	0.003864
				437.55	0.015599	0.010435	0.003888
				440.058	0.01562	0.010391	0.003911
				442.567	0.01564	0.010347	0.003935
				445.075	0.01566	0.010304	0.003958
				447.583	0.015679	0.010261	0.003982
				450.092	0.015698	0.010218	0.004006
				452.6	0.015717	0.010176	0.004029
				455.104	0.015735	0.010134	0.004053
				457.608	0.015753	0.010092	0.004077
				460.113	0.01577	0.010051	0.004101
				462.617	0.015788	0.01001	0.004125
				465.125	0.015804	0.009969	0.004149
				467.633	0.015821	0.009928	0.004173
				470.142	0.015837	0.009888	0.004197
				472.65	0.015852	0.009848	0.004221
				475.158	0.015867	0.009809	0.004246
				477.667	0.015882	0.00977	0.00427
				480.175	0.015897	0.009731	0.004294
				482.683	0.015911	0.009692	0.004319
				485.192	0.015925	0.009654	0.004343
				487.7	0.015938	0.009616	0.004367
				490.208	0.015951	0.009579	0.004392
				492.717	0.015964	0.009541	0.004417
				495.225	0.015977	0.009504	0.004441
				497.733	0.015989	0.009467	0.004466
				500.242	0.016001	0.009431	0.00449
				502.75	0.016012	0.009395	0.004515
				505.258	0.016024	0.009359	0.00454
				507.767	0.016035	0.009323	0.004565
				510.275	0.016045	0.009288	0.004589
				512.783	0.016055	0.009252	0.004614
				515.292	0.016066	0.009218	0.004639
				517.8	0.016075	0.009183	0.004664
				520.308	0.016085	0.009149	0.004689
				522.817	0.016094	0.009115	0.004714

				525.325	0.016103	0.009081	0.004739
				527.833	0.016111	0.009047	0.004764
				530.342	0.01612	0.009014	0.004789
				532.85	0.016128	0.008981	0.004814
				535.354	0.016135	0.008948	0.004839
				537.858	0.016143	0.008915	0.004864
				540.363	0.01615	0.008883	0.004889
				542.867	0.016157	0.008851	0.004914
				545.375	0.016164	0.008819	0.004939
				547.883	0.01617	0.008788	0.004964
				550.392	0.016176	0.008756	0.00499
				552.9	0.016182	0.008725	0.005015
				555.408	0.016188	0.008694	0.00504
				557.917	0.016194	0.008663	0.005065
				560.425	0.016199	0.008633	0.005091
				562.933	0.016204	0.008602	0.005116
				565.442	0.016209	0.008572	0.005141
				567.95	0.016213	0.008542	0.005166
				570.458	0.016217	0.008513	0.005192
				572.967	0.016222	0.008483	0.005217
				575.475	0.016225	0.008454	0.005242
				577.983	0.016229	0.008425	0.005268
				580.492	0.016233	0.008396	0.005293
				583	0.016236	0.008368	0.005319
				585.508	0.016239	0.008339	0.005344
				588.017	0.016242	0.008311	0.005369
				590.525	0.016244	0.008283	0.005395
				593.033	0.016247	0.008255	0.00542
				595.542	0.016249	0.008228	0.005446
				598.05	0.016251	0.0082	0.005471
				600.558	0.016253	0.008173	0.005497
				603.067	0.016254	0.008146	0.005522
				605.575	0.016256	0.008119	0.005547
				608.083	0.016257	0.008092	0.005573
				610.592	0.016258	0.008066	0.005598
				613.1	0.016259	0.008039	0.005624
				615.604	0.01626	0.008013	0.005649
				618.108	0.01626	0.007987	0.005675
				620.613	0.01626	0.007961	0.0057
				623.117	0.016261	0.007936	0.005726
				625.625	0.016261	0.00791	0.005751
				628.133	0.01626	0.007885	0.005777
				630.642	0.01626	0.00786	0.005802
				633.15	0.01626	0.007835	0.005828

				635.658	0.016259	0.00781	0.005853
				638.167	0.016258	0.007785	0.005878
				640.675	0.016257	0.007761	0.005904
				643.183	0.016256	0.007737	0.005929
				645.692	0.016255	0.007712	0.005955
				648.2	0.016253	0.007688	0.00598
				650.708	0.016252	0.007665	0.006006
				653.217	0.01625	0.007641	0.006031
				655.725	0.016248	0.007617	0.006057
				658.233	0.016246	0.007594	0.006082
				660.742	0.016244	0.007571	0.006107
				663.25	0.016242	0.007547	0.006133
				665.758	0.016239	0.007524	0.006158
				668.267	0.016237	0.007502	0.006184
				670.775	0.016234	0.007479	0.006209
				673.283	0.016231	0.007456	0.006234
				675.792	0.016228	0.007434	0.00626
				678.3	0.016225	0.007412	0.006285
				680.808	0.016222	0.00739	0.006311
				683.317	0.016219	0.007368	0.006336
				685.825	0.016215	0.007346	0.006361
				688.333	0.016211	0.007324	0.006387
				690.842	0.016208	0.007302	0.006412
				693.35	0.016204	0.007281	0.006437
				695.858	0.0162	0.00726	0.006463
				698.367	0.016196	0.007238	0.006488
				700.875	0.016192	0.007217	0.006513
				703.383	0.016187	0.007196	0.006538
				705.887	0.016183	0.007176	0.006563
				708.392	0.016178	0.007155	0.006589
				710.896	0.016174	0.007134	0.006614
				713.4	0.016169	0.007114	0.006639
				715.908	0.016164	0.007094	0.006664
				718.417	0.016159	0.007073	0.006689
				720.925	0.016154	0.007053	0.006714
				723.433	0.016149	0.007033	0.00674
				725.942	0.016144	0.007013	0.006765
				728.45	0.016139	0.006994	0.00679
				730.958	0.016133	0.006974	0.006815
				733.467	0.016128	0.006954	0.00684
				735.975	0.016122	0.006935	0.006865
				738.483	0.016116	0.006916	0.00689
				740.992	0.016111	0.006896	0.006915
				743.5	0.016105	0.006877	0.00694

				746.008	0.016099	0.006858	0.006965
				748.517	0.016093	0.006839	0.00699
				751.025	0.016086	0.006821	0.007015
				753.533	0.01608	0.006802	0.00704
				756.042	0.016074	0.006783	0.007065
				758.55	0.016067	0.006765	0.00709
				761.058	0.016061	0.006747	0.007115
				763.567	0.016054	0.006728	0.007139
				766.075	0.016048	0.00671	0.007164
				768.583	0.016041	0.006692	0.007189
				771.092	0.016034	0.006674	0.007214
				773.6	0.016027	0.006656	0.007239
				776.108	0.01602	0.006638	0.007263
				778.617	0.016013	0.006621	0.007288
				781.125	0.016006	0.006603	0.007313
				783.633	0.015999	0.006586	0.007337
				786.137	0.015992	0.006568	0.007362
				788.642	0.015985	0.006551	0.007387
				791.146	0.015977	0.006534	0.007411
				793.65	0.01597	0.006517	0.007436
				796.158	0.015962	0.0065	0.00746
				798.667	0.015955	0.006483	0.007485
				801.175	0.015947	0.006466	0.007509
				803.683	0.015939	0.006449	0.007534
				806.192	0.015932	0.006432	0.007558
				808.7	0.015924	0.006416	0.007583
				811.208	0.015916	0.006399	0.007607
				813.717	0.015908	0.006383	0.007631
				816.225	0.0159	0.006366	0.007656
				818.733	0.015892	0.00635	0.00768
				821.242	0.015884	0.006334	0.007705
				823.75	0.015876	0.006318	0.007729
				826.258	0.015867	0.006302	0.007753
				828.767	0.015859	0.006286	0.007777
				831.275	0.015851	0.00627	0.007802
				833.783	0.015842	0.006254	0.007826
				833.783	0.015842	0.006254	0.007826

Appendix 3: Table of ^{1}H NMR Tracking data for compounds **2a-f**

Table of ^{1}H NMR integration and COPASI fitted data for **2a** → *trans*-fused- and *cis*-fused-**3a** (*p*-OMe) in MeCN

Integral (6.21,6.09)	Integral (5.36,5.25)	Integral (4.47,4.39)	Sum	Time (min)	HA conc (M)	Trans conc (M)	Cis conc (M)	Time (min)	fitted HA conc (M)	fitted Trans conc (M)	fitted Cis conc (M)
71557.3	4783.77	9595.57	85936.64	3.5	0.083268	0.005567	0.011166	0.875	0.095425	0.0015	0.003075
68059.4	5954.73	11990.2	86004.33	4.58	0.079135	0.006924	0.013941	1.75	0.091227	0.00287	0.005902
64974.7	7014.44	14121.5	86110.64	5.65	0.075455	0.008146	0.016399	2.625	0.087363	0.004125	0.008512
62026.7	8005.85	16180.8	86213.35	6.73	0.071946	0.009286	0.018768	3.5	0.083795	0.005277	0.010927
59319.3	8786.13	17826.6	85932.03	7.82	0.06903	0.010225	0.020745	3.77083	0.082746	0.005615	0.011639
56955.3	9541.54	19365.8	85862.64	8.9	0.066333	0.011113	0.022554	4.04167	0.081721	0.005944	0.012335
54709.5	10187.7	21026.5	85923.7	9.98	0.063672	0.011857	0.024471	4.3125	0.080719	0.006265	0.013016
52850.7	10852.2	22551.9	86254.8	11.05	0.061273	0.012582	0.026146	4.58333	0.07974	0.006578	0.013682
50820.1	11389.2	23844.6	86053.9	12.13	0.059056	0.013235	0.027709	4.85	0.078798	0.006879	0.014323
49047.2	11924.3	25109.9	86081.4	13.22	0.056978	0.013852	0.02917	5.11667	0.077877	0.007172	0.014951
46768.6	12550.7	26537.4	85856.7	14.75	0.054473	0.014618	0.030909	5.38333	0.076975	0.007459	0.015566
45266.3	13082	27717	86065.3	15.82	0.052595	0.0152	0.032205	5.65	0.076093	0.007739	0.016168
43746.5	13411.3	28976	86133.8	16.9	0.050789	0.01557	0.033641	5.92083	0.075217	0.008016	0.016767
42414.4	13757.7	29769	85941.1	17.98	0.049353	0.016008	0.034639	6.19167	0.074359	0.008287	0.017354
41189.1	14021.4	30722.8	85933.3	19.07	0.047931	0.016317	0.035752	6.4625	0.073519	0.008552	0.017929
40009.6	14340.4	32067.4	86417.4	20.15	0.046298	0.016594	0.037108	6.73333	0.072697	0.008811	0.018492
38854.9	14664	32464.1	85983	21.22	0.045189	0.017055	0.037756	7.00417	0.071892	0.009063	0.019045
37750.1	14985.5	33173.5	85909.1	22.3	0.043942	0.017443	0.038615	7.275	0.071104	0.00931	0.019586

36718.5	15235.5	33802.5	85756.5	23.38	0.042817	0.017766	0.039417	7.54583	0.070331	0.009551	0.020117
35753.5	15385.9	34556.9	85696.3	24.47	0.041721	0.017954	0.040325	7.81667	0.069575	0.009787	0.020638
34869.7	15581.3	35525.9	85976.9	25.55	0.040557	0.018123	0.04132	8.0875	0.068833	0.010017	0.021149
34043.1	15816.7	36230.3	86090.1	26.62	0.039544	0.018372	0.042084	8.35833	0.068107	0.010243	0.021651
33140.3	15949.6	36549	85638.9	27.7	0.038698	0.018624	0.042678	8.62917	0.067394	0.010463	0.022143
32497.5	16103.5	37269.2	85870.2	28.78	0.037845	0.018753	0.043402	8.9	0.066696	0.010678	0.022626
31710.2	16280	37944.2	85934.4	29.87	0.0369	0.018945	0.044155	9.17083	0.06601	0.010889	0.023101
30941	16442.9	38391.1	85775	30.95	0.036072	0.01917	0.044758	9.44167	0.065339	0.011095	0.023566
30302.9	16572.4	39053.5	85928.8	32.02	0.035265	0.019286	0.045449	9.7125	0.064679	0.011297	0.024024
29716.8	16755.2	39704.9	86176.9	33.1	0.034483	0.019443	0.046074	9.98333	0.064033	0.011494	0.024473
29020.1	16770.3	40324.2	86114.6	34.18	0.033699	0.019474	0.046826	10.25	0.063408	0.011684	0.024908
28412.3	16859.1	40769.9	86041.3	35.27	0.033022	0.019594	0.047384	10.5167	0.062794	0.01187	0.025335
27730.4	17121.8	41055	85907.2	36.35	0.032279	0.019931	0.04779	10.7833	0.062192	0.012053	0.025756
27166.6	17154.5	41567.8	85888.9	37.42	0.03163	0.019973	0.048397	11.05	0.0616	0.012231	0.026169
26643.4	17219.5	42234.4	86097.3	38.5	0.030946	0.02	0.049054	11.3208	0.06101	0.012409	0.026582
26148.4	17319.4	42727.3	86195.1	39.58	0.030336	0.020093	0.04957	11.5917	0.060431	0.012582	0.026987
25655.6	17296.4	43024.1	85976.1	40.67	0.02984	0.020118	0.050042	11.8625	0.059861	0.012752	0.027386
25307.6	17394.7	43650.4	86352.7	41.73	0.029307	0.020144	0.050549	12.1333	0.059302	0.012919	0.027779
24746.2	17457	43797.2	86000.4	42.82	0.028775	0.020299	0.050927	12.4042	0.058753	0.013082	0.028165
24223.5	17502.3	44244.8	85970.6	43.9	0.028176	0.020358	0.051465	12.675	0.058213	0.013242	0.028545
23712.2	17486.7	44774.2	85973.1	44.98	0.027581	0.02034	0.052079	12.9458	0.057683	0.013398	0.028919

23405.5	17663.2	44920	85988.7	46.07	0.027219	0.020541	0.052239	13.2167	0.057162	0.013552	0.029287
23048	17724.4	45477.8	86250.2	47.13	0.026722	0.02055	0.052728	13.6	0.056439	0.013763	0.029798
22684.7	17637.7	45638.1	85960.5	48.22	0.02639	0.020518	0.053092	13.9833	0.055733	0.013969	0.030298
22302	17661.4	46234.9	86198.3	49.3	0.025873	0.020489	0.053638	14.3667	0.055044	0.014169	0.030787
21815.4	17609.2	46672.4	86097	50.38	0.025338	0.020453	0.054209	14.75	0.054371	0.014363	0.031266
21601.8	17794	46808.8	86204.6	51.47	0.025059	0.020642	0.0543	15.0167	0.053912	0.014495	0.031593
21061	17763.4	47163.5	85987.9	52.53	0.024493	0.020658	0.054849	15.2833	0.05346	0.014624	0.031916
20840.2	17747.7	47676.2	86264.1	53.62	0.024159	0.020574	0.055268	15.55	0.053016	0.014751	0.032234
20478.5	17783.9	48006.4	86268.8	54.7	0.023738	0.020615	0.055647	15.8167	0.052578	0.014875	0.032547
20150	17799.6	48381.5	86331.1	55.78	0.02334	0.020618	0.056042	16.0875	0.052141	0.014999	0.032861
19981.4	17781.5	48464.6	86227.5	56.87	0.023173	0.020622	0.056206	16.3583	0.05171	0.01512	0.03317
19737.2	17773	48777	86287.2	57.93	0.022874	0.020597	0.056529	16.6292	0.051286	0.015239	0.033475
19398.3	17728.2	49081.7	86208.2	59.02	0.022502	0.020564	0.056934	16.9	0.050869	0.015356	0.033775
19011.7	17824.1	49458.2	86294	60.1	0.022031	0.020655	0.057314	17.1708	0.050458	0.01547	0.034072
18800.3	17790.9	49757.4	86348.6	61.18	0.021773	0.020604	0.057624	17.4417	0.050054	0.015582	0.034364
18489	17688.6	49990.3	86167.9	62.27	0.021457	0.020528	0.058015	17.7125	0.049656	0.015692	0.034652
18342.5	17657	50200	86199.5	63.33	0.021279	0.020484	0.058237	17.9833	0.049263	0.0158	0.034937
17997.3	17808.8	50860.9	86667	64.42	0.020766	0.020549	0.058685	18.2542	0.048877	0.015906	0.035217
17720.5	17721.4	51132.8	86574.7	65.5	0.020468	0.020469	0.059062	18.525	0.048496	0.01601	0.035494
17608.5	17835.1	51220.3	86663.9	66.58	0.020318	0.02058	0.059102	18.7958	0.048122	0.016111	0.035767
17246.2	17732.1	51552.4	86530.7	67.67	0.019931	0.020492	0.059577	19.0667	0.047752	0.016211	0.036037
16697.3	17736.7	52078.6	86512.	70.3	0.0193	0.020502	0.060198	19.3375	0.047388	0.016309	0.036303

			6								
14944.4	17443	54612	86999.4	80.33	0.017178	0.02005	0.062773	19.6083	0.04703	0.016405	0.036566
13602.7	17069.7	56681.5	87353.9	90.37	0.015572	0.019541	0.064887	19.8792	0.046676	0.016499	0.036825
12360.6	16527.9	58534.6	87423.1	100.43	0.014139	0.018906	0.066956	20.15	0.046328	0.016591	0.037081
6497.08	12167	70580.4	89244.48	197.07	0.00728	0.013633	0.079087	20.4167	0.04599	0.016681	0.03733
4722.62	9913.76	75485.7	90122.08	269.48	0.00524	0.011	0.083759	20.6833	0.045656	0.016768	0.037576
3592.23	8575.56	78511.8	90679.59	329.68	0.003961	0.009457	0.086582	20.95	0.045328	0.016854	0.037819
								21.2167	0.045003	0.016938	0.038058
								21.4875	0.044679	0.017022	0.038299
								21.7583	0.044358	0.017105	0.038537
								22.0292	0.044043	0.017186	0.038772
								22.3	0.043731	0.017265	0.039004
								22.5708	0.043424	0.017343	0.039233
								22.8417	0.043121	0.017419	0.03946
								23.1125	0.042822	0.017494	0.039684
								23.3833	0.042527	0.017567	0.039905
								23.6542	0.042236	0.017639	0.040124
								23.925	0.041949	0.01771	0.040341
								24.1958	0.041666	0.017779	0.040555
								24.4667	0.041387	0.017848	0.040766
								24.7375	0.041111	0.017914	0.040975
								25.0083	0.040838	0.01798	0.041182
								25.2792	0.04057	0.018044	0.041387
								25.55	0.040304	0.018107	0.041589
								25.8167	0.040046	0.018168	0.041786
								26.0833	0.039792	0.018228	0.041981
								26.35	0.03954	0.018286	0.042174
								26.6167	0.039292	0.018344	0.042365

								26.8875	0.039042	0.018401	0.042557
								27.1583	0.038796	0.018457	0.042746
								27.4292	0.038553	0.018512	0.042934
								27.7	0.038313	0.018566	0.04312
								27.9708	0.038076	0.018619	0.043304
								28.2417	0.037842	0.018671	0.043486
								28.5125	0.037611	0.018722	0.043667
								28.7833	0.037382	0.018772	0.043845
								29.0542	0.037157	0.018822	0.044022
								29.325	0.036934	0.01887	0.044197
								29.5958	0.036713	0.018917	0.04437
								29.8667	0.036496	0.018963	0.044542
								30.1375	0.03628	0.019008	0.044711
								30.4083	0.036068	0.019053	0.04488
								30.6792	0.035858	0.019096	0.045046
								30.95	0.03565	0.019139	0.045211
								31.2167	0.035448	0.01918	0.045372
								31.4833	0.035248	0.019221	0.045532
								31.75	0.03505	0.01926	0.04569
								32.0167	0.034855	0.019299	0.045846
								32.2875	0.034659	0.019338	0.046004
								32.5583	0.034465	0.019375	0.04616
								32.8292	0.034273	0.019413	0.046315
								33.1	0.034083	0.019449	0.046468
								33.3708	0.033896	0.019484	0.04662
								33.6417	0.03371	0.019519	0.04677
								33.9125	0.033527	0.019553	0.04692
								34.1833	0.033346	0.019587	0.047068
								34.4542	0.033166	0.019619	0.047214
								34.725	0.032989	0.019651	0.04736
								34.9958	0.032814	0.019683	0.047504

								35.2667	0.03264	0.019714	0.047647
								35.5375	0.032468	0.019744	0.047788
								35.8083	0.032299	0.019773	0.047929
								36.0792	0.032131	0.019802	0.048068
								36.35	0.031964	0.01983	0.048206
								36.6167	0.031803	0.019857	0.048341
								36.8833	0.031642	0.019883	0.048474
								37.15	0.031484	0.019909	0.048607
								37.4167	0.031327	0.019935	0.048739
								37.6875	0.031169	0.01996	0.048871
								37.9583	0.031013	0.019984	0.049003
								38.2292	0.030859	0.020008	0.049133
								38.5	0.030706	0.020032	0.049263
								38.7708	0.030554	0.020055	0.049391
								39.0417	0.030405	0.020077	0.049518
								39.3125	0.030257	0.020099	0.049645
								39.5833	0.03011	0.02012	0.04977
								39.8542	0.029965	0.020141	0.049895
								40.125	0.029821	0.020161	0.050018
								40.3958	0.029679	0.020181	0.050141
								40.6667	0.029538	0.0202	0.050262
								40.9333	0.0294	0.020219	0.050381
								41.2	0.029264	0.020237	0.050499
								41.4667	0.02913	0.020254	0.050616
								41.7333	0.028996	0.020272	0.050732
								42.0042	0.028862	0.020289	0.05085
								42.275	0.028729	0.020305	0.050966
								42.5458	0.028598	0.020321	0.051081
								42.8167	0.028468	0.020337	0.051196
								43.0875	0.028339	0.020352	0.05131
								43.3583	0.028211	0.020367	0.051423

								43.6292	0.028084	0.020381	0.051535
								43.9	0.027959	0.020395	0.051646
								44.1708	0.027835	0.020408	0.051757
								44.4417	0.027712	0.020421	0.051867
								44.7125	0.02759	0.020434	0.051976
								44.9833	0.02747	0.020446	0.052084
								45.2542	0.02735	0.020458	0.052192
								45.525	0.027232	0.02047	0.052299
								45.7958	0.027114	0.020481	0.052405
								46.0667	0.026998	0.020492	0.05251
								46.3333	0.026885	0.020502	0.052613
								46.6	0.026773	0.020512	0.052716
								46.8667	0.026661	0.020521	0.052817
								47.1333	0.026551	0.020531	0.052918
								47.4042	0.02644	0.02054	0.05302
								47.675	0.02633	0.020548	0.053122
								47.9458	0.026221	0.020557	0.053222
								48.2167	0.026113	0.020565	0.053322
								48.4875	0.026006	0.020572	0.053422
								48.7583	0.0259	0.02058	0.053521
								49.0292	0.025795	0.020587	0.053619
								49.3	0.025691	0.020593	0.053716
								49.5708	0.025587	0.0206	0.053813
								49.8417	0.025485	0.020606	0.053909
								50.1125	0.025384	0.020612	0.054005
								50.3833	0.025283	0.020617	0.0541
								50.6542	0.025184	0.020622	0.054194
								50.925	0.025085	0.020627	0.054288
								51.1958	0.024987	0.020632	0.054382
								51.4667	0.02489	0.020636	0.054475
								51.7333	0.024795	0.02064	0.054565

								52	0.024701	0.020644	0.054656
								52.2667	0.024608	0.020647	0.054745
								52.5333	0.024515	0.02065	0.054835
								52.8042	0.024422	0.020653	0.054925
								53.075	0.02433	0.020656	0.055014
								53.3458	0.024239	0.020658	0.055103
								53.6167	0.024148	0.02066	0.055192
								53.8875	0.024058	0.020662	0.05528
								54.1583	0.023969	0.020664	0.055367
								54.4292	0.023881	0.020665	0.055454
								54.7	0.023793	0.020666	0.055541
								54.9708	0.023706	0.020667	0.055627
								55.2417	0.02362	0.020668	0.055712
								55.5125	0.023534	0.020669	0.055797
								55.7833	0.023449	0.020669	0.055882
								56.0542	0.023365	0.020669	0.055966
								56.325	0.023282	0.020668	0.05605
								56.5958	0.023199	0.020668	0.056133
								56.8667	0.023117	0.020667	0.056216
								57.1333	0.023037	0.020666	0.056297
								57.4	0.022958	0.020665	0.056377
								57.6667	0.022879	0.020664	0.056457
								57.9333	0.0228	0.020663	0.056537
								58.2042	0.022722	0.020661	0.056618
								58.475	0.022643	0.020659	0.056698
								58.7458	0.022566	0.020657	0.056778
								59.0167	0.022489	0.020654	0.056857
								59.2875	0.022412	0.020652	0.056936
								59.5583	0.022337	0.020649	0.057014
								59.8292	0.022261	0.020646	0.057092
								60.1	0.022187	0.020643	0.05717

								60.3708	0.022113	0.02064	0.057247
								60.6417	0.022039	0.020636	0.057324
								60.9125	0.021967	0.020633	0.057401
								61.1833	0.021894	0.020629	0.057477
								61.4542	0.021823	0.020625	0.057553
								61.725	0.021751	0.020621	0.057628
								61.9958	0.021681	0.020616	0.057703
								62.2667	0.021611	0.020612	0.057778
								62.5333	0.021542	0.020607	0.057851
								62.8	0.021474	0.020602	0.057924
								63.0667	0.021407	0.020597	0.057996
								63.3333	0.02134	0.020592	0.058068
								63.6042	0.021272	0.020587	0.058141
								63.875	0.021205	0.020581	0.058213
								64.1458	0.021139	0.020576	0.058286
								64.4167	0.021073	0.02057	0.058357
								64.6875	0.021007	0.020564	0.058429
								64.9583	0.020942	0.020558	0.0585
								65.2292	0.020878	0.020551	0.058571
								65.5	0.020814	0.020545	0.058641
								65.7708	0.02075	0.020538	0.058712
								66.0417	0.020687	0.020532	0.058781
								66.3125	0.020625	0.020525	0.058851
								66.5833	0.020562	0.020518	0.05892
								66.8542	0.020501	0.020511	0.058989
								67.125	0.020439	0.020503	0.059058
								67.3958	0.020378	0.020496	0.059126
								67.6667	0.020318	0.020488	0.059194
								68.325	0.020173	0.020469	0.059358
								68.9833	0.020031	0.02045	0.05952
								69.6417	0.01989	0.020429	0.059681

								70.3	0.019753	0.020408	0.059839
								72.8083	0.019249	0.020321	0.06043
								75.3167	0.018776	0.020225	0.060998
								77.825	0.018332	0.020122	0.061547
								80.3333	0.017913	0.02001	0.062076
								82.8417	0.017518	0.019893	0.062589
								85.35	0.017146	0.01977	0.063085
								87.8583	0.016793	0.019642	0.063566
								90.3667	0.016459	0.019509	0.064033
								92.8833	0.016141	0.019372	0.064488
								95.4	0.015839	0.019231	0.06493
								97.9167	0.015552	0.019087	0.065361
								100.433	0.015278	0.018941	0.065781
								124.592	0.013188	0.017455	0.069357
								148.75	0.011726	0.015943	0.072331
								172.908	0.01061	0.014495	0.074896
								197.067	0.009701	0.013145	0.077154
								215.171	0.009111	0.012206	0.078683
								233.275	0.008576	0.01133	0.080094
								251.379	0.008086	0.010515	0.0814
								269.483	0.007631	0.009758	0.082611
								284.533	0.007277	0.009171	0.083552
								299.583	0.006941	0.00862	0.084438
								314.633	0.006623	0.008104	0.085274
								329.683	0.006319	0.007619	0.086062
								329.683	0.006319	0.007619	0.086062

Table of ^{1}H NMR integration and COPASI fitted data for **2b** → *trans*-fused- and *cis*-fused-**3b** (*p*-tBu) in MeCN

Integral (6.54,6.40)	Integral (5.36,5.24)	Integral (4.50,4.40)	Sum	Time (min)	HA (M)	Trans (M)	Cis (M)	Fitted time	Fitted HA (M)	Fitted trans (M)	Fitted cis (M)
18574.5	384.88	259.75	19219.12	3.5	0.096646	0.002003	0.001352	0	0.1	0	0
18425	464.65	326.78	19216.43	4.57	0.095881	0.002418	0.001701	0.875	0.0993	0.000419	0.000282
18233.5	576.67	380.96	19191.13	5.65	0.09501	0.003005	0.001985	1.75	0.09861	0.000831	0.000559
18099.4	675.9	451.69	19226.99	6.73	0.094135	0.003515	0.002349	2.625	0.09793	0.001237	0.000834
17956.8	776.53	508.53	19241.85	7.82	0.093322	0.004036	0.002643	3.5	0.09726	0.001636	0.001104
17782.9	853.75	564.44	19201.09	8.9	0.092614	0.004446	0.00294	3.7675	0.097057	0.001757	0.001187
17664.9	939.82	634.41	19239.13	9.97	0.091818	0.004885	0.003297	4.035	0.096855	0.001877	0.001268
17500.7	1033.88	682.38	19216.96	11.05	0.091069	0.00538	0.003551	4.3025	0.096653	0.001997	0.00135
17362.3	1108.57	749.25	19220.12	12.13	0.090334	0.005768	0.003898	4.57	0.096453	0.002116	0.001431
17204.8	1193.52	792	19190.32	13.22	0.089654	0.006219	0.004127	4.84	0.096252	0.002236	0.001512
17023.1	1305.02	869.54	19197.66	14.62	0.088673	0.006798	0.004529	5.11	0.096052	0.002355	0.001594
16900.4	1374.66	928.26	19203.32	15.7	0.088008	0.007158	0.004834	5.38	0.095852	0.002473	0.001675
16768	1458.02	968.91	19194.93	16.78	0.087356	0.007596	0.005048	5.65	0.095653	0.002591	0.001755
16676.4	1529.8	1032.28	19238.48	17.85	0.086683	0.007952	0.005366	5.92	0.095456	0.002709	0.001836
16525.6	1603.41	1074.94	19203.95	18.93	0.086053	0.008349	0.005597	6.19	0.095259	0.002826	0.001916
16406.8	1669.41	1130.46	19206.67	20.02	0.085422	0.008692	0.005886	6.46	0.095063	0.002942	0.001995
16328	1741.79	1179.6	19249.39	21.1	0.084823	0.009049	0.006128	6.73	0.094868	0.003058	0.002075
16157.9	1810.42	1227.09	19195.41	22.18	0.084176	0.009432	0.006393	7.0025	0.094672	0.003174	0.002155
16059.4	1862.86	1271.73	19193.99	23.25	0.083669	0.009705	0.006626	7.275	0.094476	0.00329	0.002234
15979.6	1938.52	1321.38	19239.5	24.33	0.083056	0.010076	0.006868	7.5475	0.094282	0.003405	0.002313
15895.3	2011.93	1370.85	19278.08	25.42	0.082453	0.010436	0.007111	7.82	0.094089	0.003519	0.002392
15803	2075.97	1419.75	19298.72	26.5	0.081886	0.010757	0.007357	8.09	0.093898	0.003632	0.00247
15641.1	2133.9	1462.11	19237.11	27.58	0.081307	0.011093	0.0076	8.36	0.093708	0.003745	0.002548
15497.7	2193.33	1505.26	19196.29	28.65	0.080733	0.011426	0.007841	8.63	0.093518	0.003857	0.002625
15443.1	2240.57	1545.93	19229.6	29.73	0.080309	0.011652	0.008039	8.9	0.09333	0.003968	0.002702
15338	2314.5	1597.87	19250.37	30.82	0.079676	0.012023	0.0083	9.1675	0.093144	0.004078	0.002778
15235.7	2361.65	1632.47	19229.82	31.9	0.07923	0.012281	0.008489	9.435	0.092959	0.004187	0.002854
15154	2423.3	1689.74	1926	32.98	0.07	0.01	0.00	9.702	0.0927	0.00429	0.0029

			7.04		8652	2577	877	5	75	6	29
15080.1	2472.76	1721.84	19274.7	34.05	0.078238	0.012829	0.008933	9.97	0.092591	0.004405	0.003004
14988.4	2521.62	1764	19274.02	35.13	0.077765	0.013083	0.009152	10.24	0.092407	0.004513	0.00308
14841	2579.02	1808.45	19228.47	36.22	0.077182	0.013413	0.009405	10.51	0.092223	0.004622	0.003155
14788.8	2638.59	1843.14	19270.53	37.3	0.076743	0.013692	0.009565	10.78	0.09204	0.00473	0.00323
14704.5	2688.8	1880.96	19274.26	38.38	0.076291	0.01395	0.009759	11.05	0.091858	0.004837	0.003305
14578.2	2730.56	1919.44	19228.2	39.45	0.075817	0.014201	0.009982	11.32	0.091677	0.004944	0.00338
14521	2779.31	1967.9	19268.21	40.53	0.075362	0.014424	0.010213	11.59	0.091496	0.00505	0.003454
14461.2	2823.08	2003.09	19287.37	41.62	0.074978	0.014637	0.010386	11.86	0.091316	0.005156	0.003528
14367.9	2879.23	2036.38	19283.51	42.7	0.074509	0.014931	0.01056	12.13	0.091137	0.005261	0.003602
14287.5	2928.96	2110.37	19326.83	43.78	0.073926	0.015155	0.010919	12.4025	0.090957	0.005367	0.003676
14234.9	2985.35	2112.2	19332.45	44.85	0.073632	0.015442	0.010926	12.675	0.090778	0.005472	0.003749
14118.2	3043.3	2177.44	19338.94	45.93	0.073004	0.015737	0.011259	12.9475	0.0906	0.005577	0.003823
14055.3	3076.3	2213.59	19345.19	47.02	0.072655	0.015902	0.011443	13.22	0.090422	0.005681	0.003896
13943.3	3101.11	2228.69	19273.1	48.1	0.072346	0.01609	0.011564	13.57	0.090195	0.005815	0.00399
13890.9	3143.18	2260.28	19294.36	49.18	0.071995	0.016291	0.011715	13.92	0.08997	0.005947	0.004083
13811.6	3211.15	2309.12	19331.87	50.25	0.071445	0.016611	0.011945	14.27	0.089745	0.006079	0.004176
13831.5	3257.08	2369.85	19458.43	51.33	0.071082	0.016739	0.012179	14.62	0.089522	0.00621	0.004269
13733.4	3279.7	2373.23	19386.33	52.42	0.070841	0.016918	0.012242	14.89	0.08935	0.00631	0.00434
13618.4	3300.39	2381.06	19299.85	53.5	0.070562	0.017101	0.012337	15.16	0.089179	0.00641	0.00441
13526.6	3322.13	2422.07	19270.8	54.58	0.070192	0.017239	0.012569	15.43	0.089009	0.00651	0.004481
13416.1	3388.24	2463.38	19267.72	55.65	0.06963	0.017585	0.012785	15.7	0.08884	0.006609	0.004551
13401	3410.36	2502.42	19313.78	56.73	0.069386	0.017658	0.012957	15.97	0.088671	0.006708	0.004621
13322.2	3459.47	2536.4	19318.07	57.82	0.068962	0.017908	0.01313	16.24	0.088503	0.006806	0.004691
13307.3	3502.44	2562.85	19372.59	58.9	0.068691	0.018079	0.013229	16.51	0.088336	0.006904	0.00476
13165.7	3519.56	2590.92	19276.18	59.98	0.0683	0.018259	0.013441	16.78	0.08817	0.007001	0.00483
13138.3	3595.74	2630.03	19364.07	61.05	0.067849	0.018569	0.013582	17.0475	0.088005	0.007097	0.004898
13054.9	3593.17	2669.08	19317.15	62.13	0.067582	0.018601	0.013817	17.315	0.087842	0.007192	0.004966
12991.7	3614.63	2691.02	19297.35	63.22	0.067324	0.018731	0.013945	17.5825	0.087679	0.007287	0.005034
12958.3	3672.88	2739.92	19371.1	64.3	0.066895	0.018961	0.014144	17.85	0.087516	0.007382	0.005102
12880.6	3694.04	2756.34	19330.98	65.38	0.066632	0.019109	0.014259	18.12	0.087353	0.007477	0.00517

12774.4	3705.89	2761.76	19242.05	66.45	0.066388	0.019259	0.014353	18.39	0.087191	0.007572	0.005238
12813.5	3763.07	2814.44	19391.01	67.53	0.06608	0.019406	0.014514	18.66	0.087029	0.007666	0.005305
12730.8	3790.72	2819.03	19340.55	68.62	0.065824	0.0196	0.014576	18.93	0.086867	0.00776	0.005373
12673.3	3838.73	2908.95	19420.98	69.7	0.065256	0.019766	0.014978	19.2025	0.086705	0.007854	0.00544
12527	3858.07	2896.61	19281.68	70.78	0.064968	0.020009	0.015023	19.475	0.086544	0.007948	0.005508
12501.5	3887.99	2915.75	19305.24	71.85	0.064757	0.02014	0.015103	19.7475	0.086383	0.008042	0.005575
12377.6	3931.32	2965.24	19274.16	72.93	0.064219	0.020397	0.015385	20.02	0.086223	0.008135	0.005642
12388.9	3951.79	2988.72	19329.41	74.02	0.064094	0.020444	0.015462	20.29	0.086065	0.008226	0.005708
12360.8	4003.09	3050.48	19414.37	75.1	0.063668	0.020619	0.015712	20.56	0.085908	0.008318	0.005774
12246.6	4002.78	3027.3	19276.68	76.18	0.063531	0.020765	0.015704	20.83	0.085751	0.008408	0.00584
12235.4	4023.92	3067.93	19327.25	77.25	0.063306	0.02082	0.015874	21.1	0.085595	0.008499	0.005906
12281.8	4071.34	3113.73	19466.87	78.33	0.063091	0.020914	0.015995	21.37	0.08544	0.008589	0.005971
12029.7	4147.93	3200.38	19378.01	82.02	0.062079	0.021405	0.016516	21.64	0.085285	0.008679	0.006036
11452.2	4333.52	3313.97	19099.69	92.05	0.05996	0.022689	0.017351	21.91	0.085131	0.008768	0.006101
11110.1	4500.47	3646.43	19257	102.08	0.057694	0.023371	0.018936	22.18	0.084978	0.008857	0.006166
10686.8	4627.48	3787.02	19101.3	112.12	0.055948	0.024226	0.019826	22.4475	0.084826	0.008944	0.006229
10403.8	4728.08	3993.63	19125.51	122.15	0.054398	0.024721	0.020881	22.715	0.084675	0.009032	0.006293
10059.2	4881.92	4138.82	19079.94	132.18	0.052721	0.025587	0.021692	22.9825	0.084525	0.009119	0.006357
9982.44	5057.43	4444.39	19484.26	142.22	0.051233	0.025956	0.02281	23.25	0.084375	0.009205	0.00642
9757.55	5145.77	4636.34	19539.66	152.25	0.049937	0.026335	0.023728	23.52	0.084225	0.009292	0.006483
9550.57	5201.62	4783.2	19535.39	162.28	0.048889	0.026627	0.024485	23.79	0.084075	0.009379	0.006547
9303.96	5286.78	4955.94	19546.68	172.3	0.047599	0.027047	0.025354	24.06	0.083925	0.009465	0.00661
9090.12	5321.33	5096.76	19508.21	182.33	0.046596	0.027277	0.026126	24.33	0.083777	0.00955	0.006673
8946.89	5374.07	5240.69	19561.65	192.37	0.045737	0.027472	0.026791	24.6025	0.083627	0.009637	0.006736
8777.66	5406.91	5357.4	19541.97	202.4	0.044917	0.027668	0.027415	24.875	0.083478	0.009722	0.006799
8599.85	5434.66	5491.42	19525.93	212.43	0.044043	0.027833	0.028124	25.1475	0.08333	0.009808	0.006862
8472.63	5448.38	5613.05	19534.06	222.47	0.043374	0.027892	0.028735	25.42	0.083182	0.009893	0.006925
8318.65	5438.27	5709.24	19466.16	232.5	0.042734	0.027937	0.029329	25.69	0.083036	0.009977	0.006987
8183.09	5465.62	5860.58	19509.29	242.53	0.041945	0.028015	0.03004	25.96	0.082891	0.01006	0.007049
8081.78	5482.27	5983.28	19547.33	252.55	0.041345	0.028046	0.030609	26.23	0.082747	0.010143	0.00711
7984.36	5467.8	6056.43	19508.59	262.58	0.040927	0.028028	0.031045	26.5	0.082602	0.010226	0.007171

7856.79	5461.29	6187.8	19505.88	272.62	0.040279	0.027998	0.031723	26.77	0.082459	0.010309	0.007233
7756.5	5446.95	6323.22	19526.67	282.65	0.039723	0.027895	0.032382	27.04	0.082316	0.010391	0.007294
7672.5	5468.18	6397.63	19538.31	292.68	0.039269	0.027987	0.032744	27.31	0.082174	0.010472	0.007354
7583.07	5458.69	6486.07	19527.83	302.72	0.038832	0.027953	0.033214	27.58	0.082032	0.010554	0.007415
7523.57	5422.45	6634.52	19580.54	312.75	0.038424	0.027693	0.033883	27.8475	0.081892	0.010634	0.007475
7415.54	5425.57	6687.21	19528.32	322.78	0.037973	0.027783	0.034244	28.115	0.081752	0.010714	0.007534
7374.89	5391.14	6785.58	19551.61	332.8	0.03772	0.027574	0.034706	28.3825	0.081613	0.010793	0.007594
7286.17	5369.45	6856.18	19511.8	342.83	0.037342	0.027519	0.035139	28.65	0.081475	0.010872	0.007653
7178.8	5348.03	6936.66	19463.49	352.87	0.036883	0.027477	0.035639	28.92	0.081336	0.010952	0.007712
7143.06	5349.48	7082.4	19574.94	362.9	0.036491	0.027328	0.036181	29.19	0.081197	0.011031	0.007772
7047.72	5295.62	7138.91	19482.25	372.93	0.036175	0.027182	0.036643	29.46	0.081059	0.01111	0.007831
6998.15	5285.35	7266.44	19549.94	382.97	0.035796	0.027035	0.037169	29.73	0.080922	0.011188	0.00789
6921.07	5252.1	7289.53	19462.7	393	0.035561	0.026985	0.037454	30.0025	0.080783	0.011267	0.007949
6858.99	5212.93	7392.57	19464.49	403.03	0.035238	0.026782	0.03798	30.275	0.080646	0.011346	0.008009
6499.31	5190.92	7611.57	19301.8	413.07	0.033672	0.026893	0.039435	30.5475	0.080508	0.011424	0.008068
6754.53	5154.83	7540.03	19449.39	423.08	0.034729	0.026504	0.038767	30.82	0.080372	0.011502	0.008126
6694.08	5130	7621.4	19445.48	433.12	0.034425	0.026381	0.039194	31.09	0.080237	0.011579	0.008185
6629.98	5105.3	7689.44	19424.72	443.15	0.034132	0.026282	0.039586	31.36	0.080102	0.011655	0.008242
6597.34	5075.43	7794.3	19467.07	453.18	0.03389	0.026072	0.040038	31.63	0.079969	0.011731	0.0083
6555.03	5032.44	7826.88	19414.35	463.22	0.033764	0.025921	0.040315	31.9	0.079835	0.011807	0.008358
6468.96	5001.74	7889.84	19360.54	473.25	0.033413	0.025835	0.040752	32.17	0.079702	0.011883	0.008415
6446.84	4962.31	7951.63	19360.78	483.28	0.033298	0.025631	0.041071	32.44	0.07957	0.011958	0.008472
6378.57	4937.7	8060.76	19377.03	493.32	0.032918	0.025482	0.0416	32.71	0.079438	0.012033	0.008529
6336.04	4916.15	8034.79	19286.98	503.35	0.032851	0.025489	0.041659	32.98	0.079307	0.012107	0.008586
6298.74	4849.1	8126.38	19274.22	513.37	0.03268	0.025158	0.042162	33.2475	0.079177	0.012181	0.008642
6255.35	4835.78	8247.45	19338.58	523.4	0.032346	0.025006	0.042648	33.515	0.079048	0.012254	0.008698
6221.46	4803.84	8273.25	19298.55	533.43	0.032238	0.024892	0.04287	33.7825	0.078919	0.012327	0.008754
6171.48	4785.08	8362.42	19318.98	543.47	0.031945	0.024769	0.043286	34.05	0.078791	0.012399	0.00881
6122.09	4740.51	8422.53	19285.13	553.5	0.031745	0.024581	0.043674	34.32	0.078662	0.012472	0.008866
6087.37	4707.25	8497.95	19292.57	563.53	0.031553	0.024399	0.044048	34.59	0.078534	0.012545	0.008922
6059.4	4724.65	8556.06	19340.11	573.57	0.031331	0.024429	0.04424	34.86	0.078406	0.012617	0.008977

6047.65	4709.58	8664.59	19421.82	583.6	0.031138	0.024249	0.044613	35.13	0.078278	0.012689	0.009033
5995.5	4673.02	8744.46	19412.98	593.62	0.030884	0.024072	0.045044	35.4025	0.07815	0.012761	0.009089
5962.71	4620.56	8749	19332.27	603.65	0.030843	0.023901	0.045256	35.675	0.078023	0.012833	0.009144
5959.5	4627.57	8840.48	19427.55	613.68	0.030676	0.02382	0.045505	35.9475	0.077895	0.012905	0.0092
5888.95	4564.11	8892.78	19345.84	623.72	0.03044	0.023592	0.045967	36.22	0.077769	0.012976	0.009255
5864.2	4593.53	8976.3	19434.03	633.75	0.030175	0.023637	0.046189	36.49	0.077644	0.013047	0.00931
5818	4497.85	8941.99	19257.84	643.78	0.030211	0.023356	0.046433	36.76	0.077519	0.013117	0.009364
5812.05	4507.79	9081.07	19400.91	653.82	0.029958	0.023235	0.046807	37.03	0.077395	0.013187	0.009418
5777.11	4456.62	9135.99	19369.72	663.85	0.029825	0.023008	0.047166	37.3	0.077271	0.013256	0.009473
5751.87	4422.2	9178.66	19352.73	673.88	0.029721	0.022851	0.047428	37.57	0.077148	0.013326	0.009527
5706.61	4416.01	9238.05	19360.67	683.9	0.029475	0.022809	0.047716	37.84	0.077025	0.013395	0.00958
5676.41	4376.79	9270.91	19324.11	693.93	0.029375	0.022649	0.047976	38.11	0.076903	0.013463	0.009634
5663.93	4383.62	9385.72	19433.27	703.97	0.029146	0.022557	0.048297	38.38	0.076781	0.013532	0.009688
5645.59	4369.62	9435.97	19451.18	714	0.029024	0.022465	0.048511	38.6475	0.076661	0.013599	0.009741
5595.32	4307.44	9485.96	19388.72	724.03	0.028859	0.022216	0.048925	38.915	0.076541	0.013666	0.009793
5590.64	4284.25	9473.16	19348.05	734.07	0.028895	0.022143	0.048962	39.1825	0.076421	0.013733	0.009846
5541.92	4244.54	9598.79	19385.25	744.1	0.028588	0.021896	0.049516	39.45	0.076302	0.0138	0.009898
5522.56	4224.32	9646.57	19393.45	754.13	0.028476	0.021782	0.049741	39.72	0.076183	0.013866	0.009951
5496.17	4173.4	9728.79	19398.36	764.15	0.028333	0.021514	0.050153	39.99	0.076064	0.013933	0.010004
5481.01	4226.41	9770.14	19477.56	774.18	0.02814	0.021699	0.050161	40.26	0.075945	0.013999	0.010056
5449.82	4159.95	9790.18	19399.95	784.22	0.028092	0.021443	0.050465	40.53	0.075826	0.014065	0.010108
5413.15	4148.89	9874.67	19436.71	794.25	0.02785	0.021346	0.050804	40.8025	0.075707	0.014132	0.010161
5413.96	4134.59	9957.85	19506.4	804.28	0.027755	0.021196	0.051049	41.075	0.075589	0.014198	0.010213
5398.69	4098.6	9982.75	19480.04	814.32	0.027714	0.02104	0.051246	41.3475	0.075471	0.014264	0.010266
5356.58	4117.92	10055.3	19529.8	824.35	0.027428	0.021085	0.051487	41.62	0.075353	0.014329	0.010318
5330.36	4022.43	10003.4	19356.19	834.38	0.027538	0.020781	0.051681	41.89	0.075237	0.014394	0.010369
5297.04	3998.43	10122	19417.47	844.4	0.02728	0.020592	0.052128	42.16	0.075121	0.014458	0.010421
5305.86	3991.38	10192.6	19489.84	854.43	0.027224	0.020479	0.052297	42.43	0.075006	0.014522	0.010472
5271.96	3966.93	10225.5	19464.39	864.47	0.027085	0.02038	0.052534	42.7	0.074891	0.014586	0.010523
5240.26	3939.71	10264.8	19444.77	874.5	0.026949	0.020261	0.05279	42.97	0.074776	0.01465	0.010574
5199.64	3972.46	10336.7	19508.8	884.53	0.026653	0.020362	0.052985	43.24	0.074662	0.014713	0.010625

5180.34	3875.7	10326.4	19382.44	894.57	0.026727	0.019996	0.053277	43.51	0.074548	0.014776	0.010675
5146.66	3863.64	10379.9	19390.2	904.6	0.026543	0.019926	0.053532	43.78	0.074435	0.014839	0.010726
								44.0475	0.074323	0.014901	0.010776
								44.315	0.074212	0.014963	0.010826
								44.5825	0.074101	0.015024	0.010875
								44.85	0.07399	0.015085	0.010925
								45.12	0.073879	0.015147	0.010975
								45.39	0.073768	0.015208	0.011024
								45.66	0.073658	0.015269	0.011074
								45.93	0.073548	0.01533	0.011123
								46.2025	0.073437	0.015391	0.011173
								46.475	0.073327	0.015451	0.011222
								46.7475	0.073217	0.015512	0.011272
								47.02	0.073107	0.015572	0.011321
								47.29	0.072999	0.015631	0.01137
								47.56	0.072891	0.015691	0.011418
								47.83	0.072784	0.015749	0.011467
								48.1	0.072677	0.015808	0.011515
								48.37	0.07257	0.015867	0.011563
								48.64	0.072464	0.015925	0.011611
								48.91	0.072358	0.015983	0.011659
								49.18	0.072253	0.01604	0.011707
								49.4475	0.072149	0.016097	0.011754
								49.715	0.072045	0.016154	0.011801
								49.9825	0.071941	0.016211	0.011848
								50.25	0.071838	0.016267	0.011895
								50.52	0.071735	0.016323	0.011942
								50.79	0.071631	0.01638	0.011989
								51.06	0.071528	0.016436	0.012036
								51.33	0.071426	0.016491	0.012083
								51.6025	0.071323	0.016548	0.01213

								51.875	0.07122	0.016603	0.012177
								52.1475	0.071117	0.016659	0.012224
								52.42	0.071015	0.016714	0.01227
								52.69	0.070915	0.016769	0.012317
								52.96	0.070814	0.016824	0.012363
								53.23	0.070714	0.016878	0.012408
								53.5	0.070614	0.016932	0.012454
								53.77	0.070515	0.016985	0.0125
								54.04	0.070416	0.017039	0.012545
								54.31	0.070317	0.017092	0.012591
								54.58	0.070219	0.017145	0.012636
								54.8475	0.070122	0.017198	0.012681
								55.115	0.070025	0.01725	0.012726
								55.3825	0.069928	0.017302	0.01277
								55.65	0.069832	0.017354	0.012814
								55.92	0.069735	0.017406	0.012859
								56.19	0.069639	0.017457	0.012904
								56.46	0.069543	0.017509	0.012948
								56.73	0.069447	0.01756	0.012993
								57.0025	0.069351	0.017612	0.013037
								57.275	0.069255	0.017663	0.013082
								57.5475	0.069159	0.017715	0.013126
								57.82	0.069064	0.017766	0.013171
								58.09	0.06897	0.017816	0.013214
								58.36	0.068876	0.017866	0.013258
								58.63	0.068783	0.017916	0.013302
								58.9	0.068689	0.017966	0.013345
								59.17	0.068597	0.018015	0.013389
								59.44	0.068504	0.018064	0.013432
								59.71	0.068412	0.018113	0.013475
								59.98	0.06832	0.018162	0.013518

								60.2475	0.068229	0.018211	0.01356
								60.515	0.068139	0.018259	0.013603
								60.7825	0.068048	0.018306	0.013645
								61.05	0.067959	0.018354	0.013687
								61.32	0.067868	0.018402	0.01373
								61.59	0.067778	0.01845	0.013772
								61.86	0.067688	0.018497	0.013815
								62.13	0.067599	0.018544	0.013857
								62.4025	0.067509	0.018592	0.013899
								62.675	0.067419	0.018639	0.013942
								62.9475	0.06733	0.018686	0.013984
								63.22	0.067241	0.018733	0.014026
								63.49	0.067153	0.01878	0.014068
								63.76	0.067065	0.018826	0.014109
								64.03	0.066977	0.018872	0.014151
								64.3	0.06689	0.018918	0.014192
								64.57	0.066804	0.018963	0.014233
								64.84	0.066717	0.019009	0.014275
								65.11	0.066631	0.019054	0.014316
								65.38	0.066545	0.019099	0.014357
								65.6475	0.06646	0.019143	0.014397
								65.915	0.066375	0.019188	0.014437
								66.1825	0.066291	0.019232	0.014478
								66.45	0.066207	0.019275	0.014518
								66.72	0.066122	0.01932	0.014558
								66.99	0.066038	0.019364	0.014599
								67.26	0.065954	0.019407	0.014639
								67.53	0.06587	0.019451	0.014679
								67.8025	0.065786	0.019495	0.01472
								68.075	0.065702	0.019538	0.01476
								68.3475	0.065618	0.019582	0.0148

								68.62	0.065535	0.019625	0.014841
								68.89	0.065452	0.019668	0.01488
								69.16	0.06537	0.01971	0.01492
								69.43	0.065288	0.019753	0.014959
								69.7	0.065207	0.019795	0.014999
								69.97	0.065125	0.019837	0.015038
								70.24	0.065044	0.019879	0.015077
								70.51	0.064963	0.01992	0.015117
								70.78	0.064883	0.019962	0.015156
								71.0475	0.064803	0.020003	0.015194
								71.315	0.064724	0.020043	0.015233
								71.5825	0.064645	0.020084	0.015271
								71.85	0.064566	0.020124	0.01531
								72.12	0.064487	0.020165	0.015348
								72.39	0.064408	0.020206	0.015387
								72.66	0.064329	0.020246	0.015425
								72.93	0.06425	0.020286	0.015464
								73.2025	0.064171	0.020327	0.015502
								73.475	0.064093	0.020367	0.015541
								73.7475	0.064014	0.020407	0.015579
								74.02	0.063936	0.020447	0.015618
								74.29	0.063859	0.020486	0.015656
								74.56	0.063782	0.020525	0.015693
								74.83	0.063705	0.020564	0.015731
								75.1	0.063628	0.020603	0.015769
								75.37	0.063552	0.020642	0.015806
								75.64	0.063476	0.02068	0.015844
								75.91	0.0634	0.020719	0.015881
								76.18	0.063325	0.020757	0.015919
								76.4475	0.06325	0.020795	0.015955
								76.715	0.063176	0.020832	0.015992

								76.9825	0.063101	0.02087	0.016029
								77.25	0.063027	0.020907	0.016066
								77.52	0.062953	0.020945	0.016103
								77.79	0.062879	0.020982	0.016139
								78.06	0.062805	0.021019	0.016176
								78.33	0.062731	0.021056	0.016213
								79.2525	0.062481	0.021181	0.016338
								80.175	0.062234	0.021305	0.016461
								81.0975	0.061989	0.021427	0.016584
								82.02	0.061747	0.021547	0.016706
								84.5275	0.061101	0.021866	0.017033
								87.035	0.060474	0.022173	0.017354
								89.5425	0.059864	0.022468	0.017668
								92.05	0.059271	0.022753	0.017977
								94.5575	0.058694	0.023027	0.01828
								97.065	0.058133	0.02329	0.018577
								99.5725	0.057587	0.023544	0.018869
								102.08	0.057055	0.023789	0.019156
								104.59	0.056537	0.024024	0.019439
								107.1	0.056033	0.024251	0.019716
								109.61	0.055542	0.024469	0.019989
								112.12	0.055063	0.024679	0.020258
								114.628	0.054598	0.024881	0.020521
								117.135	0.054144	0.025075	0.020781
								119.643	0.053702	0.025262	0.021037
								122.15	0.053271	0.025441	0.021288
								124.658	0.05285	0.025614	0.021536
								127.165	0.05244	0.02578	0.02178
								129.673	0.052041	0.025939	0.022021
								132.18	0.05165	0.026092	0.022258
								134.69	0.051269	0.026239	0.022492

								137.2	0.050897	0.02638	0.022722
								139.71	0.050534	0.026516	0.02295
								142.22	0.05018	0.026646	0.023174
								144.727	0.049834	0.026771	0.023395
								147.235	0.049497	0.02689	0.023613
								149.743	0.049167	0.027005	0.023829
								152.25	0.048845	0.027114	0.024041
								154.757	0.04853	0.027219	0.024251
								157.265	0.048222	0.027319	0.024459
								159.773	0.047921	0.027415	0.024663
								162.28	0.047627	0.027507	0.024866
								164.785	0.04734	0.027594	0.025065
								167.29	0.04706	0.027678	0.025263
								169.795	0.046785	0.027757	0.025458
								172.3	0.046516	0.027833	0.025651
								174.808	0.046253	0.027905	0.025842
								177.315	0.045996	0.027974	0.02603
								179.823	0.045744	0.028039	0.026217
								182.33	0.045497	0.028101	0.026402
								184.84	0.045256	0.02816	0.026585
								187.35	0.045019	0.028215	0.026766
								189.86	0.044787	0.028268	0.026945
								192.37	0.044561	0.028317	0.027122
								194.878	0.044339	0.028364	0.027298
								197.385	0.044121	0.028407	0.027471
								199.893	0.043908	0.028448	0.027643
								202.4	0.0437	0.028487	0.027814
								204.908	0.043495	0.028523	0.027982
								207.415	0.043294	0.028556	0.02815
								209.923	0.043098	0.028587	0.028315
								212.43	0.042905	0.028616	0.028479

								214.94	0.042716	0.028642	0.028642
								217.45	0.042531	0.028666	0.028803
								219.96	0.042349	0.028688	0.028963
								222.47	0.04217	0.028708	0.029122
								224.977	0.041995	0.028726	0.029279
								227.485	0.041824	0.028742	0.029434
								229.993	0.041655	0.028756	0.029589
								232.5	0.04149	0.028768	0.029742
								235.007	0.041328	0.028779	0.029894
								237.515	0.041168	0.028787	0.030044
								240.023	0.041012	0.028794	0.030194
								242.53	0.040859	0.0288	0.030342
								245.035	0.040708	0.028803	0.030489
								247.54	0.04056	0.028805	0.030635
								250.045	0.040414	0.028806	0.03078
								252.55	0.040272	0.028805	0.030923
								255.058	0.040131	0.028803	0.031066
								257.565	0.039993	0.028799	0.031208
								260.072	0.039857	0.028795	0.031348
								262.58	0.039724	0.028788	0.031488
								265.09	0.039593	0.028781	0.031627
								267.6	0.039463	0.028772	0.031765
								270.11	0.039336	0.028762	0.031902
								272.62	0.039212	0.028751	0.032038
								275.128	0.039089	0.028739	0.032173
								277.635	0.038968	0.028725	0.032307
								280.142	0.03885	0.028711	0.03244
								282.65	0.038733	0.028695	0.032572
								285.157	0.038618	0.028679	0.032704
								287.665	0.038505	0.028662	0.032834
								290.173	0.038393	0.028643	0.032964

								292.68	0.038283	0.028624	0.033093
								295.19	0.038175	0.028604	0.033221
								297.7	0.038069	0.028583	0.033349
								300.21	0.037964	0.028561	0.033475
								302.72	0.037861	0.028538	0.033601
								305.228	0.037759	0.028515	0.033726
								307.735	0.037659	0.02849	0.033851
								310.243	0.037561	0.028465	0.033974
								312.75	0.037463	0.02844	0.034097
								315.257	0.037368	0.028413	0.034219
								317.765	0.037273	0.028386	0.034341
								320.272	0.03718	0.028358	0.034462
								322.78	0.037088	0.02833	0.034582
								325.285	0.036998	0.028301	0.034701
								327.79	0.036909	0.028271	0.03482
								330.295	0.036821	0.028241	0.034938
								332.8	0.036734	0.02821	0.035056
								335.308	0.036648	0.028179	0.035173
								337.815	0.036564	0.028147	0.035289
								340.322	0.036481	0.028115	0.035405
								342.83	0.036398	0.028082	0.03552
								345.34	0.036317	0.028049	0.035634
								347.85	0.036237	0.028015	0.035748
								350.36	0.036158	0.027981	0.035862
								352.87	0.036079	0.027946	0.035975
								355.378	0.036002	0.027911	0.036087
								357.885	0.035926	0.027875	0.036199
								360.392	0.035851	0.027839	0.03631
								362.9	0.035777	0.027803	0.03642
								365.407	0.035703	0.027766	0.036531
								367.915	0.035631	0.027729	0.03664

								370.423	0.035559	0.027692	0.036749
								372.93	0.035488	0.027654	0.036858
								375.44	0.035418	0.027616	0.036966
								377.95	0.035349	0.027578	0.037073
								380.46	0.03528	0.027539	0.03718
								382.97	0.035213	0.0275	0.037287
								385.478	0.035146	0.027461	0.037393
								387.985	0.03508	0.027422	0.037499
								390.493	0.035015	0.027382	0.037604
								393	0.03495	0.027342	0.037708
								395.507	0.034886	0.027302	0.037813
								398.015	0.034823	0.027261	0.037916
								400.522	0.03476	0.027221	0.038019
								403.03	0.034698	0.02718	0.038122
								405.54	0.034637	0.027139	0.038225
								408.05	0.034576	0.027097	0.038327
								410.56	0.034516	0.027056	0.038428
								413.07	0.034457	0.027014	0.03853
								415.572	0.034398	0.026972	0.03863
								418.075	0.03434	0.02693	0.03873
								420.577	0.034282	0.026888	0.03883
								423.08	0.034225	0.026846	0.038929
								425.59	0.034169	0.026804	0.039028
								428.1	0.034113	0.026761	0.039127
								430.61	0.034057	0.026718	0.039225
								433.12	0.034002	0.026675	0.039323
								435.628	0.033948	0.026632	0.03942
								438.135	0.033894	0.026589	0.039517
								440.642	0.03384	0.026546	0.039614
								443.15	0.033787	0.026503	0.03971
								445.657	0.033735	0.026459	0.039806

								448.165	0.033683	0.026416	0.039901
								450.673	0.033631	0.026372	0.039996
								453.18	0.03358	0.026329	0.040091
								455.69	0.03353	0.026285	0.040186
								458.2	0.033479	0.026241	0.04028
								460.71	0.033429	0.026197	0.040373
								463.22	0.03338	0.026153	0.040467
								465.728	0.033331	0.026109	0.04056
								468.235	0.033282	0.026065	0.040652
								470.743	0.033234	0.026021	0.040745
								473.25	0.033186	0.025977	0.040837
								475.757	0.033139	0.025933	0.040928
								478.265	0.033092	0.025889	0.041019
								480.772	0.033045	0.025845	0.04111
								483.28	0.032999	0.025801	0.041201
								485.79	0.032953	0.025756	0.041291
								488.3	0.032907	0.025712	0.041381
								490.81	0.032861	0.025668	0.041471
								493.32	0.032816	0.025623	0.04156
								495.827	0.032772	0.025579	0.041649
								498.335	0.032727	0.025535	0.041738
								500.843	0.032683	0.025491	0.041826
								503.35	0.03264	0.025446	0.041914
								505.855	0.032596	0.025402	0.042002
								508.36	0.032553	0.025358	0.042089
								510.865	0.032511	0.025314	0.042176
								513.37	0.032468	0.025269	0.042263
								515.878	0.032426	0.025225	0.042349
								518.385	0.032384	0.025181	0.042435
								520.892	0.032342	0.025137	0.042521
								523.4	0.032301	0.025092	0.042607

								525.908	0.03226	0.025048	0.042692
								528.415	0.032219	0.025004	0.042777
								530.922	0.032178	0.02496	0.042862
								533.43	0.032138	0.024916	0.042946
								535.94	0.032098	0.024872	0.04303
								538.45	0.032058	0.024828	0.043114
								540.96	0.032018	0.024784	0.043198
								543.47	0.031979	0.02474	0.043281
								545.977	0.03194	0.024696	0.043364
								548.485	0.031901	0.024652	0.043447
								550.993	0.031862	0.024608	0.043529
								553.5	0.031824	0.024565	0.043612
								556.007	0.031786	0.024521	0.043693
								558.515	0.031748	0.024477	0.043775
								561.023	0.03171	0.024434	0.043856
								563.53	0.031672	0.02439	0.043937
								566.04	0.031635	0.024347	0.044018
								568.55	0.031598	0.024303	0.044099
								571.06	0.031561	0.02426	0.044179
								573.57	0.031524	0.024217	0.044259
								576.078	0.031487	0.024173	0.044339
								578.585	0.031451	0.02413	0.044419
								581.092	0.031415	0.024087	0.044498
								583.6	0.031379	0.024044	0.044577
								586.105	0.031343	0.024001	0.044656
								588.61	0.031308	0.023958	0.044734
								591.115	0.031272	0.023916	0.044812
								593.62	0.031237	0.023873	0.04489
								596.128	0.031202	0.02383	0.044968
								598.635	0.031167	0.023788	0.045045
								601.142	0.031132	0.023745	0.045123

								603.65	0.031098	0.023703	0.0452
								606.158	0.031063	0.02366	0.045276
								608.665	0.031029	0.023618	0.045353
								611.172	0.030995	0.023576	0.045429
								613.68	0.030961	0.023534	0.045505
								616.19	0.030928	0.023492	0.045581
								618.7	0.030894	0.02345	0.045657
								621.21	0.03086	0.023408	0.045732
								623.72	0.030827	0.023366	0.045807
								626.227	0.030794	0.023324	0.045882
								628.735	0.030761	0.023282	0.045957
								631.243	0.030728	0.023241	0.046031
								633.75	0.030696	0.023199	0.046105
								636.257	0.030663	0.023158	0.046179
								638.765	0.030631	0.023117	0.046253
								641.273	0.030598	0.023076	0.046326
								643.78	0.030566	0.023034	0.0464
								646.29	0.030534	0.022993	0.046473
								648.8	0.030502	0.022952	0.046545
								651.31	0.030471	0.022911	0.046618
								653.82	0.030439	0.022871	0.046691
								656.328	0.030408	0.02283	0.046763
								658.835	0.030376	0.022789	0.046835
								661.342	0.030345	0.022749	0.046906
								663.85	0.030314	0.022709	0.046978
								666.358	0.030283	0.022668	0.047049
								668.865	0.030252	0.022628	0.04712
								671.372	0.030221	0.022588	0.047191
								673.88	0.030191	0.022548	0.047261
								676.385	0.03016	0.022508	0.047332
								678.89	0.03013	0.022468	0.047402

								681.395	0.0301	0.022429	0.047472
								683.9	0.03007	0.022389	0.047541
								686.408	0.03004	0.022349	0.047611
								688.915	0.03001	0.02231	0.04768
								691.422	0.02998	0.022271	0.047749
								693.93	0.029951	0.022231	0.047818
								696.44	0.029921	0.022192	0.047887
								698.95	0.029892	0.022153	0.047956
								701.46	0.029862	0.022114	0.048024
								703.97	0.029833	0.022075	0.048092
								706.477	0.029804	0.022036	0.04816
								708.985	0.029775	0.021998	0.048228
								711.493	0.029746	0.021959	0.048295
								714	0.029717	0.021921	0.048362
								716.507	0.029688	0.021882	0.048429
								719.015	0.02966	0.021844	0.048496
								721.523	0.029631	0.021806	0.048563
								724.03	0.029603	0.021768	0.04863
								726.54	0.029575	0.02173	0.048696
								729.05	0.029546	0.021692	0.048762
								731.56	0.029518	0.021654	0.048828
								734.07	0.02949	0.021616	0.048894
								736.578	0.029462	0.021579	0.048959
								739.085	0.029434	0.021541	0.049025
								741.592	0.029407	0.021504	0.04909
								744.1	0.029379	0.021467	0.049155
								746.608	0.029351	0.021429	0.049219
								749.115	0.029324	0.021392	0.049284
								751.622	0.029297	0.021355	0.049348
								754.13	0.029269	0.021318	0.049412
								756.635	0.029242	0.021282	0.049476

								759.14	0.029215	0.021245	0.04954
								761.645	0.029188	0.021208	0.049604
								764.15	0.029161	0.021172	0.049667
								766.658	0.029134	0.021136	0.04973
								769.165	0.029108	0.021099	0.049793
								771.672	0.029081	0.021063	0.049856
								774.18	0.029054	0.021027	0.049919
								776.69	0.029028	0.020991	0.049981
								779.2	0.029001	0.020955	0.050044
								781.71	0.028975	0.020919	0.050106
								784.22	0.028949	0.020883	0.050168
								786.727	0.028923	0.020848	0.05023
								789.235	0.028897	0.020812	0.050291
								791.743	0.028871	0.020777	0.050353
								794.25	0.028845	0.020742	0.050414
								796.757	0.028819	0.020706	0.050475
								799.265	0.028793	0.020671	0.050536
								801.773	0.028767	0.020636	0.050597
								804.28	0.028742	0.020601	0.050657
								806.79	0.028716	0.020566	0.050718
								809.3	0.028691	0.020532	0.050778
								811.81	0.028665	0.020497	0.050838
								814.32	0.02864	0.020463	0.050898
								816.828	0.028615	0.020428	0.050957
								819.335	0.028589	0.020394	0.051017
								821.842	0.028564	0.02036	0.051076
								824.35	0.028539	0.020325	0.051135
								826.858	0.028514	0.020291	0.051194
								829.365	0.02849	0.020257	0.051253
								831.872	0.028465	0.020224	0.051312
								834.38	0.02844	0.02019	0.05137

								836.885	0.028415	0.020156	0.051429
								839.39	0.028391	0.020123	0.051487
								841.895	0.028366	0.020089	0.051545
								844.4	0.028342	0.020056	0.051602
								846.908	0.028318	0.020023	0.05166
								849.415	0.028293	0.01999	0.051717
								851.922	0.028269	0.019956	0.051775
								854.43	0.028245	0.019924	0.051832
								856.94	0.028221	0.019891	0.051889
								859.45	0.028197	0.019858	0.051946
								861.96	0.028173	0.019825	0.052002
								864.47	0.028149	0.019792	0.052059
								866.977	0.028125	0.01976	0.052115
								869.485	0.028101	0.019728	0.052171
								871.993	0.028077	0.019695	0.052227
								874.5	0.028054	0.019663	0.052283
								877.007	0.02803	0.019631	0.052339
								879.515	0.028007	0.019599	0.052395
								882.023	0.027983	0.019567	0.05245
								884.53	0.02796	0.019535	0.052505
								887.04	0.027937	0.019503	0.05256
								889.55	0.027913	0.019472	0.052615
								892.06	0.02789	0.01944	0.05267
								894.57	0.027867	0.019409	0.052725
								897.078	0.027844	0.019377	0.052779
								899.585	0.027821	0.019346	0.052833
								902.092	0.027798	0.019315	0.052887
								904.6	0.027775	0.019284	0.052941
								904.6	0.027775	0.019284	0.052941

Table of ^{1}H NMR integration and COPASI fitted data for **2c** → *trans*-fused- and *cis*-fused-**3c** (*p*-Me) in MeCN

Integral (6.31,6.18)	Integral (5.34,5.25)	Integral (4.49,4.39)	Sum	Time (min)	HA (M)	Trans (M)	Cis (M)	Fitted time (min)	Fitted HA (M)	Fitted trans (M)	Fitted cis (M)
89618.7	1267.67	960.88	91847.25	3.5	0.097574	0.00138	0.001046	0	0.1	0	0
88214.6	1966.94	1572.39	91753.93	5.27	0.096143	0.002144	0.001714	0.875	0.099373	0.000349	0.000277
87624.4	2309.84	1900.05	91834.29	6.35	0.095416	0.002515	0.002069	1.75	0.098754	0.000694	0.000551
86633.8	2729.86	2187.4	91551.06	7.43	0.094629	0.002982	0.002389	2.625	0.098143	0.001034	0.000822
86088.5	3106.14	2444.04	91638.68	8.5	0.093943	0.00339	0.002667	3.5	0.09754	0.00137	0.00109
85590.1	3441.79	2801.55	91833.44	9.58	0.093201	0.003748	0.003051	3.9425	0.097238	0.001538	0.001224
84449.8	3813.94	3099.46	91363.2	10.67	0.092433	0.004174	0.003392	4.385	0.096938	0.001704	0.001358
83826	4209.23	3395.25	91430.48	11.75	0.091683	0.004604	0.003713	4.8275	0.096639	0.00187	0.001491
83152	4551.82	3683.21	91387.03	12.83	0.090989	0.004981	0.00403	5.27	0.096343	0.002035	0.001623
82364.4	4867.33	3980.93	91212.66	13.9	0.090299	0.005336	0.004364	5.54	0.096163	0.002135	0.001703
81937.6	5218.44	4216.9	91372.94	14.98	0.089674	0.005711	0.004615	5.81	0.095983	0.002234	0.001783
81243.1	5515.01	4418.58	91176.69	16.07	0.089105	0.006049	0.004846	6.08	0.095805	0.002333	0.001862
80937.4	5835.66	4798.04	91571.1	17.15	0.088387	0.006373	0.00524	6.35	0.095627	0.002432	0.001942
79814.1	6134.95	4956.19	90905.24	18.23	0.087799	0.006749	0.005452	6.62	0.095449	0.00253	0.002021
79252.1	6470.49	5252.09	90974.68	19.3	0.087114	0.007112	0.005773	6.89	0.095273	0.002628	0.002099
78651.4	6703.89	5421.79	90777.08	20.38	0.086642	0.007385	0.005973	7.16	0.095097	0.002725	0.002178
78164.2	7011.05	5701.99	90877.24	21.47	0.086011	0.007715	0.006274	7.43	0.094921	0.002822	0.002256
77385.3	7315.95	5889.59	90590.84	22.55	0.085423	0.008076	0.006501	7.6975	0.094748	0.002918	0.002333
76848.7	7629.52	6235.41	90713.63	23.63	0.08471	0.008411	0.006874	7.965	0.094576	0.003014	0.00241

					6						
76194.8	7870.23	6418.43	90483.46	24.7	0.084209	0.008698	0.007093	8.2325	0.094404	0.003109	0.002487
75884.1	8112.37	6626.72	90623.19	25.78	0.083736	0.008952	0.007312	8.5	0.094233	0.003203	0.002564
74992.9	8440.27	6847.54	90280.71	26.87	0.083066	0.009349	0.007585	8.77	0.094061	0.003298	0.00264
74574.5	8678.23	7132.16	90384.89	27.95	0.082508	0.009601	0.007891	9.04	0.09389	0.003393	0.002717
74052	8879.04	7242.49	90173.53	29.03	0.082122	0.009847	0.008032	9.31	0.093719	0.003487	0.002793
73806.7	9178.03	7445.86	90430.59	30.1	0.081617	0.010149	0.008234	9.58	0.093549	0.003581	0.00287
73185.9	9360.85	7617.4	90164.15	31.18	0.08117	0.010382	0.008448	9.8525	0.093378	0.003676	0.002946
72614.1	9584.78	8021.49	90220.37	32.27	0.080485	0.010624	0.008891	10.125	0.093208	0.00377	0.003022
71944.5	9965.62	8145.34	90055.46	33.35	0.079889	0.011066	0.009045	10.3975	0.093038	0.003864	0.003099
71359.3	10141.9	8236.5	89737.7	34.42	0.07952	0.011302	0.009178	10.67	0.092869	0.003957	0.003174
71022.4	10311.2	8388.01	89721.61	35.5	0.079159	0.011492	0.009349	10.94	0.092702	0.004049	0.003249
70509.3	10504.1	8641.57	89654.97	36.58	0.078645	0.011716	0.009639	11.21	0.092536	0.004141	0.003324
70414.3	10710.8	8877.6	90002.7	37.67	0.078236	0.011901	0.009864	11.48	0.09237	0.004232	0.003398
69647.6	10984.9	9055.93	89688.43	38.75	0.077655	0.012248	0.010097	11.75	0.092205	0.004323	0.003472
69255.1	11182.7	9341.08	89778.88	39.82	0.07714	0.012456	0.010405	12.02	0.09204	0.004414	0.003546
68863.9	11353.1	9517.04	89734.04	40.9	0.076742	0.012652	0.010606	12.29	0.091877	0.004504	0.00362
68486.3	11578.5	9472.7	89537.5	41.98	0.076489	0.012931	0.01058	12.56	0.091713	0.004594	0.003693
68102.2	11752.2	9859.84	89714.24	43.07	0.07591	0.0131	0.01099	12.83	0.091551	0.004683	0.003766
67324	11989.6	10027.7	89341.3	44.15	0.075356	0.01342	0.011224	13.0975	0.09139	0.004772	0.003838
66968.8	12168.2	10094	89231	45.22	0.075051	0.013637	0.011312	13.365	0.09123	0.00486	0.00391
66494.2	12327.4	10332	89153.6	46.3	0.074584	0.013827	0.011589	13.6325	0.091071	0.004947	0.003982
65996.3	12374.6	10432.5	88803.4	47.38	0.07431	0.013935	0.011748	13.9	0.090912	0.005035	0.004054

					7						
65683.6	12667.1	10636.8	88987.5	48.47	0.073812	0.014235	0.011953	14.17	0.090752	0.005122	0.004125
65206.1	12803.8	10717.3	88727.2	49.55	0.073491	0.014431	0.012079	14.44	0.090593	0.00521	0.004197
64896.1	12981.5	11135.9	89013.5	50.62	0.072906	0.014584	0.01251	14.71	0.090435	0.005297	0.004269
64557	13201.3	11027.5	88785.8	51.7	0.072711	0.014869	0.01242	14.98	0.090277	0.005384	0.00434
64076.1	13284	11455.5	88815.6	52.78	0.072145	0.014957	0.012898	15.2525	0.090118	0.005471	0.004411
63475.8	13462.8	11574.9	88513.5	53.87	0.071713	0.01521	0.013077	15.525	0.089959	0.005558	0.004483
63513.9	13683.7	11782.3	88979.9	54.95	0.07138	0.015378	0.013242	15.7975	0.089802	0.005644	0.004554
63396.1	13896.5	11907.6	89200.2	56.02	0.071072	0.015579	0.013349	16.07	0.089645	0.00573	0.004625
62723.1	13977.9	11950	88651	57.1	0.070753	0.015767	0.01348	16.34	0.08949	0.005815	0.004695
62302.9	14194.2	12191.9	88689	58.18	0.070249	0.016004	0.013747	16.61	0.089335	0.0059	0.004765
62235.5	14411	12327.6	88974.1	59.27	0.069948	0.016197	0.013855	16.88	0.089181	0.005984	0.004835
61825	14588.7	12240.5	88654.2	60.35	0.069737	0.016456	0.013807	17.15	0.089028	0.006069	0.004904
61577.2	14702.2	12586.5	88865.9	61.42	0.069292	0.016544	0.014163	17.42	0.088875	0.006152	0.004973
61110.2	14843.4	12802.3	88755.9	62.5	0.068852	0.016724	0.014424	17.69	0.088722	0.006236	0.005042
60867.3	14926.9	13014.8	88809	63.58	0.068537	0.016808	0.014655	17.96	0.088571	0.006319	0.005111
60487.9	15116.6	13112.2	88716.7	64.67	0.068181	0.017039	0.01478	18.23	0.088419	0.006401	0.005179
60062.4	15335.6	13245.1	88643.1	65.75	0.067758	0.0173	0.014942	18.4975	0.08827	0.006483	0.005247
59717.9	15310.6	13335.5	88364	66.82	0.067582	0.017327	0.015092	18.765	0.088121	0.006564	0.005314
59463.8	15483	13459.3	88406.1	67.9	0.067262	0.017513	0.015224	19.0325	0.087973	0.006645	0.005382
58915.1	15626.5	13637.1	88178.7	68.98	0.066813	0.017721	0.015465	19.3	0.087825	0.006726	0.005449
58532	15817.6	13823.7	88173.3	71.05	0.06638	0.017939	0.015678	19.57	0.087677	0.006807	0.005516

					3						
56150.9	16871	15025	88046.9	81.08	0.063774	0.019161	0.017065	19.84	0.087529	0.006888	0.005583
53812.5	17730.2	16207	87749.7	91.1	0.061325	0.020205	0.01847	20.11	0.087381	0.006969	0.00565
51954	18685.5	17219.8	87859.3	101.13	0.059133	0.021268	0.019599	20.38	0.087234	0.007049	0.005717
50260	19706.6	18321.9	88288.5	111.17	0.056927	0.022321	0.020752	20.6525	0.087086	0.007129	0.005784
49173.5	20293.4	19227.4	88694.3	121.2	0.055442	0.02288	0.021678	20.925	0.086939	0.00721	0.005851
47310.3	20919.7	19829.9	88059.9	131.23	0.053725	0.023756	0.022519	21.1975	0.086792	0.00729	0.005918
45952.5	21243.5	20624.1	87820.1	141.27	0.052326	0.02419	0.023484	21.47	0.086646	0.00737	0.005985
44843.9	21771.9	21344.9	87960.7	151.3	0.050982	0.024752	0.024266	21.74	0.086501	0.007448	0.00605
43891.2	22259.3	22132.8	88283.3	161.33	0.049716	0.025213	0.02507	22.01	0.086357	0.007527	0.006116
42778.4	22873	22836.7	88488.1	171.35	0.048344	0.025849	0.025808	22.28	0.086214	0.007605	0.006181
41335.8	22914.7	23427	87677.5	181.38	0.047145	0.026135	0.02672	22.55	0.086071	0.007682	0.006247
40423.3	23137.1	24047.8	87608.2	191.42	0.046141	0.02641	0.027449	22.82	0.085929	0.00776	0.006311
39672.4	23290.8	24498.7	87461.9	201.45	0.04536	0.02663	0.028011	23.09	0.085787	0.007837	0.006376
38841.9	23587.1	25123.5	87552.5	211.48	0.044364	0.026941	0.028695	23.36	0.085645	0.007914	0.006441
38085.9	23678.5	25557.6	87322	221.52	0.043615	0.027116	0.029268	23.63	0.085504	0.00799	0.006505
37109.5	23802.4	25908.1	86820	231.55	0.042743	0.027416	0.029841	23.8975	0.085365	0.008066	0.006569
36385.2	23998.9	26443.4	86827.5	241.58	0.041905	0.02764	0.030455	24.165	0.085227	0.008141	0.006632
36027.6	23782.8	26687	86497.4	251.6	0.041652	0.027495	0.030853	24.4325	0.085088	0.008216	0.006695
35177.1	24181.8	27330.5	86689.4	261.63	0.040578	0.027895	0.031527	24.7	0.084951	0.008291	0.006758
34682.4	24002.2	27815	86499.6	271.67	0.040095	0.027748	0.032156	24.97	0.084812	0.008366	0.006822
34170.7	24203.2	28221.2	86595.1	281.7	0.03946	0.02795	0.03259	25.24	0.084674	0.008441	0.006885

33660.7	24186.9	28687.2	86534.8	291.73	0.038898	0.02795	0.033151	25.51	0.084537	0.008516	0.006948
33220.6	24216.7	29207.3	86644.6	301.77	0.038341	0.027949	0.033709	25.78	0.0844	0.00859	0.007011
32874.5	24500.2	29721.9	87096.6	311.8	0.037745	0.02813	0.034125	26.0525	0.084262	0.008665	0.007074
32758.8	24696.4	30161.8	87617	321.83	0.037389	0.028187	0.034425	26.325	0.084124	0.008739	0.007137
32755.8	24549.9	30648.6	87954.3	331.87	0.037242	0.027912	0.034846	26.5975	0.083987	0.008813	0.0072
31938.3	24774.8	30988.7	87701.8	341.88	0.036417	0.028249	0.035334	26.87	0.083851	0.008887	0.007262
31525.8	24464.4	31286	87276.2	351.92	0.036122	0.028031	0.035847	27.14	0.083716	0.00896	0.007324
31059	24281.8	31591.4	86932.2	361.95	0.035728	0.027932	0.03634	27.41	0.083582	0.009032	0.007386
30575	24452.1	31819.2	86846.3	371.98	0.035206	0.028156	0.036639	27.68	0.083448	0.009105	0.007447
30908.8	24833.9	32894.2	88636.9	382.02	0.034871	0.028018	0.037111	27.95	0.083315	0.009177	0.007509
30450.2	24939.1	33136.1	88525.4	392.05	0.034397	0.028172	0.037431	28.22	0.083182	0.009249	0.00757
30068.4	24577.3	33402.6	88048.3	402.08	0.03415	0.027913	0.037937	28.49	0.083049	0.00932	0.007631
29823.6	24796	33883.5	88503.1	412.12	0.033698	0.028017	0.038285	28.76	0.082917	0.009391	0.007691
29675.4	24481.3	34278.4	88435.1	422.13	0.033556	0.027683	0.038761	29.03	0.082786	0.009462	0.007752
29217.7	24476.3	34517.7	88211.7	432.17	0.033122	0.027747	0.039131	29.2975	0.082656	0.009532	0.007812
29063.8	24607	34988.1	88658.9	442.2	0.032782	0.027755	0.039464	29.565	0.082526	0.009602	0.007871
29012	24323.6	35240.8	88576.4	452.23	0.032754	0.027461	0.039786	29.8325	0.082397	0.009672	0.007931
28715.4	24589.5	35750	89054.9	462.27	0.032245	0.027612	0.040144	30.1	0.082269	0.009741	0.00799
28486	24384.3	35957.2	88827.5	472.3	0.032069	0.027451	0.04048	30.37	0.082139	0.009811	0.00805
28464	24637.3	36564.7	89666	482.33	0.031744	0.027477	0.040779	30.64	0.08201	0.00988	0.00811
28176.8	24414.4	36726.4	89317.6	492.37	0.031547	0.027334	0.041119	30.91	0.081882	0.009949	0.008169

27973.4	24326.8	37102.5	89402.7	502.38	0.031289	0.02721	0.0415	31.18	0.081754	0.010018	0.008228
27461.7	24130.3	36993.8	88585.8	512.42	0.031	0.027239	0.04176	31.4525	0.081625	0.010087	0.008288
27497.3	24086.9	37272	88856.2	522.45	0.030946	0.027108	0.041946	31.725	0.081497	0.010156	0.008347
27139.4	23730.1	37550.9	88420.4	532.48	0.030694	0.026838	0.042469	31.9975	0.081369	0.010225	0.008406
27000.3	23617.4	37914.4	88532.1	542.52	0.030498	0.026677	0.042826	32.27	0.081241	0.010294	0.008465
26920.7	23810.1	38332.9	89063.7	552.55	0.030226	0.026734	0.04304	32.54	0.081115	0.010361	0.008524
26620.6	23834.4	38368.7	88823.7	562.58	0.02997	0.026833	0.043196	32.81	0.08099	0.010429	0.008582
26404.9	23671.4	38432.4	88508.7	572.62	0.029833	0.026745	0.043422	33.08	0.080865	0.010496	0.00864
26286.3	23581.5	38881.7	88749.5	582.63	0.029619	0.026571	0.043811	33.35	0.08074	0.010563	0.008697
26181.2	23109.2	38919.6	88210	592.67	0.029681	0.026198	0.044122	33.6175	0.080617	0.010629	0.008755
25955.9	23276	39202.4	88434.3	602.7	0.02935	0.02632	0.044329	33.885	0.080494	0.010694	0.008812
25677.3	23260.2	39687.5	88625	612.73	0.028973	0.026246	0.044781	34.1525	0.080372	0.01076	0.008868
25665.1	23168.8	39727.2	88561.1	622.77	0.02898	0.026161	0.044859	34.42	0.08025	0.010825	0.008925
25604.8	23106.7	40396.2	89107.7	632.8	0.028735	0.025931	0.045334	34.69	0.080127	0.010891	0.008982
25538.3	22869.1	40402.4	88809.8	642.83	0.028756	0.025751	0.045493	34.96	0.080005	0.010956	0.009039
25181.6	22941.6	40512.4	88635.6	652.87	0.02841	0.025883	0.045707	35.23	0.079883	0.011021	0.009095
25420.5	23068.9	41630	90119.4	662.88	0.028208	0.025598	0.046194	35.5	0.079762	0.011086	0.009152
25327.2	22872.7	41677.5	89877.4	672.92	0.02818	0.025449	0.046372	35.77	0.079641	0.011151	0.009208
25161.4	22573.4	42013	89747.8	682.95	0.028036	0.025152	0.046812	36.04	0.07952	0.011215	0.009265
25602.3	23468.5	43107.6	92178.4	692.98	0.027775	0.02546	0.046765	36.31	0.0794	0.01128	0.009321
25498.4	22999.6	43582.4	92080.4	703.02	0.027691	0.024978	0.047331	36.58	0.07928	0.011344	0.009376
25209.4	23112.2	43433.8	91755.4	713.05	0.027475	0.025189	0.047337	36.8525	0.07916	0.011408	0.009433
25309.5	23046.3	43690.8	92046.6	723.08	0.02749	0.025038	0.047466	37.125	0.079039	0.011472	0.009489

					6						
25125.6	22931.6	44011.3	92068.5	733.12	0.02729	0.024907	0.047803	37.3975	0.07892	0.011536	0.009545
25092.1	23009.4	44420.7	92522.2	743.13	0.02712	0.024869	0.048011	37.67	0.0788	0.011599	0.0096
24980.7	22935.7	44725	92641.4	753.17	0.026965	0.024758	0.048278	37.94	0.078682	0.011662	0.009656
24945.2	22625.9	45037.3	92608.4	763.2	0.026936	0.024432	0.048632	38.21	0.078565	0.011725	0.00971
24782.2	22477.5	44991.4	92251.1	773.23	0.026864	0.024366	0.048771	38.48	0.078448	0.011787	0.009765
24516.9	22208.9	45132.7	91858.5	783.27	0.02669	0.024177	0.049133	38.75	0.078331	0.011849	0.00982
24323.7	22451	45247	92021.7	793.3	0.026433	0.024398	0.04917	39.0175	0.078216	0.011911	0.009874
24326.7	22262.7	45605.4	92194.8	803.33	0.026386	0.024147	0.049466	39.285	0.078101	0.011972	0.009928
24310.7	22392.8	46003.4	92706.9	813.37	0.026223	0.024154	0.049622	39.5525	0.077986	0.012032	0.009981
24295.4	22282.9	46322.2	92900.5	823.38	0.026152	0.023986	0.049862	39.82	0.077872	0.012093	0.010035
24198.3	22237.9	46412.3	92848.5	833.42	0.026062	0.023951	0.049987	40.09	0.077757	0.012154	0.010089
23815.2	21983.6	46633.2	92432	843.45	0.025765	0.023784	0.050451	40.36	0.077643	0.012215	0.010143
24003.3	21719.8	46919.2	92642.3	853.48	0.02591	0.023445	0.050646	40.63	0.077529	0.012275	0.010196
23611.4	21951.4	47087	92649.8	863.52	0.025485	0.023693	0.050823	40.9	0.077415	0.012336	0.01025
23712.8	21518.7	47324.2	92555.7	873.55	0.02562	0.023249	0.051131	41.17	0.077301	0.012396	0.010303
								41.44	0.077188	0.012456	0.010356
								41.71	0.077076	0.012515	0.010409
								41.98	0.076963	0.012575	0.010462
								42.2525	0.07685	0.012635	0.010515
								42.525	0.076738	0.012694	0.010568
								42.7975	0.076625	0.012754	0.010621
								43.07	0.076513	0.012813	0.010674
								43.34	0.076403	0.012871	0.010726
								43.61	0.076293	0.012929	0.010778
								43.88	0.076183	0.012987	0.01083

								44.15	0.076073	0.013045	0.010882
								44.4175	0.075965	0.013102	0.010933
								44.685	0.075857	0.013159	0.010984
								44.9525	0.07575	0.013216	0.011035
								45.22	0.075643	0.013272	0.011085
								45.49	0.075535	0.013329	0.011136
								45.76	0.075428	0.013385	0.011187
								46.03	0.075321	0.013442	0.011238
								46.3	0.075214	0.013498	0.011289
								46.57	0.075107	0.013554	0.011339
								46.84	0.075001	0.013609	0.01139
								47.11	0.074895	0.013665	0.01144
								47.38	0.07479	0.01372	0.01149
								47.6525	0.074684	0.013776	0.01154
								47.925	0.074578	0.013831	0.011591
								48.1975	0.074473	0.013887	0.011641
								48.47	0.074368	0.013942	0.011691
								48.74	0.074264	0.013996	0.01174
								49.01	0.07416	0.01405	0.01179
								49.28	0.074057	0.014104	0.011839
								49.55	0.073954	0.014158	0.011888
								49.8175	0.073853	0.014211	0.011936
								50.085	0.073752	0.014264	0.011985
								50.3525	0.073651	0.014317	0.012033
								50.62	0.07355	0.014369	0.012081
								50.89	0.073449	0.014422	0.012129
								51.16	0.073348	0.014475	0.012178
								51.43	0.073247	0.014527	0.012226
								51.7	0.073147	0.014579	0.012274
								51.97	0.073047	0.014631	0.012322
								52.24	0.072947	0.014683	0.01237

								52.51	0.072848	0.014735	0.012417
								52.78	0.072748	0.014787	0.012465
								53.0525	0.072649	0.014839	0.012513
								53.325	0.072549	0.01489	0.012561
								53.5975	0.07245	0.014942	0.012608
								53.87	0.072351	0.014993	0.012656
								54.14	0.072254	0.015044	0.012703
								54.41	0.072156	0.015094	0.01275
								54.68	0.072059	0.015144	0.012796
								54.95	0.071963	0.015194	0.012843
								55.2175	0.071867	0.015244	0.012889
								55.485	0.071772	0.015293	0.012935
								55.7525	0.071677	0.015342	0.012981
								56.02	0.071582	0.015391	0.013027
								56.29	0.071487	0.01544	0.013073
								56.56	0.071392	0.01549	0.013119
								56.83	0.071297	0.015538	0.013165
								57.1	0.071203	0.015587	0.01321
								57.37	0.071109	0.015636	0.013256
								57.64	0.071015	0.015684	0.013301
								57.91	0.070921	0.015732	0.013347
								58.18	0.070828	0.01578	0.013392
								58.4525	0.070734	0.015829	0.013437
								58.725	0.07064	0.015877	0.013483
								58.9975	0.070547	0.015925	0.013528
								59.27	0.070454	0.015973	0.013573
								59.54	0.070362	0.01602	0.013618
								59.81	0.07027	0.016067	0.013663
								60.08	0.070179	0.016114	0.013707
								60.35	0.070088	0.016161	0.013752
								60.6175	0.069998	0.016207	0.013795

								60.885	0.069908	0.016253	0.013839
								61.1525	0.069819	0.016298	0.013883
								61.42	0.06973	0.016344	0.013926
								61.69	0.06964	0.01639	0.01397
								61.96	0.06955	0.016436	0.014014
								62.23	0.069461	0.016481	0.014058
								62.5	0.069372	0.016527	0.014101
								62.77	0.069283	0.016572	0.014145
								63.04	0.069195	0.016617	0.014188
								63.31	0.069107	0.016662	0.014231
								63.58	0.069019	0.016707	0.014274
								63.8525	0.06893	0.016752	0.014318
								64.125	0.068842	0.016797	0.014361
								64.3975	0.068754	0.016842	0.014404
								64.67	0.068666	0.016886	0.014447
								64.94	0.06858	0.016931	0.01449
								65.21	0.068493	0.016974	0.014532
								65.48	0.068407	0.017018	0.014575
								65.75	0.068321	0.017062	0.014617
								66.0175	0.068236	0.017105	0.014659
								66.285	0.068152	0.017148	0.014701
								66.5525	0.068067	0.01719	0.014742
								66.82	0.067983	0.017233	0.014784
								67.09	0.067899	0.017276	0.014826
								67.36	0.067814	0.017319	0.014867
								67.63	0.06773	0.017361	0.014909
								67.9	0.067646	0.017404	0.01495
								68.17	0.067562	0.017446	0.014992
								68.44	0.067479	0.017488	0.015033
								68.71	0.067396	0.01753	0.015074
								68.98	0.067313	0.017572	0.015115

								69.4975	0.067154	0.017652	0.015194
								70.015	0.066997	0.017731	0.015272
								70.5325	0.06684	0.01781	0.01535
								71.05	0.066684	0.017888	0.015428
								73.5575	0.065941	0.01826	0.015799
								76.065	0.065218	0.01862	0.016162
								78.5725	0.064512	0.01897	0.016518
								81.08	0.063825	0.019308	0.016867
								83.585	0.063156	0.019636	0.017208
								86.09	0.062503	0.019954	0.017543
								88.595	0.061867	0.020262	0.017872
								91.1	0.061246	0.02056	0.018194
								93.6075	0.06064	0.02085	0.018511
								96.115	0.060048	0.021131	0.018821
								98.6225	0.059471	0.021403	0.019126
								101.13	0.058907	0.021667	0.019426
								103.64	0.058356	0.021924	0.01972
								106.15	0.057818	0.022172	0.02001
								108.66	0.057293	0.022413	0.020294
								111.17	0.05678	0.022647	0.020574
								113.678	0.056279	0.022873	0.020848
								116.185	0.055789	0.023093	0.021118
								118.693	0.055311	0.023306	0.021384
								121.2	0.054843	0.023512	0.021645
								123.708	0.054386	0.023712	0.021902
								126.215	0.053939	0.023906	0.022155
								128.723	0.053501	0.024094	0.022404
								131.23	0.053074	0.024277	0.02265
								133.74	0.052655	0.024454	0.022892
								136.25	0.052245	0.024625	0.02313
								138.76	0.051844	0.024792	0.023364

								141.27	0.051451	0.024953	0.023596
								143.778	0.051068	0.025109	0.023823
								146.285	0.050692	0.02526	0.024048
								148.793	0.050324	0.025407	0.024269
								151.3	0.049964	0.025549	0.024488
								153.808	0.049611	0.025687	0.024703
								156.315	0.049265	0.02582	0.024915
								158.823	0.048926	0.025949	0.025125
								161.33	0.048595	0.026074	0.025331
								163.835	0.04827	0.026195	0.025535
								166.34	0.047952	0.026312	0.025736
								168.845	0.04764	0.026425	0.025935
								171.35	0.047334	0.026535	0.026131
								173.857	0.047034	0.026641	0.026325
								176.365	0.04674	0.026744	0.026517
								178.873	0.046451	0.026843	0.026706
								181.38	0.046168	0.026939	0.026893
								183.89	0.04589	0.027032	0.027078
								186.4	0.045618	0.027122	0.02726
								188.91	0.045351	0.027209	0.027441
								191.42	0.045088	0.027293	0.027619
								193.927	0.044831	0.027373	0.027796
								196.435	0.044579	0.027451	0.02797
								198.943	0.044331	0.027526	0.028142
								201.45	0.044088	0.027599	0.028313
								203.957	0.04385	0.027669	0.028481
								206.465	0.043616	0.027736	0.028648
								208.973	0.043386	0.027801	0.028813
								211.48	0.04316	0.027863	0.028977
								213.99	0.042938	0.027923	0.029139
								216.5	0.04272	0.027981	0.029299

								219.01	0.042506	0.028037	0.029457
								221.52	0.042296	0.02809	0.029614
								224.028	0.042089	0.028141	0.02977
								226.535	0.041887	0.02819	0.029923
								229.043	0.041687	0.028237	0.030075
								231.55	0.041492	0.028282	0.030226
								234.058	0.041299	0.028325	0.030376
								236.565	0.04111	0.028366	0.030524
								239.073	0.040924	0.028406	0.03067
								241.58	0.040742	0.028443	0.030815
								244.085	0.040562	0.028479	0.030959
								246.59	0.040386	0.028513	0.031102
								249.095	0.040212	0.028545	0.031243
								251.6	0.040042	0.028576	0.031383
								254.107	0.039874	0.028605	0.031522
								256.615	0.039708	0.028632	0.031659
								259.123	0.039546	0.028658	0.031796
								261.63	0.039386	0.028683	0.031931
								264.14	0.039229	0.028706	0.032065
								266.65	0.039074	0.028728	0.032199
								269.16	0.038922	0.028748	0.032331
								271.67	0.038772	0.028767	0.032462
								274.178	0.038624	0.028784	0.032592
								276.685	0.038479	0.0288	0.032721
								279.193	0.038336	0.028815	0.032848
								281.7	0.038196	0.028829	0.032975
								284.207	0.038057	0.028842	0.033101
								286.715	0.037921	0.028853	0.033226
								289.223	0.037787	0.028863	0.03335
								291.73	0.037655	0.028872	0.033473
								294.24	0.037525	0.02888	0.033595

								296.75	0.037396	0.028887	0.033717
								299.26	0.03727	0.028893	0.033837
								301.77	0.037146	0.028898	0.033957
								304.277	0.037023	0.028902	0.034075
								306.785	0.036903	0.028904	0.034193
								309.293	0.036784	0.028906	0.03431
								311.8	0.036667	0.028907	0.034426
								314.308	0.036552	0.028907	0.034541
								316.815	0.036438	0.028906	0.034656
								319.322	0.036326	0.028905	0.03477
								321.83	0.036215	0.028902	0.034883
								324.34	0.036107	0.028899	0.034995
								326.85	0.035999	0.028894	0.035107
								329.36	0.035893	0.028889	0.035218
								331.87	0.035789	0.028883	0.035328
								334.373	0.035686	0.028877	0.035437
								336.875	0.035585	0.02887	0.035545
								339.378	0.035485	0.028861	0.035653
								341.88	0.035387	0.028853	0.03576
								344.39	0.03529	0.028843	0.035867
								346.9	0.035194	0.028833	0.035973
								349.41	0.035099	0.028822	0.036079
								351.92	0.035006	0.028811	0.036184
								354.428	0.034914	0.028799	0.036288
								356.935	0.034823	0.028786	0.036391
								359.443	0.034733	0.028773	0.036494
								361.95	0.034645	0.028759	0.036596
								364.457	0.034558	0.028745	0.036698
								366.965	0.034471	0.02873	0.036799
								369.473	0.034386	0.028714	0.0369
								371.98	0.034302	0.028698	0.037

								374.49	0.03422	0.028681	0.037099
								377	0.034138	0.028664	0.037198
								379.51	0.034057	0.028647	0.037297
								382.02	0.033977	0.028628	0.037395
								384.527	0.033898	0.02861	0.037492
								387.035	0.033821	0.028591	0.037589
								389.543	0.033744	0.028571	0.037685
								392.05	0.033668	0.028551	0.03778
								394.558	0.033593	0.028531	0.037876
								397.065	0.033519	0.02851	0.03797
								399.572	0.033446	0.028489	0.038065
								402.08	0.033374	0.028467	0.038158
								404.59	0.033303	0.028445	0.038252
								407.1	0.033232	0.028423	0.038345
								409.61	0.033163	0.0284	0.038437
								412.12	0.033094	0.028377	0.038529
								414.623	0.033026	0.028354	0.03862
								417.125	0.032959	0.02833	0.038711
								419.628	0.032893	0.028306	0.038802
								422.13	0.032827	0.028281	0.038891
								424.64	0.032762	0.028257	0.038981
								427.15	0.032698	0.028231	0.039071
								429.66	0.032635	0.028206	0.039159
								432.17	0.032572	0.02818	0.039248
								434.678	0.03251	0.028154	0.039336
								437.185	0.032449	0.028128	0.039424
								439.693	0.032388	0.028101	0.039511
								442.2	0.032328	0.028075	0.039598
								444.707	0.032269	0.028048	0.039684
								447.215	0.03221	0.02802	0.03977
								449.723	0.032152	0.027993	0.039856

								452.23	0.032095	0.027965	0.039941
								454.74	0.032038	0.027937	0.040026
								457.25	0.031981	0.027908	0.04011
								459.76	0.031926	0.02788	0.040195
								462.27	0.031871	0.027851	0.040278
								464.777	0.031816	0.027822	0.040362
								467.285	0.031762	0.027793	0.040445
								469.793	0.031709	0.027764	0.040528
								472.3	0.031656	0.027734	0.04061
								474.808	0.031604	0.027704	0.040692
								477.315	0.031552	0.027674	0.040774
								479.822	0.031501	0.027644	0.040855
								482.33	0.03145	0.027614	0.040936
								484.84	0.0314	0.027583	0.041017
								487.35	0.03135	0.027553	0.041097
								489.86	0.031301	0.027522	0.041177
								492.37	0.031252	0.027491	0.041257
								494.873	0.031204	0.02746	0.041336
								497.375	0.031156	0.027429	0.041415
								499.878	0.031109	0.027398	0.041493
								502.38	0.031062	0.027366	0.041572
								504.89	0.031016	0.027334	0.04165
								507.4	0.03097	0.027303	0.041728
								509.91	0.030924	0.027271	0.041805
								512.42	0.030879	0.027239	0.041883
								514.928	0.030834	0.027207	0.04196
								517.435	0.03079	0.027174	0.042036
								519.943	0.030746	0.027142	0.042112
								522.45	0.030702	0.02711	0.042188
								524.957	0.030659	0.027077	0.042264
								527.465	0.030616	0.027044	0.04234

								529.973	0.030574	0.027012	0.042415
								532.48	0.030531	0.026979	0.04249
								534.99	0.03049	0.026946	0.042565
								537.5	0.030448	0.026913	0.042639
								540.01	0.030407	0.02688	0.042713
								542.52	0.030367	0.026847	0.042787
								545.027	0.030326	0.026813	0.042861
								547.535	0.030286	0.02678	0.042934
								550.043	0.030247	0.026747	0.043007
								552.55	0.030207	0.026713	0.04308
								555.058	0.030168	0.02668	0.043152
								557.565	0.03013	0.026646	0.043224
								560.072	0.030091	0.026613	0.043296
								562.58	0.030053	0.026579	0.043368
								565.09	0.030015	0.026545	0.04344
								567.6	0.029978	0.026511	0.043511
								570.11	0.029941	0.026477	0.043582
								572.62	0.029904	0.026444	0.043653
								575.122	0.029867	0.02641	0.043723
								577.625	0.029831	0.026376	0.043793
								580.128	0.029795	0.026342	0.043863
								582.63	0.029759	0.026308	0.043933
								585.14	0.029724	0.026274	0.044003
								587.65	0.029688	0.02624	0.044072
								590.16	0.029653	0.026206	0.044141
								592.67	0.029619	0.026171	0.04421
								595.178	0.029584	0.026137	0.044279
								597.685	0.02955	0.026103	0.044347
								600.193	0.029516	0.026069	0.044415
								602.7	0.029482	0.026035	0.044483
								605.207	0.029449	0.026	0.044551

								607.715	0.029415	0.025966	0.044619
								610.223	0.029382	0.025932	0.044686
								612.73	0.029349	0.025898	0.044753
								615.24	0.029317	0.025863	0.04482
								617.75	0.029284	0.025829	0.044887
								620.26	0.029252	0.025795	0.044953
								622.77	0.02922	0.02576	0.04502
								625.277	0.029189	0.025726	0.045086
								627.785	0.029157	0.025692	0.045151
								630.293	0.029126	0.025657	0.045217
								632.8	0.029095	0.025623	0.045282
								635.308	0.029064	0.025589	0.045348
								637.815	0.029033	0.025554	0.045413
								640.322	0.029003	0.02552	0.045477
								642.83	0.028972	0.025486	0.045542
								645.34	0.028942	0.025451	0.045607
								647.85	0.028912	0.025417	0.045671
								650.36	0.028882	0.025383	0.045735
								652.87	0.028853	0.025349	0.045799
								655.372	0.028824	0.025314	0.045862
								657.875	0.028794	0.02528	0.045926
								660.378	0.028765	0.025246	0.045989
								662.88	0.028737	0.025212	0.046052
								665.39	0.028708	0.025178	0.046114
								667.9	0.028679	0.025144	0.046177
								670.41	0.028651	0.025109	0.04624
								672.92	0.028623	0.025075	0.046302
								675.428	0.028595	0.025041	0.046364
								677.935	0.028567	0.025007	0.046426
								680.443	0.028539	0.024973	0.046488
								682.95	0.028512	0.024939	0.046549

								685.457	0.028484	0.024905	0.046611
								687.965	0.028457	0.024871	0.046672
								690.473	0.02843	0.024837	0.046733
								692.98	0.028403	0.024803	0.046794
								695.49	0.028376	0.024769	0.046854
								698	0.02835	0.024736	0.046915
								700.51	0.028323	0.024702	0.046975
								703.02	0.028297	0.024668	0.047036
								705.527	0.028271	0.024634	0.047095
								708.035	0.028244	0.0246	0.047155
								710.543	0.028219	0.024567	0.047215
								713.05	0.028193	0.024533	0.047274
								715.558	0.028167	0.0245	0.047334
								718.065	0.028141	0.024466	0.047393
								720.572	0.028116	0.024432	0.047452
								723.08	0.028091	0.024399	0.04751
								725.59	0.028066	0.024366	0.047569
								728.1	0.028041	0.024332	0.047627
								730.61	0.028016	0.024299	0.047686
								733.12	0.027991	0.024265	0.047744
								735.622	0.027966	0.024232	0.047802
								738.125	0.027942	0.024199	0.047859
								740.628	0.027917	0.024166	0.047917
								743.13	0.027893	0.024133	0.047974
								745.64	0.027869	0.0241	0.048032
								748.15	0.027845	0.024067	0.048089
								750.66	0.027821	0.024034	0.048146
								753.17	0.027797	0.024001	0.048203
								755.678	0.027773	0.023968	0.048259
								758.185	0.027749	0.023935	0.048316
								760.693	0.027726	0.023902	0.048372

								763.2	0.027702	0.023869	0.048428
								765.707	0.027679	0.023837	0.048484
								768.215	0.027656	0.023804	0.04854
								770.723	0.027633	0.023771	0.048596
								773.23	0.02761	0.023739	0.048652
								775.74	0.027587	0.023706	0.048707
								778.25	0.027564	0.023674	0.048762
								780.76	0.027541	0.023641	0.048818
								783.27	0.027519	0.023609	0.048873
								785.777	0.027496	0.023577	0.048927
								788.285	0.027474	0.023544	0.048982
								790.793	0.027451	0.023512	0.049037
								793.3	0.027429	0.02348	0.049091
								795.808	0.027407	0.023448	0.049145
								798.315	0.027385	0.023416	0.049199
								800.822	0.027363	0.023384	0.049253
								803.33	0.027341	0.023352	0.049307
								805.84	0.027319	0.02332	0.049361
								808.35	0.027298	0.023288	0.049414
								810.86	0.027276	0.023256	0.049468
								813.37	0.027254	0.023225	0.049521
								815.872	0.027233	0.023193	0.049574
								818.375	0.027212	0.023162	0.049627
								820.878	0.027191	0.02313	0.049679
								823.38	0.027169	0.023099	0.049732
								825.89	0.027148	0.023067	0.049785
								828.4	0.027127	0.023036	0.049837
								830.91	0.027106	0.023005	0.049889
								833.42	0.027085	0.022973	0.049942
								835.928	0.027065	0.022942	0.049993
								838.435	0.027044	0.022911	0.050045

								840.943	0.027023	0.02288	0.050097
								843.45	0.027003	0.022849	0.050149
								845.957	0.026982	0.022818	0.0502
								848.465	0.026962	0.022787	0.050251
								850.973	0.026942	0.022756	0.050302
								853.48	0.026921	0.022725	0.050353
								855.99	0.026901	0.022695	0.050404
								858.5	0.026881	0.022664	0.050455
								861.01	0.026861	0.022633	0.050506
								863.52	0.026841	0.022603	0.050556
								866.027	0.026821	0.022572	0.050607
								868.535	0.026801	0.022542	0.050657
								871.043	0.026781	0.022512	0.050707
								873.55	0.026762	0.022481	0.050757
								873.55	0.026762	0.022481	0.050757

Table of ^{1}H NMR integration and COPASI fitted data for **2d** → *trans*-fused- and *cis*-fused-**3d** (*p*-F) in MeCN

Integral (6.54,6.43)	Integral (5.40,5.29)	Integral (4.54,4.45)	Sum	Time (min)	HA (M)	Trans (M)	Cis (M)	Fitted time (min)	Fitted HA (M)	Fitted trans (M)	Fitted cis (M)
				0	0.1	0	0	0	0.1	0	0
22604.3	144.16	39.76	22788.22	3.5	0.099193	0.000633	0.000174	0.875	0.099774	0.000161	6.52E-05
22562.6	210.43	62.55	22835.58	4.58	0.098805	0.000922	0.000274	1.75	0.09955	0.00032	0.00013
22431.3	219.31	82.96	22733.57	5.65	0.09867	0.000965	0.000365	2.625	0.099326	0.000479	0.000195
22383.6	271.26	97.05	22751.91	6.73	0.098381	0.001192	0.000427	3.5	0.099104	0.000637	0.000259
22714.7	345.79	134.17	23194.65	7.82	0.097931	0.001491	0.000578	3.77083	0.099035	0.000686	0.000279
22834.1	417.38	159.16	23410.64	8.9	0.097537	0.001783	0.00068	4.04167	0.098966	0.000735	0.000299
22789.4	450.07	179.48	23418.94	9.98	0.097312	0.001922	0.000766	4.3125	0.098898	0.000784	0.000319
22509.1	497.49	193.26	23199.84	11.05	0.097023	0.002144	0.000833	4.58333	0.09883	0.000832	0.000338
22463.9	545.31	213.34	23222.55	12.13	0.096733	0.002348	0.000919	4.85	0.098762	0.00088	0.000358
22153.4	578.93	231.03	22963.36	13.22	0.096473	0.002521	0.001006	5.11667	0.098695	0.000928	0.000377
22142.4	631.61	247.79	23021.8	14.3	0.09618	0.002744	0.001076	5.38333	0.098628	0.000975	0.000397
21999	689.64	251.91	22940.55	15.38	0.095896	0.003006	0.001098	5.65	0.098561	0.001023	0.000416
21910.4	743.59	278.51	22932.5	16.45	0.095543	0.003243	0.001214	5.92083	0.098493	0.001071	0.000436
21778	778.73	284.63	22841.36	17.53	0.095345	0.003409	0.001246	6.19167	0.098426	0.001119	0.000455
21825.7	828.43	327.22	22981.35	18.62	0.094971	0.003605	0.001424	6.4625	0.098358	0.001167	0.000475
21839.5	858.84	319.93	23018.27	19.7	0.094879	0.003731	0.00139	6.73333	0.098291	0.001215	0.000494
21601.2	899.36	362.76	22863.32	20.77	0.09448	0.003934	0.001587	7.00417	0.098223	0.001263	0.000514
21693.5	931.92	372.76	22998.18	21.85	0.094327	0.004052	0.001621	7.275	0.098156	0.001311	0.000533

21865.3	1024.03	377.99	23267.32	22.93	0.093974	0.004401	0.001625	7.54583	0.098089	0.001359	0.000553
21706.8	1058.22	425.49	23190.51	24.02	0.093602	0.004563	0.001835	7.81667	0.098021	0.001406	0.000572
21581.2	1106.68	442.84	23130.72	25.1	0.093301	0.004784	0.001915	8.0875	0.097954	0.001454	0.000592
21607.7	1147.14	457.04	23211.88	26.17	0.093089	0.004942	0.001969	8.35833	0.097887	0.001502	0.000611
21894	1221.32	494.22	23609.54	27.25	0.092734	0.005173	0.002093	8.62917	0.097821	0.001549	0.000631
21894.8	1284.89	516.18	23695.87	28.33	0.092399	0.005422	0.002178	8.9	0.097754	0.001596	0.00065
21827.5	1340.27	541	23708.77	29.42	0.092065	0.005653	0.002282	9.17083	0.097687	0.001644	0.000669
21726	1362.84	554.1	23642.94	30.5	0.091892	0.005764	0.002344	9.44167	0.097621	0.001691	0.000688
21633.4	1416.39	578.23	23628.02	31.57	0.091558	0.005995	0.002447	9.7125	0.097554	0.001738	0.000708
21592.3	1447.43	591.46	23631.19	32.65	0.091372	0.006125	0.002503	9.98333	0.097488	0.001785	0.000727
21475	1498.39	624.88	23598.27	33.73	0.091002	0.00635	0.002648	10.25	0.097423	0.001832	0.000746
21445	1539.18	659.88	23644.06	34.82	0.090699	0.00651	0.002791	10.5167	0.097357	0.001878	0.000765
21452.2	1580.81	643.4	23676.41	35.9	0.090606	0.006677	0.002717	10.7833	0.097292	0.001924	0.000784
21404.9	1607.25	656.5	23668.65	36.98	0.090436	0.006791	0.002774	11.05	0.097227	0.00197	0.000802
21364	1645.42	673.88	23683.3	38.05	0.090207	0.006948	0.002845	11.3208	0.097162	0.002017	0.000821
21268.6	1687.86	701.59	23658.05	39.13	0.0899	0.007134	0.002966	11.5917	0.097096	0.002064	0.000841
21155.7	1752.02	703.37	23611.09	40.22	0.089601	0.00742	0.002979	11.8625	0.09703	0.00211	0.00086
21159.5	1778.1	728.37	23665.97	41.3	0.089409	0.007513	0.003078	12.1333	0.096965	0.002157	0.000879
21171.9	1829.55	744.5	23745.95	42.38	0.08916	0.007705	0.003135	12.4042	0.096899	0.002203	0.000898
21026.2	1874.9	754.61	23655.71	43.45	0.088884	0.007926	0.00319	12.675	0.096834	0.00225	0.000917
21002	1908.94	772.8	23683.7	44.53	0.08867	0.00806	0.00326	12.9458	0.096768	0.002296	0.000936

			4		7		3				
20926.6	1922.57	791.94	23641.11	45.62	0.088518	0.008132	0.00335	13.2167	0.096703	0.002342	0.000954
20945.4	1983.78	804.93	23734.11	46.7	0.08825	0.008358	0.003391	13.4875	0.096638	0.002389	0.000973
20901.4	2014.2	833.95	23749.55	47.77	0.088008	0.008481	0.003511	13.7583	0.096573	0.002435	0.000992
20962.5	2028.58	828.66	23819.74	48.85	0.088005	0.008516	0.003479	14.0292	0.096508	0.002481	0.001011
20820.6	2088.09	852.92	23761.61	49.93	0.087623	0.008788	0.003589	14.3	0.096443	0.002527	0.00103
20694	2139.09	851.46	23684.55	51.02	0.087373	0.009032	0.003595	14.5708	0.096379	0.002573	0.001049
20680.8	2146.32	884.12	23711.24	52.1	0.087219	0.009052	0.003729	14.8417	0.096314	0.002619	0.001067
20671	2217.84	913.91	23802.75	53.17	0.086843	0.009318	0.00384	15.1125	0.09625	0.002664	0.001086
20635.5	2247.03	910.11	23792.64	54.25	0.086731	0.009444	0.003825	15.3833	0.096185	0.00271	0.001105
20566	2279.42	950.69	23796.11	55.33	0.086426	0.009579	0.003995	15.65	0.096122	0.002755	0.001123
20511	2291.1	934.71	23736.81	56.42	0.08641	0.009652	0.003938	15.9167	0.096059	0.0028	0.001142
20395.4	2357.05	958.17	23710.62	57.5	0.086018	0.009941	0.004041	16.1833	0.095995	0.002845	0.00116
20337.3	2353.56	974.65	23665.51	58.57	0.085936	0.009945	0.004118	16.45	0.095932	0.00289	0.001178
20314.1	2406.74	997.74	23718.58	59.65	0.085646	0.010147	0.004207	16.7208	0.095868	0.002935	0.001197
20349.6	2446.89	1007.87	23804.36	60.73	0.085487	0.010279	0.004234	16.9917	0.095804	0.00298	0.001215
20367.3	2498.6	1020.57	23886.47	61.82	0.085267	0.01046	0.004273	17.2625	0.095741	0.003026	0.001234
20275.5	2496	1042.14	23813.64	62.9	0.085142	0.010481	0.004376	17.5333	0.095677	0.003071	0.001252
20209.6	2548.85	1039.6	23798.05	63.97	0.084921	0.01071	0.004368	17.8042	0.095613	0.003116	0.001271
20144.1	2548.74	1053.07	23745.91	65.05	0.084832	0.010733	0.004435	18.075	0.09555	0.003161	0.001289
20044.1	2615.04	1076.95	23736.09	66.13	0.084446	0.011017	0.004537	18.3458	0.095486	0.003206	0.001308

20055.1	2660.13	1099.13	23814.36	67.22	0.084214	0.01117	0.004615	18.6167	0.095423	0.003251	0.001326
19980.2	2687.35	1108.53	23776.08	68.7	0.084035	0.011303	0.004662	18.8875	0.09536	0.003296	0.001345
19609.4	2971.75	1229.95	23811.1	78.4	0.082354	0.012481	0.005165	19.1583	0.095297	0.00334	0.001363
19260.3	3256.21	1346.71	23863.22	88.1	0.080711	0.013645	0.005643	19.4292	0.095234	0.003385	0.001381
18879.6	3488.35	1444.32	23812.27	97.78	0.079285	0.014649	0.006065	19.7	0.095171	0.00343	0.0014
18690.3	3710.5	1546.75	23947.55	107.48	0.078047	0.015494	0.006459	19.9667	0.095109	0.003474	0.001418
18325.4	3952.28	1638.47	23916.15	117.18	0.076624	0.016526	0.006851	20.2333	0.095047	0.003518	0.001435
17959.3	4136.8	1724.77	23820.87	126.88	0.075393	0.017366	0.007241	20.5	0.094985	0.003561	0.001453
17795.9	4351.65	1812.03	23959.58	136.58	0.074275	0.018162	0.007563	20.7667	0.094924	0.003605	0.001471
17478.6	4535.65	1920.37	23934.62	146.28	0.073026	0.01895	0.008023	21.0375	0.094861	0.003649	0.00149
17374.3	4744.95	1994.93	24114.18	155.98	0.07205	0.019677	0.008273	21.3083	0.094799	0.003694	0.001508
17021.7	4880.76	2087.54	23990	165.68	0.070953	0.020345	0.008702	21.5792	0.094736	0.003738	0.001526
16741.3	5063.26	2137.73	23942.29	175.37	0.069924	0.021148	0.008929	21.85	0.094674	0.003782	0.001544
16516.9	5231.18	2213.83	23961.91	185.07	0.06893	0.021831	0.009239	22.1208	0.094612	0.003826	0.001562
16314.7	5372.8	2276.06	23963.56	194.77	0.068081	0.022421	0.009498	22.3917	0.09455	0.00387	0.00158
16151.9	5520.85	2346.24	24018.99	204.47	0.067246	0.022985	0.009768	22.6625	0.094488	0.003914	0.001598
15979.1	5658.35	2418.17	24055.62	214.17	0.066426	0.023522	0.010052	22.9333	0.094426	0.003958	0.001616
15727.2	5820.07	2488.41	24035.68	223.87	0.065433	0.024214	0.010353	23.2042	0.094364	0.004002	0.001634
15556.4	5911.32	2534.03	24001.75	233.57	0.064814	0.024629	0.010558	23.475	0.094303	0.004045	0.001652
15416.9	6077.54	2600.11	24094.55	243.27	0.063985	0.025224	0.010791	23.7458	0.094241	0.004089	0.00167
15205.2	6146.62	2695.73	24047.5	252.95	0.06323	0.02556	0.01121	24.0167	0.09418	0.004133	0.001688

			5								
14910.4	6261.59	2729.55	23901.54	262.65	0.062383	0.026197	0.01142	24.2875	0.094118	0.004176	0.001706
14896.8	6404.44	2780.53	24081.77	272.35	0.061859	0.026595	0.011546	24.5583	0.094057	0.00422	0.001724
14726.9	6510.84	2810.26	24048	282.05	0.06124	0.027074	0.011686	24.8292	0.093996	0.004263	0.001742
14528.5	6583.64	2854.65	23966.79	291.75	0.060619	0.02747	0.011911	25.1	0.093934	0.004306	0.001759
14438.7	6697.14	2928.72	24064.56	301.45	0.06	0.02783	0.01217	25.3667	0.093874	0.004349	0.001777
14339.3	6758.14	2972.91	24070.35	311.15	0.059572	0.028077	0.012351	25.6333	0.093814	0.004392	0.001794
14127.4	6852.67	3037.09	24017.16	320.85	0.058822	0.028532	0.012646	25.9	0.093754	0.004434	0.001812
13979.5	6951.89	3072.34	24003.73	330.55	0.058239	0.028962	0.012799	26.1667	0.093694	0.004476	0.001829
13958.8	7059.33	3121.35	24139.48	340.23	0.057826	0.029244	0.01293	26.4375	0.093634	0.00452	0.001847
13757.5	7096.49	3177.43	24031.42	349.93	0.057248	0.02953	0.013222	26.7083	0.093573	0.004562	0.001865
13507.8	7245.71	3229.47	23982.98	359.63	0.056322	0.030212	0.013466	26.9792	0.093512	0.004605	0.001882
13600.5	7330.38	3234.3	24165.18	369.33	0.056281	0.030334	0.013384	27.25	0.093452	0.004648	0.0019
13422.5	7351.63	3341.11	24115.24	379.03	0.05566	0.030485	0.013855	27.5208	0.093391	0.004691	0.001918
13382.5	7470.24	3369.94	24222.68	388.73	0.055248	0.03084	0.013912	27.7917	0.093331	0.004734	0.001935
13213	7544.26	3416.21	24173.47	398.43	0.054659	0.031209	0.014132	28.0625	0.093271	0.004776	0.001953
12997.7	7623.68	3420.16	24041.54	408.13	0.054064	0.03171	0.014226	28.3333	0.093211	0.004819	0.00197
12883.5	7671.36	3486.41	24041.27	417.83	0.053589	0.031909	0.014502	28.6042	0.093151	0.004862	0.001988
12928.3	7723.35	3507.48	24159.13	427.52	0.053513	0.031969	0.014518	28.875	0.093091	0.004904	0.002005
12810	7846.54	3540.37	24196.91	437.22	0.052941	0.032428	0.014631	29.1458	0.093031	0.004946	0.002023
12677.6	7835.73	3596.21	24109.54	446.92	0.052583	0.032501	0.014916	29.4167	0.092971	0.004989	0.00204

12520.6	7939.05	3615.05	24074.7	456.62	0.052007	0.032977	0.015016	29.6875	0.092911	0.005031	0.002058
12474.5	7953.57	3657.99	24086.06	466.32	0.051791	0.033021	0.015187	29.9583	0.092852	0.005073	0.002075
12406.1	8037.17	3712.89	24156.16	476.02	0.051358	0.033272	0.01537	30.2292	0.092792	0.005115	0.002092
12212.7	8063.51	3718.86	23995.07	485.72	0.050897	0.033605	0.015498	30.5	0.092733	0.005158	0.00211
12196.9	8145.23	3751.55	24093.68	495.42	0.050623	0.033807	0.015571	30.7667	0.092674	0.005199	0.002127
11939.5	8191.85	3766.57	23897.92	505.1	0.04996	0.034279	0.015761	31.0333	0.092616	0.00524	0.002144
12055.7	8344.33	3813.02	24213.05	514.8	0.04979	0.034462	0.015748	31.3	0.092558	0.005282	0.002161
12009.8	8287.73	3861.16	24158.69	524.5	0.049712	0.034305	0.015982	31.5667	0.0925	0.005323	0.002178
11808.2	8345.65	3862.98	24016.83	534.2	0.049166	0.034749	0.016084	31.8375	0.092441	0.005365	0.002195
11864.8	8394.39	3921.53	24180.72	543.9	0.049067	0.034715	0.016218	32.1083	0.092382	0.005406	0.002212
11605.1	8432.62	3974.07	24011.79	553.6	0.048331	0.035119	0.01655	32.3792	0.092323	0.005448	0.002229
11688.1	8546.52	3953.06	24187.68	563.3	0.048323	0.035334	0.016343	32.65	0.092264	0.00549	0.002247
11574.5	8482.57	3998.32	24055.39	573	0.048116	0.035263	0.016621	32.9208	0.092205	0.005531	0.002264
11431.5	8574.74	4030.67	24036.91	582.68	0.047558	0.035673	0.016769	33.1917	0.092147	0.005573	0.002281
11391.7	8611.14	4092.12	24094.96	592.38	0.047278	0.035738	0.016983	33.4625	0.092088	0.005614	0.002298
11277.7	8666.39	4103.58	24047.67	602.08	0.046897	0.036038	0.017064	33.7333	0.09203	0.005655	0.002315
11374.2	8669.65	4122.17	24166.02	611.78	0.047067	0.035875	0.017058	34.0042	0.091971	0.005697	0.002332
11232.9	8764.92	4143.09	24140.91	621.48	0.046531	0.036307	0.017162	34.275	0.091913	0.005738	0.002349
11181.5	8760.32	4159.93	24101.75	631.18	0.046393	0.036347	0.01726	34.5458	0.091855	0.005779	0.002366
11130.1	8786.23	4165.56	24081.89	640.88	0.046218	0.036485	0.017297	34.8167	0.091797	0.00582	0.002383
11085.9	8839.18	4216.41	24141.4	650.58	0.04592	0.036614	0.01746	35.0875	0.091739	0.005861	0.0024

			9		1		5				
10956	8809.13	4207.98	23973.11	660.27	0.045701	0.036746	0.017553	35.3583	0.091681	0.005902	0.002417
10871.9	8922.89	4255.92	24050.71	669.97	0.045204	0.0371	0.017696	35.6292	0.091623	0.005943	0.002434
10786.6	8905.36	4300.75	23992.71	679.67	0.044958	0.037117	0.017925	35.9	0.091565	0.005984	0.002451
10692.5	8902.38	4338.7	23933.58	689.37	0.044676	0.037196	0.018128	36.1708	0.091507	0.006025	0.002468
10652.8	8961.01	4344.87	23958.68	699.07	0.044463	0.037402	0.018135	36.4417	0.09145	0.006066	0.002485
10641.2	8967.43	4341.91	23950.54	708.77	0.04443	0.037441	0.018129	36.7125	0.091392	0.006107	0.002501
10527.3	8985	4396.96	23909.26	718.47	0.04403	0.03758	0.01839	36.9833	0.091335	0.006147	0.002518
10479.2	9047.28	4367.53	23894.01	728.17	0.043857	0.037864	0.018279	37.25	0.091278	0.006187	0.002535
10414.2	9071.69	4410.73	23896.62	737.85	0.04358	0.037962	0.018458	37.5167	0.091222	0.006227	0.002551
10517.6	9112.48	4454.57	24084.65	747.55	0.043669	0.037835	0.018495	37.7833	0.091165	0.006267	0.002568
10279.1	9153.58	4493.33	23926.01	757.25	0.042962	0.038258	0.01878	38.05	0.091109	0.006307	0.002584
10192.7	9108.39	4492.41	23793.5	766.95	0.042838	0.038281	0.018881	38.3208	0.091052	0.006347	0.002601
10408	9171.53	4552.85	24132.38	776.65	0.043129	0.038005	0.018866	38.5917	0.090995	0.006387	0.002618
10236	9197.75	4492.56	23926.31	786.35	0.042781	0.038442	0.018777	38.8625	0.090938	0.006428	0.002634
10133.3	9224.41	4557.09	23914.8	796.05	0.042373	0.038572	0.019056	39.1333	0.090881	0.006468	0.002651
10261.9	9263.64	4573.26	24098.8	805.75	0.042583	0.03844	0.018977	39.4042	0.090825	0.006508	0.002667
10016.8	9234.42	4593.99	23845.21	815.43	0.042008	0.038727	0.019266	39.675	0.090768	0.006548	0.002684
10026	9290.22	4629.52	23945.74	825.13	0.04187	0.038797	0.019333	39.9458	0.090711	0.006588	0.002701
9901.91	9322.11	4650.17	23874.19	834.83	0.041475	0.039047	0.019478	40.2167	0.090655	0.006628	0.002717
9841.81	9364.98	4681.68	23888.47	844.53	0.041199	0.039203	0.019598	40.4875	0.090598	0.006668	0.002734

9790.33	9376.43	4627.2	23793.96	854.23	0.041146	0.039407	0.019447	40.7583	0.090542	0.006708	0.00275
9823.96	9399.95	4701.22	23925.13	863.93	0.041061	0.039289	0.01965	41.0292	0.090486	0.006748	0.002767
9823.83	9418.31	4705.83	23947.97	873.63	0.041022	0.039328	0.01965	41.3	0.090429	0.006787	0.002783
9811.93	9370.17	4717.72	23899.82	883.33	0.041054	0.039206	0.01974	41.5708	0.090373	0.006827	0.0028
9626.81	9433.58	4769.03	23829.42	893.02	0.040399	0.039588	0.020013	41.8417	0.090317	0.006867	0.002816
9622.74	9449.18	4804.64	23876.56	902.72	0.040302	0.039575	0.020123	42.1125	0.090261	0.006906	0.002832
								42.3833	0.090205	0.006946	0.002849
								42.65	0.09015	0.006985	0.002865
								42.9167	0.090096	0.007023	0.002881
								43.1833	0.090041	0.007062	0.002897
								43.45	0.089986	0.007101	0.002913
								43.7208	0.089931	0.00714	0.002929
								43.9917	0.089875	0.007179	0.002946
								44.2625	0.08982	0.007218	0.002962
								44.5333	0.089765	0.007257	0.002978
								44.8042	0.089709	0.007296	0.002994
								45.075	0.089654	0.007335	0.00301
								45.3458	0.089599	0.007374	0.003027
								45.6167	0.089544	0.007413	0.003043
								45.8875	0.089489	0.007452	0.003059
								46.1583	0.089434	0.007491	0.003075
								46.4292	0.08938	0.007529	0.003091
								46.7	0.089325	0.007568	0.003107
								46.9667	0.089271	0.007606	0.003123
								47.2333	0.089218	0.007644	0.003139
								47.5	0.089164	0.007682	0.003154
								47.7667	0.089111	0.007719	0.00317
								48.0375	0.089056	0.007758	0.003186

								48.3083	0.089002	0.007796	0.003202
								48.5792	0.088948	0.007834	0.003218
								48.85	0.088894	0.007873	0.003234
								49.1208	0.08884	0.007911	0.00325
								49.3917	0.088786	0.007949	0.003265
								49.6625	0.088732	0.007987	0.003281
								49.9333	0.088678	0.008025	0.003297
								50.2042	0.088625	0.008063	0.003313
								50.475	0.088571	0.0081	0.003329
								50.7458	0.088517	0.008138	0.003344
								51.0167	0.088464	0.008176	0.00336
								51.2875	0.088411	0.008214	0.003376
								51.5583	0.088357	0.008251	0.003391
								51.8292	0.088304	0.008289	0.003407
								52.1	0.088251	0.008327	0.003423
								52.3667	0.088198	0.008363	0.003438
								52.6333	0.088146	0.0084	0.003454
								52.9	0.088094	0.008437	0.003469
								53.1667	0.088042	0.008474	0.003484
								53.4375	0.087989	0.008511	0.0035
								53.7083	0.087936	0.008548	0.003515
								53.9792	0.087884	0.008586	0.003531
								54.25	0.087831	0.008623	0.003546
								54.5208	0.087779	0.00866	0.003562
								54.7917	0.087726	0.008697	0.003577
								55.0625	0.087674	0.008734	0.003593
								55.3333	0.087621	0.008771	0.003608
								55.6042	0.087569	0.008807	0.003623
								55.875	0.087517	0.008844	0.003639
								56.1458	0.087465	0.008881	0.003654
								56.4167	0.087413	0.008918	0.00367

								56.6875	0.0873<br>61	0.00895<br>4	0.0036<br>85
								56.9583	0.0873<br>09	0.00899<br>1	0.0037
								57.2292	0.0872<br>57	0.00902<br>8	0.0037<br>15
								57.5	0.0872<br>05	0.00906<br>4	0.0037<br>31
								57.7667	0.0871<br>54	0.0091	0.0037<br>46
								58.0333	0.0871<br>04	0.00913<br>6	0.0037<br>61
								58.3	0.0870<br>53	0.00917<br>2	0.0037<br>76
								58.5667	0.0870<br>02	0.00920<br>7	0.0037<br>91
								58.8375	0.0869<br>51	0.00924<br>4	0.0038<br>06
								59.1083	0.0868<br>99	0.00928	0.0038<br>21
								59.3792	0.0868<br>48	0.00931<br>6	0.0038<br>36
								59.65	0.0867<br>97	0.00935<br>2	0.0038<br>51
								59.9208	0.0867<br>46	0.00938<br>8	0.0038<br>66
								60.1917	0.0866<br>95	0.00942<br>4	0.0038<br>81
								60.4625	0.0866<br>44	0.00946	0.0038<br>96
								60.7333	0.0865<br>93	0.00949<br>6	0.0039<br>11
								61.0042	0.0865<br>42	0.00953<br>2	0.0039<br>26
								61.275	0.0864<br>91	0.00956<br>8	0.0039<br>41
								61.5458	0.0864<br>4	0.00960<br>3	0.0039<br>56
								61.8167	0.0863<br>9	0.00963<br>9	0.0039<br>71
								62.0875	0.0863<br>39	0.00967<br>5	0.0039<br>86
								62.3583	0.0862<br>89	0.00971	0.0040<br>01
								62.6292	0.0862<br>38	0.00974<br>6	0.0040<br>16
								62.9	0.0861<br>88	0.00978<br>1	0.0040<br>31
								63.1667	0.0861<br>38	0.00981<br>6	0.0040<br>46
								63.4333	0.0860<br>89	0.00985<br>1	0.0040<br>6
								63.7	0.0860<br>39	0.00988<br>6	0.0040<br>75
								63.9667	0.0859<br>9	0.00992<br>1	0.0040<br>89
								64.2375	0.0859<br>4	0.00995<br>6	0.0041<br>04
								64.5083	0.0858<br>9	0.00999<br>1	0.0041<br>19
								64.7792	0.0858<br>4	0.01002<br>6	0.0041<br>34

								65.05	0.08579	0.010061	0.004148
								65.3208	0.08574	0.010096	0.004163
								65.5917	0.085691	0.010131	0.004178
								65.8625	0.085641	0.010166	0.004193
								66.1333	0.085592	0.010201	0.004207
								66.4042	0.085542	0.010236	0.004222
								66.675	0.085493	0.010271	0.004236
								66.9458	0.085443	0.010306	0.004251
								67.2167	0.085394	0.010341	0.004266
								67.5875	0.085326	0.010388	0.004286
								67.9583	0.085259	0.010435	0.004306
								68.3292	0.085192	0.010483	0.004325
								68.7	0.085125	0.01053	0.004345
								71.125	0.084689	0.010836	0.004474
								73.55	0.084259	0.011139	0.004602
								75.975	0.083834	0.011438	0.004728
								78.4	0.083414	0.011733	0.004853
								80.825	0.082998	0.012025	0.004977
								83.25	0.082588	0.012313	0.005099
								85.675	0.082182	0.012598	0.00522
								88.1	0.081781	0.01288	0.00534
								90.5208	0.081385	0.013157	0.005458
								92.9417	0.080994	0.013431	0.005575
								95.3625	0.080607	0.013702	0.00569
								97.7833	0.080225	0.01397	0.005805
								100.208	0.079846	0.014235	0.005919
								102.633	0.079472	0.014497	0.006031
								105.058	0.079101	0.014756	0.006142
								107.483	0.078735	0.015012	0.006253
								109.908	0.078374	0.015265	0.006362
								112.333	0.078016	0.015515	0.00647

								114.758	0.077662	0.015762	0.006576
								117.183	0.077312	0.016006	0.006682
								119.608	0.076966	0.016247	0.006787
								122.033	0.076623	0.016486	0.006891
								124.458	0.076285	0.016722	0.006994
								126.883	0.07595	0.016955	0.007095
								129.308	0.075619	0.017185	0.007196
								131.733	0.075291	0.017413	0.007296
								134.158	0.074967	0.017638	0.007394
								136.583	0.074647	0.017861	0.007492
								139.008	0.07433	0.018081	0.007589
								141.433	0.074016	0.018299	0.007685
								143.858	0.073706	0.018514	0.00778
								146.283	0.073399	0.018727	0.007874
								148.708	0.073095	0.018938	0.007968
								151.133	0.072794	0.019146	0.00806
								153.558	0.072497	0.019352	0.008152
								155.983	0.072203	0.019555	0.008242
								158.408	0.071912	0.019756	0.008332
								160.833	0.071624	0.019955	0.008421
								163.258	0.071339	0.020152	0.008509
								165.683	0.071056	0.020347	0.008597
								168.104	0.070778	0.020539	0.008683
								170.525	0.070502	0.020729	0.008769
								172.946	0.070229	0.020917	0.008854
								175.367	0.069959	0.021103	0.008938
								177.792	0.069691	0.021287	0.009021
								180.217	0.069426	0.02147	0.009104
								182.642	0.069164	0.02165	0.009186
								185.067	0.068904	0.021828	0.009268
								187.492	0.068647	0.022005	0.009348

								189.917	0.068393	0.022179	0.009428
								192.342	0.068141	0.022352	0.009507
								194.767	0.067891	0.022523	0.009586
								197.192	0.067645	0.022692	0.009664
								199.617	0.0674	0.022859	0.009741
								202.042	0.067158	0.023024	0.009817
								204.467	0.066919	0.023188	0.009893
								206.892	0.066682	0.02335	0.009968
								209.317	0.066447	0.02351	0.010043
								211.742	0.066215	0.023669	0.010117
								214.167	0.065985	0.023825	0.01019
								216.592	0.065757	0.023981	0.010263
								219.017	0.065531	0.024134	0.010335
								221.442	0.065308	0.024286	0.010407
								223.867	0.065086	0.024436	0.010477
								226.292	0.064867	0.024585	0.010548
								228.717	0.064651	0.024732	0.010617
								231.142	0.064436	0.024878	0.010687
								233.567	0.064223	0.025022	0.010755
								235.992	0.064012	0.025165	0.010823
								238.417	0.063804	0.025306	0.010891
								240.842	0.063597	0.025446	0.010957
								243.267	0.063393	0.025584	0.011024
								245.688	0.06319	0.02572	0.01109
								248.108	0.06299	0.025856	0.011155
								250.529	0.062791	0.025989	0.011219
								252.95	0.062595	0.026122	0.011284
								255.375	0.0624	0.026253	0.011347
								257.8	0.062206	0.026383	0.011411
								260.225	0.062015	0.026512	0.011473
								262.65	0.061825	0.026639	0.011536

								265.075	0.061638	0.026765	0.011598
								267.5	0.061452	0.02689	0.011659
								269.925	0.061267	0.027013	0.01172
								272.35	0.061085	0.027135	0.01178
								274.775	0.060904	0.027256	0.01184
								277.2	0.060725	0.027376	0.011899
								279.625	0.060547	0.027494	0.011958
								282.05	0.060371	0.027612	0.012017
								284.475	0.060197	0.027728	0.012075
								286.9	0.060025	0.027843	0.012133
								289.325	0.059854	0.027957	0.01219
								291.75	0.059684	0.028069	0.012247
								294.175	0.059516	0.028181	0.012303
								296.6	0.05935	0.028291	0.012359
								299.025	0.059185	0.028401	0.012414
								301.45	0.059022	0.028509	0.01247
								303.875	0.05886	0.028616	0.012524
								306.3	0.0587	0.028722	0.012579
								308.725	0.058541	0.028827	0.012633
								311.15	0.058383	0.028931	0.012686
								313.575	0.058227	0.029034	0.012739
								316	0.058073	0.029136	0.012792
								318.425	0.057919	0.029237	0.012844
								320.85	0.057768	0.029336	0.012896
								323.275	0.057617	0.029435	0.012948
								325.7	0.057468	0.029533	0.012999
								328.125	0.05732	0.02963	0.01305
								330.55	0.057174	0.029726	0.0131
								332.971	0.057029	0.029821	0.01315
								335.392	0.056885	0.029915	0.0132
								337.812	0.056743	0.030008	0.01325

								340.233	0.056602	0.0301	0.013299
								342.658	0.056462	0.030191	0.013347
								345.083	0.056323	0.030282	0.013396
								347.508	0.056185	0.030371	0.013444
								349.933	0.056049	0.03046	0.013491
								352.358	0.055913	0.030548	0.013539
								354.783	0.055779	0.030635	0.013586
								357.208	0.055647	0.030721	0.013633
								359.633	0.055515	0.030806	0.013679
								362.058	0.055384	0.030891	0.013725
								364.483	0.055255	0.030974	0.013771
								366.908	0.055127	0.031057	0.013817
								369.333	0.055	0.031139	0.013862
								371.758	0.054873	0.03122	0.013907
								374.183	0.054749	0.0313	0.013951
								376.608	0.054625	0.03138	0.013996
								379.033	0.054502	0.031459	0.01404
								381.458	0.05438	0.031537	0.014083
								383.883	0.054259	0.031614	0.014127
								386.308	0.05414	0.031691	0.01417
								388.733	0.054021	0.031766	0.014213
								391.158	0.053903	0.031842	0.014255
								393.583	0.053787	0.031916	0.014297
								396.008	0.053671	0.031989	0.014339
								398.433	0.053557	0.032062	0.014381
								400.858	0.053443	0.032135	0.014423
								403.283	0.05333	0.032206	0.014464
								405.708	0.053219	0.032277	0.014505
								408.133	0.053108	0.032347	0.014545
								410.558	0.052998	0.032416	0.014586
								412.983	0.052889	0.032485	0.014626

								415.408	0.052781	0.032553	0.014666
								417.833	0.052674	0.032621	0.014705
								420.254	0.052568	0.032687	0.014745
								422.675	0.052463	0.032753	0.014784
								425.096	0.052359	0.032819	0.014823
								427.517	0.052255	0.032884	0.014861
								429.942	0.052153	0.032948	0.0149
								432.367	0.052051	0.033011	0.014938
								434.792	0.05195	0.033074	0.014976
								437.217	0.05185	0.033137	0.015013
								439.642	0.051751	0.033199	0.015051
								442.067	0.051652	0.03326	0.015088
								444.492	0.051555	0.033321	0.015125
								446.917	0.051458	0.033381	0.015162
								449.342	0.051362	0.03344	0.015198
								451.767	0.051267	0.033499	0.015235
								454.192	0.051172	0.033557	0.015271
								456.617	0.051079	0.033615	0.015307
								459.042	0.050986	0.033672	0.015342
								461.467	0.050894	0.033729	0.015378
								463.892	0.050802	0.033785	0.015413
								466.317	0.050712	0.033841	0.015448
								468.742	0.050622	0.033896	0.015483
								471.167	0.050533	0.03395	0.015517
								473.592	0.050444	0.034004	0.015552
								476.017	0.050357	0.034058	0.015586
								478.442	0.05027	0.03411	0.01562
								480.867	0.050183	0.034163	0.015654
								483.292	0.050098	0.034215	0.015687
								485.717	0.050013	0.034266	0.015721
								488.142	0.049929	0.034317	0.015754

								490.567	0.049845	0.034368	0.015787
								492.992	0.049763	0.034418	0.01582
								495.417	0.049681	0.034467	0.015853
								497.838	0.049599	0.034516	0.015885
								500.258	0.049519	0.034565	0.015917
								502.679	0.049439	0.034613	0.015949
								505.1	0.049359	0.03466	0.015981
								507.525	0.04928	0.034707	0.016013
								509.95	0.049202	0.034754	0.016044
								512.375	0.049124	0.0348	0.016076
								514.8	0.049047	0.034846	0.016107
								517.225	0.048971	0.034891	0.016138
								519.65	0.048895	0.034936	0.016169
								522.075	0.04882	0.034981	0.016199
								524.5	0.048746	0.035025	0.01623
								526.925	0.048672	0.035068	0.01626
								529.35	0.048598	0.035112	0.01629
								531.775	0.048526	0.035154	0.01632
								534.2	0.048453	0.035197	0.01635
								536.625	0.048382	0.035239	0.01638
								539.05	0.048311	0.03528	0.016409
								541.475	0.04824	0.035321	0.016438
								543.9	0.04817	0.035362	0.016468
								546.325	0.048101	0.035403	0.016497
								548.75	0.048032	0.035443	0.016526
								551.175	0.047964	0.035482	0.016554
								553.6	0.047896	0.035521	0.016583
								556.025	0.047829	0.03556	0.016611
								558.45	0.047762	0.035599	0.016639
								560.875	0.047696	0.035637	0.016667
								563.3	0.04763	0.035674	0.016695

								565.725	0.047565	0.035712	0.016723
								568.15	0.047501	0.035749	0.016751
								570.575	0.047436	0.035785	0.016778
								573	0.047373	0.035822	0.016806
								575.421	0.04731	0.035857	0.016833
								577.842	0.047247	0.035893	0.01686
								580.262	0.047185	0.035928	0.016887
								582.683	0.047124	0.035963	0.016913
								585.108	0.047063	0.035997	0.01694
								587.533	0.047002	0.036032	0.016967
								589.958	0.046942	0.036066	0.016993
								592.383	0.046882	0.036099	0.017019
								594.808	0.046823	0.036132	0.017045
								597.233	0.046764	0.036165	0.017071
								599.658	0.046705	0.036198	0.017097
								602.083	0.046647	0.03623	0.017123
								604.508	0.04659	0.036262	0.017148
								606.933	0.046533	0.036293	0.017174
								609.358	0.046476	0.036325	0.017199
								611.783	0.04642	0.036356	0.017224
								614.208	0.046364	0.036386	0.017249
								616.633	0.046309	0.036417	0.017274
								619.058	0.046254	0.036447	0.017299
								621.483	0.0462	0.036477	0.017324
								623.908	0.046146	0.036506	0.017348
								626.333	0.046092	0.036535	0.017373
								628.758	0.046039	0.036564	0.017397
								631.183	0.045986	0.036593	0.017421
								633.608	0.045934	0.036621	0.017445
								636.033	0.045882	0.036649	0.017469
								638.458	0.04583	0.036677	0.017493

								640.883	0.045779	0.036704	0.017517
								643.308	0.045728	0.036732	0.017541
								645.733	0.045678	0.036758	0.017564
								648.158	0.045628	0.036785	0.017587
								650.583	0.045578	0.036812	0.017611
								653.004	0.045529	0.036838	0.017634
								655.425	0.04548	0.036863	0.017657
								657.846	0.045431	0.036889	0.01768
								660.267	0.045383	0.036914	0.017703
								662.692	0.045335	0.036939	0.017725
								665.117	0.045288	0.036964	0.017748
								667.542	0.045241	0.036989	0.01777
								669.967	0.045194	0.037013	0.017793
								672.392	0.045148	0.037037	0.017815
								674.817	0.045102	0.037061	0.017837
								677.242	0.045056	0.037085	0.017859
								679.667	0.045011	0.037108	0.017881
								682.092	0.044966	0.037131	0.017903
								684.517	0.044921	0.037154	0.017925
								686.942	0.044877	0.037177	0.017947
								689.367	0.044833	0.037199	0.017968
								691.792	0.044789	0.037221	0.01799
								694.217	0.044746	0.037243	0.018011
								696.642	0.044703	0.037265	0.018032
								699.067	0.04466	0.037287	0.018054
								701.492	0.044618	0.037308	0.018075
								703.917	0.044575	0.037329	0.018096
								706.342	0.044534	0.03735	0.018116
								708.767	0.044492	0.037371	0.018137
								711.192	0.044451	0.037391	0.018158
								713.617	0.04441	0.037411	0.018179

								716.042	0.04437	0.037431	0.018199
								718.467	0.04433	0.037451	0.01822
								720.892	0.04429	0.037471	0.01824
								723.317	0.04425	0.03749	0.01826
								725.742	0.044211	0.037509	0.01828
								728.167	0.044172	0.037528	0.0183
								730.588	0.044133	0.037547	0.01832
								733.008	0.044094	0.037566	0.01834
								735.429	0.044056	0.037584	0.01836
								737.85	0.044018	0.037602	0.01838
								740.275	0.043981	0.03762	0.018399
								742.7	0.043943	0.037638	0.018419
								745.125	0.043906	0.037655	0.018438
								747.55	0.04387	0.037673	0.018458
								749.975	0.043833	0.03769	0.018477
								752.4	0.043797	0.037707	0.018496
								754.825	0.043761	0.037724	0.018515
								757.25	0.043725	0.037741	0.018534
								759.675	0.04369	0.037757	0.018553
								762.1	0.043654	0.037774	0.018572
								764.525	0.043619	0.03779	0.018591
								766.95	0.043585	0.037806	0.01861
								769.375	0.04355	0.037821	0.018628
								771.8	0.043516	0.037837	0.018647
								774.225	0.043482	0.037853	0.018665
								776.65	0.043448	0.037868	0.018684
								779.075	0.043415	0.037883	0.018702
								781.5	0.043382	0.037898	0.01872
								783.925	0.043349	0.037913	0.018739
								786.35	0.043316	0.037927	0.018757
								788.775	0.043284	0.037942	0.018775

								791.2	0.043251	0.037956	0.018793
								793.625	0.043219	0.03797	0.018811
								796.05	0.043187	0.037984	0.018828
								798.475	0.043156	0.037998	0.018846
								800.9	0.043125	0.038012	0.018864
								803.325	0.043094	0.038025	0.018881
								805.75	0.043063	0.038039	0.018899
								808.171	0.043032	0.038052	0.018916
								810.592	0.043002	0.038065	0.018934
								813.012	0.042971	0.038078	0.018951
								815.433	0.042942	0.03809	0.018968
								817.858	0.042912	0.038103	0.018985
								820.283	0.042882	0.038116	0.019002
								822.708	0.042853	0.038128	0.01902
								825.133	0.042824	0.03814	0.019036
								827.558	0.042795	0.038152	0.019053
								829.983	0.042766	0.038164	0.01907
								832.408	0.042737	0.038176	0.019087
								834.833	0.042709	0.038187	0.019104
								837.258	0.042681	0.038199	0.01912
								839.683	0.042653	0.03821	0.019137
								842.108	0.042625	0.038221	0.019153
								844.533	0.042598	0.038232	0.01917
								846.958	0.042571	0.038243	0.019186
								849.383	0.042543	0.038254	0.019202
								851.808	0.042517	0.038265	0.019219
								854.233	0.04249	0.038275	0.019235
								856.658	0.042463	0.038286	0.019251
								859.083	0.042437	0.038296	0.019267
								861.508	0.042411	0.038306	0.019283
								863.933	0.042385	0.038316	0.019299

								866.358	0.042359	0.038326	0.019315
								868.783	0.042333	0.038336	0.019331
								871.208	0.042308	0.038346	0.019346
								873.633	0.042283	0.038355	0.019362
								876.058	0.042258	0.038365	0.019378
								878.483	0.042233	0.038374	0.019393
								880.908	0.042208	0.038383	0.019409
								883.333	0.042183	0.038392	0.019424
								885.754	0.042159	0.038401	0.01944
								888.175	0.042135	0.03841	0.019455
								890.596	0.042111	0.038419	0.01947
								893.017	0.042087	0.038428	0.019486
								895.442	0.042063	0.038436	0.019501
								897.867	0.04204	0.038444	0.019516
								900.292	0.042016	0.038453	0.019531
								902.717	0.041993	0.038461	0.019546
								902.717	0.041993	0.038461	0.019546

Table of ^{1}H NMR integration and COPASI fitted data for **2e** → *trans*-fused- and *cis*-fused-**3e** (*p*-H) in MeCN

**Integral (6.37,6.27)**	**Integral (5.39,5.30)**	**Integral (4.67,4.64)**	**Sum**	**Time (min)**	**HA (M)**	**Trans (M)**	**Cis (M)**	**Fitted time (min)**	**Fitted HA (M)**	**Fitted trans (M)**	**Fitted cis (M)**
80391.2	151.55	22.87	80565.62	**3.5**	0.099784	0.000188	2.83868E-05	0	0.1	0	0
80268.9	242.99	47.05	80558.94	**4.58**	0.09964	0.000302	5.84044E-05	0.875	0.099928	5.38E-05	1.87E-05
80417.1	318.97	30.92	80766.99	**5.67**	0.099567	0.000395	3.8283E-05	1.75	0.099855	0.000107	3.73E-05
80113	361.51	61.38	80535.89	**6.75**	0.099475	0.000449	7.62145E-05	2.625	0.099783	0.000161	5.60E-05
80117.9	419.43	47.42	80584.75	**7.83**	0.099421	0.00052	5.88449E-05	3.5	0.099711	0.000215	7.46E-05
79723.2	442.39	75.56	80241.15	**8.9**	0.099355	0.000551	9.41661E-05	3.77083	0.099689	0.000231	8.03E-05
79904.4	515.01	111.71	80531.12	**9.98**	0.099222	0.00064	0.000138717	4.04167	0.099666	0.000248	8.61E-05
79505.4	567.95	180.86	80254.2	**11.07**	0.099067	0.000708	0.000225359	4.3125	0.099644	0.000264	9.18E-05
79665.2	945.26	300.44	80910.9	**18.48**	0.09846	0.001168	0.000371322	4.58333	0.099622	0.000281	9.76E-05
78836.8	1444.13	458.99	80739.92	**28.52**	0.097643	0.001789	0.00056848	4.85417	0.0996	0.000297	0.000103
78071.3	1912.66	665.48	80649.44	**38.55**	0.096803	0.002372	0.000825151	5.125	0.099577	0.000314	0.000109
77331.3	2355.7	761.13	80448.13	**48.58**	0.096126	0.002928	0.000946113	5.39583	0.099555	0.00033	0.000115
76884.4	2833.21	991.1	80708.71	**58.6**	0.095262	0.00351	0.001227996	5.66667	0.099533	0.000347	0.00012
76313.2	3344.43	1168.63	80826.26	**68.63**	0.094416	0.004138	0.001445854	5.9375	0.099511	0.000363	0.000126
76139.1	3667.71	1297.67	81104.48	**78.67**	0.093878	0.004522	0.001599998	6.20833	0.099489	0.00038	0.000132
75451.7	4223.42	1300.54	80975.66	**88.7**	0.093178	0.005216	0.001606088	6.47917	0.099466	0.000396	0.000138
74973	4560.18	1642.98	81176.16	**98.73**	0.092358	0.005618	0.002023969	6.75	0.099444	0.000412	0.000143
74124.6	5023.67	1661.15	80809.42	**108.77**	0.091728	0.006217	0.002055639	7.02083	0.099422	0.000429	0.000149
73768	5410.66	1862.45	81041.1	**118.8**	0.09102	0.006676	0.00229815	7.29167	0.0994	0.000445	0.000155

			1		5		5				
73300.9	5797.72	2035.6	81134.22	**128.82**	0.090345	0.007146	0.002508929	7.5625	0.099378	0.000462	0.000161
72747.3	6129.54	2184	81060.84	**138.85**	0.089744	0.007562	0.002694273	7.83333	0.099356	0.000478	0.000166
72086.9	6477.63	2279.11	80843.64	**148.88**	0.089168	0.008013	0.002819158	8.1	0.099334	0.000494	0.000172
71934.7	6873.84	2463.06	81271.6	**158.92**	0.088511	0.008458	0.003030653	8.36667	0.099312	0.00051	0.000177
71457.5	7242.17	2592.66	81292.33	**168.95**	0.087902	0.008909	0.003189305	8.63333	0.099291	0.000526	0.000183
70988.2	7620.57	2677.22	81285.99	**178.98**	0.087331	0.009375	0.003293581	8.9	0.099269	0.000543	0.000189
70676.7	7996.01	2842.22	81514.93	**189.02**	0.086704	0.009809	0.003486748	9.17083	0.099247	0.000559	0.000194
70013.1	8302.56	2884.44	81200.1	**199.05**	0.086223	0.010225	0.003552262	9.44167	0.099225	0.000575	0.0002
69596	8609.42	3071.39	81276.81	**209.08**	0.085628	0.010593	0.003778925	9.7125	0.099203	0.000592	0.000206
69131.5	8992.64	3155.44	81279.58	**219.1**	0.085054	0.011064	0.003882205	9.98333	0.099181	0.000608	0.000211
68617.7	9262.35	3235.56	81115.61	**229.13**	0.084592	0.011419	0.003988825	10.2542	0.099159	0.000624	0.000217
68473.9	9649.96	3401.74	81525.6	**239.17**	0.083991	0.011837	0.004172603	10.525	0.099137	0.000641	0.000223
67874.5	9955.41	3512.88	81342.79	**249.2**	0.083443	0.012239	0.004318613	10.7958	0.099115	0.000657	0.000228
67573.6	10283.2	3623.67	81480.47	**259.23**	0.082932	0.01262	0.004447287	11.0667	0.099093	0.000673	0.000234
67336.7	10506.3	3572.74	81415.74	**269.27**	0.082707	0.012905	0.004388267	12.9208	0.098943	0.000784	0.000273
66769.3	10769.6	3842.77	81381.67	**279.3**	0.082045	0.013233	0.004721911	14.775	0.098793	0.000895	0.000312
66384.9	11094.8	3953.36	81433.06	**289.33**	0.081521	0.013624	0.004854736	16.6292	0.098644	0.001006	0.00035
65902.2	11530.4	3847.55	81280.15	**299.35**	0.08108	0.014186	0.00473369	18.4833	0.098496	0.001116	0.000388
65538.1	11770.6	4200.11	81508.81	**309.38**	0.080406	0.014441	0.005152952	20.9917	0.098296	0.001264	0.00044
65592	12035	4033.9	81660.9	**319.42**	0.080322	0.014738	0.004939818	23.5	0.098097	0.001412	0.000492

65053.5	12299.3	4349.32	81702.12	**329.45**	0.079623	0.015054	0.005323387	26.0083	0.097899	0.001558	0.000543
64185.3	12500.7	5002.79	81688.79	**339.48**	0.078573	0.015303	0.006124206	28.5167	0.097702	0.001704	0.000594
64250.8	12829.6	4684.1	81764.5	**349.52**	0.07858	0.015691	0.00572877	31.025	0.097506	0.001849	0.000645
63997.9	13063.8	4746.94	81808.64	**359.55**	0.078229	0.015969	0.005802492	33.5333	0.097311	0.001994	0.000695
63662	13280	4802.15	81744.15	**369.58**	0.07788	0.016246	0.00587461	36.0417	0.097117	0.002138	0.000745
63189.5	13451	4920.92	81561.42	**379.62**	0.077475	0.016492	0.006033392	38.55	0.096924	0.002281	0.000795
62954.2	13570.1	4992.81	81517.11	**389.63**	0.077228	0.016647	0.006124861	41.0583	0.096732	0.002423	0.000845
62745.5	13867.6	5052.43	81665.53	**399.67**	0.076832	0.016981	0.006186735	43.5667	0.09654	0.002565	0.000895
62425.9	14058.6	5173.43	81657.93	**409.7**	0.076448	0.017216	0.00633549	46.075	0.09635	0.002706	0.000944
62072.4	14300.2	5181.69	81554.29	**419.73**	0.076112	0.017535	0.006353669	48.5833	0.096161	0.002846	0.000994
61567.1	14473	5275.48	81315.58	**429.77**	0.075714	0.017799	0.006487662	51.0875	0.095973	0.002985	0.001042
61336	14877.1	5330.15	81543.25	**439.8**	0.075219	0.018244	0.006536593	53.5917	0.095785	0.003124	0.001091
61180.4	15009.9	5409.71	81600.01	**449.83**	0.074976	0.018394	0.006629546	56.0958	0.095599	0.003262	0.00114
60886.2	15183.7	5652.62	81722.52	**459.87**	0.074504	0.01858	0.006916845	58.6	0.095414	0.003399	0.001188
60493.2	15440.9	5631.22	81565.32	**469.88**	0.074165	0.018931	0.006903939	61.1083	0.095229	0.003535	0.001236
60463.8	15569.9	5832.34	81866.04	**479.92**	0.073857	0.019019	0.007124248	63.6167	0.095045	0.003672	0.001284
59994.3	15811.7	5768.42	81574.42	**489.95**	0.073545	0.019383	0.007071359	66.125	0.094862	0.003807	0.001331
59845.7	15943.4	5984.22	81773.32	**499.98**	0.073185	0.019497	0.007318059	68.6333	0.09468	0.003942	0.001379
59438.6	16156.3	6022.95	81617.85	**510.02**	0.072825	0.019795	0.007379452	71.1417	0.094498	0.004076	0.001426
59390.6	16355.3	6018.78	81764.68	**520.05**	0.072636	0.020003	0.0073611	73.65	0.094318	0.004209	0.001473
59108.9	16620.3	6182.88	81912.08	**530.08**	0.072161	0.02029	0.007548191	76.1583	0.094138	0.004342	0.00152

58604.6	16993.7	6035.56	81633.86	**540.12**	0.07179	0.020817	0.007393452	78.6667	0.09396	0.004474	0.001567
58487.8	16871.9	6256.35	81616.05	**550.13**	0.071662	0.020672	0.007665588	81.175	0.093782	0.004605	0.001613
58218.7	17056.4	6332.99	81608.09	**560.17**	0.071339	0.0209	0.007760248	83.6833	0.093605	0.004736	0.001659
58002.4	17603.5	6082.13	81688.03	**570.2**	0.071005	0.02155	0.007445558	86.1917	0.093429	0.004866	0.001705
57780.9	17538.2	6574.34	81893.44	**580.23**	0.070556	0.021416	0.00802792	88.7	0.093254	0.004995	0.001751
57497.1	17802.1	6542.3	81841.5	**590.27**	0.070254	0.021752	0.007993866	91.2083	0.093079	0.005124	0.001797
57687.6	17916.8	6937.3	82541.7	**600.3**	0.069889	0.021706	0.0084046	93.7167	0.092906	0.005252	0.001842
56973	18128.8	6707.42	81809.22	**610.33**	0.069641	0.0216	0.008198856	96.225	0.092733	0.00538	0.001887
56718.5	18185.1	6772.58	81676.18	**620.37**	0.069443	0.022265	0.008291989	98.7333	0.092561	0.005507	0.001932
56468.2	18623.9	6710.17	81802.27	**630.38**	0.06903	0.022767	0.008202914	101.242	0.09239	0.005633	0.001977
56272.8	18535.2	6832.57	81640.57	**640.42**	0.068927	0.022703	0.008369087	103.75	0.092219	0.005759	0.002022
56272.7	18879.5	6921.72	82073.92	**650.45**	0.068563	0.023003	0.008433519	106.258	0.09205	0.005884	0.002066
55853.2	18840.9	6981.07	81675.17	**660.48**	0.068385	0.023068	0.008547359	108.767	0.091881	0.006009	0.00211
55856.6	18841.7	7443.97	82142.27	**670.52**	0.068	0.022938	0.009062289	111.275	0.091713	0.006132	0.002154
55426.9	19339.4	7020.37	81786.67	**680.55**	0.06777	0.023646	0.008583758	113.783	0.091546	0.006256	0.002198
55188.1	19202.6	7233.29	81623.99	**690.58**	0.067613	0.023526	0.008861721	116.292	0.09138	0.006378	0.002242
55078.6	19383.3	7347.5	81809.4	**700.62**	0.067326	0.023693	0.008981242	118.8	0.091214	0.0065	0.002285
54656.1	19495.2	7411.02	81562.32	**710.63**	0.067011	0.023902	0.009086328	121.304	0.09105	0.006622	0.002329
54381	19854.8	7363.04	81598.84	**720.67**	0.066644	0.024332	0.009023462	123.808	0.090886	0.006742	0.002372
54274.1	20015.6	7526.4	81816.1	**730.7**	0.066337	0.024464	0.009199167	126.313	0.090723	0.006863	0.002414
54004.9	20151.3	7484.33	81640.5	**740.73**	0.06615	0.024683	0.00916742	128.817	0.090561	0.006982	0.002457

			3								
53893.8	20107.8	7769.69	81771.29	**750.77**	0.065908	0.02459	0.009501733	131.325	0.090399	0.007101	0.0025
53685	20168	7712.15	81565.15	**760.8**	0.065819	0.024726	0.009455202	133.833	0.090238	0.00722	0.002542
53549.4	20916.2	7271.52	81737.12	**770.83**	0.065514	0.02559	0.008896227	136.342	0.090078	0.007338	0.002584
53244.6	20483.3	7607.15	81335.05	**780.87**	0.065463	0.025184	0.009352856	138.85	0.089919	0.007455	0.002626
53232.9	20690.1	7703.53	81626.53	**790.88**	0.065215	0.025347	0.009437532	141.358	0.08976	0.007572	0.002668
53001	20764.2	8053.41	81818.61	**800.92**	0.064779	0.025378	0.009843005	143.867	0.089602	0.007688	0.00271
52570.5	20872.9	7999.25	81442.65	**810.95**	0.064549	0.025629	0.009821942	146.375	0.089445	0.007804	0.002751
52494.2	21063.8	8003.78	81561.78	**820.98**	0.064361	0.025826	0.00981315	148.883	0.089288	0.007919	0.002793
52214.4	21462.5	8002.89	81679.79	**831.02**	0.063926	0.026276	0.009797883	151.392	0.089132	0.008034	0.002834
52187.7	21329.6	8241.81	81759.11	**841.05**	0.063831	0.026088	0.010080601	153.9	0.088977	0.008148	0.002875
51996.5	21606	8295.5	81898	**851.08**	0.063489	0.026382	0.010129063	156.408	0.088823	0.008262	0.002916
51742.4	21826.3	8193.91	81762.61	**861.12**	0.063284	0.026695	0.010021586	158.917	0.088669	0.008375	0.002956
51538	21980.2	8180.33	81698.53	**871.13**	0.063083	0.026904	0.010012824	161.425	0.088516	0.008487	0.002997
51488.6	21803.5	8269.69	81561.79	**881.17**	0.063128	0.026732	0.010139172	163.933	0.088364	0.008599	0.003037
								166.442	0.088213	0.00871	0.003077
								168.95	0.088062	0.008821	0.003117
								171.458	0.087912	0.008932	0.003157
								173.967	0.087762	0.009041	0.003196
								176.475	0.087613	0.009151	0.003236
								178.983	0.087465	0.00926	0.003275
								181.492	0.087318	0.009368	0.003314
								184	0.087171	0.009476	0.003353
								186.508	0.087025	0.009583	0.003392

								189.017	0.08688	0.009689	0.003431
								191.525	0.086735	0.009796	0.003469
								194.033	0.086591	0.009901	0.003508
								196.542	0.086447	0.010007	0.003546
								199.05	0.086305	0.010111	0.003584
								201.558	0.086163	0.010216	0.003622
								204.067	0.086021	0.010319	0.00366
								206.575	0.08588	0.010423	0.003697
								209.083	0.08574	0.010526	0.003735
								211.587	0.085601	0.010628	0.003772
								214.092	0.085462	0.010729	0.003809
								216.596	0.085324	0.01083	0.003846
								219.1	0.085186	0.010931	0.003882
								221.608	0.085049	0.011032	0.003919
								224.117	0.084913	0.011132	0.003956
								226.625	0.084777	0.011231	0.003992
								229.133	0.084642	0.01133	0.004028
								231.642	0.084507	0.011429	0.004064
								234.15	0.084373	0.011527	0.0041
								236.658	0.08424	0.011624	0.004136
								239.167	0.084107	0.011721	0.004172
								241.675	0.083975	0.011818	0.004207
								244.183	0.083843	0.011915	0.004243
								246.692	0.083712	0.01201	0.004278
								249.2	0.083581	0.012106	0.004313
								251.708	0.083451	0.012201	0.004348
								254.217	0.083322	0.012295	0.004383
								256.725	0.083193	0.012389	0.004417
								259.233	0.083065	0.012483	0.004452
								261.742	0.082938	0.012576	0.004486
								264.25	0.08281	0.012669	0.004521

								266.758	0.082684	0.012761	0.004555
								269.267	0.082558	0.012853	0.004589
								271.775	0.082433	0.012945	0.004623
								274.283	0.082308	0.013036	0.004656
								276.792	0.082183	0.013127	0.00469
								279.3	0.08206	0.013217	0.004724
								281.808	0.081936	0.013307	0.004757
								284.317	0.081814	0.013396	0.00479
								286.825	0.081692	0.013485	0.004823
								289.333	0.08157	0.013574	0.004856
								291.837	0.081449	0.013662	0.004889
								294.342	0.081329	0.01375	0.004922
								296.846	0.081209	0.013837	0.004954
								299.35	0.08109	0.013924	0.004987
								301.858	0.080971	0.01401	0.005019
								304.367	0.080852	0.014097	0.005051
								306.875	0.080734	0.014183	0.005083
								309.383	0.080617	0.014268	0.005115
								311.892	0.0805	0.014353	0.005147
								314.4	0.080384	0.014438	0.005179
								316.908	0.080268	0.014522	0.00521
								319.417	0.080152	0.014606	0.005242
								321.925	0.080037	0.01469	0.005273
								324.433	0.079923	0.014773	0.005304
								326.942	0.079809	0.014856	0.005336
								329.45	0.079696	0.014938	0.005367
								331.958	0.079583	0.01502	0.005397
								334.467	0.07947	0.015102	0.005428
								336.975	0.079358	0.015183	0.005459
								339.483	0.079247	0.015264	0.005489
								341.992	0.079136	0.015345	0.00552

								344.5	0.079025	0.015425	0.00555
								347.008	0.078915	0.015505	0.00558
								349.517	0.078805	0.015584	0.005611
								352.025	0.078696	0.015663	0.00564
								354.533	0.078588	0.015742	0.00567
								357.042	0.078479	0.015821	0.0057
								359.55	0.078372	0.015899	0.00573
								362.058	0.078264	0.015977	0.005759
								364.567	0.078157	0.016054	0.005789
								367.075	0.078051	0.016131	0.005818
								369.583	0.077945	0.016208	0.005847
								372.092	0.07784	0.016284	0.005876
								374.6	0.077735	0.01636	0.005905
								377.108	0.07763	0.016436	0.005934
								379.617	0.077526	0.016511	0.005963
								382.121	0.077422	0.016586	0.005992
								384.625	0.077319	0.016661	0.00602
								387.129	0.077217	0.016735	0.006049
								389.633	0.077114	0.016809	0.006077
								392.142	0.077012	0.016883	0.006105
								394.65	0.076911	0.016956	0.006133
								397.158	0.07681	0.017029	0.006161
								399.667	0.076709	0.017102	0.006189
								402.175	0.076609	0.017174	0.006217
								404.683	0.076509	0.017246	0.006245
								407.192	0.076409	0.017318	0.006273
								409.7	0.07631	0.01739	0.0063
								412.208	0.076212	0.017461	0.006328
								414.717	0.076114	0.017532	0.006355
								417.225	0.076016	0.017602	0.006382
								419.733	0.075918	0.017672	0.006409

								422.242	0.075822	0.017742	0.006436
								424.75	0.075725	0.017812	0.006463
								427.258	0.075629	0.017881	0.00649
								429.767	0.075533	0.01795	0.006517
								432.275	0.075438	0.018019	0.006544
								434.783	0.075343	0.018087	0.00657
								437.292	0.075248	0.018155	0.006597
								439.8	0.075154	0.018223	0.006623
								442.308	0.07506	0.018291	0.006649
								444.817	0.074967	0.018358	0.006676
								447.325	0.074874	0.018425	0.006702
								449.833	0.074781	0.018491	0.006728
								452.342	0.074689	0.018558	0.006754
								454.85	0.074597	0.018624	0.006779
								457.358	0.074505	0.018689	0.006805
								459.867	0.074414	0.018755	0.006831
								462.371	0.074324	0.01882	0.006856
								464.875	0.074234	0.018885	0.006882
								467.379	0.074144	0.018949	0.006907
								469.883	0.074054	0.019014	0.006932
								472.392	0.073965	0.019078	0.006958
								474.9	0.073876	0.019141	0.006983
								477.408	0.073788	0.019205	0.007008
								479.917	0.073699	0.019268	0.007033
								482.425	0.073612	0.019331	0.007057
								484.933	0.073524	0.019394	0.007082
								487.442	0.073437	0.019456	0.007107
								489.95	0.07335	0.019518	0.007132
								492.458	0.073264	0.01958	0.007156
								494.967	0.073178	0.019642	0.00718
								497.475	0.073092	0.019703	0.007205

								499.983	0.073007	0.019764	0.007229
								502.492	0.072922	0.019825	0.007253
								505	0.072837	0.019886	0.007277
								507.508	0.072753	0.019946	0.007301
								510.017	0.072669	0.020006	0.007325
								512.525	0.072585	0.020066	0.007349
								515.033	0.072502	0.020125	0.007373
								517.542	0.072419	0.020185	0.007397
								520.05	0.072336	0.020244	0.00742
								522.558	0.072254	0.020302	0.007444
								525.067	0.072172	0.020361	0.007467
								527.575	0.072091	0.020419	0.00749
								530.083	0.072009	0.020477	0.007514
								532.592	0.071928	0.020535	0.007537
								535.1	0.071848	0.020592	0.00756
								537.608	0.071767	0.02065	0.007583
								540.117	0.071687	0.020707	0.007606
								542.621	0.071608	0.020763	0.007629
								545.125	0.071529	0.02082	0.007652
								547.629	0.07145	0.020876	0.007674
								550.133	0.071371	0.020932	0.007697
								552.642	0.071293	0.020988	0.007719
								555.15	0.071215	0.021043	0.007742
								557.658	0.071137	0.021099	0.007764
								560.167	0.07106	0.021154	0.007787
								562.675	0.070982	0.021209	0.007809
								565.183	0.070906	0.021263	0.007831
								567.692	0.070829	0.021318	0.007853
								570.2	0.070753	0.021372	0.007875
								572.708	0.070677	0.021426	0.007897
								575.217	0.070601	0.021479	0.007919

								577.725	0.070526	0.021533	0.007941
								580.233	0.070451	0.021586	0.007963
								582.742	0.070376	0.021639	0.007985
								585.25	0.070302	0.021692	0.008006
								587.758	0.070228	0.021745	0.008028
								590.267	0.070154	0.021797	0.008049
								592.775	0.07008	0.021849	0.008071
								595.283	0.070007	0.021901	0.008092
								597.792	0.069934	0.021953	0.008113
								600.3	0.069861	0.022004	0.008134
								602.808	0.069789	0.022056	0.008156
								605.317	0.069717	0.022107	0.008177
								607.825	0.069645	0.022157	0.008198
								610.333	0.069574	0.022208	0.008218
								612.842	0.069502	0.022258	0.008239
								615.35	0.069431	0.022309	0.00826
								617.858	0.069361	0.022359	0.008281
								620.367	0.06929	0.022408	0.008301
								622.871	0.06922	0.022458	0.008322
								625.375	0.06915	0.022507	0.008342
								627.879	0.069081	0.022556	0.008363
								630.383	0.069012	0.022605	0.008383
								632.892	0.068943	0.022654	0.008404
								635.4	0.068874	0.022702	0.008424
								637.908	0.068805	0.022751	0.008444
								640.417	0.068737	0.022799	0.008464
								642.925	0.068669	0.022847	0.008484
								645.433	0.068602	0.022894	0.008504
								647.942	0.068534	0.022942	0.008524
								650.45	0.068467	0.022989	0.008544
								652.958	0.0684	0.023036	0.008564

								655.467	0.068333	0.023083	0.008583
								657.975	0.068267	0.02313	0.008603
								660.483	0.068201	0.023177	0.008623
								662.992	0.068135	0.023223	0.008642
								665.5	0.068069	0.023269	0.008662
								668.008	0.068004	0.023315	0.008681
								670.517	0.067939	0.023361	0.0087
								673.025	0.067874	0.023406	0.00872
								675.533	0.067809	0.023452	0.008739
								678.042	0.067745	0.023497	0.008758
								680.55	0.067681	0.023542	0.008777
								683.058	0.067617	0.023587	0.008796
								685.567	0.067554	0.023631	0.008815
								688.075	0.06749	0.023676	0.008834
								690.583	0.067427	0.02372	0.008853
								693.092	0.067364	0.023764	0.008872
								695.6	0.067302	0.023808	0.008891
								698.108	0.067239	0.023852	0.008909
								700.617	0.067177	0.023895	0.008928
								703.121	0.067115	0.023938	0.008946
								705.625	0.067054	0.023982	0.008965
								708.129	0.066992	0.024024	0.008983
								710.633	0.066931	0.024067	0.009002
								713.142	0.06687	0.02411	0.00902
								715.65	0.066809	0.024152	0.009038
								718.158	0.066749	0.024195	0.009057
								720.667	0.066689	0.024237	0.009075
								723.175	0.066629	0.024279	0.009093
								725.683	0.066569	0.02432	0.009111
								728.192	0.066509	0.024362	0.009129
								730.7	0.06645	0.024403	0.009147

								733.208	0.066391	0.024445	0.009165
								735.717	0.066332	0.024486	0.009183
								738.225	0.066273	0.024526	0.0092
								740.733	0.066215	0.024567	0.009218
								743.242	0.066157	0.024608	0.009236
								745.75	0.066099	0.024648	0.009253
								748.258	0.066041	0.024688	0.009271
								750.767	0.065983	0.024728	0.009288
								753.275	0.065926	0.024768	0.009306
								755.783	0.065869	0.024808	0.009323
								758.292	0.065812	0.024848	0.009341
								760.8	0.065755	0.024887	0.009358
								763.308	0.065699	0.024926	0.009375
								765.817	0.065643	0.024965	0.009392
								768.325	0.065586	0.025004	0.009409
								770.833	0.065531	0.025043	0.009427
								773.342	0.065475	0.025081	0.009444
								775.85	0.06542	0.02512	0.009461
								778.358	0.065364	0.025158	0.009477
								780.867	0.065309	0.025196	0.009494
								783.371	0.065255	0.025234	0.009511
								785.875	0.0652	0.025272	0.009528
								788.379	0.065146	0.02531	0.009545
								790.883	0.065092	0.025347	0.009561
								793.392	0.065038	0.025384	0.009578
								795.9	0.064984	0.025421	0.009594
								798.408	0.064931	0.025458	0.009611
								800.917	0.064877	0.025495	0.009627
								803.425	0.064824	0.025532	0.009644
								805.933	0.064771	0.025569	0.00966
								808.442	0.064719	0.025605	0.009677

								810.95	0.064666	0.025641	0.009693
								813.458	0.064614	0.025677	0.009709
								815.967	0.064561	0.025713	0.009725
								818.475	0.06451	0.025749	0.009741
								820.983	0.064458	0.025785	0.009757
								823.492	0.064406	0.02582	0.009773
								826	0.064355	0.025856	0.009789
								828.508	0.064304	0.025891	0.009805
								831.017	0.064253	0.025926	0.009821
								833.525	0.064202	0.025961	0.009837
								836.033	0.064151	0.025996	0.009853
								838.542	0.064101	0.026031	0.009869
								841.05	0.064051	0.026065	0.009884
								843.558	0.064001	0.0261	0.0099
								846.067	0.063951	0.026134	0.009916
								848.575	0.063901	0.026168	0.009931
								851.083	0.063852	0.026202	0.009947
								853.592	0.063802	0.026236	0.009962
								856.1	0.063753	0.026269	0.009978
								858.608	0.063704	0.026303	0.009993
								861.117	0.063655	0.026336	0.010008
								863.621	0.063607	0.02637	0.010024
								866.125	0.063559	0.026403	0.010039
								868.629	0.063511	0.026436	0.010054
								871.133	0.063463	0.026468	0.010069
								873.642	0.063415	0.026501	0.010084
								876.15	0.063367	0.026534	0.010099
								878.658	0.06332	0.026566	0.010114
								881.167	0.063272	0.026599	0.010129

Table of ^{1}H NMR integration and COPASI fitted data for **2f** → *trans*-fused- and *cis*-fused-**3f** (*p*-Cl) in MeCN

Integral (6.42,6.22)	Integral (5.38,5.33)	Integral (2.77,2.64)	Sum	Time (min)	HA (M)	Trans (M)	Cis (M)	Fitted time (min)	fitted HA (M)	Fitted trans (M)	Fitted cis (M)
108099	147.5	259.68	108506.2	3.5	0.099625	0.000136	0.000239	0	0.1	0	0
105450	280.05	871.45	106601.5	4.58	0.09892	0.000263	0.000817	0.875	0.099979	1.31E-05	8.40E-06
105320	313.38	1552.68	107186.1	5.67	0.098259	0.000292	0.001449	1.75	0.099957	2.61E-05	1.68E-05
105648	283.6	845.09	106776.7	6.73	0.098943	0.000266	0.000791	2.625	0.099936	3.91E-05	2.52E-05
105545	408.69	1372.92	107326.6	7.82	0.09834	0.000381	0.001279	3.5	0.099914	5.22E-05	3.35E-05
105373	302	848.68	106523.7	8.9	0.09892	0.000284	0.000797	3.77083	0.099908	5.62E-05	3.61E-05
105650	348.86	479.55	106478.4	9.98	0.099222	0.000328	0.00045	4.04167	0.099901	6.02E-05	3.87E-05
106121	325.65	88.64	106535.3	11.07	0.099611	0.000306	8.32E-05	4.3125	0.099895	6.43E-05	4.13E-05
106060	323.34	305.6	106688.9	12.13	0.09941	0.000303	0.000286	4.58333	0.099888	6.83E-05	4.38E-05
105519	451.88	648.64	106619.5	13.22	0.098968	0.000424	0.000608	4.85417	0.099881	7.23E-05	4.64E-05
105765	355.25	1184.14	107304.4	14.3	0.098565	0.000331	0.001104	5.125	0.099875	7.63E-05	4.90E-05
107389	295.94	325.33	108010.3	15.38	0.099425	0.000274	0.000301	5.39583	0.099868	8.04E-05	5.16E-05
105605	470.57	417.48	106493.1	16.47	0.099166	0.000442	0.000392	5.66667	0.099862	8.44E-05	5.41E-05
106932	424.94	185.32	107542.3	17.53	0.099433	0.000395	0.000172	5.93333	0.099855	8.84E-05	5.67E-05
105994	484.96	390.08	106869	18.62	0.099181	0.000454	0.000365	6.2	0.099849	9.23E-05	5.92E-05
107138	348.05	157.71	107643.8	21.88	0.09953	0.000323	0.000147	6.46667	0.099842	9.63E-05	6.17E-05
105581	717.17	722.91	107021.1	31.92	0.098654	0.00067	0.000675	6.73333	0.099836	0.0001	6.42E-05
105362	816.96	351.08	106530	41.93	0.098904	0.000767	0.00033	7.00417	0.099829	0.000104	6.68E-05
105428	993.72	1420.28	107842	51.97	0.097762	0.000921	0.001317	7.275	0.099822	0.000108	6.94E-05
105730	1119.52	886.55	107736.	62	0.09813	0.001039	0.00082	7.54583	0.099816	0.000112	7.19E-05

			1		8		3				
105971	1207.27	596.99	107775.3	72.03	0.098326	0.00112	0.000554	7.81667	0.099809	0.000116	7.45E-05
104692	1534.32	1748.39	107974.7	82.07	0.09696	0.001421	0.001619	8.0875	0.099803	0.00012	7.70E-05
105227	1573.62	726.12	107526.7	92.1	0.097861	0.001463	0.000675	8.35833	0.099796	0.000124	7.96E-05
104603	1769.92	1507.7	107880.6	102.13	0.096962	0.001641	0.001398	8.62917	0.09979	0.000128	8.21E-05
104384	1947.31	1831.37	108162.7	112.15	0.096506	0.0018	0.001693	8.9	0.099783	0.000132	8.47E-05
104226	2032.1	2074.84	108332.9	122.18	0.096209	0.001876	0.001915	9.17083	0.099776	0.000136	8.72E-05
103764	2238.21	2143.64	108145.9	132.22	0.095948	0.00207	0.001982	9.44167	0.09977	0.00014	8.98E-05
104451	2245.66	460.86	107157.5	142.25	0.097474	0.002096	0.00043	9.7125	0.099763	0.000144	9.23E-05
103530	2443.76	2019.41	107993.2	152.28	0.095867	0.002263	0.00187	9.98333	0.099757	0.000148	9.49E-05
104101	2591.4	1971.94	108664.3	162.32	0.095801	0.002385	0.001815	10.2542	0.09975	0.000152	9.74E-05
105029	2561.17	750.29	108340.5	172.35	0.096943	0.002364	0.000693	10.525	0.099744	0.000156	1.00E-04
103612	2807.79	1322.02	107741.8	182.38	0.096167	0.002606	0.001227	10.7958	0.099737	0.00016	0.000103
106208	2855.35	772.63	109836	192.42	0.096697	0.0026	0.000703	11.0667	0.099731	0.000164	0.000105
103061	3056.95	1971.74	108089.7	202.43	0.095348	0.002828	0.001824	11.3333	0.099724	0.000168	0.000108
102512	3215.67	2139.15	107866.8	212.47	0.095036	0.002981	0.001983	11.6	0.099718	0.000172	0.00011
102700	3429.61	1934.21	108063.8	222.5	0.095036	0.003174	0.00179	11.8667	0.099711	0.000176	0.000113
102486	3529.64	2012.48	108028.1	232.53	0.09487	0.003267	0.001863	12.1333	0.099705	0.00018	0.000115
101961	3646.16	1596.82	107204	242.57	0.095109	0.003401	0.00149	12.4042	0.099698	0.000184	0.000118
101913	3792.75	2264.02	107969.8	252.6	0.09439	0.003513	0.002097	12.675	0.099692	0.000188	0.00012
101404	3920.74	3084.97	108409.7	262.63	0.093538	0.003617	0.002846	12.9458	0.099685	0.000192	0.000123

101509	4028.76	2569.86	108107.6	272.67	0.093896	0.003727	0.002377	13.2167	0.099679	0.000196	0.000125
101767	4078.07	2379.72	108224.8	282.68	0.094033	0.003768	0.002199	13.4875	0.099672	0.0002	0.000128
101139	4313.24	3250.99	108703.2	292.72	0.093041	0.003968	0.002991	13.7583	0.099666	0.000204	0.00013
104518	4223.04	962.75	109703.8	302.75	0.095273	0.003849	0.000878	14.0292	0.099659	0.000208	0.000133
101131	4454.43	2472.29	108057.7	312.78	0.09359	0.004122	0.002288	14.3	0.099653	0.000212	0.000135
100738	4627.63	3140.62	108506.3	322.82	0.092841	0.004265	0.002894	14.5708	0.099646	0.000216	0.000138
102213	4630.56	1507.93	108351.5	332.85	0.094335	0.004274	0.001392	14.8417	0.09964	0.00022	0.00014
101046	4853.13	1945.51	107844.6	342.88	0.093696	0.0045	0.001804	15.1125	0.099633	0.000224	0.000143
101818	4793.81	1001.33	107613.1	352.92	0.094615	0.004455	0.00093	15.3833	0.099627	0.000228	0.000145
100763	5058.88	2155.85	107977.7	362.93	0.093318	0.004685	0.001997	15.6542	0.09962	0.000232	0.000148
100153	5241.94	2865.9	108260.8	372.97	0.092511	0.004842	0.002647	15.925	0.099614	0.000236	0.00015
101045	5294.31	1995.45	108334.8	383	0.093271	0.004887	0.001842	16.1958	0.099607	0.00024	0.000153
101398	5226.39	1079.91	107704.3	393.03	0.094145	0.004853	0.001003	16.4667	0.099601	0.000244	0.000155
99258.8	5585.25	3651.48	108495.5	403.07	0.091487	0.005148	0.003366	16.7333	0.099594	0.000248	0.000158
99641	5638.92	3272.44	108552.4	413.1	0.091791	0.005195	0.003015	17	0.099588	0.000252	0.00016
100050	5745.69	1909.79	107705.5	423.13	0.092892	0.005335	0.001773	17.2667	0.099581	0.000256	0.000163
99432.7	5833.09	3178.83	108444.6	433.17	0.09169	0.005379	0.002931	17.5333	0.099575	0.00026	0.000165
99756.6	5923.22	2215.52	107895.3	443.18	0.092457	0.00549	0.002053	17.8042	0.099569	0.000264	0.000168
99776.6	5997.85	2028.53	107803	453.22	0.092555	0.005564	0.001882	18.075	0.099562	0.000268	0.00017
98897.6	6152.53	3289.03	108339.2	463.25	0.091285	0.005679	0.003036	18.3458	0.099556	0.000272	0.000173
98788.2	6271	3236.89	108296.	473.28	0.09122	0.005791	0.00298	18.6167	0.099549	0.000276	0.000175

			1				9				
98839	6377.71	2755.06	107971.8	483.32	0.091542	0.005907	0.002552	19.4333	0.09953	0.000288	0.000183
99762.5	6409.56	2262.67	108434.7	493.35	0.092002	0.005911	0.002087	20.25	0.09951	0.0003	0.00019
99099.7	6498.6	1910.32	107508.6	503.38	0.092178	0.006045	0.001777	21.0667	0.099491	0.000312	0.000198
98116	6736.93	3094.67	107947.6	513.4	0.090892	0.006241	0.002867	21.8833	0.099471	0.000324	0.000205
98285.7	6752.11	2731.4	107769.2	523.43	0.0912	0.006265	0.002534	24.3917	0.099412	0.00036	0.000228
98030.2	6962.5	4208.46	109201.2	533.47	0.08977	0.006376	0.003854	26.9	0.099352	0.000397	0.000251
98382.3	6985.33	3130.67	108498.3	543.5	0.090676	0.006438	0.002885	29.4083	0.099293	0.000433	0.000273
97564.2	7139.89	3115.31	107819.4	553.53	0.090489	0.006622	0.002889	31.9167	0.099234	0.00047	0.000296
98134.4	7228.99	3197.83	108561.2	563.57	0.090395	0.006659	0.002946	34.4208	0.099176	0.000506	0.000318
97893.8	7289.65	3109.65	108293.1	573.6	0.090397	0.006731	0.002872	36.925	0.099118	0.000542	0.00034
98011.7	7355.08	2344.29	107711.1	583.63	0.090995	0.006829	0.002176	39.4292	0.099059	0.000579	0.000362
97261	7501.35	3023.42	107785.8	593.65	0.090235	0.006959	0.002805	41.9333	0.099001	0.000615	0.000384
97898.5	7541.91	2558.47	107998.9	603.68	0.090648	0.006983	0.002369	44.4417	0.098944	0.000651	0.000406
96497	7780.07	4417.32	108694.4	613.72	0.088778	0.007158	0.004064	46.95	0.098886	0.000687	0.000428
97139.4	7751.46	3289.41	108180.3	623.75	0.089794	0.007165	0.003041	49.4583	0.098828	0.000722	0.000449
96639.7	7923.84	3156.48	107720	633.78	0.089714	0.007356	0.00293	51.9667	0.098771	0.000758	0.000471
98899.9	7795.62	2232.25	108927.8	643.82	0.090794	0.007157	0.002049	54.475	0.098714	0.000794	0.000492
96127.8	8175.03	4446.68	108749.5	653.85	0.088394	0.007517	0.004089	56.9833	0.098657	0.00083	0.000513
95891.6	8268.47	4368.11	108528.2	663.88	0.088356	0.007619	0.004025	59.4917	0.098601	0.000865	0.000534
97312.8	8259.73	2592.34	108164.9	673.9	0.089967	0.007636	0.002397	62	0.098544	0.000901	0.000555

95685.7	8487.38	4519.5	108692.6	683.93	0.088033	0.007809	0.004158	64.5083	0.098488	0.000936	0.000576
96104.9	8525.83	3874.74	108505.5	693.97	0.088571	0.007858	0.003571	67.0167	0.098432	0.000971	0.000597
96402.7	8542.34	3347.74	108292.8	704	0.08902	0.007888	0.003091	69.525	0.098376	0.001007	0.000617
96165.2	8647.71	2964.47	107777.4	714.03	0.089226	0.008024	0.002751	72.0333	0.098321	0.001042	0.000638
96200.5	8678.6	2685.11	107564.2	724.07	0.089435	0.008068	0.002496	74.5417	0.098265	0.001077	0.000658
96946.7	8741.14	2493.3	108181.1	734.1	0.089615	0.00808	0.002305	77.05	0.09821	0.001112	0.000678
94564.6	8934.97	3539.82	107039.4	744.13	0.088346	0.008347	0.003307	79.5583	0.098155	0.001147	0.000698
95539.5	8952.33	3682.33	108174.2	754.15	0.08832	0.008276	0.003404	82.0667	0.0981	0.001182	0.000718
94502.9	9134.79	4640.78	108278.5	764.18	0.087278	0.008436	0.004286	84.575	0.098045	0.001217	0.000738
94636.3	9234.69	4521.09	108392.1	774.22	0.087309	0.00852	0.004171	87.0833	0.097991	0.001251	0.000758
94677.7	9274.91	3486.26	107438.9	784.25	0.088122	0.008633	0.003245	89.5917	0.097937	0.001286	0.000777
94092	9467.09	4804.82	108363.9	794.28	0.08683	0.008736	0.004434	92.1	0.097883	0.001321	0.000797
94623	9471.55	3290.69	107385.2	804.32	0.088115	0.00882	0.003064	94.6083	0.097829	0.001355	0.000816
93731.9	9616.12	4614.85	107962.9	814.35	0.086819	0.008907	0.004274	97.1167	0.097775	0.00139	0.000835
94622.7	9593.33	3880.47	108096.5	824.38	0.087535	0.008875	0.00359	99.625	0.097722	0.001424	0.000855
94619.4	9708.17	2915.6	107243.2	834.4	0.088229	0.009052	0.002719	102.133	0.097668	0.001458	0.000874
94005	9812.1	3477.92	107295	844.43	0.087614	0.009145	0.003241	104.637	0.097615	0.001492	0.000893
93740.4	9866.37	3821.23	107428	854.47	0.087259	0.009184	0.003557	107.142	0.097562	0.001526	0.000911
96379.9	9797.28	2538.61	108715.8	864.5	0.088653	0.009012	0.002335	109.646	0.09751	0.00156	0.00093
93106.1	10134.4	5126.63	108367.1	874.53	0.085917	0.009352	0.004731	112.15	0.097457	0.001594	0.000949
93782.8	10051	3645.55	107479.	884.57	0.08725	0.009352	0.00339	114.658	0.097405	0.001628	0.000967

			4		7		2				
92876.8	10200.5	3808.19	106885.5	894.6	0.086894	0.009543	0.003563	117.167	0.097353	0.001662	0.000985
92121	10309.2	4958.89	107389.1	904.63	0.085782	0.0096	0.004618	119.675	0.097301	0.001696	0.001004
92040.1	10260.4	3942.75	106243.3	914.67	0.086631	0.009657	0.003711	122.183	0.097249	0.001729	0.001022
								124.692	0.097197	0.001763	0.00104
								127.2	0.097146	0.001797	0.001058
								129.708	0.097094	0.00183	0.001076
								132.217	0.097043	0.001863	0.001093
								134.725	0.096992	0.001897	0.001111
								137.233	0.096941	0.00193	0.001129
								139.742	0.096891	0.001963	0.001146
								142.25	0.09684	0.001996	0.001163
								144.758	0.09679	0.002029	0.001181
								147.267	0.09674	0.002062	0.001198
								149.775	0.09669	0.002095	0.001215
								152.283	0.09664	0.002128	0.001232
								154.792	0.096591	0.002161	0.001248
								157.3	0.096541	0.002194	0.001265
								159.808	0.096492	0.002226	0.001282
								162.317	0.096443	0.002259	0.001298
								164.825	0.096394	0.002291	0.001315
								167.333	0.096345	0.002324	0.001331
								169.842	0.096297	0.002356	0.001347
								172.35	0.096248	0.002388	0.001363
								174.858	0.0962	0.002421	0.001379
								177.367	0.096152	0.002453	0.001395
								179.875	0.096104	0.002485	0.001411
								182.383	0.096057	0.002517	0.001427
								184.892	0.096009	0.002549	0.001442
								187.4	0.0959	0.00258	0.0014

									62	1	58
								189.908	0.095914	0.002612	0.001473
								192.417	0.095867	0.002644	0.001489
								194.921	0.09582	0.002676	0.001504
								197.425	0.095774	0.002707	0.001519
								199.929	0.095727	0.002739	0.001534
								202.433	0.095681	0.00277	0.001549
								204.942	0.095634	0.002802	0.001564
								207.45	0.095588	0.002833	0.001579
								209.958	0.095542	0.002864	0.001594
								212.467	0.095496	0.002895	0.001608
								214.975	0.095451	0.002927	0.001623
								217.483	0.095405	0.002958	0.001637
								219.992	0.09536	0.002989	0.001652
								222.5	0.095314	0.00302	0.001666
								225.008	0.095269	0.003051	0.00168
								227.517	0.095224	0.003081	0.001694
								230.025	0.095179	0.003112	0.001708
								232.533	0.095135	0.003143	0.001722
								235.042	0.09509	0.003174	0.001736
								237.55	0.095046	0.003204	0.00175
								240.058	0.095002	0.003235	0.001764
								242.567	0.094958	0.003265	0.001777
								245.075	0.094914	0.003295	0.001791
								247.583	0.09487	0.003326	0.001804
								250.092	0.094826	0.003356	0.001818
								252.6	0.094783	0.003386	0.001831
								255.108	0.09474	0.003416	0.001844
								257.617	0.094696	0.003446	0.001857
								260.125	0.094653	0.003476	0.00187
								262.633	0.09461	0.003506	0.001883

								265.142	0.094568	0.003536	0.001896
								267.65	0.094525	0.003566	0.001909
								270.158	0.094482	0.003596	0.001922
								272.667	0.09444	0.003626	0.001934
								275.171	0.094398	0.003655	0.001947
								277.675	0.094356	0.003685	0.001959
								280.179	0.094314	0.003714	0.001972
								282.683	0.094272	0.003744	0.001984
								285.192	0.094231	0.003773	0.001996
								287.7	0.094189	0.003802	0.002009
								290.208	0.094148	0.003832	0.002021
								292.717	0.094106	0.003861	0.002033
								295.225	0.094065	0.00389	0.002045
								297.733	0.094024	0.003919	0.002057
								300.242	0.093983	0.003948	0.002069
								302.75	0.093943	0.003977	0.00208
								305.258	0.093902	0.004006	0.002092
								307.767	0.093862	0.004035	0.002104
								310.275	0.093821	0.004064	0.002115
								312.783	0.093781	0.004092	0.002127
								315.292	0.093741	0.004121	0.002138
								317.8	0.093701	0.00415	0.002149
								320.308	0.093661	0.004178	0.002161
								322.817	0.093621	0.004207	0.002172
								325.325	0.093582	0.004235	0.002183
								327.833	0.093542	0.004264	0.002194
								330.342	0.093503	0.004292	0.002205
								332.85	0.093464	0.00432	0.002216
								335.358	0.093425	0.004349	0.002227
								337.867	0.093386	0.004377	0.002238
								340.375	0.093347	0.004405	0.002248

								342.883	0.093308	0.004433	0.002259
								345.392	0.09327	0.004461	0.00227
								347.9	0.093231	0.004489	0.00228
								350.408	0.093193	0.004517	0.00229
								352.917	0.093155	0.004545	0.002301
								355.421	0.093116	0.004572	0.002311
								357.925	0.093079	0.0046	0.002321
								360.429	0.093041	0.004628	0.002332
								362.933	0.093003	0.004655	0.002342
								365.442	0.092965	0.004683	0.002352
								367.95	0.092928	0.00471	0.002362
								370.458	0.09289	0.004738	0.002372
								372.967	0.092853	0.004765	0.002382
								375.475	0.092816	0.004793	0.002391
								377.983	0.092779	0.00482	0.002401
								380.492	0.092742	0.004847	0.002411
								383	0.092705	0.004874	0.002421
								385.508	0.092669	0.004901	0.00243
								388.017	0.092632	0.004928	0.00244
								390.525	0.092595	0.004955	0.002449
								393.033	0.092559	0.004982	0.002459
								395.542	0.092523	0.005009	0.002468
								398.05	0.092487	0.005036	0.002477
								400.558	0.092451	0.005063	0.002486
								403.067	0.092415	0.00509	0.002496
								405.575	0.092379	0.005116	0.002505
								408.083	0.092343	0.005143	0.002514
								410.592	0.092308	0.00517	0.002523
								413.1	0.092272	0.005196	0.002532
								415.608	0.092237	0.005223	0.00254
								418.117	0.092202	0.005249	0.002549

								420.625	0.092166	0.005276	0.002558
								423.133	0.092131	0.005302	0.002567
								425.642	0.092096	0.005328	0.002575
								428.15	0.092062	0.005354	0.002584
								430.658	0.092027	0.005381	0.002593
								433.167	0.091992	0.005407	0.002601
								435.671	0.091958	0.005433	0.00261
								438.175	0.091923	0.005459	0.002618
								440.679	0.091889	0.005485	0.002626
								443.183	0.091855	0.005511	0.002634
								445.692	0.091821	0.005536	0.002643
								448.2	0.091787	0.005562	0.002651
								450.708	0.091753	0.005588	0.002659
								453.217	0.091719	0.005614	0.002667
								455.725	0.091686	0.005639	0.002675
								458.233	0.091652	0.005665	0.002683
								460.742	0.091618	0.005691	0.002691
								463.25	0.091585	0.005716	0.002699
								465.758	0.091552	0.005742	0.002707
								468.267	0.091519	0.005767	0.002714
								470.775	0.091486	0.005792	0.002722
								473.283	0.091453	0.005818	0.00273
								475.792	0.09142	0.005843	0.002737
								478.3	0.091387	0.005868	0.002745
								480.808	0.091354	0.005893	0.002752
								483.317	0.091322	0.005919	0.00276
								485.825	0.091289	0.005944	0.002767
								488.333	0.091257	0.005969	0.002775
								490.842	0.091224	0.005994	0.002782
								493.35	0.091192	0.006019	0.002789
								495.858	0.09116	0.006043	0.002797

								498.367	0.091128	0.006068	0.002804
								500.875	0.091096	0.006093	0.002811
								503.383	0.091064	0.006118	0.002818
								505.887	0.091033	0.006142	0.002825
								508.392	0.091001	0.006167	0.002832
								510.896	0.09097	0.006192	0.002839
								513.4	0.090938	0.006216	0.002846
								515.908	0.090907	0.006241	0.002853
								518.417	0.090875	0.006265	0.00286
								520.925	0.090844	0.006289	0.002866
								523.433	0.090813	0.006314	0.002873
								525.942	0.090782	0.006338	0.00288
								528.45	0.090751	0.006362	0.002886
								530.958	0.09072	0.006387	0.002893
								533.467	0.09069	0.006411	0.002899
								535.975	0.090659	0.006435	0.002906
								538.483	0.090629	0.006459	0.002912
								540.992	0.090598	0.006483	0.002919
								543.5	0.090568	0.006507	0.002925
								546.008	0.090537	0.006531	0.002932
								548.517	0.090507	0.006555	0.002938
								551.025	0.090477	0.006579	0.002944
								553.533	0.090447	0.006603	0.00295
								556.042	0.090417	0.006626	0.002957
								558.55	0.090387	0.00665	0.002963
								561.058	0.090358	0.006674	0.002969
								563.567	0.090328	0.006697	0.002975
								566.075	0.090298	0.006721	0.002981
								568.583	0.090269	0.006744	0.002987
								571.092	0.090239	0.006768	0.002993
								573.6	0.09021	0.006791	0.002999

								576.108	0.090181	0.006815	0.003004
								578.617	0.090152	0.006838	0.00301
								581.125	0.090122	0.006862	0.003016
								583.633	0.090093	0.006885	0.003022
								586.137	0.090065	0.006908	0.003027
								588.642	0.090036	0.006931	0.003033
								591.146	0.090007	0.006954	0.003039
								593.65	0.089978	0.006977	0.003044
								596.158	0.08995	0.007	0.00305
								598.667	0.089921	0.007023	0.003055
								601.175	0.089893	0.007046	0.003061
								603.683	0.089865	0.007069	0.003066
								606.192	0.089836	0.007092	0.003072
								608.7	0.089808	0.007115	0.003077
								611.208	0.08978	0.007138	0.003082
								613.717	0.089752	0.00716	0.003088
								616.225	0.089724	0.007183	0.003093
								618.733	0.089696	0.007206	0.003098
								621.242	0.089668	0.007228	0.003103
								623.75	0.089641	0.007251	0.003108
								626.258	0.089613	0.007274	0.003114
								628.767	0.089585	0.007296	0.003119
								631.275	0.089558	0.007319	0.003124
								633.783	0.08953	0.007341	0.003129
								636.292	0.089503	0.007363	0.003134
								638.8	0.089476	0.007386	0.003139
								641.308	0.089449	0.007408	0.003143
								643.817	0.089422	0.00743	0.003148
								646.325	0.089395	0.007452	0.003153
								648.833	0.089368	0.007475	0.003158
								651.342	0.089341	0.007497	0.003163

								653.85	0.089314	0.007519	0.003167
								656.358	0.089287	0.007541	0.003172
								658.867	0.08926	0.007563	0.003177
								661.375	0.089234	0.007585	0.003181
								663.883	0.089207	0.007607	0.003186
								666.387	0.089181	0.007628	0.003191
								668.892	0.089155	0.00765	0.003195
								671.396	0.089128	0.007672	0.0032
								673.9	0.089102	0.007694	0.003204
								676.408	0.089076	0.007715	0.003209
								678.917	0.08905	0.007737	0.003213
								681.425	0.089024	0.007759	0.003217
								683.933	0.088998	0.00778	0.003222
								686.442	0.088972	0.007802	0.003226
								688.95	0.088946	0.007823	0.00323
								691.458	0.088921	0.007845	0.003235
								693.967	0.088895	0.007866	0.003239
								696.475	0.088869	0.007888	0.003243
								698.983	0.088844	0.007909	0.003247
								701.492	0.088818	0.00793	0.003251
								704	0.088793	0.007952	0.003255
								706.508	0.088768	0.007973	0.00326
								709.017	0.088743	0.007994	0.003264
								711.525	0.088717	0.008015	0.003268
								714.033	0.088692	0.008036	0.003272
								716.542	0.088667	0.008057	0.003276
								719.05	0.088642	0.008078	0.00328
								721.558	0.088617	0.008099	0.003283
								724.067	0.088593	0.00812	0.003287
								726.575	0.088568	0.008141	0.003291
								729.083	0.088543	0.008162	0.003295

								731.592	0.088518	0.008183	0.003299
								734.1	0.088494	0.008204	0.003303
								736.608	0.088469	0.008224	0.003306
								739.117	0.088445	0.008245	0.00331
								741.625	0.088421	0.008266	0.003314
								744.133	0.088396	0.008286	0.003317
								746.637	0.088372	0.008307	0.003321
								749.142	0.088348	0.008328	0.003325
								751.646	0.088324	0.008348	0.003328
								754.15	0.0883	0.008369	0.003332
								756.658	0.088276	0.008389	0.003335
								759.167	0.088252	0.008409	0.003339
								761.675	0.088228	0.00843	0.003342
								764.183	0.088204	0.00845	0.003346
								766.692	0.08818	0.00847	0.003349
								769.2	0.088157	0.008491	0.003352
								771.708	0.088133	0.008511	0.003356
								774.217	0.08811	0.008531	0.003359
								776.725	0.088086	0.008551	0.003363
								779.233	0.088063	0.008571	0.003366
								781.742	0.088039	0.008592	0.003369
								784.25	0.088016	0.008612	0.003372
								786.758	0.087993	0.008632	0.003376
								789.267	0.08797	0.008652	0.003379
								791.775	0.087947	0.008672	0.003382
								794.283	0.087923	0.008691	0.003385
								796.792	0.0879	0.008711	0.003388
								799.3	0.087878	0.008731	0.003391
								801.808	0.087855	0.008751	0.003394
								804.317	0.087832	0.008771	0.003398
								806.825	0.087809	0.00879	0.003401

								809.333	0.087786	0.00881	0.003404
								811.842	0.087764	0.00883	0.003407
								814.35	0.087741	0.008849	0.00341
								816.858	0.087719	0.008869	0.003412
								819.367	0.087696	0.008888	0.003415
								821.875	0.087674	0.008908	0.003418
								824.383	0.087651	0.008927	0.003421
								826.887	0.087629	0.008947	0.003424
								829.392	0.087607	0.008966	0.003427
								831.896	0.087585	0.008986	0.00343
								834.4	0.087563	0.009005	0.003432
								836.908	0.087541	0.009024	0.003435
								839.417	0.087519	0.009043	0.003438
								841.925	0.087497	0.009063	0.003441
								844.433	0.087475	0.009082	0.003443
								846.942	0.087453	0.009101	0.003446
								849.45	0.087431	0.00912	0.003449
								851.958	0.087409	0.009139	0.003451
								854.467	0.087388	0.009158	0.003454
								856.975	0.087366	0.009177	0.003457
								859.483	0.087345	0.009196	0.003459
								861.992	0.087323	0.009215	0.003462
								864.5	0.087302	0.009234	0.003464
								867.008	0.08728	0.009253	0.003467
								869.517	0.087259	0.009272	0.003469
								872.025	0.087238	0.009291	0.003472
								874.533	0.087216	0.009309	0.003474
								877.042	0.087195	0.009328	0.003477
								879.55	0.087174	0.009347	0.003479
								882.058	0.087153	0.009366	0.003481
								884.567	0.087132	0.009384	0.003484

								887.075	0.087111	0.009403	0.003486
								889.583	0.08709	0.009421	0.003488
								892.092	0.087069	0.00944	0.003491
								894.6	0.087048	0.009458	0.003493
								897.108	0.087028	0.009477	0.003495
								899.617	0.087007	0.009495	0.003498
								902.125	0.086986	0.009514	0.0035
								904.633	0.086966	0.009532	0.003502
								907.142	0.086945	0.009551	0.003504
								909.65	0.086925	0.009569	0.003507
								912.158	0.086904	0.009587	0.003509
								914.667	0.086884	0.009605	0.003511

Appendix 4. Table of ^{1}H NMR integration and COPASI fitted data for **2c** → *trans*-fused- and *cis*-fused-**3c** (*p*-Me) in $CDCl_3$ with HFIP (12 equiv.)

Time (min)	2c (M)	Trans (M)	Cis |(M)	Fitted time	Fitted HA	Fitted trans	Fitted cis
0	0.1	0	0	0	0.1	0	0
3.5	0.016424	0.026734	0.056842	0.875	0.02071	0.044326	0.034964
4.58333	0.013617	0.02345	0.062933	1.75	0.016052	0.040084	0.043865
5.66667	0.01136	0.020577	0.068063	2.625	0.014319	0.034871	0.050809
6.73333	0.009782	0.017974	0.072244	3.5	0.012979	0.030276	0.056745
7.81667	0.008505	0.015485	0.076011	3.77083	0.012597	0.02899	0.058413
8.9	0.007347	0.013796	0.078857	4.04167	0.012228	0.027764	0.060008
9.98333	0.006408	0.012079	0.081512	4.3125	0.011871	0.026596	0.061533
11.0667	0.00583	0.009629	0.084542	4.58333	0.011524	0.025483	0.062993
12.1333	0.005333	0.007977	0.08669	4.85417	0.011189	0.024422	0.064389
13.2167	0.004678	0.006956	0.088366	5.125	0.010864	0.023411	0.065725
14.3	0.0039	0.006941	0.089158	5.39583	0.010549	0.022448	0.067003
15.3833	0.003372	0.006531	0.090097	5.66667	0.010244	0.021529	0.068227
16.4667	0.003033	0.0061	0.090867	5.93333	0.009953	0.020667	0.06938
17.5333	0.002604	0.005754	0.091642	6.2	0.009671	0.019844	0.070485
18.6167	0.002382	0.005433	0.092186	6.46667	0.009397	0.019059	0.071544
19.7	0.001945	0.005082	0.092973	6.73333	0.009132	0.018309	0.072559
20.7833	0.001798	0.004648	0.093555	7.00417	0.008872	0.017583	0.073546
21.8667	0.001687	0.004247	0.094066	7.275	0.008619	0.01689	0.074491
22.9333	0.001449	0.004112	0.094439	7.54583	0.008375	0.016228	0.075397
24.0167	0.001343	0.002835	0.095822	7.81667	0.008138	0.015597	0.076264
25.1	0.001226	0.003909	0.094865	8.0875	0.007909	0.014995	0.077095
26.1833	0.001125	0.003777	0.095098	8.35833	0.007688	0.014421	0.077892
27.2667	0.001039	0.003653	0.095308	8.62917	0.007473	0.013872	0.078655
28.3333	0.001051	0.002278	0.096672	8.9	0.007266	0.013348	0.079386
29.4167	0.000878	0.003435	0.095686	9.17083	0.007065	0.012848	0.080087
30.5	0.000868	0.003499	0.095633	9.44167	0.006871	0.01237	0.080759
31.5833	0.000693	0.003296	0.09601	9.7125	0.006683	0.011914	0.081403
32.6667	0.000594	0.002605	0.096801	9.98333	0.006501	0.011478	0.082021
33.7333	0.000649	0.003253	0.096099	10.2542	0.006325	0.011062	0.082613
34.8167	0.000546	0.002973	0.096481	10.525	0.006155	0.010664	0.083181
35.9	0.000546	0.002219	0.097235	10.7958	0.005991	0.010284	0.083725
36.9833	0.000624	0.001189	0.098188	11.0667	0.005832	0.00992	0.084247
38.05	0.0006	0.003035	0.096365	11.3333	0.005681	0.009578	0.084741
39.1333	0.00047	0.002942	0.096588	11.6	0.005535	0.009251	0.085214
40.2167	0.000306	0.003068	0.096627	11.8667	0.005393	0.008938	0.085669
41.3	0.000342	0.002837	0.096821	12.1333	0.005257	0.008638	0.086105
42.3833	0.000409	0.002885	0.096706	12.4042	0.005122	0.008347	0.086531
43.45	0.000318	0.00285	0.096832	12.675	0.004993	0.008068	0.086939
44.5333	0.000418	0.002922	0.09666	12.9458	0.004868	0.007802	0.087331
45.6167	0.000302	0.002984	0.096714	13.2167	0.004747	0.007547	0.087707

46.7	0.000353	0.002906	0.096741	13.4875	0.00463	0.007303	0.088067
47.7833	0.000392	0.002823	0.096785	13.7583	0.004517	0.007069	0.088414
48.85	0.000357	0.002676	0.096967	14.0292	0.004408	0.006846	0.088746
49.9333	0.000444	0.001497	0.098059	14.3	0.004303	0.006632	0.089065
51.0167	0.000351	0.002712	0.096937	14.5708	0.004201	0.006427	0.089372
52.1	0.000465	0.000872	0.098664	14.8417	0.004103	0.006231	0.089666
53.1833	0.000422	0.002745	0.096833	15.1125	0.004008	0.006043	0.089948
54.25	0.000321	0.002827	0.096852	15.3833	0.003917	0.005864	0.090219
55.3333	0.000426	0.002858	0.096716	15.6542	0.003829	0.005691	0.09048
56.4167	0.000239	0.002672	0.097089	15.925	0.003744	0.005526	0.09073
57.5	0.000245	0.002876	0.096879	16.1958	0.003662	0.005368	0.09097
58.5833	0.000343	0.002938	0.096719	16.4667	0.003583	0.005217	0.091201
59.65	0.000283	0.002834	0.096884	16.7333	0.003507	0.005074	0.091419
60.7333	0.000297	0.002788	0.096916	17	0.003435	0.004937	0.091628
61.8167	0.000252	0.002615	0.097133	17.2667	0.003365	0.004805	0.09183
62.9	0.000349	0.002821	0.09683	17.5333	0.003297	0.004679	0.092024
63.9833	0.000304	0.00274	0.096956	17.8042	0.003231	0.004557	0.092213
65.05	0.000412	0.002697	0.096891	18.075	0.003167	0.004439	0.092394
66.1333	0.000223	0.003156	0.096621	18.3458	0.003105	0.004326	0.092569
67.2167	0.000294	0.002687	0.097019	18.6167	0.003046	0.004218	0.092736
				18.8875	0.002989	0.004114	0.092897
				19.1583	0.002933	0.004014	0.093052
				19.4292	0.00288	0.003919	0.093201
				19.7	0.002829	0.003827	0.093344
				19.9708	0.00278	0.003739	0.093481
				20.2417	0.002732	0.003655	0.093613
				20.5125	0.002686	0.003574	0.09374
				20.7833	0.002642	0.003497	0.093862
				21.0542	0.002599	0.003422	0.093979
				21.325	0.002558	0.003351	0.094091
				21.5958	0.002518	0.003282	0.0942
				21.8667	0.00248	0.003216	0.094304
				22.1333	0.002444	0.003154	0.094402
				22.4	0.002409	0.003094	0.094497
				22.6667	0.002375	0.003037	0.094588
				22.9333	0.002343	0.002982	0.094675
				23.2042	0.002311	0.002928	0.094761
				23.475	0.002281	0.002876	0.094843
				23.7458	0.002251	0.002827	0.094922
				24.0167	0.002223	0.002779	0.094998
				24.2875	0.002196	0.002733	0.095071
				24.5583	0.002169	0.002689	0.095141
				24.8292	0.002144	0.002647	0.095209
				25.1	0.00212	0.002606	0.095274

				25.3708	0.002096	0.002567	0.095336
				25.6417	0.002074	0.00253	0.095396
				25.9125	0.002052	0.002494	0.095454
				26.1833	0.002031	0.00246	0.095509
				26.4542	0.002011	0.002426	0.095563
				26.725	0.001992	0.002395	0.095614
				26.9958	0.001973	0.002364	0.095663
				27.2667	0.001955	0.002334	0.095711
				27.5333	0.001938	0.002307	0.095756
				27.8	0.001921	0.00228	0.095799
				28.0667	0.001906	0.002254	0.09584
				28.3333	0.00189	0.002229	0.09588
				28.6042	0.001875	0.002205	0.095919
				28.875	0.001861	0.002182	0.095957
				29.1458	0.001847	0.00216	0.095993
				29.4167	0.001834	0.002138	0.096028
				29.6875	0.001821	0.002118	0.096061
				29.9583	0.001809	0.002098	0.096093
				30.2292	0.001797	0.002079	0.096124
				30.5	0.001786	0.00206	0.096154
				30.7708	0.001775	0.002043	0.096183
				31.0417	0.001764	0.002026	0.09621
				31.3125	0.001754	0.00201	0.096237
				31.5833	0.001744	0.001994	0.096262
				31.8542	0.001735	0.001979	0.096286
				32.125	0.001726	0.001964	0.09631
				32.3958	0.001717	0.001951	0.096332
				32.6667	0.001709	0.001937	0.096354
				32.9333	0.001701	0.001925	0.096375
				33.2	0.001693	0.001912	0.096395
				33.4667	0.001686	0.001901	0.096414
				33.7333	0.001679	0.001889	0.096432
				34.0042	0.001672	0.001878	0.09645
				34.275	0.001665	0.001868	0.096467
				34.5458	0.001659	0.001858	0.096484
				34.8167	0.001652	0.001848	0.0965
				35.0875	0.001646	0.001839	0.096515
				35.3583	0.001641	0.001829	0.09653
				35.6292	0.001635	0.001821	0.096544
				35.9	0.00163	0.001812	0.096558
				36.1708	0.001625	0.001804	0.096571
				36.4417	0.00162	0.001797	0.096583
				36.7125	0.001615	0.001789	0.096596
				36.9833	0.001611	0.001782	0.096607

				37.25	0.001606	0.001775	0.096618
				37.5167	0.001602	0.001769	0.096629
				37.7833	0.001598	0.001763	0.096639
				38.05	0.001595	0.001757	0.096649
				38.3208	0.001591	0.001751	0.096659
				38.5917	0.001587	0.001745	0.096668
				38.8625	0.001584	0.00174	0.096677
				39.1333	0.00158	0.001734	0.096685
				39.4042	0.001577	0.001729	0.096694
				39.675	0.001574	0.001724	0.096702
				39.9458	0.001571	0.00172	0.096709
				40.2167	0.001568	0.001715	0.096716
				40.4875	0.001566	0.001711	0.096724
				40.7583	0.001563	0.001707	0.09673
				41.0292	0.00156	0.001703	0.096737
				41.3	0.001558	0.001699	0.096743
				41.5708	0.001556	0.001695	0.096749
				41.8417	0.001553	0.001692	0.096755
				42.1125	0.001551	0.001688	0.096761
				42.3833	0.001549	0.001685	0.096766
				42.65	0.001547	0.001682	0.096771
				42.9167	0.001545	0.001679	0.096776
				43.1833	0.001543	0.001676	0.096781
				43.45	0.001542	0.001673	0.096785
				43.7208	0.00154	0.001671	0.09679
				43.9917	0.001538	0.001668	0.096794
				44.2625	0.001537	0.001666	0.096798
				44.5333	0.001535	0.001663	0.096802
				44.8042	0.001534	0.001661	0.096806
				45.075	0.001532	0.001659	0.096809
				45.3458	0.001531	0.001656	0.096813
				45.6167	0.001529	0.001654	0.096816
				45.8875	0.001528	0.001652	0.09682
				46.1583	0.001527	0.001651	0.096823
				46.4292	0.001526	0.001649	0.096826
				46.7	0.001525	0.001647	0.096829
				46.9708	0.001524	0.001645	0.096831
				47.2417	0.001522	0.001644	0.096834
				47.5125	0.001521	0.001642	0.096837
				47.7833	0.001521	0.001641	0.096839
				48.05	0.00152	0.001639	0.096841
				48.3167	0.001519	0.001638	0.096844
				48.5833	0.001518	0.001636	0.096846
				48.85	0.001517	0.001635	0.096848

				49.1208	0.001516	0.001634	0.09685
				49.3917	0.001515	0.001633	0.096852
				49.6625	0.001515	0.001632	0.096854
				49.9333	0.001514	0.00163	0.096856
				50.2042	0.001513	0.001629	0.096857
				50.475	0.001513	0.001628	0.096859
				50.7458	0.001512	0.001627	0.096861
				51.0167	0.001511	0.001626	0.096862
				51.2875	0.001511	0.001626	0.096864
				51.5583	0.00151	0.001625	0.096865
				51.8292	0.00151	0.001624	0.096867
				52.1	0.001509	0.001623	0.096868
				52.3708	0.001509	0.001622	0.096869
				52.6417	0.001508	0.001621	0.09687
				52.9125	0.001508	0.001621	0.096872
				53.1833	0.001507	0.00162	0.096873
				53.45	0.001507	0.001619	0.096874
				53.7167	0.001507	0.001619	0.096875
				53.9833	0.001506	0.001618	0.096876
				54.25	0.001506	0.001618	0.096877
				54.5208	0.001505	0.001617	0.096878
				54.7917	0.001505	0.001616	0.096879
				55.0625	0.001505	0.001616	0.096879
				55.3333	0.001504	0.001615	0.09688
				55.6042	0.001504	0.001615	0.096881
				55.875	0.001504	0.001614	0.096882
				56.1458	0.001503	0.001614	0.096883
				56.4167	0.001503	0.001614	0.096883
				56.6875	0.001503	0.001613	0.096884
				56.9583	0.001503	0.001613	0.096885
				57.2292	0.001502	0.001612	0.096885
				57.5	0.001502	0.001612	0.096886
				57.7708	0.001502	0.001612	0.096887
				58.0417	0.001502	0.001611	0.096887
				58.3125	0.001501	0.001611	0.096888
				58.5833	0.001501	0.001611	0.096888
				58.85	0.001501	0.00161	0.096889
				59.1167	0.001501	0.00161	0.096889
				59.3833	0.001501	0.00161	0.09689
				59.65	0.001501	0.001609	0.09689
				59.9208	0.0015	0.001609	0.09689
				60.1917	0.0015	0.001609	0.096891
				60.4625	0.0015	0.001609	0.096891
				60.7333	0.0015	0.001608	0.096892

				61.0042	0.0015	0.001608	0.096892
				61.275	0.0015	0.001608	0.096892
				61.5458	0.001499	0.001608	0.096893
				61.8167	0.001499	0.001608	0.096893
				62.0875	0.001499	0.001607	0.096893
				62.3583	0.001499	0.001607	0.096894
				62.6292	0.001499	0.001607	0.096894
				62.9	0.001499	0.001607	0.096894
				63.1708	0.001499	0.001607	0.096895
				63.4417	0.001499	0.001607	0.096895
				63.7125	0.001499	0.001606	0.096895
				63.9833	0.001498	0.001606	0.096895
				64.25	0.001498	0.001606	0.096896
				64.5167	0.001498	0.001606	0.096896
				64.7833	0.001498	0.001606	0.096896
				65.05	0.001498	0.001606	0.096896
				65.3208	0.001498	0.001606	0.096896
				65.5917	0.001498	0.001605	0.096897
				65.8625	0.001498	0.001605	0.096897
				66.1333	0.001498	0.001605	0.096897
				66.4042	0.001498	0.001605	0.096897
				66.675	0.001498	0.001605	0.096897
				66.9458	0.001498	0.001605	0.096897
				67.2167	0.001498	0.001605	0.096898
				67.2167	0.001498	0.001605	0.096898

Appendix 5. Table of ^{1}H NMR integration and COPASI fitted data for **2f** → *trans*-fused- and *cis*-fused-**3f** (*p*-Cl) in $CDCl_3$ with HFIP (12 equiv.)

Time (min)	2f (M)	Trans (M)	Cis (M)	Fitted time (min)	Fitted HA (M)	Fitted trans (M)	Fitted cis (M)
0	0.1	0	0	0	0.1	0	0
3.5	0.093467	0.005521	0.001013	0.875	0.098298	0.001343	0.000359
4.58333	0.091561	0.007061	0.001378	1.75	0.096656	0.002638	0.000706
5.66667	0.089993	0.008324	0.001684	2.625	0.09507	0.003887	0.001043
6.73333	0.088236	0.009844	0.00192	3.5	0.093538	0.005091	0.00137
7.81667	0.086656	0.01112	0.002225	3.77083	0.093075	0.005455	0.00147
8.9	0.085167	0.012246	0.002587	4.04167	0.092616	0.005816	0.001568
9.98333	0.083713	0.013467	0.00282	4.3125	0.092162	0.006172	0.001666
11.05	0.082383	0.014542	0.003075	4.58333	0.091713	0.006524	0.001762
12.1333	0.081076	0.015553	0.003372	4.85417	0.091269	0.006873	0.001858
13.2167	0.079731	0.016665	0.003604	5.125	0.090829	0.007218	0.001953
14.3	0.078505	0.01761	0.003885	5.39583	0.090393	0.007559	0.002047
15.3833	0.077459	0.018412	0.004128	5.66667	0.089962	0.007897	0.002141
16.45	0.076207	0.019429	0.004364	5.93333	0.089542	0.008226	0.002232
17.5333	0.075157	0.020235	0.004608	6.2	0.089127	0.008551	0.002322
18.6167	0.073948	0.021194	0.004858	6.46667	0.088715	0.008873	0.002412
19.7	0.073113	0.021871	0.005016	6.73333	0.088307	0.009192	0.002501
20.7833	0.072105	0.022656	0.00524	7.00417	0.087898	0.009512	0.00259
21.85	0.071249	0.023334	0.005417	7.275	0.087492	0.009829	0.002679
22.9333	0.070205	0.024114	0.005682	7.54583	0.08709	0.010143	0.002767
24.0167	0.069367	0.024801	0.005831	7.81667	0.086693	0.010453	0.002854
25.1	0.068506	0.025421	0.006073	8.0875	0.086299	0.01076	0.002941
26.1833	0.067654	0.026093	0.006254	8.35833	0.085909	0.011064	0.003027
27.25	0.067026	0.026636	0.006338	8.62917	0.085523	0.011365	0.003112
28.3333	0.066127	0.027278	0.006595	8.9	0.085141	0.011663	0.003196
29.4167	0.065575	0.027632	0.006793	9.17083	0.084763	0.011957	0.00328
30.5	0.064646	0.028397	0.006956	9.44167	0.084388	0.012249	0.003363
31.5833	0.063954	0.029001	0.007046	9.7125	0.084017	0.012538	0.003445
32.65	0.063237	0.029437	0.007326	9.98333	0.083649	0.012823	0.003527
33.7333	0.062614	0.029886	0.0075	10.25	0.083291	0.013102	0.003607
34.8167	0.061956	0.030417	0.007627	10.5167	0.082936	0.013378	0.003686
38.1667	0.059993	0.031878	0.008129	10.7833	0.082584	0.013651	0.003765
48.2	0.055288	0.035223	0.009489	11.05	0.082236	0.013921	0.003843
58.2167	0.051466	0.037813	0.010721	11.3208	0.081886	0.014193	0.003922
68.25	0.048577	0.03959	0.011833	11.5917	0.081538	0.014462	0.004
78.2833	0.046244	0.040937	0.012819	11.8625	0.081195	0.014728	0.004077
88.3167	0.044178	0.042083	0.013739	12.1333	0.080854	0.014992	0.004154
98.35	0.042506	0.042766	0.014728	12.4042	0.080517	0.015253	0.004231
108.383	0.041068	0.043204	0.015729	12.675	0.080182	0.015511	0.004306
118.417	0.039887	0.043673	0.01644	12.9458	0.079851	0.015767	0.004382

128.45	0.038866	0.043878	0.017256	13.2167	0.079523	0.016021	0.004456
138.483	0.037957	0.044013	0.01803	13.4875	0.079198	0.016272	0.00453
148.5	0.037232	0.044033	0.018735	13.7583	0.078876	0.01652	0.004604
158.533	0.036467	0.044027	0.019506	14.0292	0.078557	0.016766	0.004677
168.567	0.03589	0.043803	0.020307	14.3	0.078241	0.01701	0.004749
178.6	0.035248	0.043688	0.021064	14.5708	0.077928	0.017251	0.004821
188.633	0.034819	0.043449	0.021732	14.8417	0.077617	0.01749	0.004893
198.667	0.034418	0.043233	0.022349	15.1125	0.07731	0.017726	0.004964
208.7	0.033924	0.043033	0.023043	15.3833	0.077005	0.017961	0.005034
218.733	0.033692	0.042637	0.023671	15.65	0.076708	0.018189	0.005103
228.75	0.033161	0.042388	0.024451	15.9167	0.076413	0.018416	0.005171
238.783	0.032981	0.041989	0.02503	16.1833	0.076121	0.01864	0.005239
248.817	0.032631	0.041638	0.025731	16.45	0.075831	0.018862	0.005307
258.85	0.032303	0.041269	0.026427	16.7208	0.07554	0.019085	0.005375
268.883	0.03209	0.040829	0.027081	16.9917	0.075251	0.019307	0.005442
278.917	0.031866	0.040556	0.027579	17.2625	0.074965	0.019526	0.00551
288.95	0.031613	0.040067	0.028319	17.5333	0.074681	0.019743	0.005576
298.983	0.031313	0.0398	0.028887	17.8042	0.0744	0.019958	0.005642
309.017	0.031101	0.039511	0.029388	18.075	0.074121	0.020171	0.005708
319.033	0.030922	0.039032	0.030046	18.3458	0.073845	0.020382	0.005773
329.067	0.030748	0.038642	0.03061	18.6167	0.073571	0.020591	0.005838
339.1	0.0305	0.038242	0.031258	18.8875	0.073299	0.020798	0.005903
349.133	0.030297	0.037945	0.031758	19.1583	0.07303	0.021003	0.005967
359.167	0.03009	0.037607	0.032303	19.4292	0.072763	0.021206	0.006031
369.2	0.029877	0.037093	0.03303	19.7	0.072499	0.021408	0.006094
379.233	0.02972	0.036938	0.033342	19.9708	0.072236	0.021607	0.006157
389.267	0.029467	0.036556	0.033977	20.2417	0.071976	0.021805	0.006219
399.283	0.029231	0.036154	0.034615	20.5125	0.071719	0.022001	0.006281
409.317	0.029016	0.035822	0.035162	20.7833	0.071463	0.022194	0.006343
419.35	0.028972	0.035416	0.035612	21.05	0.071214	0.022384	0.006403
429.383	0.028717	0.035092	0.036191	21.3167	0.070966	0.022571	0.006463
439.417	0.028554	0.034599	0.036847	21.5833	0.070721	0.022757	0.006522
449.45	0.028475	0.034377	0.037148	21.85	0.070478	0.022941	0.006581
459.483	0.028202	0.034043	0.037755	22.1208	0.070233	0.023126	0.006641
469.517	0.028097	0.033723	0.03818	22.3917	0.06999	0.02331	0.006701
479.55	0.027865	0.033399	0.038736	22.6625	0.069749	0.023492	0.00676
489.567	0.027627	0.033076	0.039297	22.9333	0.06951	0.023672	0.006818
499.6	0.027437	0.032775	0.039788	23.2042	0.069273	0.023851	0.006876
509.633	0.027187	0.032492	0.040322	23.475	0.069038	0.024028	0.006934
519.667	0.027044	0.03221	0.040746	23.7458	0.068805	0.024203	0.006992
529.7	0.026939	0.031764	0.041297	24.0167	0.068574	0.024377	0.007049
539.733	0.026863	0.031448	0.04169	24.2875	0.068345	0.024549	0.007106
549.767	0.026567	0.031161	0.042272	24.5583	0.068118	0.02472	0.007163
559.8	0.026382	0.030981	0.042637	24.8292	0.067892	0.024889	0.007219

569.833	0.02621	0.030692	0.043097	25.1	0.067669	0.025057	0.007275
579.85	0.026137	0.030321	0.043542	25.3708	0.067447	0.025223	0.00733
589.883	0.025966	0.030024	0.044011	25.6417	0.067227	0.025387	0.007386
599.917	0.025695	0.029889	0.044416	25.9125	0.067009	0.025551	0.007441
609.95	0.025697	0.029416	0.044887	26.1833	0.066793	0.025712	0.007495
619.983	0.025511	0.029097	0.045392	26.45	0.066581	0.02587	0.007549
630.017	0.025242	0.028907	0.04585	26.7167	0.066372	0.026027	0.007602
640.05	0.025184	0.028679	0.046137	26.9833	0.066164	0.026182	0.007655
650.067	0.024924	0.028386	0.04669	27.25	0.065957	0.026335	0.007707
660.1	0.024846	0.028077	0.047077	27.5208	0.06575	0.02649	0.00776
670.133	0.024633	0.027825	0.047542	27.7917	0.065543	0.026643	0.007813
680.167	0.024564	0.027527	0.047909	28.0625	0.065339	0.026795	0.007866
690.2	0.024315	0.02736	0.048325	28.3333	0.065136	0.026946	0.007918
700.233	0.024141	0.027019	0.04884	28.6042	0.064935	0.027096	0.00797
710.267	0.02403	0.026777	0.049194	28.875	0.064735	0.027244	0.008022
720.3	0.023798	0.026613	0.04959	29.1458	0.064537	0.02739	0.008073
730.333	0.023674	0.026438	0.049888	29.4167	0.06434	0.027536	0.008124
740.35	0.02362	0.026074	0.050306	29.6875	0.064145	0.02768	0.008175
750.383	0.023436	0.02589	0.050674	29.9583	0.063952	0.027823	0.008226
760.417	0.023354	0.02562	0.051026	30.2292	0.06376	0.027964	0.008276
770.45	0.023227	0.025391	0.051383	30.5	0.063569	0.028105	0.008326
780.483	0.023011	0.025112	0.051877	30.7708	0.06338	0.028244	0.008376
790.517	0.022811	0.024947	0.052242	31.0417	0.063193	0.028382	0.008425
800.55	0.022692	0.024626	0.052681	31.3125	0.063007	0.028519	0.008475
810.583	0.022507	0.024489	0.053004	31.5833	0.062822	0.028655	0.008524
820.6	0.02255	0.024216	0.053234	31.85	0.062642	0.028787	0.008572
830.633	0.022258	0.024054	0.053688	32.1167	0.062463	0.028918	0.008619
840.667	0.0222	0.023754	0.054046	32.3833	0.062285	0.029048	0.008667
850.7	0.022056	0.023485	0.054459	32.65	0.062109	0.029177	0.008714
860.733	0.021877	0.023287	0.054836	32.9208	0.061931	0.029307	0.008762
870.767	0.021684	0.023143	0.055173	33.1917	0.061755	0.029436	0.008809
880.8	0.021484	0.022965	0.055551	33.4625	0.06158	0.029564	0.008857
890.833	0.021397	0.022696	0.055908	33.7333	0.061406	0.02969	0.008904
900.85	0.02133	0.022461	0.05621	34.0042	0.061233	0.029816	0.008951
910.883	0.021192	0.02225	0.056558	34.275	0.061062	0.02994	0.008997
920.917	0.020943	0.022153	0.056905	34.5458	0.060893	0.030064	0.009044
930.95	0.020894	0.021926	0.05718	34.8167	0.060724	0.030186	0.00909
940.983	0.020777	0.021654	0.057569	35.6542	0.060211	0.030558	0.009231
951.017	0.020694	0.021454	0.057852	36.4917	0.05971	0.03092	0.009371
961.05	0.020537	0.021316	0.058147	37.3292	0.05922	0.031272	0.009508
971.083	0.020342	0.021125	0.058533	38.1667	0.058741	0.031616	0.009643
981.117	0.020311	0.020942	0.058747	40.675	0.05737	0.032592	0.010037
991.133	0.020115	0.020772	0.059113	43.1833	0.056089	0.033495	0.010416
1001.17	0.020031	0.020566	0.059403	45.6917	0.054888	0.034331	0.010781

1011.2	0.019968	0.020344	0.059689	48.2	0.053763	0.035105	0.011132
1021.23	0.019776	0.020166	0.060059	50.7042	0.052708	0.035821	0.011471
1031.27	0.01962	0.02005	0.06033	53.2083	0.051718	0.036484	0.011798
1041.3	0.019491	0.019861	0.060648	55.7125	0.050786	0.037099	0.012115
				58.2167	0.04991	0.037668	0.012423
				60.725	0.049082	0.038196	0.012722
				63.2333	0.048302	0.038685	0.013013
				65.7417	0.047566	0.039138	0.013296
				68.25	0.046871	0.039557	0.013573
				70.7583	0.046214	0.039944	0.013842
				73.2667	0.045592	0.040303	0.014105
				75.775	0.045004	0.040633	0.014363
				78.2833	0.044447	0.040939	0.014615
				80.7917	0.043919	0.04122	0.014861
				83.3	0.043418	0.041479	0.015103
				85.8083	0.042943	0.041717	0.015341
				88.3167	0.042491	0.041935	0.015574
				90.825	0.042062	0.042135	0.015803
				93.3333	0.041654	0.042318	0.016028
				95.8417	0.041266	0.042485	0.01625
				98.35	0.040896	0.042636	0.016468
				100.858	0.040544	0.042773	0.016683
				103.367	0.040208	0.042897	0.016895
				105.875	0.039888	0.043008	0.017105
				108.383	0.039582	0.043107	0.017311
				110.892	0.03929	0.043195	0.017515
				113.4	0.039011	0.043273	0.017717
				115.908	0.038744	0.04334	0.017916
				118.417	0.038489	0.043399	0.018113
				120.925	0.038244	0.043448	0.018308
				123.433	0.03801	0.043489	0.0185
				125.942	0.037786	0.043523	0.018691
				128.45	0.037571	0.043549	0.01888
				130.958	0.037364	0.043568	0.019068
				133.467	0.037165	0.043581	0.019253
				135.975	0.036975	0.043588	0.019438
				138.483	0.036791	0.043589	0.01962
				140.987	0.036615	0.043584	0.019801
				143.492	0.036446	0.043574	0.01998
				145.996	0.036282	0.04356	0.020158
				148.5	0.036125	0.04354	0.020335
				151.008	0.035973	0.043517	0.020511
				153.517	0.035826	0.043489	0.020686
				156.025	0.035684	0.043457	0.020859

				158.533	0.035547	0.043422	0.021031
				161.042	0.035414	0.043384	0.021203
				163.55	0.035285	0.043342	0.021373
				166.058	0.035161	0.043297	0.021542
				168.567	0.035041	0.043249	0.02171
				171.075	0.034924	0.043198	0.021878
				173.583	0.03481	0.043145	0.022044
				176.092	0.0347	0.04309	0.02221
				178.6	0.034593	0.043032	0.022375
				181.108	0.034489	0.042972	0.022539
				183.617	0.034388	0.04291	0.022702
				186.125	0.03429	0.042846	0.022864
				188.633	0.034194	0.042781	0.023026
				191.142	0.0341	0.042713	0.023187
				193.65	0.034009	0.042644	0.023347
				196.158	0.03392	0.042574	0.023506
				198.667	0.033833	0.042502	0.023665
				201.175	0.033748	0.042429	0.023823
				203.683	0.033665	0.042354	0.023981
				206.192	0.033584	0.042279	0.024138
				208.7	0.033504	0.042202	0.024294
				211.208	0.033426	0.042124	0.02445
				213.717	0.03335	0.042046	0.024605
				216.225	0.033275	0.041966	0.024759
				218.733	0.033201	0.041886	0.024913
				221.237	0.033129	0.041804	0.025066
				223.742	0.033058	0.041723	0.025219
				226.246	0.032989	0.04164	0.025371
				228.75	0.03292	0.041557	0.025523
				231.258	0.032853	0.041473	0.025674
				233.767	0.032786	0.041389	0.025825
				236.275	0.032721	0.041304	0.025975
				238.783	0.032657	0.041218	0.026125
				241.292	0.032593	0.041133	0.026275
				243.8	0.03253	0.041046	0.026424
				246.308	0.032469	0.04096	0.026572
				248.817	0.032407	0.040873	0.02672
				251.325	0.032347	0.040785	0.026868
				253.833	0.032288	0.040698	0.027015
				256.342	0.032229	0.04061	0.027161
				258.85	0.032171	0.040522	0.027308
				261.358	0.032113	0.040434	0.027453
				263.867	0.032056	0.040345	0.027599
				266.375	0.032	0.040257	0.027744

				268.883	0.031944	0.040168	0.027888
				271.392	0.031889	0.040079	0.028033
				273.9	0.031834	0.03999	0.028176
				276.408	0.03178	0.039901	0.02832
				278.917	0.031726	0.039812	0.028463
				281.425	0.031672	0.039722	0.028605
				283.933	0.031619	0.039633	0.028748
				286.442	0.031567	0.039544	0.028889
				288.95	0.031515	0.039454	0.029031
				291.458	0.031463	0.039365	0.029172
				293.967	0.031412	0.039276	0.029313
				296.475	0.031361	0.039187	0.029453
				298.983	0.03131	0.039097	0.029593
				301.492	0.03126	0.039008	0.029733
				304	0.031209	0.038919	0.029872
				306.508	0.03116	0.03883	0.030011
				309.017	0.03111	0.038741	0.030149
				311.521	0.031061	0.038652	0.030287
				314.025	0.031012	0.038563	0.030425
				316.529	0.030964	0.038474	0.030562
				319.033	0.030915	0.038386	0.030699
				321.542	0.030867	0.038297	0.030836
				324.05	0.030819	0.038208	0.030972
				326.558	0.030772	0.03812	0.031108
				329.067	0.030724	0.038032	0.031244
				331.575	0.030677	0.037944	0.03138
				334.083	0.03063	0.037856	0.031515
				336.592	0.030583	0.037768	0.03165
				339.1	0.030536	0.03768	0.031784
				341.608	0.03049	0.037592	0.031918
				344.117	0.030443	0.037505	0.032052
				346.625	0.030397	0.037418	0.032185
				349.133	0.030351	0.03733	0.032319
				351.642	0.030305	0.037243	0.032451
				354.15	0.03026	0.037156	0.032584
				356.658	0.030214	0.03707	0.032716
				359.167	0.030169	0.036983	0.032848
				361.675	0.030124	0.036897	0.032979
				364.183	0.030079	0.036811	0.033111
				366.692	0.030034	0.036725	0.033242
				369.2	0.029989	0.036639	0.033372
				371.708	0.029944	0.036553	0.033502
				374.217	0.0299	0.036468	0.033632
				376.725	0.029856	0.036383	0.033762

				379.233	0.029811	0.036297	0.033891
				381.742	0.029767	0.036213	0.034021
				384.25	0.029723	0.036128	0.034149
				386.758	0.029679	0.036043	0.034278
				389.267	0.029635	0.035959	0.034406
				391.771	0.029592	0.035875	0.034534
				394.275	0.029548	0.035791	0.034661
				396.779	0.029505	0.035707	0.034788
				399.283	0.029462	0.035624	0.034915
				401.792	0.029418	0.035541	0.035041
				404.3	0.029375	0.035457	0.035168
				406.808	0.029332	0.035374	0.035294
				409.317	0.029289	0.035292	0.035419
				411.825	0.029246	0.035209	0.035545
				414.333	0.029203	0.035127	0.03567
				416.842	0.029161	0.035044	0.035795
				419.35	0.029118	0.034962	0.03592
				421.858	0.029076	0.034881	0.036044
				424.367	0.029033	0.034799	0.036168
				426.875	0.028991	0.034718	0.036292
				429.383	0.028949	0.034636	0.036415
				431.892	0.028907	0.034555	0.036538
				434.4	0.028864	0.034474	0.036661
				436.908	0.028823	0.034394	0.036784
				439.417	0.028781	0.034313	0.036906
				441.925	0.028739	0.034233	0.037028
				444.433	0.028697	0.034153	0.03715
				446.942	0.028655	0.034073	0.037271
				449.45	0.028614	0.033994	0.037392
				451.958	0.028572	0.033914	0.037513
				454.467	0.028531	0.033835	0.037634
				456.975	0.02849	0.033756	0.037754
				459.483	0.028448	0.033677	0.037874
				461.992	0.028407	0.033599	0.037994
				464.5	0.028366	0.03352	0.038114
				467.008	0.028325	0.033442	0.038233
				469.517	0.028284	0.033364	0.038352
				472.025	0.028243	0.033286	0.038471
				474.533	0.028202	0.033209	0.038589
				477.042	0.028162	0.033131	0.038707
				479.55	0.028121	0.033054	0.038825
				482.054	0.02808	0.032977	0.038943
				484.558	0.02804	0.032901	0.03906
				487.063	0.027999	0.032824	0.039177

				489.567	0.027959	0.032748	0.039293
				492.075	0.027919	0.032672	0.03941
				494.583	0.027878	0.032596	0.039526
				497.092	0.027838	0.03252	0.039642
				499.6	0.027798	0.032444	0.039758
				502.108	0.027758	0.032369	0.039873
				504.617	0.027718	0.032294	0.039988
				507.125	0.027678	0.032219	0.040103
				509.633	0.027638	0.032144	0.040218
				512.142	0.027599	0.032069	0.040333
				514.65	0.027559	0.031995	0.040447
				517.158	0.027519	0.03192	0.040561
				519.667	0.02748	0.031846	0.040674
				522.175	0.02744	0.031772	0.040788
				524.683	0.027401	0.031699	0.040901
				527.192	0.027361	0.031625	0.041014
				529.7	0.027322	0.031552	0.041126
				532.208	0.027283	0.031479	0.041239
				534.717	0.027243	0.031406	0.041351
				537.225	0.027204	0.031333	0.041463
				539.733	0.027165	0.031261	0.041574
				542.242	0.027126	0.031188	0.041686
				544.75	0.027087	0.031116	0.041797
				547.258	0.027048	0.031044	0.041908
				549.767	0.027009	0.030972	0.042018
				552.275	0.026971	0.030901	0.042129
				554.783	0.026932	0.030829	0.042239
				557.292	0.026893	0.030758	0.042349
				559.8	0.026855	0.030687	0.042458
				562.308	0.026816	0.030616	0.042568
				564.817	0.026778	0.030546	0.042677
				567.325	0.026739	0.030475	0.042786
				569.833	0.026701	0.030405	0.042894
				572.337	0.026663	0.030335	0.043002
				574.842	0.026625	0.030265	0.04311
				577.346	0.026587	0.030195	0.043218
				579.85	0.026548	0.030126	0.043326
				582.358	0.02651	0.030057	0.043433
				584.867	0.026472	0.029987	0.04354
				587.375	0.026435	0.029918	0.043647
				589.883	0.026397	0.02985	0.043754
				592.392	0.026359	0.029781	0.04386
				594.9	0.026321	0.029712	0.043967
				597.408	0.026283	0.029644	0.044072

				599.917	0.026246	0.029576	0.044178
				602.425	0.026208	0.029508	0.044284
				604.933	0.026171	0.02944	0.044389
				607.442	0.026133	0.029373	0.044494
				609.95	0.026096	0.029305	0.044599
				612.458	0.026059	0.029238	0.044703
				614.967	0.026021	0.029171	0.044808
				617.475	0.025984	0.029104	0.044912
				619.983	0.025947	0.029038	0.045016
				622.492	0.02591	0.028971	0.045119
				625	0.025873	0.028905	0.045223
				627.508	0.025836	0.028838	0.045326
				630.017	0.025799	0.028772	0.045429
				632.525	0.025762	0.028707	0.045531
				635.033	0.025725	0.028641	0.045634
				637.542	0.025689	0.028575	0.045736
				640.05	0.025652	0.02851	0.045838
				642.554	0.025615	0.028445	0.04594
				645.058	0.025579	0.02838	0.046041
				647.563	0.025542	0.028316	0.046142
				650.067	0.025506	0.028251	0.046243
				652.575	0.025469	0.028187	0.046344
				655.083	0.025433	0.028122	0.046445
				657.592	0.025397	0.028058	0.046545
				660.1	0.025361	0.027994	0.046645
				662.608	0.025324	0.02793	0.046745
				665.117	0.025288	0.027867	0.046845
				667.625	0.025252	0.027803	0.046945
				670.133	0.025216	0.02774	0.047044
				672.642	0.02518	0.027677	0.047143
				675.15	0.025144	0.027614	0.047242
				677.658	0.025108	0.027551	0.047341
				680.167	0.025073	0.027488	0.047439
				682.675	0.025037	0.027426	0.047537
				685.183	0.025001	0.027364	0.047635
				687.692	0.024966	0.027301	0.047733
				690.2	0.02493	0.027239	0.047831
				692.708	0.024894	0.027178	0.047928
				695.217	0.024859	0.027116	0.048025
				697.725	0.024824	0.027054	0.048122
				700.233	0.024788	0.026993	0.048219
				702.742	0.024753	0.026932	0.048315
				705.25	0.024718	0.026871	0.048412
				707.758	0.024683	0.02681	0.048508

				710.267	0.024647	0.026749	0.048604
				712.775	0.024612	0.026689	0.048699
				715.283	0.024577	0.026628	0.048795
				717.792	0.024542	0.026568	0.04889
				720.3	0.024507	0.026508	0.048985
				722.808	0.024472	0.026448	0.04908
				725.317	0.024438	0.026388	0.049174
				727.825	0.024403	0.026328	0.049269
				730.333	0.024368	0.026269	0.049363
				732.837	0.024334	0.02621	0.049457
				735.342	0.024299	0.026151	0.04955
				737.846	0.024265	0.026092	0.049644
				740.35	0.02423	0.026033	0.049737
				742.858	0.024196	0.025974	0.04983
				745.367	0.024161	0.025916	0.049923
				747.875	0.024127	0.025857	0.050016
				750.383	0.024093	0.025799	0.050108
				752.892	0.024058	0.025741	0.050201
				755.4	0.024024	0.025683	0.050293
				757.908	0.02399	0.025625	0.050385
				760.417	0.023956	0.025568	0.050477
				762.925	0.023922	0.02551	0.050568
				765.433	0.023888	0.025453	0.050659
				767.942	0.023854	0.025396	0.050751
				770.45	0.02382	0.025338	0.050841
				772.958	0.023786	0.025282	0.050932
				775.467	0.023753	0.025225	0.051023
				777.975	0.023719	0.025168	0.051113
				780.483	0.023685	0.025112	0.051203
				782.992	0.023652	0.025055	0.051293
				785.5	0.023618	0.024999	0.051383
				788.008	0.023585	0.024943	0.051472
				790.517	0.023551	0.024887	0.051562
				793.025	0.023518	0.024832	0.051651
				795.533	0.023484	0.024776	0.05174
				798.042	0.023451	0.02472	0.051829
				800.55	0.023418	0.024665	0.051917
				803.058	0.023385	0.02461	0.052006
				805.567	0.023352	0.024555	0.052094
				808.075	0.023318	0.0245	0.052182
				810.583	0.023285	0.024445	0.05227
				813.087	0.023252	0.024391	0.052357
				815.592	0.02322	0.024336	0.052444
				818.096	0.023187	0.024282	0.052531

				820.6	0.023154	0.024228	0.052618
				823.108	0.023121	0.024174	0.052705
				825.617	0.023088	0.02412	0.052792
				828.125	0.023056	0.024066	0.052878
				830.633	0.023023	0.024013	0.052965
				833.142	0.02299	0.023959	0.053051
				835.65	0.022958	0.023906	0.053137
				838.158	0.022925	0.023853	0.053222
				840.667	0.022893	0.023799	0.053308
				843.175	0.022861	0.023746	0.053393
				845.683	0.022828	0.023694	0.053478
				848.192	0.022796	0.023641	0.053563
				850.7	0.022764	0.023588	0.053648
				853.208	0.022731	0.023536	0.053733
				855.717	0.022699	0.023484	0.053817
				858.225	0.022667	0.023432	0.053901
				860.733	0.022635	0.02338	0.053985
				863.242	0.022603	0.023328	0.054069
				865.75	0.022571	0.023276	0.054153
				868.258	0.022539	0.023224	0.054237
				870.767	0.022507	0.023173	0.05432
				873.275	0.022476	0.023121	0.054403
				875.783	0.022444	0.02307	0.054486
				878.292	0.022412	0.023019	0.054569
				880.8	0.02238	0.022968	0.054651
				883.308	0.022349	0.022917	0.054734
				885.817	0.022317	0.022867	0.054816
				888.325	0.022286	0.022816	0.054898
				890.833	0.022254	0.022766	0.05498
				893.337	0.022223	0.022715	0.055062
				895.842	0.022192	0.022665	0.055143
				898.346	0.02216	0.022615	0.055224
				900.85	0.022129	0.022566	0.055306
				903.358	0.022098	0.022516	0.055387
				905.867	0.022067	0.022466	0.055467
				908.375	0.022036	0.022416	0.055548
				910.883	0.022004	0.022367	0.055629
				913.392	0.021973	0.022318	0.055709
				915.9	0.021942	0.022269	0.055789
				918.408	0.021911	0.02222	0.055869
				920.917	0.021881	0.022171	0.055949
				923.425	0.02185	0.022122	0.056029
				925.933	0.021819	0.022073	0.056108
				928.442	0.021788	0.022025	0.056187

				930.95	0.021757	0.021976	0.056267
				933.458	0.021727	0.021928	0.056346
				935.967	0.021696	0.02188	0.056424
				938.475	0.021666	0.021832	0.056503
				940.983	0.021635	0.021784	0.056581
				943.492	0.021605	0.021736	0.05666
				946	0.021574	0.021688	0.056738
				948.508	0.021544	0.021641	0.056816
				951.017	0.021513	0.021593	0.056894
				953.525	0.021483	0.021546	0.056971
				956.033	0.021453	0.021499	0.057049
				958.542	0.021423	0.021451	0.057126
				961.05	0.021392	0.021404	0.057203
				963.558	0.021362	0.021358	0.05728
				966.067	0.021332	0.021311	0.057357
				968.575	0.021302	0.021264	0.057434
				971.083	0.021272	0.021218	0.05751
				973.592	0.021242	0.021171	0.057586
				976.1	0.021213	0.021125	0.057663
				978.608	0.021183	0.021079	0.057739
				981.117	0.021153	0.021033	0.057814
				983.621	0.021123	0.020987	0.05789
				986.125	0.021094	0.020941	0.057965
				988.629	0.021064	0.020896	0.05804
				991.133	0.021034	0.02085	0.058116
				993.642	0.021005	0.020805	0.058191
				996.15	0.020975	0.020759	0.058265
				998.658	0.020946	0.020714	0.05834
				1001.17	0.020916	0.020669	0.058415
				1003.68	0.020887	0.020624	0.058489
				1006.18	0.020858	0.020579	0.058563
				1008.69	0.020828	0.020534	0.058637
				1011.2	0.020799	0.02049	0.058711
				1013.71	0.02077	0.020445	0.058785
				1016.22	0.020741	0.020401	0.058859
				1018.72	0.020712	0.020356	0.058932
				1021.23	0.020682	0.020312	0.059005
				1023.74	0.020653	0.020268	0.059079
				1026.25	0.020624	0.020224	0.059152
				1028.76	0.020595	0.02018	0.059224
				1031.27	0.020567	0.020136	0.059297
				1033.78	0.020538	0.020093	0.05937
				1036.28	0.020509	0.020049	0.059442
				1038.79	0.02048	0.020006	0.059514

				1041.3	0.020451	0.019963	0.059586
				1041.3	0.020451	0.019963	0.059586
